# Fecal microbiome alterations in treatment-naive de novo Parkinson’s disease

**DOI:** 10.1038/s41531-022-00395-8

**Published:** 2022-10-10

**Authors:** Jeffrey M. Boertien, Kirsi Murtomäki, Pedro A. B. Pereira, Sygrid van der Zee, Tuomas H. Mertsalmi, Reeta Levo, Tanja Nojonen, Elina Mäkinen, Elina Jaakkola, Pia Laine, Lars Paulin, Eero Pekkonen, Valtteri Kaasinen, Petri Auvinen, Filip Scheperjans, Teus van Laar, PPNN Study Group, N. A. Verwey, B. van Harten, A. T. Portman, M. J. H. Langedijk, P. G. Oomes, B. J. A. M. Jansen, T. van Wieren, S. J. A. van den Bogaard, W. van Steenbergen, R. Duyff, J. P. van Amerongen, P. S. S. Fransen, S. K. L. Polman, R. T. Zwartbol, M. E. van Kesteren, J. P. Braakhekke, J. Trip, L. Koops, C. J. de Langen, G. de Jong, J. E. S. Hartono, H. Ybema, A. L. Bartels, F. E. Reesink, A. G. Postma, G. J. H. Vonk, J. M. T. H. Oen, M. J. Brinkman, T. Mondria, R. S. Holscher, A. A. E. van der Meulen, A. W. F. Rutgers, W. A. Boekestein, L. K. Teune, P. J. L. Orsel, J. E. Hoogendijk, T. van Laar

**Affiliations:** 1 Department of Neurology, University Medical Center Groningen, University of Groningen, Groningen, the Netherlands; 2 Department of Neurology, Helsinki University Hospital and Clinicum, University of Helsinki, Helsinki, Finland; 3 Institute of Biotechnology, DNA Sequencing and Genomics Laboratory, University of Helsinki, Helsinki, Finland; 4 Clinical Neurosciences, University of Turku and Neurocenter, Turku University Hospital, Turku, Finland; 5 Department of Neurology, Medisch Centrum Leeuwarden, Leeuwarden, the Netherlands; 6 Department of Neurology, Treant Zorggroep locations, Stadskanaal, Emmen, Hoogeveen, the Netherlands; 7 Department of Neurology, Tjongerschans Ziekenhuis Heerenveen, Heerenveen, the Netherlands; 8 Department of Neurology, Isala, Zwolle, Meppel the Netherlands; 9 Department of Neurology, Ommelander Ziekenhuis Groningen, Scheemda, the Netherlands; 10 Department of Neurology, Nij Smellinghe Ziekenhuis Drachten, Drachten, the Netherlands; 11 Department of Neurology, Antonius Zorggroep, Sneek, the Netherlands; 12 Department of Neurology, Martini Ziekenhuis, Groningen, the Netherlands; 13 Department of Neurology, Wilhelmina Ziekenhuis Assen, Assen, the Netherlands; 14 Department of Neurology, Sionsberg, Dokkum, the Netherlands; 15 Department of Neurology, University Medical Center Groningen, Groningen, the Netherlands

**Keywords:** Parkinson's disease, Parkinson's disease

## Abstract

Gut microbiota alterations in Parkinson’s disease (PD) have been found in several studies and are suggested to contribute to the pathogenesis of PD. However, previous results could not be adequately adjusted for a potential confounding effect of PD medication and disease duration, as almost all PD participants were already using dopaminergic medication and were included several years after diagnosis. Here, the gut microbiome composition of treatment-naive de novo PD subjects was assessed compared to healthy controls (HC) in two large independent case-control cohorts (*n* = 136 and 56 PD, *n* = 85 and 87 HC), using 16S-sequencing of fecal samples. Relevant variables such as technical batches, diet and constipation were assessed for their potential effects. Overall gut microbiome composition differed between PD and HC in both cohorts, suggesting gut microbiome alterations are already present in de novo PD subjects at the time of diagnosis, without the possible confounding effect of dopaminergic medication. Although no differentially abundant taxon could be replicated in both cohorts, multiple short chain fatty acids (SCFA) producing taxa were decreased in PD in both cohorts. In particular, several taxa belonging to the family Lachnospiraceae were decreased in abundance. Fewer taxonomic differences were found compared to previous studies, indicating smaller effect sizes in de novo PD.

## Introduction

Parkinson’s disease (PD) is a neurodegenerative disorder, clinically characterized by motor symptoms like bradykinesia, rigidity, and tremor^1^. Non-motor symptoms can precede the cardinal motor symptoms by years^2^. In particular, constipation is one of the earliest non-motor manifestations of PD and can occur up to twenty years before diagnosis^3^. The occurrence of non-motor symptoms can be used to identify probable prodromal PD subjects and predict conversion to motor PD^4^. Complementary to the early gastrointestinal symptomatology, alpha-synuclein (aSyn) pathology, gut inflammation and increased gut permeability are present in the early and prodromal stages of the disease^5,6^. In addition, ascending denervation along the vagal nerve is suggested in a subgroup of PD subjects, suggesting a possible gastrointestinal origin^7^.

The relation between gut health and PD has led to an increased interest in the putative role of the gut microbiome in PD. Several preclinical studies found evidence that gut microbiota may impact PD pathology via for instance cross-seeding of aSyn or inflammatory signaling through microbial metabolites, such as short chain fatty acids (SCFA)^8–10^. In addition, over fifteen case-control studies have found alterations in gut microbiome composition in PD^11–15^. Though some associations could be robustly replicated, various results were inconsistent across several studies, in part due to differences in study population and methodology.

Of particular concern is the fact that almost all participants of previous studies were included several years after diagnosis and were already using dopaminergic medication^11^. Therefore, a possible PD-related effect cannot adequately be distinguished from a potential confounding effect of the dopaminergic medication. Levels of Lachnospiraceae, *Bifidobacterium* and *Lactobacillus* have indeed been correlated to levodopa dosage^14,16,17^. Additionally, a recent mendelian randomization study suggested the association between PD and *Bifidobacterium* to be based on reverse causation^18^. Also, Catechol-O-methyltransferase (COMT) inhibitors have been associated with gut microbiome changes^17^. Moreover, possible microbial biomarkers cannot be assessed for their potential as a diagnostic marker, as data at the time of diagnosis is lacking, and longer disease duration is associated with larger differences in gut microbiome composition in PD^19^.

In addition to established PD cases, gut microbiome changes have been described in probable prodromal PD subjects, with two studies reporting changes comparable to PD, and a third study linking gut microbiome changes to specific prodromal symptoms rather than overall prodromal risk^20–22^. Polysomnography proven REM sleep behavior disorder (RBD) is the prodromal symptom with the highest predictive value for conversion to PD^23^. The recently proposed dichotomy of body-first and brain-first PD suggests a possible gastrointestinal (body-first) origin of PD in subjects with RBD at the time of diagnosis and during the prodromal stages of the disease^7^. Moreover, RBD is proposed to co-occur with other prodromal autonomic symptoms, such as constipation^24^. Therefore, results found in probable prodromal subjects might only be representative of PD subjects with a possible gastrointestinal site-of-origin.

There is still a large unmet need to assess the gut microbiome of treatment-naive de novo PD subjects at the time of diagnosis, without the confounding effect of dopaminergic medication. Currently, the largest gut microbiome assessment of a treatment-naive de novo PD cohort consists of a subcohort of 39 subjects by Barichella et al^19^. Compared to already treated, more advanced PD subjects, the gut microbiome composition was less different from that of HC and only showed the family Lachnospiraceae and two of its genera (*Roseburia* and an unclassified genus) to be differentially abundant compared to HC.

The current gut microbiome study concerns two large, independent, treatment-naive cohorts from the Netherlands (NL cohort) and Finland (FIN cohort). The gut microbiome of fecal samples was investigated using 16S rRNA gene sequencing. Both cohorts comprise both PD and HC subjects from the same geographical area. Both cohorts were analyzed separately due to their different geographical origin and the use of different methodologies for stool sample collection and DNA extraction. Relevant variables, such as technical batches, diet and constipation, were assessed for their potential effects on the relationship between PD status and gut microbiome composition. Subsequently, a thorough investigation of the gut microbiome of two large treatment-naive de novo PD cohorts could be conducted.

## Results

### Clinical characteristics

For the NL cohort, 136 PD subjects and 85 HC could be included. The PD and HC groups were similar in terms of age and BMI, but differed in terms of the sex ratio (*p* = 0.026). In the FIN cohort 56 PD subjects and 87 HC were included. There was no statistically significant difference in age, sex, and BMI between the FIN cohort PD and HC groups. As expected, gastrointestinal dysfunction was higher in PD compared to HC, with lower Bristol Stool Chart scores for stool consistency and lower stool frequency, indicating harder stool consistency and increased levels of constipation. However, these associations were statistically significant only in the NL cohort. An overview of the clinical characteristics is provided in Table 1.

**Table 1:** Clinical characteristics.

	NL cohort			FIN cohort		
	PD	HC	*p*-value	PD	HC	*p*-value
*n*	136	85	—	56	87	—
Age, mean ± sd	66.0 ± 8.86	65.9 ± 9.69	0.91	64.9 ± 10.77	67.1 ± 7.3	0.18
Female, *n*(%)	41 (30%)	39 (46%)	0.026	27 (48%)	43 (49%)	1.0
BMI, mean ± sd	26.5 ± 4.46	26.1 ± 3.73	0.52	26.3 ± 5.62	26.6 ± 3.98	0.76
Stool consistency, mean ± sd	3.40 ± 0.94	3.85 ± 0.85	3.5E-04	3.21 ± 0.87	3.42 ± 0.81	0.15
Stool frequency, median [IQR]	1 [0.82–1.29]	1.17 [1–1.73]	2.0E-05	1 [0.86–1.57]	1.14 [1–1.57]	0.13
Gastric anacidic medication, *n*(%)	32 (24%)	14 (16%)	8.0E-03	8 (14%)	3 (3%)	0.025
Constipation medication, *n*(%)	16 (12%)	1 (1%)	2.7E-04	6 (11%)	7 (8%)	0.77
MDS-UPDRSIII total, mean ± sd	31.8 ± 11.63	—	—	33.8 ± 13.03	—	—
Hoehn and Yahr, median [IQR]	2 [1–2]	—	—	2 [1–2]	—	—
Motor symptom duration in months, mean ± sd	21.6 ± 14.36	—	—	20.9 ± 14.29	—	—

NL cohort, Dutch case-control cohort; FIN cohort, Finnish case-control cohort; PD, Parkinson’s disease; HC, healthy control; p-value, nominal p-value; n, number of participants; sd, standard deviation; BMI, body mass index; stool consistency, average stool consistency score according to the Bristol Stool Chart; stool frequency, average stool frequency per day; IQR, interquartile range; MDS-UPDRS, Movement Disorders Society Unified Parkinson’s Disease Rating Scale; motor symptom duration in months estimated retrospectively by participants counting.

### Variable selection

Given the primary aim to describe gut microbiome changes for the first time in treatment-naive de novo PD subjects, all clinical and technical metadata were investigated for their potential to influence the effect of PD status on gut microbiome composition. The variables age, sex, BMI, stool consistency and DNA extraction batch were selected a priori as potentially relevant nuisance variables. To ensure a large effect of another variable on the relation between PD status and gut microbiome composition would not be missed, all nuisance variables were screened for their potential effect using a PERMANOVA in a model with PD status:

- distance-matrix ~ PD status + variable.

Variables that shifted the explained variance of PD status (R2) by at least ten percent compared to the univariable explained variance of PD status (distance-matrix ~ PD status), were added to the list of relevant model covariates (Supplementary Table 3). Subsequently, the selection of relevant covariates was investigated for collinearity with PD status. Variables with a generalized variance inflation factor (GVIF) ≥ 2 with PD status were excluded. In the overall model, a GVIF of 3 was used as threshold for collinearity, excluding variables with a higher GVIF or one of several variables that only showed collinearity amongst each other (Supplementary Table 4). This resulted in the following models for the multivariate analysis of overall gut microbiome composition.

For the NL cohort, model (1): distance-matrix ~ PD status + age + sex + BMI + stool consistency + NMSQ constipation + stool frequency + DNA extraction batch

For the FIN cohort, model (2): distance-matrix ~ PD status + age + sex + BMI + stool consistency + CSI total score + strained defecation (stool control) + number of sequences

For the differential abundance analysis, covariates were selected that showed a significant relationship with the overall gut microbiome composition in the final model with non-stringent *p* < 0.1, resulting in the following two models.

For the NL cohort, model (3): taxon ~ PD status + age + BMI + stool consistency + stool frequency

For the FIN cohort, model (4): taxon ~ PD status + stool consistency + number of sequences

### Overall microbiome composition

**Fig. 1:**
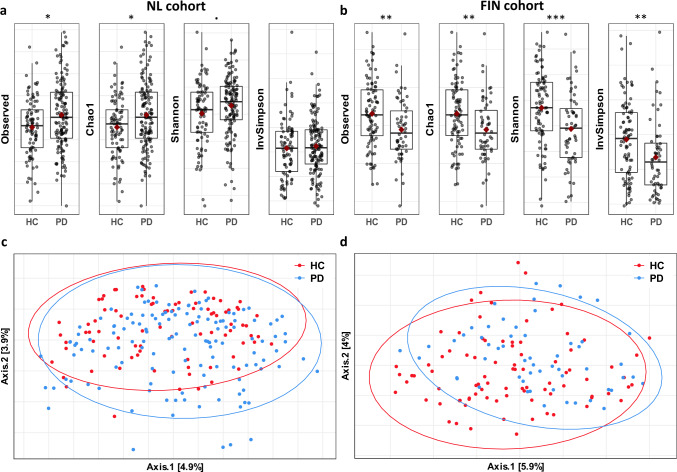
Intra-sample (alpha diversity) and inter-sample differences in microbial community structure between PD and HC. **a** Alpha diversity indices indicate increased intra-sample diversity in PD in the NL cohort, whereas in **b** intra-sample diversity in PD is reduced in the FIN cohort. Each box represents the first quartile, median and third quartile at the lower, middle and upper boundaries, with the whiskers representing points within 1.5 times the interquartile range and the red diamond representing the mean. Each point represents one sample. Univariable, uncorrected (Mann-Whitney U test) *p*-values are 0.016, 0.016, 0.060 and 0.56 for the NL cohort and 4.3E-03, 4.2E-03, 7.8E-04, and 3.9E-03 for the FIN cohort for, respectively, observed richness, Chao1, Shannon and Inverse Simpson. **c**, **d** Inter-sample differences in microbial community structure were visualized using a principal component analysis of the Aitchinson distance with each red point representing one HC sample and each blue point representing one PD sample. A statistically significant difference was found between PD and HC with p = 6.4E-03 in the NL cohort **c**, and *p* = 4.0E-04 in the FIN cohort **d**. Sample sizes: 136 PD and 85 HC for the NL cohort and 56 PD and 87 HC in the FIN cohort. *p* < 0.1; *p < 0.05; **p < 0.01; ***p < 0.001; NL cohort, Dutch case-control cohort; FIN cohort, Finnish case-control cohort; PD, Parkinson’s disease; HC, healthy control.

Within-sample diversity (i.e. alpha diversity) indices indicated opposite effects in the two cohorts (Fig. 1a). In the NL cohort, alpha diversity was higher in PD subjects with statistically significant differences in observed richness and Chao1 (nominal *p*-values < 0.05, uncorrected Mann-Whitney U test) and a statistically non-significant increase in the Shannon alpha diversity index. In contrast, alpha diversity was lower in the FIN cohort, with statistically significant differences in observed richness, Chao1, Shannon and inverse Simpson (nominal *p*-values < 0.01, uncorrected Mann-Whitney U test).

Overall gut microbiome composition (i.e. beta diversity) showed a large overlap between PD and HC (Fig. 1b). PERMANOVA revealed univariable statistically significant differences between PD and HC in both cohorts: *p* = 6.4E-03 and *p* = 4.0E-04 for the NL and FIN cohort respectively (Table 2). After adjusting for selected covariates (see variable selection) a statistically significant difference between PD and HC remained, with *p* = 0.030 and *p* = 0.016 for the NL and FIN cohort respectively. When assessing differences in overall gut microbiome, using (non-compositional) Bray-Curtis distances, the results were similar, although no statistically significant difference between PD and HC was found in the NL cohort when adjusting for covariates (*p* = 0.17).

**Table 2:** Comparisons of overall gut microbiome composition between PD and HC.

	NL cohort				FIN cohort			
	Aitchinson		Bray-Curtis		Aitchinson		Bray-Curtis	
	var %	*p*-value	var %	*p*-value	var %	*p*-value	var %	*p*-value
Univariable								
group	0.62	6.4E-03	0.74	9.1E-03	1.18	4.0E-04	1.29	1.1E-03
Adjusted for confounders								
group	0.57	0.030	0.54	0.17	0.92	0.016	1.03	0.020
age	0.70	4.0E-04	0.90	3.0E-04	0.63	0.85	0.64	0.66
sex	0.49	0.25	0.61	0.067	0.77	0.17	0.68	0.55
BMI	0.81	<1.0E-04	0.99	3.0E-04	0.77	0.18	0.81	0.21
stoolconsistency	0.56	0.033	0.62	0.050	0.83	0.073	0.82	0.19
NMSQ constipation	0.52	0.10	0.55	0.15	—	—	—	—
stoolfrequency	0.57	0.027	0.62	0.056	—	—	—	—
DNA extraction batch	6.50	0.37	6.72	0.26	—	—	—	—
CSI total score	—	—	—	—	0.74	0.27	0.74	0.36
Strained defecation	—	—	—	—	0.69	0.52	0.66	0.61
Number of sequences	—	—	—	—	0.87	0.034	0.77	0.30

Differences in overall gut microbiome composition between PD and HC were assessed in a univariable analysis, and in a multivariable analysis with adjustment for selected variables. Each variable was adjusted for all other variables in the multivariable analysis (marginal testing). In addition to Aitchinson distances, the analyses were also performed with Bray-Curtis distances. Sample sizes for the univariable analyses were 136 PD and 85 HC for the NL cohort and 56 PD and 87 HC in the FIN cohort. Sample sizes for the multivariable analyses were 131 PD and 84 HC for the NL cohort and 54 PD and 87 HC for the FIN cohort. For the analyses based on Bray-Curtis distances, four additional PD and one additional HC sample were excluded from the NL cohort and one additional PD sample was excluded from the FIN cohort, due to insufficient number of sequences after rarefaction. P-values were based on 10,000 permutations, making the lowest possible p-value 9.999E-05, written as <1.0E-04 in the above table.

NL cohort, Dutch case-control cohort; FIN cohort, Finnish case-control cohort; PD, Parkinson’s disease; HC, healthy control; BMI, body mass index; NMSQ, Non-Motor Symptom Questionnaire; CSI, Constipation Severity Instrument; strained defecation, level of straining during defecation measured in FIN cohort stool diary; number of sequences, number of sequences per sample.

### Differential abundance

**Fig. 2:**
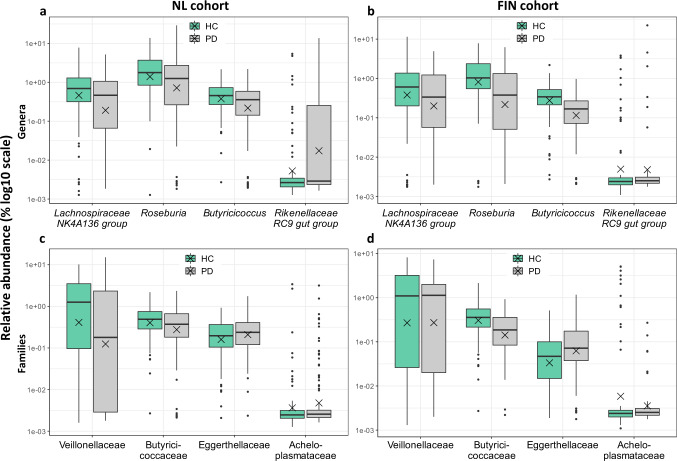
Relative abundances of genera and families identified as differentially abundant in at least one of the two cohorts. Relative abundances of genera identified with DESeq2 and/or ANCOM in at least one of the two cohorts are depicted in **a** for the NL cohort and **b** for the FIN cohort. Relative abundances of families identified with DESeq2 and/or ANCOM in at least one of the two cohorts are depicted in **c** for the NL cohort and **d** for the FIN cohort. Each box represents the first quartile, median and third quartile at the lower, middle, and upper boundaries, with the whiskers representing points within 1.5 times the interquartile range and the X representing the mean for HC (green) and PD (gray). Sample sizes: 131 PD and 84 HC subjects for the NL cohort and 55 PD and 87 HC subjects for the FIN cohort. NL cohort, Dutch case-control cohort; FIN cohort, Finnish case-control cohort; PD, Parkinson’s disease; HC, healthy control; DESeq2, Differential Expression analysis for Sequence count data; ANCOM, ANalysis of Composition of Microbiomes.

Differential abundance analyses revealed several statistically significant differentially abundant taxa at the ASV, genus and family level (Table 3). Only one taxon at the ASV level, belonging to the Lachnospiraceae *GCA-900066575* genus, was identified as differentially abundant by both ANCOM and DESeq2 in the FIN cohort (Supplementary Table 5). None of the differentially abundant taxa were shared between the NL and FIN cohort. However, some similarity between the two cohorts can be discerned. Both at the ASV and genus level several taxa belonging to the Lachnospiraceae family were reduced in abundance in PD, in particular strains belonging to the Lachnospiraceae genus *Roseburia*. At the family level, Lachnospiraceae was reduced in PD in both cohorts, although this difference was not statistically significant after correction for multiple testing in DESeq2 nor identified as differentially abundant in ANCOM (Supplementary Table 7). Relative abundances of genera and families that were identified as statistically significant differentially abundant in one of the two cohorts, are depicted in Fig. 2.

**Table 3:** Differentially abundant taxa between PD and HC.

				NL cohort					FIN cohort				
Level	Family	Genus	Species	baseMean	log2 Fold Change	DESeq2 (FDR)	ANCOM (W)	ANCOM significant	baseMean	log2 Fold Change	DESeq2 (FDR)	ANCOM (W)	ANCOM significant
**ASV**	Lachnospiraceae	Lachnospiraceae NK4A136 group	—	74.61	−0.76	1.0	285	**yes**	102.99	0.07	1.0	0	no
**ASV**	Lachnospiraceae	Lachnoclostridium	—	90.76	−0.82	1.0	313	**yes**	99.74	−0.66	1.0	216	no
**ASV**	Lachnospiraceae	Roseburia	—	100.23	−0.48	1.0	274	**yes**	15.64	−2.92	0.24	207	no
**ASV**	Lachnospiraceae	Roseburia	inulinivorans	266.76	−0.14	1.0	153	no	207.61	−1.16	0.74	258	**yes**
**ASV**	Lachnospiraceae	Lachnoclostridium	edouardi	83.60	−0.48	1.0	0	no	83.08	−0.96	0.97	252	**yes**
**ASV**	Lachnospiraceae	Lachnospiraceae UCG-004	—	39.01	−0.33	1.0	0	no	65.47	−1.09	0.97	252	**yes**
**ASV**	Lachnospiraceae	GCA-900066575	—	17.16	−0.23	1.0	0	no	14.32	−3.50	**2.0E-03**	274	**yes**
**ASV**	Oscillospiraceae	Colidextribacter	—	10.45	0.24	1.0	0	no	16.60	−1.27	0.97	248	**yes**
**ASV**	Christensenellaceae	Christensenellaceae R-7 group	—	8.41	1.19	1.0	6	no	23.85	1.61	0.89	279	**yes**
**ASV**	Clostridia UCG-014 (order)^1^	—	—	138.12	−5.11	**0.049**	0	no	86.72	1.06	1.0	0	no
**ASV**	Akkermansiaceae	Akkermansia	muciniphila^2^	—	—	—	—	—	68.26	11.12	**2.8E-07**	141	no
**Genus**	Lachnospiraceae	Lachnospiraceae NK4A136 group	—	367.11	−0.43	0.92	88	**yes**	407.63	−0.33	0.98	0	no
**Genus**	Rikenellaceae	Rikenellaceae RC9 gut group	—	157.67	2.41	0.41	89	**yes**	136.94	0.79	0.98	0	no
**Genus**	Lachnospiraceae	Roseburia	—	896.86	−0.14	0.94	73	no	588.97	−0.50	0.88	82	**yes**
**Genus**	Butyricicoccaceae	Butyricicoccus	—	187.67	−0.21	0.92	66	no	147.39	−0.95	**0.038**	49	no
**Family**	Veillonellaceae	—	—	842.30	−0.55	0.96	42	**yes**	715.05	−0.29	0.75	0	no
**Family**	Butyricicoccaceae	—	—	221.57	−0.26	0.96	18	no	164.48	−0.98	**2.7E-03**	22	no
**Family**	Acholeplasmataceae	—	—	23.36	−1.18	0.96	0	no	81.04	−5.69	**0.033**	0	no
**Family**	Eggerthellaceae	—	—	120.86	−0.06	0.96	0	no	38.92	1.08	**0.022**	9	no

Differentially abundant taxa between PD and HC detected using DESeq2 and/or ANCOM in at least one of the two cohorts. Both analyses were adjusted for variables with p < 0.1 in the comparison of overall gut microbiome compositions (age, BMI, stool consistency and stool frequency in the NL cohort; stool consistency and number of reads in the FIN cohort). Only samples for which complete metadata concerning the selected confounders was available, were included in the differential abundance analyses, resulting in 131 PD and 84 HC subjects for the NL cohort and 55 PD and 87 HC subjects for the FIN cohort. (1) One ASV in the order Clostridia UCG-014 was identified in the NL cohort, but was not classified at lower taxonomic levels. (2) The ASV identified in the FIN cohort belonging to *Akkermansia muciniphila* was not present in the NL cohort.

NL cohort, Dutch case-control cohort; FIN cohort, Finnish case-control cohort; PD, Parkinson’s disease; HC, healthy control; BMI, body mass index; baseMean, mean of all normalized counts provided by DESeq2; log2 Fold Change, a negative change indicates lower abundance in PD compared to HC; DESeq2, Differential Expression analysis for Sequence count data; FDR, False Discovery Rate corrected p-value; ANCOM, ANalysis of Composition of Microbiomes; W, W statistic indicating the number of taxa relative to which the presented taxon was differentially abundant (maximum values for W were 371, 124 and 45 for the NL cohort and 354, 113 and 46 for the FIN cohort at the ASV, Genus and Family level, respectively); ANCOM significant, indicates whether the taxon is detected as significantly differentially abundant using a detection threshold of 0.7; ASV, Amplicon Sequence Variant.

### DNA extraction methods

Samples were randomized as much as possible during the DNA extraction and library preparation and were all sequenced together to minimize batch effects (see Methods). Nonetheless, as the two different cohorts were initiated independently, different methods of sample collection and DNA extraction were used in the two cohorts. Samples from the NL cohort were collected without preservative, frozen immediately and processed using the Qiagen Allprep DNA extraction kit. In the FIN cohort, samples were collected using a DNA preservative and shipped via postal service. DNA extraction was performed using the PSP Spin Stool Kit. To assess the influence of the different methodologies, 47 stool samples from the NL cohort were collected and processed using both the NL method (Qiagen Allprep) and FIN method (PSP Spin Stool Kit) in parallel, taking one aliquot with each method from the same fecal material. Multivariate analysis yielded a statistically significant difference in overall microbiome composition between the two DNA extraction methods (PERMANOVA, *p* = 9.999E-05). Several taxa at various taxonomic levels were identified as differentially abundant. Both ANCOM and DESeq2 identified various taxa belonging to the Lachnospiraceae family as less abundant when using the FIN extraction protocol, whereas DESeq2 also identified several taxa belonging to the order of Bacteroidales as more abundant when using the FIN protocol (Supplementary Table 8).

### Same household control subjects

Contrary to the FIN cohort, the NL cohort consisted of a substantial proportion of HC from the same household as a PD participant (*n* = 46). Similarity between same household participants was assessed by stratifying the NL cohort according to household status. First, 45 PD subjects and 45 HC, of which each case-control pair is from the same household, were compared. Second, the same 45 HC were compared to 45 PD subjects who did not have someone from their household participate as HC in the NL cohort, matched according to age and sex. Though the overall microbiome composition of age- and sex-matched PD subjects from a different household was more distant from that of the HC compared to the overall microbiome composition of non-matched PD subjects from the same household, this did not lead to the discovery of more differentially abundant taxa (Supplementary Table 9).

## Discussion

Here, we have presented the results of the two largest gut microbiome studies to date with treatment-naive de novo PD subjects, close to the time of diagnosis. Both cohorts showed alterations in gut microbiome composition of fecal samples in PD after adjustment for relevant covariates. In concordance, several taxa at the ASV, genus and family level were identified as differentially abundant between PD and HC. Though none of the differentially abundant taxa could be directly replicated in both cohorts, both cohorts showed several ASVs and genera belonging to the family of Lachnospiraceae to be reduced in PD, in particular strains of the genus *Roseburia*.

Previous gut microbiome studies in PD have consistently found differences in overall gut microbiome composition between PD and HC^11–14^. The current study confirms the presence of gut microbiome alterations in treatment-naive de novo PD. The contradictory findings on within-sample diversity (alpha diversity) can also be considered in line with the existing body of literature, with different studies finding either an increased or decreased alpha diversity in PD^11^. Recently, a meta-analysis of previously published PD microbiome data concluded that differences in alpha diversity are not a marker of PD^25^.

Regarding the differentially abundant taxa, decreased levels of Lachnospiraceae and several of its taxa have been replicated in several PD microbiome studies^11,14,15,19^. In addition, decreased levels of Lachnospiraceae and its genera have also been correlated to PD medication (eg. *UCG-004* and *Roseburia*), more depressive symptoms (eg. *Roseburia*), more advanced disease stage and worse motor symptoms^14,19,26^. In our study several ASVs belonging to the genus *Roseburia*, as well as other genera belonging to the family Lachnospiraceae, were already decreased at the time of diagnosis before treatment initiation. This is in line with previous findings of reduced levels of Lachnospiraceae and two of its genera, including *Roseburia*, in a subcohort of 39 untreated *de novo* PD subjects, suggesting reduced levels of Lachnospiraceae are at least in part independent of PD medication and more advanced disease stage^19^. Lachnospiraceae produce short chain fatty acids (SCFAs), known for their anti-inflammatory properties^27^. Several studies have linked lower levels of SCFA-producing taxa with gut inflammation, epigenetic changes, and depressive symptoms in PD^26,28–30^. In concordance, decreased levels of the SCFA-producing genus *Butyricoccus*, the families Butyricoccaceae and Veillonellaceae and an ASV within the genus *Colidextribacter* were found in the current study. On the other hand, the SCFA-producing family Rikenellaceae was increased in PD. Several taxa belonging to the class Clostridia also produce SCFAs and are inversely related to gut barrier dysfunction^31^. One ASV in the order Clostridia UCG-014 was decreased in PD in the NL cohort, but was not classified at lower taxonomic levels. Therefore, it is unclear whether this specific ASV is associated with SCFA production or gut wall integrity. Levels of the non-SCFA-producing families Eggerthellaceae and Acholeplasmataceae were respectively increased and decreased in one of the two cohorts. To our knowledge, Eggerthellaceae and Acholeplasmataceae have not been identified as differentially abundant in previous human PD microbiome studies. Also, an ASV belonging to the non-SCFA producing family Christensenellaceae was increased. Increased levels of Christensenellaceae have been found in previous PD microbiome studies and seem in particular related to increased gut transit times^32^. Increased levels of *Bifidobacterium* and *Lactobacillus* were robustly replicated in several previous PD microbiome studies, but were hypothesized to be related to dopaminergic medication use^14,18^. In accordance, no increased levels of *Bifidobacterium* were found in either cohort (NL cohort: reduced, nominal *p* = 0.07; FIN cohort: increased, nominal *p* = 0.14, Supplementary Table 6) nor of *Lactobacillus* (NL cohort: reduced, nominal *p* = 0.71; FIN cohort: increased, nominal *p* = 0.76, Supplementary Table 6). Increased levels of the genus *Akkermansia* is one of the most replicated taxonomic differences between PD and HC^11,15^. In addition, *Akkermansia* is increased in RBD-positive probable prodromal PD subjects^22^. *Akkermansia* is hypothesized to aggravate gut inflammation, increase gut permeability and is associated with levels of constipation^33^. In particular, *Akkermansia muciniphila* was increased in PD in previous studies and is known to thrive in fiber-depleted environments as it uses mucin as energy source, thereby degrading the intestinal wall and leaving the host more vulnerable to epithelial access of intestinal pathogens^34,35^. In the current study, an ASV belonging to *Akkermansia muciniphila* was increased in PD (Table 3), indicating that strains belonging to this species can already be increased at the time of diagnosis.

Fewer taxonomic differences were found in the current cohorts than reported in most previous PD microbiome studies^11^. This stresses the need for treatment-naive de novo cohorts to avoid the putative confounding effects of disease duration, deteriorating health and dopaminergic treatment. Adjusting for relevant confounders in the current study might have further filtered out non-PD specific taxa. In particular, constipation is a well-known non-motor symptom of PD and important determinant of gut microbiome composition^36,37^. Previous studies used questionnaires or single questions to assess constipation, but these methods are notorious for their low ability to detect constipation in PD^37,38^. Here, stool diaries measuring stool frequency and stool consistency were used, possibly providing a more objective and adequate adjustment for constipation^39,40^.

Both cohorts showed several differentially abundant taxa belonging to the family of Lachnospiraceae, and the direction of the difference for differentially abundant taxa was often the same. In addition, both cohorts are characterized by decreased relative abundances of SCFA-producing taxa in PD relative to HC. Nonetheless, it is striking that no taxon could be replicated in both cohorts with statistical significance. Though previous studies also reported various inconsistent results, certain study characteristics might explain inconsistencies between the two cohorts. First, the NL control cohort consisted of a large number of spouses from participants. Microbiome composition was less distant between spousal cases and controls. In addition, non-spousal controls were often spouses of PD patients from the Groningen PD expertise center who did not participate in the study. Possibly, a shared living environment with PD subjects might have ameliorated differences in gut microbiome composition in the entire NL cohort. Second, the different DNA extraction methods affected the differentially abundant taxa, in particular of taxa belonging to the family of Lachnospiraceae. Third, the recruitment differed between the two cohorts. Patients were directly referred by a neurologist for the purpose of participating in a PD study in case of the NL cohort. In the FIN cohort patients were selected after referral for a DAT-SPECT scan due to diagnostic uncertainty, possibly leading to an overrepresentation of untypical phenotypes. Fourth, the genetic background of the two cohorts might differ, with the Finnish population being particularly isolated in Europe, possibly driving different host-microbiome interactions^18,41,42^. Last, though no dietary variable was selected as a significant confounder for each cohort separately, no direct comparison of diet was possible between the two cohorts. Therefore, the NL and FIN cohorts might represent different dietary habits.

The current study has some clear advantages over previous microbiome studies in PD, such as the inclusion of treatment-naive de novo PD subjects, the use of two separate cohorts and a more adequate assessment of diet and constipation. Nonetheless, a few limitations need to be addressed. First, two different DNA extraction methods were used in both cohorts. As discussed, differences in gut microbiome composition driven by the DNA extraction method were assessed by parallel sampling of 47 stool samples using both methods. However, DNA extraction should ideally be performed using the same method for a direct comparison between cohorts.

Second, samples were randomized as much as possible during the DNA extraction and library preparation and were all sequenced together to minimize batch effects. Since already extracted HC samples from a previous publication were used to supplement the HC group of the FIN cohort, these samples could not be randomized during DNA extraction. In the NL cohort the DNA extraction batch was marked as a relevant confounder, but did not have a significant association in the final multivariable model (Table 2). In addition, batch effects from DNA extraction are less likely to impact the results of high-biomass samples such as feces^43^. However, some confounding in the FIN cohort cannot be excluded.

Third, despite presenting the two largest gut microbiome cohorts in de novo PD thus far, failed replication might still result from a lack of power. Provided a confounding effect of dopaminergic medication and the association of longer disease duration with more pronounced changes in gut microbiome composition, smaller effect sizes are expected in a treatment-naive de novo PD cohort. Arguably, at least at the taxonomic levels of genus and higher, the gut microbiome seems unsuitable as a diagnostic biomarker, although this should ideally be compared to relevant differential diagnoses or re-evaluated in relation to potential PD subtypes. Nonetheless, a lack of power might still obscure pathophysiological relevant associations. Wallen et al. were the first to describe overabundance of the putative opportunistic pathogens *Porphyromonas, Prevotella* and *Corynebacterium* at the genus level and attributed these novel findings to the larger sample size compared to previous studies^14^. Overabundance of these previously reported opportunistic taxa was not found in the current cohorts, but might be missed due to insufficient power.

Last, participants were excluded if they had used antibiotics in the month before stool sample collection. Even though antibiotics induced gut microbiota changes most often subside within 28 days, changes might persist for as long as several years, depending on the antibiotic used and inter-personal variability^44^.

Expansion of the current dataset can further confirm the association between PD medication and gut microbiota. First, the current study cannot be confounded by the effect of PD medication, but no causal inferences can be made based on the absence of previously described gut microbiome changes associated with PD medication use. Levodopa is suggested to drive positive selection of tyrosine decarboxylase producing bacteria, whereas overall dopaminergic input might influence gut microbiome composition via cerebral signaling or modulating stool transit times^45,46^. Currently, only one study investigated the influence of Levodopa initiation on gut microbiome composition in PD, but found no associated changes^47^. This study, however, only included 19 participants and included several participants who were stable users of other dopaminergic medication. Follow-up should ideally be done within two years, as gut microbiome composition does not seem to shift significantly as a result of disease progression in a two-year period^13^. This might confirm the association between dopaminergic medication and *Bifidobacterium, Lactobacillus*, and tyrosine decarboxylase producing bacteria.

Second, extended follow-up after two years would make it possible to analyze the association between disease progression and increased gut microbiome composition changes, particularly further decrease of SCFA-producing taxa and possible increases in opportunistic pathogens.

Additionally, adequate clinical subtyping could lead to the identification of subtype-specific microbiome changes. Given the large clinical heterogeneity of PD, different subtypes might represent different pathophysiological mechanisms and ports d’entrée. The recently proposed dichotomy of brain-first versus body-first PD might be of particular interest, with gut microbiome changes possibly being more pronounced in a probable body-first subtype^7^. The probable body-first subtype is currently defined by polysomnography proven RBD at the time of diagnosis and/or during the prodromal stages of PD. RBD was not included as a possible nuisance variable, as RBD is a PD-related variable and the primary aim of this study was to compare the gut microbiome of PD and HC, rather than the comparison of possible subtypes. Moreover, both cohorts used different questionnaire-based assessments, that identified different subjects as RBD-positive dependent on the metric used (data not shown). This is in line with the finding that questionnaire-based assessments of RBD have low sensitivity and specificity in de novo PD, and a lower predictive value in case of probable prodromal PD compared to polysomnography^23,48^. Adequate biomarkers of a probable body-first subtype, besides polysomnography, remain to be established and might be of interest for future research disentangling gut microbiome signatures within PD subgroups.

Third, as holds true for the majority of gut microbiome studies, the current study investigates the fecal microbiome. Nonetheless, the gut microbiome entails a large intrapersonal variety with different environments along the gastrointestinal tract, dependent on local conditions such as pH, fluid content, bile content, levels of oxygenation, and transit time^49^. Though often used synonymously, fecal microbiome composition can therefore not be considered as a snapshot of the much broader gut microbiome. In particular, the luminal microbiome of the colon might differ from the mucosal microbiome due to different^49^. Sampling at different sites along the gastrointestinal tract would provide valuable insights in the different environments that make up the PD gut microbiome, whereas mucosal biopsies would allow for the interrogation of direct microbiome-host interactions along the gut mucosal wall.

Last, though taxonomic research can already provide insight into the relationship between PD and gut microbiota, integration with data on the functionality of the intestine and the gut microbiome, could further elucidate relevant mechanisms. These include shotgun metagenomics and metabolomics studies, as well as markers of gut permeability, intestinal inflammation and systemic inflammation. Shotgun metagenomic sequencing would provide complete taxonomy data at the species level and would additionally provide data on the metabolic pathways associated with the sequenced gene fragments^48,49^. Metabolomic analysis would provide actual functional readouts of the gut microbiome and could confirm whether the current finding of reduced levels of SCFA-producing taxa is representative for the overall functional output of the gut microbiome in both PD cohorts. Current data on the metabolome of gut microbiota suggests reduced levels of SCFAs in PD, which would be in line with previous taxonomic studies and our current findings^29^. However, metabolomic data of treatment-naive de novo PD subjects is still lacking and could generate valuable hypotheses to be assessed in pre-clinical functional studies. Data on gut permeability, intestinal inflammation, and systemic inflammation can further indicate the means through which gut microbiota might increase vulnerability to PD pathology^5^.

In conclusion, our findings suggest fewer taxonomic differences in treatment-naive de novo PD subjects compared to previous studies with already treated patients in the more advanced stages of the disease. Although our finding of reduced levels of SCFA-producing taxa are in line with previous studies, none of our findings could be directly replicated with statistical significance in both cohorts, showing the importance of comparing multiple cohorts for analyzing highly variable data such as the microbiome. In addition, the importance of adequately assessing the effects of potential nuisance variables, such as constipation and batch effects of laboratory procedures, can be highlighted. Further enquiry of the metabolic profile of the gut microbiome in treatment-naive de novo PD and longitudinal analysis of the current dataset can provide further insight in relevant pathophysiological mechanisms and the extent to which gut microbiome changes might be confined to specific PD subtypes and medication effects.

## Methods

### Study population

For the Dutch (NL) cohort, treatment-naive de novo PD participants were included as part of the Dutch Parkinson Cohort of de novo PD subjects (DUPARC, ClinicalTrials.gov identifier NCT04180865)^50^. Inclusion criteria were PD diagnosis by a movement disorder specialist according to the Movement Disorders Society (MDS) clinical diagnostic criteria^1^, confirmed by a dopaminergic deficit quantified by FDOPA-PET or one-year follow-up if no FDOPA-PET was performed (*n* = 8). HC did not have a neurodegenerative disorder and could not be classified as probable prodromal PD^4^. HC were recruited from the same geographical area and were spouses of PD participants, caregivers of PD patients at the PD expertise center Groningen, or respondents to local advertisements.

For the Finnish (FIN) cohort, treatment-naive de novo PD participants were included as part of the Non-Motor Symptoms and DopAmine Transporter binding study (NMDAT study, ClinicalTrials.gov identifier NCT02650843). The patients were scanned with [I-123]FP-CIT SPECT because of parkinsonism or tremor for which they were referred to imaging by their neurologist. Inclusion criteria for this microbiome study were PD diagnosis by a movement disorder specialist according to the MDS clinical diagnostic criteria^1^, confirmed by a dopaminergic deficit quantified by [I-123]FP-CIT SPECT. [I-123]FP-CIT SPECT were analyzed with BRASS software (Hermes Medical Solutions AB, Stockholm, Sweden), in which a dopaminergic deficit was defined as more than two standard deviations below the reference mean in any of the six analyzed regions. The study subjects were required to be aged 18 or over and to be able to understand and answer the questionnaires in Finnish. Only the patients who also filled out the gastrointestinal questionnaires were included in the current analyses. HC samples of the FIN cohort were from participants of the previously described PD microbiome follow-up study by Aho et al.^13^ and HC of the GAMbling and DopAmine Transporter binding (GAMDAT) study^51^.

Shared exclusion criteria for both cohorts comprised major confounders of gut microbiome composition (eg. recent antibiotics usage in the previous month). A complete overview of the exclusion criteria per study is provided in Supplementary Table 1. All studies were approved by their respective local ethics committee and all participants provided written informed consent: the ethics committee of the University Medical Center Groningen provided ethical approval for the DUPARC study; the ethics committee of the Turku Hospital district provided ethical approval for the NMDAT and GAMDAT study; the ethics committee of the Hospital District of Helsinki and Uusimaa provided ethical approval for the Aho et al. follow-up study.

### Data and stool sample collection

Participants in the NL cohort collected their stool sample at home and stored it in their home freezer (−20 °C). Stool samples were then stored at −80 °C at the University Medical Center Groningen (UMCG) and were kept frozen on dry ice during all transportation. PD participants in the NL cohort were extensively characterized, as previously described^50^, with HC receiving a more selective assessment. For the current study, determinants of gut microbiome composition were selected as potential confounders in three different categories: (1) the medical history, including disease history, medication use (eg. use of proton pump inhibitors, opioids, antidepressants), and family history; (2) nutrient intake based on a dietary diary that was filled out for three consecutive days, including alcohol usage; (3) gastrointestinal functioning represented by stool consistency and frequency based on a stool diary that was filled out for seven consecutive days, with consistency represented by the Bristol Stool Chart, and the subjective sense of constipation asked with question 5 of the Non-Motor Symptom Questionnaire (NMSQ)^52^. In addition, motor-symptomatology was examined using the Movement Disorder Society Unified Parkinson Disease Rating Scale (MDS-UPDRS) part III^53^.

Participants in the FIN cohort collected their stool sample at home using a collection tube with DNA-stabilizing buffer (PSP Spin Stool DNA Plus Kit, STRATEC Molecular), which were send via postal services to the University of Helsinki laboratory where they were stored at −80 °C. If samples were not immediately shipped, they were stored at home in the refrigerator. Participants in the FIN cohort received similar assessments of potential confounders of gut microbiome composition and PD status in the categories of medical history, diet and gastrointestinal function. Notable differences compared to the NL cohort were the usage of a food frequency questionnaire, concerning food consumption in the month before stool sampling, instead of a dietary diary. Also, there were several additional measures of subjective constipation instead of question 5 of the NMSQ: a question about strained defecation in the stool diary and the ROMEIII, Wexner, and Constipation Severity Index (CSI) questionnaires. A complete overview of the assessed endpoints per cohort is provided in Supplementary Table 2.

### Laboratory procedures

All laboratory procedures were performed at the Institute of Biotechnology, University of Helsinki. DNA extraction of the NL samples was performed using the Qiagen Allprep DNA/RNA Mini Kit, whereas the PSP Spin Stool DNA Plus Kit (Invitek Molecular, Germany) was used for the FIN samples. To assess the possible influence of both sample collection and DNA extraction protocols, a subset of the NL participants (*n* = 47) collected an additional stool sample from the same feces as their regular stool sample collection. The additional sample was collected using the FIN cohort protocol with PSP tubes and processed using the PSP Spin Stool Kit. The V4 hypervariable region of the 16S rRNA gene was amplified using a mixture of universal primers 515F1-4 (5′-GTGCCAGCMGCCGCGGTAA-3′) and 806R1-4 (5′-GGACTACHVGGGTWTCTAAT-3′) with partial Illumina TruSeq adapter sequences added to the 5′ ends:

- F1 ACACTCTTTCCCTACACGACGCTCTTCCGATCT,
- F2 ACACTCTTTCCCTACACGACGCTCTTCCGATCTca,
- F3 ACACTCTTTCCCTACACGACGCTCTTCCGATCTgca,
- F4 ACACTCTTTCCCTACACGACGCTCTTCCGATCTagcaatt,
- R1 GTGACTGGAGTTCAGACGTGTGCTCTTCCGATCT,
- R2 GTGACTGGAGTTCAGACGTGTGCTCTTCCGATCTca,
- R3 GTGACTGGAGTTCAGACGTGTGCTCTTCCGATCTatct,
- R4 GTGACTGGAGTTCAGACGTGTGCTCTTCCGATCTtctact

The additional nucleotides (non-capitalized letters) are introduced for mixing in sequencing. The 2-step PCR amplification was done as described in Aho et al. 2019^13^. Every DNA extraction and amplification batch included a blank control to assess possible contamination and case and control samples were semi-randomized, where possible, to avoid batch effects. Barcodes were selected using BARCOSEL^54^. All samples were sequenced together for four runs using Illumina MiSeq (v3 600 cycle kit), with 325 bases for the forward and 285 bases for the reverse read.

### Bioinformatics

Forward and reverse primers were removed from R1 and R2 reads using cutadapt (v2.10)^55^. Further bioinformatics were performed with DADA2 (v1.18)^56^. Forward sequences were trimmed at 200 nucleotides (nt) and reverse sequences at 150nt, quality trimmed at the first instance of a nucleotide (or group of nucleotides) with quality score 2. Reads with more than 10 expected errors or ambiguous nucleotides were discarded. Amplicon sequence variants (ASVs) were inferred for each of the four runs separately, using default parameters in DADA2. An overview of the number of sequences per sample during each of the preprocessing steps (primer removal, quality filtering and length trimming, denoising forward and reverse reads, merging forward and reverse reads) is provided in Supplementary Table 10. In addition, the number of sequences of the blank control samples at the DNA extraction and PCR steps are included, showing very minimal to no contamination during the wet lab work (Supplementary Table 10). Subsequently, the four resulting sequence tables were merged, ASVs that only differed in length were collapsed and chimera were removed. Taxonomic assignments were based on the SILVA (v138) reference database^57^, in which taxonomic assignments are first performed up to genus level using a naive Bayesian classifier with 50% bootstrap confidence. Second, species are assigned based on a 100% match with the references sequence if the match corresponds to the already assigned genus.

### Statistical analysis

Due to the geographical and cultural differences, as well as the different endpoints that were assessed, the NL and FIN cohort were analyzed separately. Supporting the large influence of geographical differences, samples were more distant in terms of their overall microbiome composition when contrasting the geographical origin, compared to PD and HC status (Supplementary Fig. 2). All statistical analyses were performed with the statistical software R, v4.0.3. The threshold for statistical significance was set at *p* < 0.05 (two-sided), or adjusted *p* < 0.05 if corrected for multiple comparisons. Comparison of clinical and technical variables were performed using a Chi-square test for categorical variables and a Student’s t-test or a Mann-Whitney U test for continuous variables, depending on the distribution of the variable. For microbiota analyses, the sequence tables, taxonomy tables and metadata tables were merged in a phyloseq object (phyloseq, v1.34.0)^58^ after which data transformations and subsequent analyses were performed using the R packages microbiome (v1.12.0) and vegan (v2.5–7). Multivariate analyses of overall community structure were performed on Aitchinson distances: Euclidean distances calculated after centered log-ratio (clr) transformation of the ASV count data^59^. Statistical significance was calculated using a PERMANOVA with adjustment for selected variables (see variable selection)^60^. PERMANOVA was performed with marginal testing, meaning all variables were assessed whilst holding constant all other variables in the model. In parallel, a non-compositional approach was performed using Bray-Curtis distances after rarefaction^59^. Cut-offs for rarefaction were identified for both datasets separately, based on the plateauing of the rarefaction curves depicting sample richness (Supplementary Fig. 1). Similarly, the differential abundance analyses were performed with adjustment for selected confounders using both a compositional approach with ANCOM (v2.1, using default settings and code from: github.com/FrederickHuangLin/ANCOM-Code-Archive) and a non-compositional approach with DESeq2 (v1.30.1)^61–63^. Continuous variables were scaled and centered for the DESeq2 analysis, to improve model convergence. Differential abundance analyses were performed at the ASV, genus and family level after filtering out taxa which occurred in fewer than ten percent of the samples. Taxa were identified as differentially abundant with a detection threshold of 0.7 in ANCOM or with a false discovery rate (FDR) adjusted p-value < 0.05 in DESeq. Alpha diversity was investigated using alpha diversity indices Chao1, Shannon, Inverse Simpson, and observed richness, which were tested univariably using a Mann-Whitney U test. Results were plotted using ggplot2 (v3.3.3), with relative abundances calculated after adding a pseudo-count of 1 to sequence count data, in order to present data on a logarithmic scale.

### Variable selection

Given the primary aim to describe gut microbiome changes for the first time in treatment-naive de novo PD subjects, all clinical data described under “data and stool sample collection” and technical metadata (eg. DNA extraction batch, PCR batch, number of reads) were investigated for their potential to influence the effect of group status on gut microbiome composition. The variables age, sex, BMI, stool consistency and DNA extraction batch were selected a priori as potentially relevant nuisance variables. To ensure a large effect of another variable on the relation between PD status and gut microbiome composition would not be missed, all variables were screened for their potential effect using a PERMANOVA in a model with PD status: distance-matrix ~ PD status + variable

Variables that shifted the explained variance of PD status (R2) by at least ten percent compared to the univariable explained variance of PD status (distance-matrix ~ PD status), were added to the list of relevant model covariates (Supplementary Table 3). Subsequently, the selection of relevant covariates was investigated for collinearity with PD status. Variables with a generalized variance inflation factor (GVIF) ≥ 2 with PD status were excluded. In the overall model, a GVIF of 3 was used as threshold for collinearity, excluding variables with a higher GVIF or one of several variables that only showed collinearity amongst each other (Supplementary Table 4). This resulted in the following models for the multivariate analysis of overall gut microbiome composition.

For the NL cohort, model (1): distance-matrix ~ PD status + age + sex + BMI + stool consistency + NMSQ constipation + stool frequency + DNA extraction batch

For the FIN cohort, model (2): distance-matrix ~ PD status + age + sex + BMI + stool consistency + CSI total score + strained defecation (stool control) + number of sequences

For the differential abundance analysis, covariates were selected that showed a significant relationship with the overall gut microbiome composition in the final model with p < 0.1.

For the NL cohort, model (3): taxon ~ PD status + age + BMI + stool consistency + stool frequency

For the FIN cohort, model (4): taxon ~ PD status + stool consistency + number of sequences

## Supplementary information

Supplementary Information

## Data Availability

Sequencing data of the V4 region of the 16S rRNA gene are available via the European Nucleotide Archive (ENA) under accession number PRJEB55464. Sample metadata can be requested from the respective principal investigators upon request.

## Code availability

No custom codes were used. All relevant software, packages, and their versions are stated in the “Methods” section.
