# Supplementary material for: Fecal microbiome alterations in treatment-naive de novo Parkinson’s disease
Source: NPJ Parkinsons Dis. 2022 Oct 10;8:129. doi: 10.1038/s41531-022-00395-8 (PMC9551094; doi:10.1038/s41531-022-00395-8)
Supplement: Supplementary file 1 — Supplementary Information [file 41531_2022_395_MOESM1_ESM.pdf]

Supplementary Material

Contents

- Supplementary table 1: Exclusion criteria (p.2)
- Supplementary table 2: Overview of assessed endpoints (p.7)
- Supplementary figure 1: Rarefaction curves (p.8)
- Supplementary figure 2: Overall microbiome composition of both cohorts according to (A) group (PD and HC) status and (B) country of origin (p.9)
- Supplementary tables 3: Variable selection (p.10)
- Supplementary tables 4: Collinearity screening (p.23)
- Supplementary tables 5: Differential abundances at ASV level (p.26)
- Supplementary tables 6: Differential abundances at Genus level (p.68)
- Supplementary tables 7: Differential abundances at Family level (p.84)
- Supplementary tables 8: Comparison DNA extraction methods (p.92)
- Supplementary tables 9: Same household stratification (p.97)
- Supplementary tables 10: Lost reads per sample (p.98)

Supplementary table 1: exclusion criteria

Exclusion criteria NL cohort

All participants:<br>• Active or persistent primary disease of the gastrointestinal tract<br>• History of peritonitis, severe endometriosis, abdominal, intestinal or urogenital fistula,<br>• Hepatobiliar or pancreatic disease (except asymptomatic cholecystolithiasis)<br>• History of abdominal or anorectal surgery, except minor surgery such as uncomplicated appendectomy or cholecystectomy (>6 months ago).<br>• Severe gynaecological prolapse (grade III)<br>• Cancer and/or adjuvant treatment within the last 6 months<br>• Within the last three months: severe hypo- or hyperkalemia, narcosis, analgosedation, endoscopic procedure of the gastrointestinal tract, abdominal trauma<br>• Within the last three months: gastrointestinal tract infection, food intoxication<br>• Antibiotic use in the last month
PD participants:<br>• History of dopaminergic medication use*
HC participants:<br>• History of neurodegenerative disease, in particular signs of parkinsonism.<br>• Probable prodromal PD[5]

*One PD participant in the NL cohort had used levodopa/carbidopa (62.5mg 3 times daily) for a maximum period of one month, but had stopped at least 4 months before stool sample collection. Due to the short duration of treatment, the low dose, and a wash-out period of at least four months, the patient was not excluded. In addition, the gut microbiome composition of the participant proofed not to be an outlier when visualized in the PCA plot of overall gut microbiome composition.

Exclusion criteria FIN cohort

All participants NMDAT:

- Any kind of limitation affecting their ability to understand the informed consent, such as significant mental health problems or cognitive problems (Mini-Mental State Examination (MMSE)<18

PD participants NMDAT:

- History of dopaminergic medication use
- Antibiotic use in the last month
- Imaging with a scanner whose binding ratios cannot be compared to other scanners´ values

HC participants GAMDAT:

- Evidence of clinically significant cardiovascular, renal, hepatic, hematological, gastrointestinal, pulmonary, endocrinological or neurological disease
- Evidence of current alcohol or substance use disorder within last 6 months (DSM-V)
- Intoxicated or recent (less than 36 h) drug or alcohol usage
- Body weight > 180 kg (scanner limit)
- Strong susceptibility to allergic reactions or nausea
- Blood donation within 60 days prior to the study
- Has undergone a prior PET or SPECT study
- Current treatment with amphetamine derivatives, methylphenidate, bupropion or other medications known to interfere with DAT imaging if the medication cannot be stopped for a minimum of 1 month before DAT imaging
- Any contraindication to magnetic resonance imaging (MRI)

- Current pregnancy (i.e. has to be surgically sterilized, at least one year postmenopausal, or negative serum pregnancy test before the study) or lactation in women
- Current psychiatric DSM-V Axis-I disorder (e.g. major depression, bipolar disorder psychoses)

HC participants Aho et al (assessed at baseline inclusion for Scheperjans 2015[6]):

- Active smoking in last 6 months
- Diagnosis of dementia or MMSE < 25 points
- Diagnosis of major depression or GDS-15 > 9 points
- Diagnosis of psychosis
- Any signs of parkinsonism
- Hyposmia
- REM sleep behaviour disorder
- Restless legs syndrome
- NMSQuest score >3 (not including items 4, 6, 7, 10, 18, 23, 27)
- First degree relative or more than one relative with PD
- HIV infection
- Living in same household with PD patient
- Active or persistent primary disease of gastrointestinal tract: e.g. celiac disease, pernicious anemia, autoimmune gastritis, symptomatic diverticulosis, inflammatory bowel disease, irritable bowel syndrome, strictures, adhesions, varicosis or diverticulum of the esophagus, Meckel's diverticulum (Exception: other forms of chronic gastritis)
- Endocrinological disease (Exception: diabetes mellitus without polyneuropathy; treated hypothyreosis with normal thyrotropin level)

- Alcohol abuse
- B-hypovitaminosis
- History of hepatobiliar or pancreatic disease (Exception: asymptomatic cholecystolithiasis)
- Previous abdominal or anorectal surgery (Exceptions: can be enrolled
- after uncomplicated haemorrhoid procedure and 1 year after uncomplicated appendectomy, inguinal hernia repair, cholecystectomia, or gynecologial surgery if symptoms resolved and no signs of adhesions)
- Severe gynaecological prolapse (grade III) (Exception: can be enrolled after repair procedure if symptoms resolved and no signs of adhesions)
- History of peritonitis, severe endometriosis, polyneuropathy, polio, spina bifida, severe symptomatic spinal stenosis (if not symptom free for at least 1 year), paraparesis of any cause, symptomatic peripheral arteriosclerosis, any signs or history of intestinal ischemia (e.g. claudication), aortic aneurysm or dissection, connective tissue disease, autoimmune disease, sarcoidosis outside of the lungs or skin, cancer that is not considered cured, abdominal, intestinal, or urogenital fistula (Exceptions: treated autoimmunehypothyreosis with normal thyrotropin level)
- Heart failure (Exception: isolated left ventricular heart failure NYHA ≤ II)
- Known severe renal insufficiency (glomerular filtration rate < 30 ml/min)
- One of the following within the previous 2 months: severe hypokalemia or hyperkalemia demanding hospital treatment, narcosis or analgosedation, endoscopic procedure of the gastrointestinal tract, abdominal trauma
- Any of the following within the last 2 months: gastrointestinal or respiratory tract infection, food intoxication, major epistaxis requiring treatment by a physician

- Antibiotic treatment within the last month
- Any drug abuse
- Any regular use (>2 times a week) of the following medications over the last 2 months: opioids, loperamide, inhaled β-agonists or anticholinergics, glucocorticoids (oral or parenteral), tricyclic antidepressants, antihistaminics with systemic anticholinergic effects, metoclopramide, cholinergics, anticholinergics (except for PD indication), domperidone, protone pump inhibitors

Supplementary table 2: overview of assessed endpoints

Variable	NL cohort PD (DUPARC)	NL cohort HC (DUPARC)	FIN cohort PD (NMDAT)	FIN cohort HC (GAMDAT)	FIN cohort HC (Aho et al.)
**Technical variables**					
DNA extraction method	X	X	X	X	X
DNA extraction batch	X	X	X	X	X
PCR batch	X	X	X	X	X
Number of reads	X	X	X	X	X
**Medical history**					
Age	X	X	X	X	X
Sex	X	X	X	X	X
BMI	X	X	X	X	X
Disease history	X	X	X	X	X
Medication use	X	X	X	X	X
Family anamnesis	X	X	X	X	X
Allergies	X	X	X	X	X
Lactose intolerance	X	X	X	X	X
Alcohol use	X	X	X	X	X
Smoking	X	X	X	X	X
Drug use	X	X	X	X	X
**Gastrointestinal health**					
Stool diary	X	X	X	X	X
Bristol Stool Chart	X	X	X*	X*	X*
RomeIII			X	X	X
Constipation Severity Index			X	X	X
Wexner			X	X	X
NMSQ question 5: constipation	X	X			
**Dietary habits**					
Dietary diary	X	X			
Food frequency questionnaire			X	X	X
**PD clinical characteristics**					
MDS-UPDRS part III	X	X	X		
Hoehn and Yahr	X	X	X		

MDS-UPDRS, Movement Disorder Society Unified Parkinson Disease Rating Scale; NMSQ, non-motor symptom questionnaire.

*A modified version of the Bristol Stool Chart was used in the FIN cohort

Supplementary figure 1: rarefaction curves

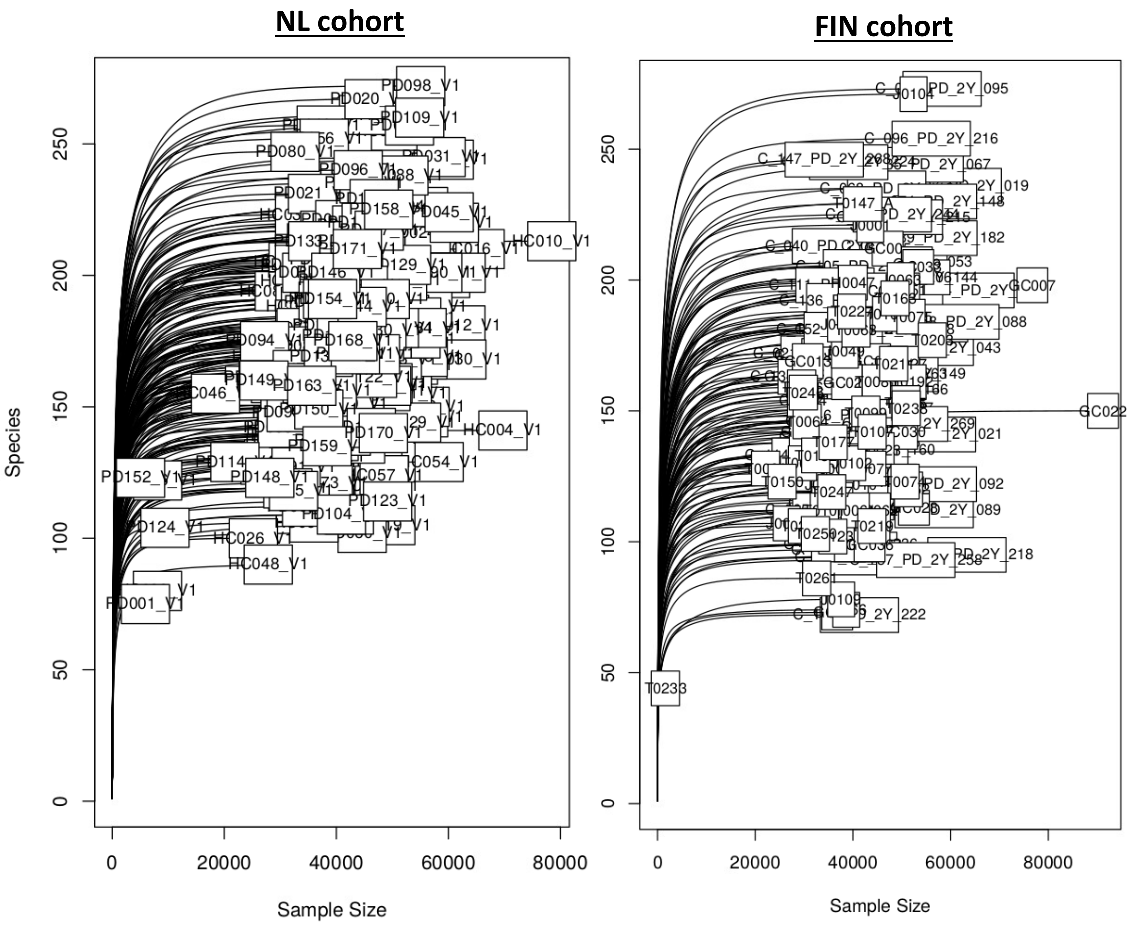

<u>Supplementary figure 2</u>

A. Group **B. Country**

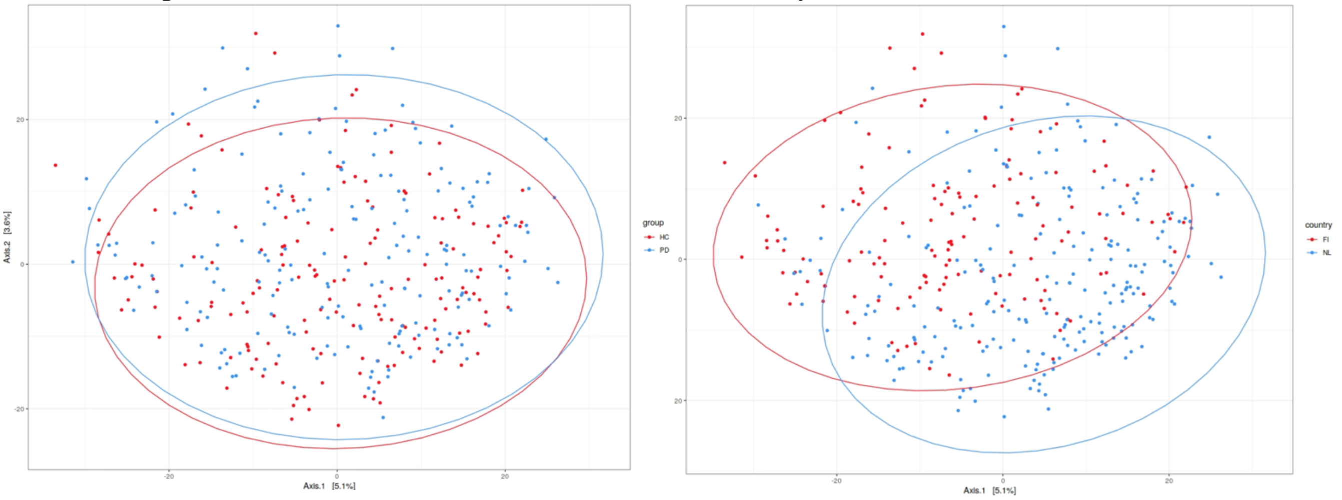

Overall microbiome composition of both cohorts according to (A) group (PD and HC) status and (B) country of origin. A larger distance between the overall microbiome composition of the two different countries can be observed compared to group status. This was also confirmed by means of PERMANOVA using 1E6 permutations: group status R2 = 0.00534, p = 9E-06; country R2 = 0.02218, p = 1E-06 (lowest possible p-value with 1E6 permutations).

Supplementary table 3A: variable selection NL cohort

Screening for the influence of assessed variables on the relationship between PD status and overall microbiome composition.
Variables were incorporated in a model with PD status (overall microbiome composition ~ PD status + variable) to assess the extend to which the variable changes the explained variance (R2) of PD status on overall microbiome composition.
Variables with >10 percent shift in the R2 (percShift_R2) of PD status, compared to the univariable R2 of PD status (calculated for each comparison separately dependent on the removal of samples due to missing data of the variable investigated) were added to the list of nuisance variables.

**variable**	**PDstatus_univar_R2**	**PDstatus_univar_p**	**PDstatus_model_R2**	**PDstatus_model_p**	**variable_model_R2**	**variable_model_p**	**percShift_R2**
nmsq_constipation	0,006250416	0,00579942	0,004998816	0,155584442	0,006759422	0,00109989	-20,02426782
stoolconsistency_avg	0,00610101	0,00909909	0,004938913	0,192080792	0,008526829	1,00E-04	-19,04762185
stoolfrequency_avg	0,00610101	0,00909909	0,005010382	0,158684132	0,007507731	0,00029997	-17,87618121
stoolsample_consistency	0,006244639	0,00689931	0,005671915	0,03039696	0,006633301	0,00229977	-9,171449391
DNA_extraction_batch	0,006187374	0,00639936	0,006567274	0,00119988	0,064550231	0,264073593	6,139922693
med_anacidic_oldhels	0,006187374	0,00639936	0,005914009	0,01089891	0,008115701	1,00E-04	-4,418116966
med_constipation	0,006187374	0,00639936	0,005928591	0,00979902	0,004918726	0,182581742	-4,182442225
fam_ibd	0,006187374	0,00639936	0,0064352	0,00349965	0,004873057	0,249975002	4,005347921
med_ssri	0,006187374	0,00639936	0,006427533	0,00329967	0,006114376	0,01189881	3,881434766
med_ppi	0,006187374	0,00639936	0,005956429	0,009999	0,00785643	1,00E-04	-3,732513931
hist_hypothyroid	0,006187374	0,00639936	0,005957175	0,0109989	0,004522376	0,453154685	-3,72047231
DNA_extraction_concentration	0,006187374	0,00639936	0,005975232	0,0089991	0,01011654	1,00E-04	-3,428627577
hist_nongi_cancertreatment	0,006187374	0,00639936	0,005977808	0,00959904	0,00450225	0,458254175	-3,387003136
mono_disaccharides_total_g	0,00610095	0,01209879	0,005895415	0,02169783	0,007268414	0,00089991	-3,368893099
zeaxanthin_ug	0,00610095	0,01209879	0,006284186	0,00779922	0,004134495	0,696630337	3,003396771
med_thyroidsupplement	0,006187374	0,00639936	0,00600466	0,00959904	0,004673989	0,333566643	-2,953021333
med_nsaids	0,006187374	0,00639936	0,006010591	0,00929907	0,005289828	0,085291471	-2,857152087
fattyacids_unsaturated_trans_total_g	0,00610095	0,01209879	0,005937777	0,01939806	0,005851968	0,066493351	-2,67454573
nrofreads_seqtab_nolengthfilt	0,006187374	0,00639936	0,006337526	0,00449955	0,006774987	0,00169983	2,426744008
med_calciumantagonist	0,006187374	0,00639936	0,00604783	0,00869913	0,00494172	0,172882712	-2,255297022
hist_dental	0,006187374	0,00639936	0,006047937	0,00829917	0,004419252	0,466853315	-2,253581209
hist_nongi_cancer	0,006187374	0,00639936	0,006053522	0,00839916	0,004387314	0,574842516	-2,163312245
med_vitd	0,006187374	0,00639936	0,006053549	0,00819918	0,006375584	0,00379962	-2,162869556
med_plateletaggregationinhibitor	0,006187374	0,00639936	0,006057965	0,00779922	0,004935526	0,173282672	-2,09149538
sex	0,006187374	0,00639936	0,006059445	0,00809919	0,005153689	0,099590041	-2,067579486
fattyacids_polyunsaturated_n6cis_g	0,00610095	0,01209879	0,006220538	0,00979902	0,005271042	0,122687731	1,960150572
hist_otherautoimmune	0,006187374	0,00639936	0,006304036	0,00409959	0,005009744	0,167583242	1,885476098
PCR_batch	0,006187374	0,00639936	0,006075187	0,00839916	0,065287289	0,177182282	-1,813155205

variable	PDstatus_univar_R2	PDstatus_univar_p	PDstatus_model_R2	PDstatus_model_p	variable_model_R2	variable_model_p	percShift_R2
vitK2_ug	0,00610095	0,01209879	0,00621015	0,00909909	0,006248488	0,01019898	1,789882927
fam_pd_2nddegree	0,006187374	0,00639936	0,006295669	0,00469953	0,004786831	0,249575042	1,7502515
nitrogen_total_g	0,00610095	0,01209879	0,006206205	0,0089991	0,004529006	0,589841016	1,725230885
med_othergutmeds	0,006187374	0,00639936	0,006083981	0,00729927	0,006808823	0,03169683	-1,671039321
hist_esophagusgastric	0,006187374	0,00639936	0,006091007	0,00779922	0,005201951	0,105089491	-1,557484773
med_antihistaminic	0,006187374	0,00639936	0,006282869	0,00479952	0,004694215	0,316268373	1,543380764
weight	0,006464502	0,00269973	0,006562962	0,0019998	0,006493958	0,00219978	1,523082677
selenium_ug	0,00610095	0,01209879	0,006192304	0,00989901	0,005059972	0,193880612	1,497370116
med_glp1antagonists	0,006187374	0,00639936	0,006096789	0,00719928	0,004930698	0,273272673	-1,464025587
hist_ent_surgery_other	0,006187374	0,00639936	0,006277718	0,00529947	0,004310412	0,648435156	1,460138858
vitE_total_mg	0,00610095	0,01209879	0,006188849	0,00979902	0,005997506	0,01809819	1,440740359
med_otherlungmeds	0,006187374	0,00639936	0,006100576	0,00719928	0,005727052	0,071192881	-1,402832688
hist_appendectomy	0,006187374	0,00639936	0,00627326	0,00489951	0,005196615	0,094390561	1,388077642
med_calcium	0,006187374	0,00639936	0,006102958	0,00689931	0,005433542	0,082391761	-1,364319945
med_tricyclicantidepressant	0,006187374	0,00639936	0,006270629	0,00509949	0,004741888	0,311868813	1,345566881
med_insulin	0,006187374	0,00639936	0,006270529	0,00539946	0,005299468	0,081191881	1,343951927
med_anticoagulant	0,006187374	0,00639936	0,006106654	0,00719928	0,00549758	0,04979502	-1,304597157
folicacid_ug	0,00610095	0,01209879	0,006179448	0,01039896	0,003858886	0,851314869	1,286655259
med_corticosteroids_other	0,006187374	0,00639936	0,006266304	0,00489951	0,004399829	0,570142986	1,275653116
retinolequivalent_ug	0,00610095	0,01209879	0,006023968	0,01519848	0,005184776	0,144785521	-1,261798435
hist_polyneuropathy	0,006187374	0,00639936	0,006109828	0,00689931	0,005238636	0,118488151	-1,253302164
hist_cardiovascular	0,006187374	0,00639936	0,006111383	0,00689931	0,005627791	0,03329667	-1,228156148
water_g	0,00610095	0,01209879	0,006027151	0,01429857	0,004894837	0,292070793	-1,209623301
hist_nosemouth	0,006187374	0,00639936	0,006112672	0,00749925	0,004956149	0,211878812	-1,207329619
polysaccharides_total_g	0,00610095	0,01209879	0,006027569	0,01389861	0,006079442	0,01359864	-1,202783579
med_phosphodiesteraseinhibitors	0,006187374	0,00639936	0,006258815	0,00559944	0,003816565	0,800119988	1,154621191
hist_stroke	0,006187374	0,00639936	0,00611634	0,0069993	0,006047386	0,01359864	-1,148042932
fam_pd_1stdegree	0,006187374	0,00639936	0,006118363	0,00719928	0,005115236	0,110788921	-1,115358307
alpha_carotene_ug	0,00610095	0,01209879	0,006032922	0,01629837	0,004414486	0,687431257	-1,115028572
hist_ent_oldhels	0,006187374	0,00639936	0,006255615	0,00549945	0,004243237	0,710628937	1,102899414
polyols_total_g	0,00610095	0,01209879	0,006034143	0,01429857	0,005558681	0,061493851	-1,095021293
betacarotene_ug	0,00610095	0,01209879	0,006034687	0,01489851	0,006293921	0,01089891	-1,086109676
med_diuretic	0,006187374	0,00639936	0,006124848	0,0069993	0,005055853	0,130086991	-1,010548297
retinol_activityequivalent_ug	0,00610095	0,01209879	0,006041621	0,01459854	0,004675138	0,450554945	-0,972451404
nicotinicacid_mg	0,00610095	0,01209879	0,006043495	0,01429857	0,005525297	0,057294271	-0,941731545
BMI	0,006464502	0,00269973	0,00652475	0,00229977	0,008080192	1,00E-04	0,931985997

variable	PDstatus_univar_R2	PDstatus_univar_p	PDstatus_model_R2	PDstatus_model_p	variable_model_R2	variable_model_p	percShift_R2
hist_gi_surgeryother	0,006187374	0,00639936	0,006129867	0,00749925	0,004600917	0,387361264	-0,929422035
beta_tocopherol_mg	0,00610095	0,01209879	0,006045475	0,0149985	0,004814668	0,362863714	-0,909282651
hist_nongi_surgery	0,006187374	0,00639936	0,00613125	0,00669933	0,005171992	0,091390861	-0,907074109
fattyacids_polyunsaturated_total_g	0,00610095	0,01209879	0,006154424	0,01079892	0,005313716	0,108989101	0,87649084
smoking_status	0,006187374	0,00639936	0,006241519	0,0049995	0,011120028	0,00839916	0,875089782
med_cholesterol_nonstatin	0,006187374	0,00639936	0,006133733	0,00689931	0,004392191	0,548145185	-0,866943598
smoking_PY	0,006187168	0,0229977	0,006134985	0,02529747	0,006074777	0,04109589	-0,843412779
phosphor_mg	0,00610095	0,01209879	0,00614912	0,01089891	0,006521185	0,00359964	0,789555789
iodine_ug	0,00610095	0,01209879	0,006053276	0,0139986	0,006541309	0,00309969	-0,781414501
vitB6_mg	0,00610095	0,01209879	0,006054847	0,01329867	0,005599919	0,04969503	-0,755664554
med_detrusorinhibitor	0,006187374	0,00639936	0,006141327	0,00679932	0,004980107	0,149985001	-0,744218363
age	0,006187374	0,00639936	0,006142572	0,00639936	0,008428912	1,00E-04	-0,724094891
med_h2antagonists	0,006187374	0,00639936	0,006231434	0,00569943	0,006016763	0,04309569	0,712094064
med_ang2antagonist	0,006187374	0,00639936	0,006144293	0,00669933	0,004586129	0,390860914	-0,696270032
med_kortisone_oldhels	0,006187374	0,00639936	0,00623002	0,00549945	0,004274705	0,688931107	0,689236106
height	0,006464502	0,00269973	0,006420111	0,00329967	0,004759613	0,307469253	-0,68668297
fam_cardiovascular	0,006187374	0,00639936	0,006226018	0,00559944	0,004858921	0,208179182	0,624558309
med_systemic_hormonal_treatment	0,006187374	0,00639936	0,006224974	0,00539946	0,003614091	0,936006399	0,607690265
vitD3_cholecalciferol_ug	0,00610095	0,01209879	0,006065087	0,01409859	0,004500255	0,492250775	-0,587816291
fattyacids_saturated_total_g	0,00610095	0,01209879	0,00606541	0,01279872	0,007255246	0,00039996	-0,582533782
organicacids_total_g	0,00610095	0,01209879	0,006136246	0,01089891	0,007056703	0,00139986	0,578528257
proteintotal_g	0,00610095	0,01209879	0,006065948	0,01279872	0,006395607	0,00589941	-0,573707063
delta_tocopherol_mg	0,00610095	0,01209879	0,006067279	0,01359864	0,004458766	0,555744426	-0,551900434
fattyacids_monounsaturated_cis_total_g	0,00610095	0,01209879	0,006133448	0,01179882	0,005171864	0,150084992	0,532668115
magnesium_mg	0,00610095	0,01209879	0,00613298	0,01109889	0,007071851	0,00089991	0,525000377
vitK_total_ug	0,00610095	0,01209879	0,00606965	0,0129987	0,004286662	0,774422558	-0,513027143
hist_depression	0,006187374	0,00639936	0,006156121	0,00759924	0,004427145	0,503349665	-0,505118035
fam_lung	0,006187374	0,00639936	0,006156378	0,00649935	0,00537014	0,063293671	-0,500964793
med_aceinhibitor	0,006187374	0,00639936	0,006217549	0,00609939	0,004068738	0,858914109	0,48768233
alpha_tocopherol_mg	0,00610095	0,01209879	0,006130272	0,01149885	0,005789766	0,02929707	0,480613745
med_lungmeds_oldhels	0,006187374	0,00639936	0,006158134	0,00639936	0,006323795	0,00689931	-0,472582166
alcoholdiet_g	0,00610095	0,01209879	0,006072206	0,01279872	0,005147477	0,160483952	-0,471135956
med_nitrates	0,006187374	0,00639936	0,006158478	0,00639936	0,003495179	0,902109789	-0,467023914
med_opioids	0,006187374	0,00639936	0,006158856	0,00649935	0,005272343	0,110388961	-0,460907027
vitD_25hydroxy_ug	0,00610095	0,01209879	0,006128887	0,0109989	0,005634714	0,054794521	0,457908568
hist_lungdiseaseother	0,006187374	0,00639936	0,006215588	0,00609939	0,004500246	0,451654835	0,455993397

variable	PDstatus_univar_R2	PDstatus_univar_p	PDstatus_model_R2	PDstatus_model_p	variable_model_R2	variable_model_p	percShift_R2
med_folicacid	0,006187374	0,00639936	0,006159868	0,00639936	0,006131063	0,02239776	-0,444549322
med_multivit	0,006187374	0,00639936	0,006213306	0,00609939	0,003770911	0,917308269	0,419108557
zinc_mg	0,00610095	0,01209879	0,006125961	0,01109889	0,005909059	0,02269773	0,409947888
med_betablocker	0,006187374	0,00639936	0,006211904	0,00589941	0,005627755	0,02529747	0,396445858
hist_bowel	0,006187374	0,00639936	0,006211603	0,00609939	0,004526319	0,440655934	0,391591058
med_ace_ang_oldhels	0,006187374	0,00639936	0,006209674	0,00569943	0,004691872	0,304469553	0,360409743
fattotal_g	0,00610095	0,01209879	0,006122858	0,01159884	0,006106974	0,01219878	0,359093008
vitK1_ug	0,00610095	0,01209879	0,00607918	0,01289871	0,004091195	0,897510249	-0,356830271
ash_g	0,00610095	0,01209879	0,006122494	0,01179882	0,007884264	1,00E-04	0,353133896
allergies	0,006187374	0,00639936	0,006165597	0,00639936	0,00525139	0,074892511	-0,351961564
retinol_ug	0,00610095	0,01209879	0,006080218	0,0129987	0,004225272	0,826617338	-0,339812039
med_otherantidiabetic	0,006187374	0,00639936	0,006208233	0,00609939	0,00539578	0,066393361	0,337121321
lycopene_ug	0,00610095	0,01209879	0,006121494	0,01159884	0,005619904	0,063893611	0,336736289
fattyacids_polyunsaturated_n3cis_g	0,00610095	0,01209879	0,006121303	0,01149885	0,005319157	0,108189181	0,333614509
hist_liverpancreas	0,006187374	0,00639936	0,006167221	0,00639936	0,003628742	0,98980102	-0,32571367
fam_pd_3rddegree	0,006187374	0,00639936	0,006205928	0,00609939	0,004536971	0,432656734	0,299865861
beta_cryptoxanthin_ug	0,00610095	0,01209879	0,006118917	0,0119988	0,004878224	0,309669033	0,294493936
fat_energypercentage	0,00610095	0,01209879	0,006118373	0,01179882	0,005627913	0,050494951	0,285576078
med_metformin	0,006187374	0,00639936	0,006204915	0,00579942	0,008039946	1,00E-04	0,28348752
lutein_ug	0,00610095	0,01209879	0,006116138	0,01189881	0,004133105	0,861413859	0,248944698
copper_mg	0,00610095	0,01209879	0,006115884	0,01159884	0,005652908	0,104389561	0,244777125
vitB2_mg	0,00610095	0,01209879	0,00608634	0,0129987	0,005108468	0,169483052	-0,239460916
iron_nonhaem_mg	0,00610095	0,01209879	0,006086991	0,01279872	0,007481388	0,00059994	-0,228794648
med_otherantidepressant	0,006187374	0,00639936	0,006201453	0,00579942	0,004931468	0,224977502	0,227538608
hist_asthma	0,006187374	0,00639936	0,006201445	0,00609939	0,006081999	0,01619838	0,227417467
hist_hypertension	0,006187374	0,00639936	0,006173813	0,00629937	0,00485437	0,207279272	-0,219179035
calcium_mg	0,00610095	0,01209879	0,006087722	0,01219878	0,006832685	0,00229977	-0,216822902
iron_total_mg	0,00610095	0,01209879	0,00608773	0,01269873	0,006713736	0,00269973	-0,216684983
fam_otherneurologic	0,006187374	0,00639936	0,006200306	0,00619938	0,004332008	0,635436456	0,209010385
fibers_total_g	0,00610095	0,01209879	0,006088491	0,01229877	0,007832524	0,00079992	-0,204216104
med_antiepileptic	0,006187374	0,00639936	0,006174951	0,00639936	0,004060144	0,729327067	-0,200788101
hist_copd	0,006187374	0,00639936	0,006176109	0,00639936	0,006382075	0,00709929	-0,18205912
vitD_total_ug	0,00610095	0,01209879	0,006111924	0,01189881	0,004798874	0,366863314	0,179874841
hist_hypercholesterolemia	0,006187374	0,00639936	0,006176478	0,00669933	0,004200978	0,730626937	-0,176102713
gamma_tocopherol_mg	0,00610095	0,01209879	0,006090482	0,01279872	0,004779484	0,365863414	-0,171571567
folateequivalent_ug	0,00610095	0,01209879	0,006111407	0,01129887	0,007458461	0,00039996	0,171399954

variable	PDstatus_univar_R2	PDstatus_univar_p	PDstatus_model_R2	PDstatus_model_p	variable_model_R2	variable_model_p	percShift_R2
vitB12_ug	0,00610095	0,01209879	0,00609084	0,0129987	0,004823686	0,339166083	-0,165701373
hist_cholecystectomy	0,006187374	0,00639936	0,006178228	0,00639936	0,005448445	0,04959504	-0,147818673
carbstotal_g	0,00610095	0,01209879	0,006109447	0,01189881	0,00462931	0,471652835	0,139275261
med_benzodiazepines	0,006187374	0,00639936	0,006195871	0,00589941	0,005065533	0,138786121	0,137327181
fam_nonpd_neurodegenerative	0,006187374	0,00639936	0,006180102	0,00639936	0,004343773	0,621637836	-0,117536018
hist_diabetesmellitus	0,006187374	0,00639936	0,006180102	0,00619938	0,007604413	0,00029997	-0,117526213
sodium_mg	0,00610095	0,01209879	0,006107023	0,01209879	0,004810451	0,340765923	0,0995512
potassium_mg	0,00610095	0,01209879	0,006106553	0,01179882	0,005950433	0,01779822	0,091843416
folate_ug	0,00610095	0,01209879	0,006106256	0,01159884	0,007608242	0,00029997	0,086966173
hist_gi_cancer	0,006187374	0,00639936	0,006182091	0,00629937	0,004829155	0,275872413	-0,085380893
med_betasympathomimetic_inhaler	0,006187374	0,00639936	0,006182334	0,00629937	0,005783306	0,0239976	-0,081462687
hist_tonsillectomy	0,006187374	0,00639936	0,006192231	0,00639936	0,004234257	0,703029697	0,078503203
med_corticosteroids_systemic	0,006187374	0,00639936	0,006191907	0,00609939	0,003917898	0,846815318	0,073262187
vitC_mg	0,00610095	0,01209879	0,006104947	0,01219878	0,004393238	0,708629137	0,06552525
proteinanimal_g	0,00610095	0,01209879	0,006104504	0,01219878	0,00501959	0,216178382	0,058255778
hist_kidneydisease	0,006187374	0,00639936	0,006190806	0,0059994	0,003859743	0,930706929	0,055460382
iron_haem_mg	0,00610095	0,01209879	0,006102826	0,0119988	0,006139366	0,01169883	0,030748625
kcal	0,00610095	0,01209879	0,006102458	0,01189881	0,006113658	0,01209879	0,024721408
vitB1_mg	0,00610095	0,01209879	0,006102419	0,01219878	0,005020418	0,215178482	0,024077645
cholesterol_mg	0,00610095	0,01209879	0,0060997	0,01249875	0,0047482	0,389461054	-0,020489231
hist_neurologicalother	0,006187374	0,00639936	0,006188186	0,00639936	0,004432769	0,529647035	0,013119202
alcohol_unitsweek	0,006532353	0,00939906	0,006531893	0,00929907	0,005370476	0,184081592	-0,007043299
fam_diabetes	0,006187374	0,00639936	0,006187046	0,00639936	0,003882496	0,95880412	-0,005303652
fam_cancer	0,006187374	0,00639936	0,006187575	0,00639936	0,00545995	0,04269573	0,003242007
kJ	0,00610095	0,01209879	0,006101075	0,01169883	0,00690237	0,00169983	0,002044052
proteinplant_g	0,00610095	0,01209879	0,006101073	0,01159884	0,008238538	1,00E-04	0,002022665
med_statin	0,006187374	0,00639936	0,006187374	0,00629937	0,006026207	0,00979902	2,73E-12
fam_otherautoimmune	0,006187374	0,00639936	0,006187374	0,00639936	0,004280986	0,665033497	0

Supplementary table 1A: variable selection NL cohort

Screening for the influence of assessed variables on the relationship between PD status and overall microbiome composition.
Variables were incorporated in a model with PD status (overall microbiome composition ~ PD status + variable) to assess the extend to which the variable changes the explained variance (R2) of PD status on overall microbiome composition.
Variables with >10 percent shift in the R2 (percShift_R2) of PD status, compared to the univariable R2 of PD status (calculated for each comparison separately dependent on the removal of samples due to missing data of the variable investigated) were added to the list of nuisance variables.

**variable**	**PDstatus_univar_R2**	**PDstatus_univar_p**	**PDstatus_model_R2**	**PDstatus_model_p**	**variable_model_R2**	**variable_model_p**	**percShift_R2**
DNA_extraction_batch	0,011758623	0,00039996	0,005847585	0,874412559	0,112134749	0,482251775	-50,26981812
PCR_batch	0,011758623	0,00039996	0,009360723	0,00849915	0,096103782	0,04579542	-20,39269754
csi_total	0,011758623	0,00039996	0,009732188	0,00669933	0,009113853	0,01889811	-17,23360772
wexner_total	0,01145169	0,00089991	0,010208533	0,00469953	0,007228149	0,375662434	-10,85566512
nrofreads_seqtab_nolengthfilt	0,011758623	0,00039996	0,010505406	0,00209979	0,009053932	0,01759824	-10,65785774
stool_control_avg	0,011702209	0,00059994	0,010482695	0,00249975	0,010052251	0,00379962	-10,42123134
romeIII_PDS	0,011758623	0,00039996	0,010714015	0,00169983	0,006673375	0,621337866	-8,883759855
romeIII_functionaldyspepsia	0,011758623	0,00039996	0,010741597	0,00149985	0,006968301	0,455154485	-8,649197572
ffq_saturated_fatty_acids_en	0,011758623	0,00039996	0,010810045	0,00119988	0,007871577	0,131086891	-8,067081145
ffq_pyridoxine_mg	0,011758623	0,00039996	0,012550263	0,00019998	0,00891577	0,02489751	6,732416795
ffq_iron_mg	0,011758623	0,00039996	0,012524135	0,00019998	0,008382286	0,064993501	6,510216419
med_anacidic_oldhels	0,011758623	0,00039996	0,011036858	0,00149985	0,007322582	0,291270873	-6,138176995
ffq_peas	0,011758623	0,00039996	0,011129004	0,00139986	0,00694001	0,473152685	-5,354529756
ffq_vitamin_A_retinol_activity_equivalents_ug	0,011758623	0,00039996	0,01237143	0,00019998	0,007300057	0,299570043	5,211556615
stoolconsistency_avg	0,011898995	0,00029997	0,011279974	0,00059994	0,011631408	0,00069993	-5,202294224
stoolconsistency_avg_NMDATscore	0,011898995	0,00029997	0,011290627	0,00059994	0,011166488	0,00079992	-5,112764478
ffq_nut_total	0,011758623	0,00039996	0,011186544	0,00079992	0,006924831	0,483451655	-4,865187054
ffq_fructose_g	0,011758623	0,00039996	0,011199988	0,00079992	0,009617863	0,00809919	-4,750850921
ffq_berry_quark	0,011758623	0,00039996	0,01120693	0,0009999	0,007280572	0,323767623	-4,691817706
ffq_carotenoids_ug	0,011758623	0,00039996	0,011208475	0,00069993	0,008980016	0,01849815	-4,678673968
romeIII_IBS_C	0,011758623	0,00039996	0,011216228	0,00089991	0,007684675	0,202379762	-4,612745589
ffq_pasta_box	0,011758623	0,00039996	0,012295705	0,00039996	0,006396826	0,719328067	4,567561562
ffq_vitamin_B12_ug	0,011758623	0,00039996	0,012247356	0,00019998	0,007379831	0,278772123	4,156379505
ffq_selenium_ug	0,011758623	0,00039996	0,01224164	0,00029997	0,008000825	0,112088791	4,10776864
ffq_fat_en	0,011758623	0,00039996	0,011276892	0,00049995	0,007489583	0,219678032	-4,096835522
ffq_salt_g	0,011758623	0,00039996	0,012234825	0,00019998	0,008069888	0,086491351	4,049809232
ffq_sodium_mg	0,011758623	0,00039996	0,012231411	0,00029997	0,008067252	0,086791321	4,020779296
ffq_trans_fatty_acids_en	0,011758623	0,00039996	0,011309657	0,00049995	0,007899193	0,131786821	-3,818181685

variable	PDstatus_univar_R2	PDstatus_univar_p	PDstatus_model_R2	PDstatus_model_p	variable_model_R2	variable_model_p	percShift_R2
ffq_zinc_mg	0,011758623	0,00039996	0,012206683	0,00029997	0,00902997	0,02109789	3,810480804
ffq_othet_fruit_juice	0,011758623	0,00039996	0,012205073	0,00029997	0,0088871	0,02729727	3,796790912
ffq_tofu	0,011758623	0,00039996	0,01134709	0,00059994	0,009564615	0,02729727	-3,499836918
ffq_minced_meat	0,011758623	0,00039996	0,011360584	0,00059994	0,007873929	0,124587541	-3,38507827
ffq_potato_box_oven_cooked	0,011758623	0,00039996	0,011363502	0,00069993	0,006921201	0,482051795	-3,360270065
ffq_nonsugar_sodas	0,011758623	0,00039996	0,011368405	0,00049995	0,006556048	0,667733227	-3,318566968
ffq_cholesterol_mg	0,011758623	0,00039996	0,012145289	0,00029997	0,007189522	0,341265873	3,288361637
romeIII_IBS	0,011758623	0,00039996	0,01137264	0,00059994	0,00759054	0,189081092	-3,282556553
ffq_rice	0,011758623	0,00039996	0,011380774	0,0009999	0,007995369	0,109089091	-3,213380139
ffq_protein_g	0,011758623	0,00039996	0,012127721	0,00029997	0,008277937	0,062493751	3,138955068
ffq_fish_other	0,011758623	0,00039996	0,011390332	0,00059994	0,007174634	0,359464054	-3,132096784
ffq_wine_total	0,011758623	0,00039996	0,011393673	0,00059994	0,00733712	0,292470753	-3,103679307
ffq_starch_g	0,011758623	0,00039996	0,012121436	0,00039996	0,007599881	0,205779422	3,085509213
ffq_thiamin_mg	0,011758623	0,00039996	0,012108897	0,00029997	0,009217041	0,01649835	2,9788673
ffq_veggies_fresh	0,011758623	0,00039996	0,011419067	0,00059994	0,006469633	0,750324968	-2,887722754
ffq_sugars_total_g	0,011758623	0,00039996	0,011420712	0,00049995	0,008424019	0,054594541	-2,87373044
ffq_salt_cracker	0,011758623	0,00039996	0,011422237	0,00059994	0,006975808	0,454654535	-2,860764128
med_antihistaminic	0,01184978	0,00059994	0,011525673	0,00079992	0,007334191	0,346865313	-2,735130988
ffq_fish_total	0,011758623	0,00039996	0,01207632	0,00029997	0,005995901	0,904509549	2,701822548
ffq_pasta_total	0,011758623	0,00039996	0,012075088	0,00029997	0,007107673	0,385961404	2,69134677
ffq_fat_g	0,011758623	0,00039996	0,012069549	0,00029997	0,007011998	0,432656734	2,644240781
ffq_niacin_mg	0,011758623	0,00039996	0,012061028	0,00029997	0,008168835	0,078592141	2,571768913
ffq_kJ	0,011758623	0,00039996	0,01206016	0,00029997	0,008196545	0,075592441	2,564392237
med_statin	0,011758623	0,00039996	0,011459425	0,00039996	0,006624626	0,668333167	-2,544496445
ffq_kcal	0,011758623	0,00039996	0,012056485	0,00029997	0,008211649	0,073292671	2,533138945
ffq_mandarin	0,011758623	0,00039996	0,011465365	0,00049995	0,008394823	0,063493651	-2,493979275
ffq_red_wine	0,011758623	0,00039996	0,011478554	0,00049995	0,007228669	0,343965603	-2,381821116
ffq_sauerkraut	0,011758623	0,00039996	0,01148507	0,00039996	0,006418898	0,700829917	-2,326403255
ffq_veggies_total	0,011758623	0,00039996	0,011485487	0,00049995	0,008580136	0,04169583	-2,322854085
ffq_fried_potato	0,011758623	0,00039996	0,01148611	0,00039996	0,008330372	0,058894111	-2,317559097
ffq_iodine_ug	0,011758623	0,00039996	0,01202573	0,00029997	0,008059805	0,096890311	2,271584903
ffq_fatty_acid_22_6_DHA_mg	0,011758623	0,00039996	0,012025278	0,00029997	0,007690504	0,172182782	2,26773794
ffq_carrots_cooked	0,011758623	0,00039996	0,011494651	0,00029997	0,006903494	0,508149185	-2,244926434
ffq_cucumber	0,011758623	0,00039996	0,011497542	0,00059994	0,00608065	0,894710529	-2,220336103
ffq_doughnut	0,011758623	0,00039996	0,012015254	0,00029997	0,007425515	0,265673433	2,182488015
ffq_broccoli	0,011758623	0,00039996	0,012011396	0,00029997	0,007129186	0,382761724	2,149678111

variable	PDstatus_univar_R2	PDstatus_univar_p	PDstatus_model_R2	PDstatus_model_p	variable_model_R2	variable_model_p	percShift_R2
ffq_sugar	0,011758623	0,00039996	0,011506694	0,00049995	0,007126755	0,402359764	-2,142505007
ffq_hamburger	0,011758623	0,00039996	0,011509323	0,00059994	0,007922597	0,117988201	-2,120148013
ffq_sodas_total	0,011758623	0,00039996	0,011513096	0,00039996	0,005773974	0,933306669	-2,088061771
ffq_milk_products_liquid	0,011758623	0,00039996	0,01151973	0,00039996	0,00767548	0,186181382	-2,031640563
ffq_monounsaturated_fatty_acids_en	0,011758623	0,00039996	0,011521398	0,00039996	0,00728949	0,296470353	-2,017454588
ffq_folate_ug	0,011758623	0,00039996	0,011995524	0,00029997	0,009934541	0,00439956	2,014699236
ffq_liquor	0,011758623	0,00039996	0,01198999	0,00039996	0,006879519	0,503549645	1,967639941
ffq_cauliflower_fresh	0,011758623	0,00039996	0,011986372	0,00029997	0,009661078	0,01009899	1,936870485
ffq_potato	0,011758623	0,00039996	0,01153298	0,00059994	0,00632998	0,830516948	-1,918956602
ffq_organic_acids_g	0,011758623	0,00039996	0,011983732	0,00029997	0,006312912	0,693530647	1,914419438
ffq_artificially_sweetened_cider	0,011758623	0,00039996	0,011983418	0,00029997	0,00690588	0,444155584	1,911747256
ffq_glucose_g	0,011758623	0,00039996	0,01153551	0,00049995	0,008864029	0,02719728	-1,897445683
stoolfrequency_avg	0,012261259	0,00039996	0,012030692	0,00049995	0,011597035	0,00049995	-1,880453571
ffq_cauliflower_total	0,011758623	0,00039996	0,011976342	0,00029997	0,008780006	0,03239676	1,85156718
med_ssri	0,011758623	0,00039996	0,011559082	0,00049995	0,007371711	0,273172683	-1,696975511
med_mouthdesinfectant_lastmonth	0,011956616	1,00E-04	0,011754727	1,00E-04	0,007606313	0,223677632	-1,688509993
ffq_saturated_fatty_acids_g	0,011758623	0,00039996	0,011957139	0,00029997	0,007067092	0,405559444	1,688254448
romeIII_IBS_D	0,011758623	0,00039996	0,011953238	0,00039996	0,007442534	0,265073493	1,655086841
ffq_spread	0,011758623	0,00039996	0,011564562	0,00059994	0,007262074	0,316468353	-1,650376446
ffq_monounsaturated_fatty_acids_g	0,011758623	0,00039996	0,011951899	0,00029997	0,006795031	0,560643936	1,643693377
ffq_fatty_acid_20_5_EPA_mg	0,011758623	0,00039996	0,011951269	0,00029997	0,006754117	0,574642536	1,638336487
ffq_licorise	0,011758623	0,00039996	0,011566627	0,00059994	0,008785283	0,03749625	-1,6328109
ffq_tuna_total	0,011758623	0,00039996	0,011567404	0,00049995	0,006538554	0,560743926	-1,626203083
romeIII_functionalbloating	0,011758623	0,00039996	0,011569994	0,00049995	0,006874323	0,505549445	-1,60417795
ffq_nut_salted	0,011758623	0,00039996	0,011572372	0,00039996	0,007273175	0,317068293	-1,583952181
ffq_phosphorus_mg	0,011758623	0,00039996	0,011940785	0,00029997	0,008539996	0,04129587	1,549175011
ffq_tuna_oil	0,011758623	0,00039996	0,011581054	0,00039996	0,006489134	0,578942106	-1,510121344
ffq_alpha_linolenic_acid_en	0,011758623	0,00039996	0,011583231	0,00039996	0,006466609	0,755324468	-1,491606653
ffq_fishfood_salmon	0,011758623	0,00039996	0,011933989	0,00029997	0,00852988	0,0459954	1,491378192
ffq_other_veggie_foods	0,011758623	0,00039996	0,011585718	0,00049995	0,00867589	0,04569543	-1,470449538
ffq_long_drink_sugar	0,011758623	0,00039996	0,011586687	0,00039996	0,00654189	0,671432857	-1,462209913
ffq_tuna_water	0,011758623	0,00039996	0,01158698	0,00069993	0,006601142	0,667533247	-1,45972435
ffq_porridge	0,011758623	0,00039996	0,011927588	0,00029997	0,007575654	0,241275872	1,436944325
hist_psychosis	0,011758623	0,00039996	0,011591802	0,00049995	0,007045206	0,410258974	-1,418714034
ffq_carbohydrates_en	0,011758623	0,00039996	0,011593167	0,00039996	0,006993051	0,450554945	-1,407107269
ffq_grain_products	0,011758623	0,00039996	0,011919534	0,00039996	0,007371683	0,298270173	1,368450293

variable	PDstatus_univar_R2	PDstatus_univar_p	PDstatus_model_R2	PDstatus_model_p	variable_model_R2	variable_model_p	percShift_R2
ffq_liver_meal	0,011758623	0,00039996	0,011918795	0,00029997	0,006598653	0,679132087	1,362164341
romeIII_functionaldiarrhea	0,011758623	0,00039996	0,011598802	0,00039996	0,00721863	0,349765023	-1,359183105
romeIII_IBS_U	0,011758623	0,00039996	0,011600031	0,00049995	0,006442507	0,599740026	-1,348726918
ffq_quark	0,011758623	0,00039996	0,0119164	0,00029997	0,005545323	0,903909609	1,341794112
ffq_butter_oil_butterspread	0,011758623	0,00039996	0,011916057	0,00029997	0,007023607	0,429357064	1,338882988
ffq_sardines	0,011758623	0,00039996	0,011915995	0,00029997	0,006339687	0,782221778	1,338352394
ffq_liver_sausage	0,011758623	0,00039996	0,011602331	0,00039996	0,006285614	0,716828317	-1,32916888
ffq_orange_juice	0,011758623	0,00039996	0,011603253	0,00049995	0,006960863	0,468853115	-1,321329584
ffq_banan	0,011758623	0,00039996	0,011912861	0,00039996	0,006496651	0,717428257	1,311702897
ffq_cholesterol_lowering_spread	0,011758623	0,00039996	0,011605754	0,00059994	0,00675158	0,577542246	-1,300056842
hist_ibd	0,011758623	0,00039996	0,011910878	0,00029997	0,00755141	0,312068793	1,294837749
ffq_grapes	0,011758623	0,00039996	0,011910858	0,00029997	0,007731627	0,197780222	1,294668165
ffq_muesli	0,011758623	0,00039996	0,011910846	0,00039996	0,007036009	0,431356864	1,294560364
ffq_bread_porridge	0,011758623	0,00039996	0,011909048	0,00029997	0,007456532	0,269773023	1,279273205
ffq_cabbage_foods	0,011758623	0,00039996	0,011609058	0,00049995	0,008711424	0,04359564	-1,271959171
ffq_vitamin_C_mg	0,011758623	0,00039996	0,011611285	0,00049995	0,01004941	0,00379962	-1,253020277
ffq_saccarose_g	0,011758623	0,00039996	0,011613206	0,00039996	0,00746252	0,245175482	-1,236684364
ffq_ethanol_g	0,011758623	0,00039996	0,01161396	0,00039996	0,007875663	0,138886111	-1,230270271
ffq_vitamin_E_mg	0,011758623	0,00039996	0,011614175	0,00049995	0,007540805	0,202079792	-1,228442416
ffq_avocado	0,011758623	0,00039996	0,011614465	0,00049995	0,007122862	0,385161484	-1,225980423
ffq_sausage	0,011758623	0,00039996	0,011615943	0,00039996	0,0078957	0,145985401	-1,213408568
ffq_trans_fatty_acids_g	0,011758623	0,00039996	0,011897848	0,00029997	0,007442116	0,256274373	1,184022427
romeIII_functionalconstipation	0,011758623	0,00039996	0,011620646	0,00049995	0,008344568	0,070492951	-1,173408678
ffq_apple	0,011758623	0,00039996	0,011621413	0,00049995	0,007361537	0,279372063	-1,166890797
ffq_pickles	0,011758623	0,00039996	0,011626409	0,00049995	0,008898119	0,04369563	-1,124403074
med_lungmeds_oldhels	0,011758623	0,00039996	0,01162675	0,00049995	0,007629393	0,189181082	-1,121497248
ffq_licorice_salted	0,011758623	0,00039996	0,011627442	0,00049995	0,00719602	0,341665833	-1,115619314
ffq_candies_without_licorice	0,011758623	0,00039996	0,011886408	0,00039996	0,00542732	0,934006599	1,08673037
ffq_seafood	0,011758623	0,00039996	0,011884818	0,00029997	0,005751879	0,879212079	1,073214012
ffq_carbohydrates_g	0,011758623	0,00039996	0,011883709	0,00029997	0,008369347	0,059794021	1,063783289
hist_diabetesmellitus	0,011758623	0,00039996	0,011635253	0,00049995	0,009165261	0,01889811	-1,049184895
ffq_bacon	0,011758623	0,00039996	0,011880679	0,00029997	0,007514845	0,256974303	1,038014668
ffq_calsium_mg	0,011758623	0,00039996	0,011638984	0,00039996	0,008363589	0,057694231	-1,017460314
med_kortisone_oldhels	0,011758623	0,00039996	0,011642031	0,00049995	0,006662765	0,623337666	-0,991541635
hist_polyneuropathy	0,011779149	0,00049995	0,011895192	0,00039996	0,006283823	0,685631437	0,98515572
ffq_tomato	0,011758623	0,00039996	0,011642875	0,00049995	0,006302036	0,812318768	-0,984371045

variable	PDstatus_univar_R2	PDstatus_univar_p	PDstatus_model_R2	PDstatus_model_p	variable_model_R2	variable_model_p	percShift_R2
ffq_blood_meal	0,011758623	0,00039996	0,011644759	0,00039996	0,006023572	0,912108789	-0,968346078
ffq_orange	0,011758623	0,00039996	0,011871736	0,00029997	0,009648977	0,00819918	0,96195815
ffq_potassium_mg	0,011758623	0,00039996	0,011871372	0,00029997	0,008955902	0,0209979	0,958864938
ffq_processed_cheese	0,011758623	0,00039996	0,011871324	0,00039996	0,00689512	0,500349965	0,958454165
ffq_candies_total	0,011758623	0,00039996	0,011871125	0,00039996	0,005398827	0,95220478	0,956764179
ffq_vitamin_D_ug	0,011758623	0,00039996	0,011870294	0,00029997	0,008029492	0,096390361	0,949697893
ffq_margarine	0,011758623	0,00039996	0,011647748	0,00049995	0,007082796	0,394060594	-0,942926146
ffq_fishfood_other	0,011758623	0,00039996	0,011864541	0,00029997	0,006397066	0,729127087	0,900770396
ffq_berries	0,011758623	0,00039996	0,011653718	0,00049995	0,007101408	0,397760224	-0,892156519
ffq_riboflavine_mg	0,011758623	0,00039996	0,011654522	0,00039996	0,00847703	0,04839516	-0,885319174
ffq_polyunsaturated_fatty_acids_g	0,011758623	0,00039996	0,01185843	0,00029997	0,006514609	0,725927407	0,848797008
ffq_sliced_sausage	0,011758623	0,00039996	0,011658989	0,00039996	0,008388631	0,075792421	-0,847331611
ffq_salad	0,011758623	0,00039996	0,01185597	0,00039996	0,005748506	0,98080192	0,827876708
ffq_white_wine	0,011758623	0,00039996	0,011662542	0,00049995	0,006653637	0,635436456	-0,817109404
hist_appendectomy	0,011758623	0,00039996	0,011854648	0,00029997	0,007060654	0,411158884	0,816632559
ffq_cake	0,011758623	0,00039996	0,011664011	0,00039996	0,006028507	0,883511649	-0,804617052
ffq_home_beer_non_alcoholic_or_very_mild	0,011758623	0,00039996	0,011665804	0,00049995	0,00706692	0,421257874	-0,789369564
ffq_fatty_acid_22_6_DHA_en	0,011758623	0,00039996	0,01184455	0,00039996	0,008455274	0,0469953	0,730754404
ffq_sitrus_tot	0,011758623	0,00039996	0,011672918	0,00049995	0,009682696	0,00809919	-0,72887147
ffq_fatty_acid_20_5_EPA_en	0,011758623	0,00039996	0,011844103	0,00039996	0,007825802	0,128687131	0,726958622
ffq_cauliflower_cooked	0,011758623	0,00039996	0,01184224	0,00039996	0,006645144	0,645735426	0,711110291
hist_cholecystectomy	0,011758623	0,00039996	0,011675132	0,00049995	0,007648094	0,173082692	-0,710037841
ffq_long_drink_total	0,011758623	0,00039996	0,011675325	0,00039996	0,006744574	0,574942506	-0,708400295
med_diarrhea	0,01184978	0,00059994	0,011767564	0,00059994	0,00685345	0,544645535	-0,693817601
med_constipation	0,01184978	0,00059994	0,011928492	0,00049995	0,006978171	0,540445955	0,664253538
ffq_strong_wine	0,011758623	0,00039996	0,011833606	0,00039996	0,005346716	0,925607439	0,637688013
ffq_magnesium_mg	0,011758623	0,00039996	0,011833132	0,00029997	0,008610077	0,03879612	0,633653697
ffq_liver	0,011758623	0,00039996	0,011832015	0,00029997	0,006836241	0,525647435	0,624158149
ffq_jam	0,011758623	0,00039996	0,011686407	0,00049995	0,008031551	0,143485651	-0,61415564
ffq_fiber_g	0,011758623	0,00039996	0,011687006	0,00039996	0,008472276	0,054094591	-0,60905639
ffq_alpha_linolenic_acid_mg	0,011758623	0,00039996	0,011829575	0,00029997	0,006966609	0,468853115	0,603400585
ffq_cider	0,011758623	0,00039996	0,011829429	0,00029997	0,007758036	0,188381162	0,602164555
ffq_lactose_g	0,011758623	0,00039996	0,011689586	0,00039996	0,007579619	0,232876712	-0,587117212
med_antibiotics_lastmonth	0,012106477	0,00019998	0,01203563	0,00029997	0,005335732	0,97190281	-0,585201527
ffq_fruit_juice_total	0,011758623	0,00039996	0,011827411	0,00029997	0,008927572	0,02719728	0,584999413
ffq_vitamin_K_total_ug	0,011758623	0,00039996	0,011691306	0,00039996	0,007159716	0,376362364	-0,572487555

variable	PDstatus_univar_R2	PDstatus_univar_p	PDstatus_model_R2	PDstatus_model_p	variable_model_R2	variable_model_p	percShift_R2
hist_liverpancreas	0,011711177	0,00019998	0,011645053	0,00029997	0,0064	0,785421458	-0,564624276
romeIII_IBS_M	0,011758623	0,00039996	0,011698664	0,00039996	0,005929229	0,944405559	-0,509914131
ffq_sparkling_wine	0,011758623	0,00039996	0,011817965	0,00049995	0,007059981	0,417558244	0,504665463
hist_ent_oldhels	0,011758623	0,00039996	0,011701199	0,00039996	0,007322175	0,278172183	-0,488353157
ffq_protein_en	0,011758623	0,00039996	0,011815373	0,00029997	0,006791757	0,559744026	0,482621075
ffq_rye_bread	0,011758623	0,00039996	0,011811984	0,00039996	0,006572499	0,705529447	0,453805223
ffq_pizza	0,011758623	0,00039996	0,011811458	0,00039996	0,007537371	0,205779422	0,4493284
ffq_ice_cream	0,011758623	0,00039996	0,011706705	0,00039996	0,005965523	0,941605839	-0,44152785
ffq_soups	0,011758623	0,00039996	0,011708456	0,00039996	0,010629358	0,00229977	-0,426641413
age	0,011758623	0,00039996	0,011808307	0,00039996	0,006374721	0,813618638	0,422533349
ffq_beetroot	0,011758623	0,00039996	0,011709067	0,00039996	0,006519952	0,683831617	-0,421447136
ffq_spaghetti	0,011758623	0,00039996	0,011807673	0,00039996	0,007462042	0,239276072	0,417141585
ffq_meat	0,011758623	0,00039996	0,011807268	0,00039996	0,007596553	0,206979302	0,413692871
ffq_artificially_sweetened_long_drink	0,011758623	0,00039996	0,01180709	0,00039996	0,007327624	0,356364364	0,412178395
ffq_cacao	0,011758623	0,00039996	0,011805332	0,00039996	0,006215601	0,825017498	0,397232177
ffq_eggs	0,011758623	0,00039996	0,01180351	0,00039996	0,005798026	0,949705029	0,381735831
ffq_herring	0,011758623	0,00039996	0,011715773	0,00039996	0,005766653	0,875012499	-0,364414011
med_opioids	0,011758623	0,00039996	0,011800584	0,00039996	0,007354513	0,279272073	0,356856518
ffq_salad_and_grate	0,011758623	0,00039996	0,011799401	0,00039996	0,006182689	0,888711129	0,346794849
ffq_alcohol_mild	0,011758623	0,00039996	0,011719489	0,00039996	0,008041333	0,137886211	-0,332808596
ffq_sweet_bread	0,011758623	0,00039996	0,011796965	0,00039996	0,006796901	0,557344266	0,326077301
ffq_mixed_bread	0,011758623	0,00039996	0,011721138	0,00039996	0,00666984	0,629537046	-0,318789262
ffq_white_bread	0,011758623	0,00039996	0,011721138	0,00039996	0,00666984	0,629537046	-0,318789262
ffq_mixed_vegetables	0,011758623	0,00039996	0,011795735	0,00039996	0,007368073	0,299070093	0,315612227
ffq_sausage_like_salami	0,011758623	0,00039996	0,011795649	0,00039996	0,007165519	0,379862014	0,314879575
ffq_fibre_water_soluble_g	0,011758623	0,00039996	0,011795507	0,00039996	0,009420488	0,01079892	0,313675439
ffq_sugar_sodas	0,011758623	0,00039996	0,011794275	0,00039996	0,00463829	0,99650035	0,303194176
ffq_salted_licorise	0,011758623	0,00039996	0,011723986	0,00049995	0,006593105	0,650734927	-0,294566884
ffq_beer_total	0,011758623	0,00039996	0,011724612	0,00039996	0,007829947	0,180081992	-0,289240266
ffq_berry_juice	0,011758623	0,00039996	0,011724795	0,00049995	0,00599826	0,864913509	-0,287683839
ffq_coffee_total	0,011758623	0,00039996	0,011724804	0,00039996	0,007601452	0,182181782	-0,287607922
ffq_instant_coffee	0,011758623	0,00039996	0,011725494	0,00039996	0,007044616	0,423057694	-0,28174201
med_antiepileptic	0,011758623	0,00039996	0,01172568	0,00039996	0,006679791	0,574042596	-0,280159042
med_tricyclicantidepressant	0,011758623	0,00039996	0,011725822	0,00049995	0,006642838	0,577342266	-0,278955031
sex	0,011758623	0,00039996	0,011789199	0,00039996	0,007711088	0,156084392	0,260031685
med_diuretic	0,011758623	0,00039996	0,011789182	0,00039996	0,007143951	0,364763524	0,25988706

variable	PDstatus_univar_R2	PDstatus_univar_p	PDstatus_model_R2	PDstatus_model_p	variable_model_R2	variable_model_p	percShift_R2
ffq_other_fruit	0,011758623	0,00039996	0,011788295	0,00039996	0,007239298	0,319668033	0,252340853
ffq_grate	0,011758623	0,00039996	0,011729038	0,00049995	0,007185234	0,359364064	-0,251606076
ffq_cheese_total	0,011758623	0,00039996	0,011786805	0,00039996	0,007099789	0,390460954	0,239672084
ffq_cider_total	0,011758623	0,00039996	0,01178649	0,00029997	0,00766531	0,206779322	0,236993248
ffq_filter_coffee	0,011758623	0,00039996	0,011784663	0,00039996	0,008294274	0,061993801	0,221456675
med_betablocker	0,011758623	0,00039996	0,011783233	0,00039996	0,007003975	0,436156384	0,209290583
ffq_mayo_salad	0,011758623	0,00039996	0,011734164	0,00049995	0,007321217	0,330266973	-0,208011566
ffq_alcohol_en	0,011758623	0,00039996	0,011734515	0,00039996	0,00805654	0,108189181	-0,20502321
ffq_fruit_total	0,011758623	0,00039996	0,011736552	0,00039996	0,008725409	0,03449655	-0,187703284
ffq_maltose_g	0,011758623	0,00039996	0,011736772	0,00039996	0,007506989	0,254274573	-0,185832679
ffq_whole_meat_cut	0,011758623	0,00039996	0,011779783	0,00039996	0,006990313	0,438756124	0,179950952
ffq_whole_meat	0,011758623	0,00039996	0,011779677	0,00039996	0,007276229	0,304669533	0,179051305
med_ace_ang_oldhels	0,011758623	0,00039996	0,011738085	0,00049995	0,006730021	0,607039296	-0,174664413
ffq_fibre_water_insoluble_g	0,011758623	0,00039996	0,011778853	0,00039996	0,00836048	0,067193281	0,172046331
ffq_broiler_chicken_turkey	0,011758623	0,00039996	0,011739246	0,00039996	0,005592319	0,899910009	-0,164786986
ffq_crispbread	0,011758623	0,00039996	0,011777554	0,00029997	0,00713595	0,378262174	0,160993545
ffq_karelian_pie	0,011758623	0,00039996	0,011774498	0,00039996	0,006757331	0,563943606	0,135010866
ffq_polyunsaturated_fatty_acids_en	0,011758623	0,00039996	0,011744125	0,00039996	0,006505998	0,744125587	-0,12329804
med_anticongestive	0,011758623	0,00039996	0,011745255	0,00039996	0,008027804	0,116988301	-0,113685454
weight	0,011758623	0,00039996	0,01174573	0,00039996	0,006972853	0,466353365	-0,109647053
ffq_chocolade	0,011758623	0,00039996	0,011746386	0,00039996	0,00755802	0,202979702	-0,104072708
height	0,011758623	0,00039996	0,011770848	0,00039996	0,00744028	0,239576042	0,103966351
ffq_linoleic_acid_mg	0,011758623	0,00039996	0,011747892	0,00039996	0,006726068	0,605839416	-0,091263078
ffq_galactose_g	0,011758623	0,00039996	0,011769005	0,00039996	0,006619567	0,651334867	0,088288302
med_calciumantagonist	0,011758623	0,00039996	0,011768972	0,00039996	0,008190715	0,072092791	0,088015085
ffq_linoleic_acid_en	0,011758623	0,00039996	0,011749438	0,00049995	0,006393055	0,801819818	-0,078112592
ffq_olives	0,011758623	0,00039996	0,011767269	0,00059994	0,008343845	0,070592941	0,073531436
ffq_fish_soups_total	0,011758623	0,00039996	0,011750019	0,00039996	0,007168897	0,378462154	-0,073172542
ffq_cooked_potato	0,011758623	0,00039996	0,011766979	0,00039996	0,006087777	0,899810019	0,071060286
ffq_pastry	0,011758623	0,00039996	0,011750268	0,00039996	0,007268413	0,330566943	-0,071058112
ffq_apple_juice	0,011758623	0,00039996	0,011750402	0,00039996	0,008494651	0,076092391	-0,069917936
ffq_cookie	0,011758623	0,00039996	0,011765134	0,00039996	0,006734955	0,576342366	0,05537179
ffq_saccarose_en	0,011758623	0,00039996	0,011764719	0,00029997	0,006661528	0,620337966	0,05184383
ffq_sterols_mg	0,011758623	0,00039996	0,011752788	0,00039996	0,008622658	0,03639636	-0,049627333
ffq_berries_total	0,011758623	0,00039996	0,011764146	0,00039996	0,006983806	0,456854315	0,046968576
ffq_bread_total	0,011758623	0,00039996	0,011764117	0,00039996	0,007167838	0,367663234	0,046724566

variable	PDstatus_univar_R2	PDstatus_univar_p	PDstatus_model_R2	PDstatus_model_p	variable_model_R2	variable_model_p	percShift_R2
ffq_tea	0,011758623	0,00039996	0,011763782	0,00039996	0,007365019	0,276272373	0,043877668
ffq_cottage_cheese	0,011758623	0,00039996	0,011762923	0,00039996	0,007313941	0,309269073	0,036565215
ffq_cold_cuts_all	0,011758623	0,00039996	0,011762223	0,00039996	0,006760905	0,569243076	0,030613807
ffq_mushrooms	0,011758623	0,00039996	0,011755313	0,00039996	0,007588205	0,211878812	-0,028149393
ffq_pan_coffee	0,011758623	0,00039996	0,0117614	0,00039996	0,007535007	0,287671233	0,023613187
ffq_tea_like_camomilla_tea	0,011758623	0,00039996	0,011760564	0,00039996	0,005823446	0,891710829	0,01650492
hist_hypothyroid	0,011758623	0,00039996	0,011760439	0,00039996	0,006714391	0,599440056	0,015439647
ffq_cereals	0,011758623	0,00039996	0,011757576	0,00039996	0,007669946	0,186881312	-0,008907721
ffq_bun	0,011758623	0,00039996	0,011757713	0,00039996	0,00712437	0,390360964	-0,007737806
ffq_oil_supplement	0,011758623	0,00039996	0,011759433	0,00039996	0,006572391	0,683531647	0,006889082
lactose_intolerance	0,011758623	0,00039996	0,011758216	0,00039996	0,006697202	0,614838516	-0,003458179
stool_desiredfreq_avg	0,012371428	0,00029997	0,01237175	0,00029997	0,009616886	0,01209879	0,002598164
BMI	0,011758623	0,00039996	0,011758675	0,00039996	0,007944665	0,112488751	0,000438232

Supplementary table 4A: Collinearity of selected confounders NL cohort

Selected confounders were screened for (multi-)collinearity using a generalized variance inflation factor (GVIF). Variables with GVIF >3 were excluded from the final model.

GVIF overview of all selected confounders, no variables with GVIF >3, therefore all selected confounders could be included in the final model.

**variable**	**GVIF**
group	1,890077467
DNA_extraction_batch	2,558791015
age	1,170567504
sex	1,14640248
BMI	1,118098739
stoolconsistency_avg	1,356539629
nmsq_constipation	1,251301218
stoolfrequency_avg	1,372054745

Supplementary table 4B: Collinearity of selected confounders FIN cohort
Selected confounders were screened for (multi-)collinearity using a generalized variance inflation factor (GVIF). Variables with GVIF >3 were excluded from the final mode

GVIF overview of all selected confounders

**variable**	**GVIF**
group	35,87519073
DNA_extraction_batch	73644,58541
age	1,41742629
sex	1,564390055
BMI	1,338017042
stoolconsistency_avg	2,010546848
PCR_batch	8279,238652
csi_total	4,966687455
wexner_total	4,105388704
stool_control_avg	2,697706195
nrofreads_seqtab_nolengthfilt	2,310469467

GVIF overview after exclusion of variable with highest GVIF (DNA_extraction_batch)

**variable**	**GVIF**
group	2,135713372
age	1,15793292
sex	1,30996618
BMI	1,252176919
stoolconsistency_avg	1,621263842
PCR_batch	6,402108854
csi_total	4,224393027
wexner_total	3,58595482
stool_control_avg	2,076571684
nrofreads_seqtab_nolengthfilt	2,069712543

GVIF overview after exclusion of wexner_total with a GVIF > 3 and expected to be collinear with other measures of constipation (csi_total, stoolconsistency_avg and stool_control_avg)

**variable**	**GVIF**
group	2,113476544
age	1,15935343
sex	1,235062095
BMI	1,238106873
stoolconsistency_avg	1,617956552
PCR_batch	5,284672516
csi_total	1,934687424
stool_control_avg	2,064211283
nrofreads_seqtab_nolengthfilt	2,016164844

GVIF overview after exclusion of final variable with GVIF >3 (PCR-batch), providing the final model

**variable**	**GVIF**

group	1,231177056
age	1,108039833
sex	1,124546897
BMI	1,106519936
stoolconsistency_avg	1,402166618
csi_total	1,827253438
stool_control_avg	1,873973065
nrofreads_seqtab_nolengthfilt	1,14299298

Supplementary table 5a: Differential abundance analysis at ASV level of NL cohort with DESeq2 and ANCOM

All columns are spread out over two pages, after which the next two pages show the next set of rows, etc.

ASVs are numbered on both pages to easily connect the taxonomy data to the associated statistics.

ASV_nr	Phylum	Class	Order	Family	Genus	Species
1	Actinobacteriota	Actinobacteria	Bifidobacteriales	Bifidobacteriaceae	Bifidobacterium	NA
2	Verrucomicrobiota	Verrucomicrobiae	Verrucomicrobiales	Akkermansiaceae	Akkermansia	muciniphila
3	Firmicutes	Clostridia	Christensenellales	Christensenellaceae	NA	NA
4	Firmicutes	Clostridia	Lachnospirales	Lachnospiraceae	Roseburia	NA
5	Firmicutes	Incertae Sedis	DTU014	NA	NA	NA
6	Firmicutes	Clostridia	Clostridia UCG-014	NA	NA	NA
7	Firmicutes	Clostridia	Christensenellales	Christensenellaceae	Christensenellaceae R-7 group	NA
8	Firmicutes	Clostridia	Clostridia vadinBB60 group	NA	NA	NA
9	Firmicutes	Bacilli	Erysipelotrichales	Erysipelotrichaceae	Holdemanella	NA
10	Bacteroidota	Bacteroidia	Bacteroidales	Bacteroidaceae	Bacteroides	NA
11	Bacteroidota	Bacteroidia	Bacteroidales	Barnesiellaceae	Coprobacter	secundus
12	Bacteroidota	Bacteroidia	Bacteroidales	Bacteroidaceae	Bacteroides	eggerthii
13	Firmicutes	Clostridia	Oscillospirales	[Eubacterium] coprostanoligenes group	NA	NA
14	Firmicutes	Clostridia	Oscillospirales	Ruminococcaceae	Subdoligranulum	NA
15	Firmicutes	Clostridia	Oscillospirales	Ruminococcaceae	Faecalibacterium	prausnitzii
16	Firmicutes	Clostridia	Lachnospirales	Lachnospiraceae	Lachnospira	pectinoschiza
17	Firmicutes	Clostridia	Oscillospirales	Oscillospiraceae	UCG-002	NA
18	Firmicutes	Clostridia	Oscillospirales	UCG-010	NA	NA
19	Firmicutes	Clostridia	Lachnospirales	Lachnospiraceae	NA	NA
20	Bacteroidota	Bacteroidia	Bacteroidales	Bacteroidaceae	Bacteroides	coprocola
21	Firmicutes	Clostridia	Oscillospirales	Oscillospiraceae	UCG-003	NA
22	Bacteroidota	Bacteroidia	Bacteroidales	Rikenellaceae	Alistipes	finegoldii
23	Firmicutes	Clostridia	Clostridia UCG-014	NA	NA	NA
24	Firmicutes	Clostridia	Oscillospirales	Ruminococcaceae	Subdoligranulum	NA
25	Firmicutes	Negativicutes	Veillonellales-Selenomonadales	Veillonellaceae	Dialister	invisus
26	Firmicutes	Clostridia	Lachnospirales	Lachnospiraceae	Anaerostipes	hadrus
27	Bacteroidota	Bacteroidia	Bacteroidales	Barnesiellaceae	Barnesiella	NA
28	Firmicutes	Clostridia	Lachnospirales	Lachnospiraceae	Shuttleworthia	NA
29	Firmicutes	Clostridia	Lachnospirales	Lachnospiraceae	[Ruminococcus] torques group	NA
30	Firmicutes	Clostridia	Clostridia UCG-014	NA	NA	NA
31	Firmicutes	Clostridia	Oscillospirales	Oscillospiraceae	UCG-002	NA
32	Bacteroidota	Bacteroidia	Bacteroidales	Tannerellaceae	Parabacteroides	distasonis

ASV_nr	DESeq_baseMean	DESeq_log2FoldChange	DESeq_lfcSE	DESeq_stat	DESeq_pvalue	DESeq_padj	ANCOM_W	ANCOM_detected_0.7
1	403,424387	-0,569242017	0,522957904	-1,08850447	0,276372464	0,995587377	0	FALSE
2	847,5631407	0,2315891	0,525836723	0,440420172	0,659632818	0,995587377	0	FALSE
3	214,0591493	-0,040101972	0,536399748	-0,074761355	0,94040459	0,995587377	101	FALSE
4	160,8607363	0,742270256	0,885941292	0,837832329	0,402124879	0,995587377	0	FALSE
5	2,154387102	1,112332291	0,898449056	1,238058278	0,215694453	0,995587377	30	FALSE
6	145,5821205	0,515224489	1,56166847	0,329919249	0,741460979	0,995587377	4	FALSE
7	59,82475184	-0,188217424	0,563279186	-0,334145889	0,738269474	0,995587377	0	FALSE
8	1,551767704	0,426535581	1,179111445	0,361743228	0,71754392	0,995587377	0	FALSE
9	32,23710475	-0,083571251	1,625647017	-0,051407993	0,959000416	0,995587377	0	FALSE
10	56,59497155	0,166619871	0,853065961	0,19531886	0,845143333	0,995587377	0	FALSE
11	2,821591624	1,049587067	1,084198471	0,968076505	0,333006163	0,995587377	0	FALSE
12	55,95860606	-0,233417959	1,241683456	-0,187985076	0,85088835	0,995587377	0	FALSE
13	64,07665421	0,421363839	0,795239041	0,52985809	0,596210326	0,995587377	0	FALSE
14	416,0762764	-0,390644849	0,253249325	-1,542530659	0,122944694	0,995587377	0	FALSE
15	65,51157892	0,020984505	1,499472606	0,01399459	0,988834297	0,997588574	0	FALSE
16	90,36088673	-0,400778901	0,458994915	-0,87316632	0,38257243	0,995587377	0	FALSE
17	57,25920625	-0,266667709	1,234533712	-0,216006826	0,828982427	0,995587377	0	FALSE
18	2,430310908	1,841510378	1,253657857	1,468909852	0,141857242	0,995587377	6	FALSE
19	115,6287928	-0,516683486	1,5968135	-0,323571592	0,746262398	0,995587377	0	FALSE
20	71,72274918	2,231740162	1,624270743	1,373995173	0,169443181	0,995587377	3	FALSE
21	129,8012621	0,301203091	0,398076854	0,756645577	0,449262228	0,995587377	0	FALSE
22	46,5062439	-0,517276765	0,858808056	-0,602319414	0,54696154	0,995587377	0	FALSE
23	76,08359171	-0,340756108	1,664425034	-0,204729022	0,837783845	0,995587377	0	FALSE
24	47,25097307	0,324330297	0,662850909	0,489295999	0,624632153	0,995587377	0	FALSE
25	443,2633574	-0,97449575	1,000214464	-0,974286801	0,329914157	0,995587377	0	FALSE
26	16,92869662	1,125527833	1,474150177	0,763509614	0,445159531	0,995587377	0	FALSE
27	47,49233212	0,378082553	1,401824916	0,2697074	0,787385367	0,995587377	0	FALSE
28	2,672018214	-0,592873464	1,129537307	-0,524881702	0,59966543	0,995587377	0	FALSE
29	144,9493941	-0,233210767	0,608552637	-0,383222014	0,701555157	0,995587377	0	FALSE
30	138,1202478	-5,105176417	1,335486228	-3,822709894	0,000131993	0,049101411	0	FALSE
31	172,4414294	0,492317695	0,736613963	0,668352379	0,503908685	0,995587377	2	FALSE
32	86,00723822	0,503792204	0,692731317	0,727254842	0,467069856	0,995587377	0	FALSE

ASV_nr	Phylum	Class	Order	Family	Genus	Species
33	Firmicutes	Clostridia	Lachnospirales	Lachnospiraceae	Lachnospiraceae NK4A136 group	bacterium
34	Firmicutes	Clostridia	Oscillospirales	Oscillospiraceae	NK4A214 group	NA
35	Firmicutes	Clostridia	Christensenellales	Christensenellaceae	Christensenellaceae R-7 group	NA
36	Firmicutes	Clostridia	Peptostreptococcales-Tissierellales	Anaerovoraceae	Family XIII AD3011 group	NA
37	Firmicutes	Clostridia	Lachnospirales	Lachnospiraceae	Fusicatenibacter	saccharivorans
38	Firmicutes	Clostridia	Lachnospirales	Lachnospiraceae	NA	NA
39	Actinobacteriota	Coriobacteriia	Coriobacteriales	Eggerthellaceae	Senegalimassilia	anaerobia
40	Actinobacteriota	Coriobacteriia	Coriobacteriales	Eggerthellaceae	Enterorhabdus	NA
41	Bacteroidota	Bacteroidia	Bacteroidales	Rikenellaceae	Alistipes	inops
42	Firmicutes	Clostridia	Oscillospirales	Ruminococcaceae	NA	NA
43	Firmicutes	Clostridia	Oscillospirales	Butyricicoccaceae	Butyricicoccus	faecihominis
44	Bacteroidota	Bacteroidia	Bacteroidales	Rikenellaceae	Alistipes	obesi
45	Firmicutes	Clostridia	Christensenellales	Christensenellaceae	Christensenellaceae R-7 group	NA
46	Firmicutes	Clostridia	Lachnospirales	Lachnospiraceae	Blautia	massiliensis
47	Bacteroidota	Bacteroidia	Bacteroidales	Tannerellaceae	Parabacteroides	distasonis
48	Firmicutes	Clostridia	Lachnospirales	Lachnospiraceae	Lachnoclostridium	NA
49	Firmicutes	Clostridia	Oscillospirales	Ruminococcaceae	Faecalibacterium	prausnitzii
50	Firmicutes	Clostridia	Peptostreptococcales-Tissierellales	Peptostreptococcaceae	Terrisporobacter	mayombei
51	Desulfobacterota	Desulfovibrionia	Desulfovibrionales	Desulfovibrionaceae	Bilophila	wadsworthia
52	Bacteroidota	Bacteroidia	Bacteroidales	Muribaculaceae	NA	NA
53	Firmicutes	Bacilli	Erysipelotrichales	Erysipelotrichaceae	Turicibacter	sanguinis
54	Firmicutes	Clostridia	Oscillospirales	Oscillospiraceae	Oscillibacter	NA
55	Firmicutes	Clostridia	Clostridia UCG-014	NA	NA	NA
56	Firmicutes	Clostridia	Oscillospirales	Oscillospiraceae	UCG-002	NA
57	Firmicutes	Clostridia	Christensenellales	Christensenellaceae	Christensenellaceae R-7 group	NA
58	Proteobacteria	Gammaproteobacteria	Burkholderiales	Sutterellaceae	Sutterella	NA
59	Firmicutes	Clostridia	Oscillospirales	Butyricicoccaceae	Butyricicoccus	NA
60	Firmicutes	Clostridia	Lachnospirales	Lachnospiraceae	Roseburia	hominis
61	Firmicutes	Bacilli	Lactobacillales	Streptococcaceae	Streptococcus	NA
62	Firmicutes	Clostridia	Clostridia UCG-014	NA	NA	NA
63	Firmicutes	Clostridia	Oscillospirales	Ruminococcaceae	Ruminococcus	NA
64	Bacteroidota	Bacteroidia	Bacteroidales	Prevotellaceae	Prevotella	NA
65	Firmicutes	Clostridia	Oscillospirales	Oscillospiraceae	UCG-005	NA
66	Firmicutes	Clostridia	Lachnospirales	Lachnospiraceae	[Eubacterium] ventriosum group	NA
67	Firmicutes	Clostridia	Peptostreptococcales-Tissierellales	Peptostreptococcaceae	Romboutsia	NA
68	Firmicutes	Clostridia	Lachnospirales	Lachnospiraceae	Roseburia	NA

ASV_nr	DESeq_baseMean	DESeq_log2FoldChange	DESeq_lfcSE	DESeq_stat	DESeq_pvalue	DESeq_padj	ANCOM_W	ANCOM_detected_0.7
33	92,7196387	-0,120331261	0,717025645	-0,167820024	0,866724869	0,995587377	0	FALSE
34	45,81673492	0,407587254	0,755590317	0,5394289	0,589590944	0,995587377	0	FALSE
35	77,61101713	0,20586914	0,554627157	0,371184746	0,710499934	0,995587377	2	FALSE
36	1,166833383	-2,288938472	1,154228213	-1,98309004	0,047357373	0,995587377	94	FALSE
37	952,5518573	-0,624852757	0,245525797	-2,544957653	0,010929099	0,922904169	1	FALSE
38	190,6326441	-0,185250959	0,457694115	-0,404748396	0,685662464	0,995587377	0	FALSE
39	9,57437575	0,288354192	0,60746222	0,474686626	0,635010352	0,995587377	26	FALSE
40	6,270450016	0,802846009	0,621530824	1,291723561	0,196452895	0,995587377	0	FALSE
41	75,67319808	-1,057314274	0,726333086	-1,455687886	0,14547892	0,995587377	0	FALSE
42	94,3891093	0,065098758	0,63973453	0,101759018	0,918947955	0,995587377	0	FALSE
43	42,47080022	-0,872977372	0,6471314	-1,348995539	0,177338399	0,995587377	0	FALSE
44	70,02423746	0,798065508	0,438889071	1,818376352	0,06900663	0,995587377	3	FALSE
45	104,2352511	-0,127666244	0,756080901	-0,168852624	0,865912564	0,995587377	0	FALSE
46	457,1731666	-0,390190647	0,211412504	-1,845636565	0,064944994	0,995587377	22	FALSE
47	60,01617194	0,576639789	0,933422496	0,61776933	0,536727404	0,995587377	0	FALSE
48	90,75857077	-0,824282808	0,500397377	-1,647256452	0,099505345	0,995587377	313	TRUE
49	71,29285387	-0,054677969	1,197654656	-0,045654203	0,963585866	0,995587377	0	FALSE
50	5,215731805	1,066001873	1,526910111	0,698143175	0,48508766	0,995587377	0	FALSE
51	45,53190582	0,563428501	0,463596531	1,215342359	0,224235542	0,995587377	0	FALSE
52	100,6627842	0,625295973	1,270288463	0,492247227	0,622544582	0,995587377	0	FALSE
53	11,07323571	-0,050358706	1,229982207	-0,040942629	0,967341633	0,995587377	3	FALSE
54	42,54904972	-0,208383199	0,449736155	-0,463345445	0,643116776	0,995587377	0	FALSE
55	68,56298878	-0,669177189	0,721941639	-0,926913137	0,353971636	0,995587377	0	FALSE
56	45,54025015	0,372323142	1,02664643	0,36265956	0,716859213	0,995587377	0	FALSE
57	590,1661122	-0,681909555	0,531085	-1,28399325	0,199144317	0,995587377	0	FALSE
58	71,25040622	0,132464047	0,702817971	0,188475611	0,850503832	0,995587377	0	FALSE
59	56,77991786	-0,280061877	0,602216682	-0,465051675	0,641894451	0,995587377	0	FALSE
60	91,16057651	-0,442656255	0,663135197	-0,667520375	0,5044398	0,995587377	0	FALSE
61	38,41985713	-1,955043411	1,073498105	-1,821189438	0,068578065	0,995587377	0	FALSE
62	27,81164986	0,77620889	1,482717904	0,523504092	0,600623504	0,995587377	0	FALSE
63	36,67554873	-0,641530487	1,158712532	-0,553658021	0,57981291	0,995587377	0	FALSE
64	39,88587837	-0,150048229	1,343313499	-0,111700083	0,911061214	0,995587377	0	FALSE
65	69,49027826	-0,34240352	0,428719391	-0,798665811	0,424484216	0,995587377	0	FALSE
66	84,59216607	-0,621475791	0,416417048	-1,492435994	0,135584885	0,995587377	0	FALSE
67	43,67551979	0,72850763	0,570183573	1,277672077	0,201365075	0,995587377	0	FALSE
68	100,2273584	-0,476149115	0,5511589	-0,863905337	0,387639895	0,995587377	274	TRUE

ASV_nr	Phylum	Class	Order	Family	Genus	Species
69	Firmicutes	Clostridia	Oscillospirales	Ruminococcaceae	Ruminococcus	callidus
70	Firmicutes	Clostridia	Oscillospirales	Oscillospiraceae	UCG-002	NA
71	Desulfobacterota	Desulfovibrionia	Desulfovibrionales	Desulfovibrionaceae	Desulfovibrio	NA
72	Bacteroidota	Bacteroidia	Bacteroidales	Tannerellaceae	Parabacteroides	distasonis
73	Firmicutes	Clostridia	Oscillospirales	Oscillospiraceae	Intestinimonas	NA
74	Firmicutes	Clostridia	Lachnospirales	Lachnospiraceae	Lachnoclostridium	edouardi
75	Actinobacteriota	Coriobacteriia	Coriobacteriales	Coriobacteriaceae	Collinsella	aerofaciens
76	Bacteroidota	Bacteroidia	Bacteroidales	Barnesiellaceae	Barnesiella	NA
77	Firmicutes	Clostridia	Oscillospirales	Oscillospiraceae	NA	NA
78	Proteobacteria	Gammaproteobacteria	Pasteurellales	Pasteurellaceae	Haemophilus	NA
79	Actinobacteriota	Coriobacteriia	Coriobacteriales	Coriobacteriales Incertae Sedis	NA	NA
80	Firmicutes	Bacilli	Izemoplasmatales	NA	NA	NA
81	Bacteroidota	Bacteroidia	Bacteroidales	Prevotellaceae	Paraprevotella	NA
82	Firmicutes	Clostridia	Lachnospirales	Lachnospiraceae	Dorea	longicatena
83	Firmicutes	Clostridia	Clostridia vadinBB60 group	NA	NA	NA
84	Firmicutes	Clostridia	Oscillospirales	Ruminococcaceae	Ruminococcus	NA
85	Firmicutes	Negativicutes	Acidaminococcales	Acidaminococcaceae	Phascolarctobacterium	NA
86	Firmicutes	Clostridia	Oscillospirales	Ruminococcaceae	Ruminococcus	NA
87	Firmicutes	Clostridia	Clostridia UCG-014	NA	NA	NA
88	Firmicutes	Clostridia	Lachnospirales	Lachnospiraceae	[Ruminococcus] torques group	NA
89	Firmicutes	Clostridia	Clostridia UCG-014	NA	NA	NA
90	Firmicutes	Bacilli	Erysipelotrichales	Erysipelatoclostridiaceae	Erysipelotrichaceae UCG-003	bacterium
91	Firmicutes	Clostridia	Oscillospirales	Ruminococcaceae	Ruminococcus	champanellensis
92	Firmicutes	Clostridia	Oscillospirales	Ruminococcaceae	UBA1819	NA
93	Firmicutes	Clostridia	Lachnospirales	Lachnospiraceae	Lachnospiraceae NK4A136 group	NA
94	Bacteroidota	Bacteroidia	Bacteroidales	Marinifilaceae	Odoribacter	splanchnicus
95	Actinobacteriota	Coriobacteriia	Coriobacteriales	Eggerthellaceae	Adlercreutzia	equolifaciens
96	Bacteroidota	Bacteroidia	Bacteroidales	Bacteroidaceae	Bacteroides	intestinalis
97	Bacteroidota	Bacteroidia	Bacteroidales	Barnesiellaceae	Barnesiella	intestinihominis
98	Firmicutes	Clostridia	Oscillospirales	Ruminococcaceae	Ruminococcus	NA
99	Bacteroidota	Bacteroidia	Bacteroidales	Bacteroidaceae	Bacteroides	vulgatus
100	Bacteroidota	Bacteroidia	Bacteroidales	Prevotellaceae	Prevotella	copri
101	Firmicutes	Clostridia	Lachnospirales	Lachnospiraceae	[Eubacterium] xylanophilum group	NA
102	Firmicutes	Clostridia	Oscillospirales	Oscillospiraceae	UCG-002	NA
103	Firmicutes	Clostridia	Lachnospirales	Lachnospiraceae	[Eubacterium] hallii group	NA
104	Firmicutes	Clostridia	Clostridia UCG-014	NA	NA	NA

ASV_nr	DESeq_baseMean	DESeq_log2FoldChange	DESeq_lfcSE	DESeq_stat	DESeq_pvalue	DESeq_padj	ANCOM_W	ANCOM_detected_0.7
69	69,77298672	0,623976519	1,21112462	0,515204223	0,606410318	0,995587377	2	FALSE
70	31,42207306	-0,734663759	0,922223667	-0,796622105	0,425670535	0,995587377	0	FALSE
71	122,0218519	0,003461701	0,816617035	0,004239076	0,996617717	0,997588574	0	FALSE
72	33,33970451	1,050052186	1,382852834	0,759337624	0,447650616	0,995587377	3	FALSE
73	23,37189412	0,566637818	0,659309239	0,859441647	0,390096901	0,995587377	0	FALSE
74	83,59874925	-0,478847546	0,452266909	-1,058772013	0,289703622	0,995587377	0	FALSE
75	168,6728178	-0,147756233	0,267197073	-0,552985972	0,580273016	0,995587377	0	FALSE
76	55,52765658	-1,254091924	1,517195028	-0,826585838	0,408471847	0,995587377	0	FALSE
77	19,19788549	-0,222751706	0,84641817	-0,263169807	0,792419701	0,995587377	0	FALSE
78	18,43768999	-0,459462967	0,693676779	-0,66235887	0,507741257	0,995587377	0	FALSE
79	11,37717093	0,364472886	1,222499647	0,298137416	0,765598285	0,995587377	0	FALSE
80	19,09607204	0,757768643	1,174924349	0,644951008	0,518958935	0,995587377	0	FALSE
81	41,79771207	-0,126726005	1,139686679	-0,111193723	0,911462729	0,995587377	0	FALSE
82	771,1811403	-0,167849296	0,24795981	-0,676921376	0,498455841	0,995587377	0	FALSE
83	26,31798578	-0,050314147	1,337599968	-0,037615242	0,969994455	0,995587377	1	FALSE
84	97,16806451	-1,343272334	1,159718512	-1,158274461	0,24675205	0,995587377	0	FALSE
85	63,11286342	1,22233093	1,630886402	0,749488701	0,453562707	0,995587377	0	FALSE
86	60,35144369	0,221751692	1,493479501	0,148479903	0,881964045	0,995587377	0	FALSE
87	62,3951171	0,092832163	1,340889827	0,06923176	0,944805143	0,995587377	0	FALSE
88	51,66910783	-0,783742515	1,290403029	-0,607362581	0,543610315	0,995587377	0	FALSE
89	38,10297231	-1,256241684	1,594968338	-0,787627976	0,430914346	0,995587377	0	FALSE
90	154,8434986	0,008500811	0,379369129	0,022407757	0,982122693	0,997588574	0	FALSE
91	93,17626289	-0,070974697	1,754183485	-0,040460247	0,967726199	0,995587377	0	FALSE
92	28,01369877	1,868015181	0,738523329	2,529392245	0,011426025	0,922904169	166	FALSE
93	20,83317053	-0,297176619	1,326572896	-0,224018311	0,822743056	0,995587377	0	FALSE
94	29,27798241	0,327488408	0,280824274	1,166168449	0,243546346	0,995587377	5	FALSE
95	10,08643593	-0,88504256	0,672490602	-1,316066808	0,18815162	0,995587377	0	FALSE
96	27,52574542	2,339596281	1,709748616	1,368386124	0,171191245	0,995587377	1	FALSE
97	46,478684	-1,614344668	1,243655965	-1,298063704	0,194265445	0,995587377	0	FALSE
98	69,12593766	-0,100338899	1,41694609	-0,070813491	0,943546195	0,995587377	0	FALSE
99	1159,173926	0,026551402	0,353187858	0,07517643	0,940074338	0,995587377	0	FALSE
100	534,5951297	0,466197429	1,06123768	0,439295964	0,660447098	0,995587377	0	FALSE
101	42,97739151	-0,37544831	0,527828588	-0,711307266	0,47689385	0,995587377	0	FALSE
102	21,28121679	-3,106443801	1,384232954	-2,244162582	0,024821943	0,995587377	0	FALSE
103	11,52602578	-1,232782618	1,05707899	-1,166216176	0,243527054	0,995587377	0	FALSE
104	55,74819568	0,767668367	1,026940531	0,747529525	0,454743987	0,995587377	0	FALSE

ASV_nr	Phylum	Class	Order	Family	Genus	Species
105	Firmicutes	Bacilli	RF39	NA	NA	NA
106	Firmicutes	Clostridia	Oscillospirales	Oscillospiraceae	Intestinimonas	NA
107	Firmicutes	Clostridia	Lachnospirales	Lachnospiraceae	Blautia	NA
108	Firmicutes	Clostridia	Oscillospirales	Ruminococcaceae	Faecalibacterium	prausnitzii
109	Bacteroidota	Bacteroidia	Bacteroidales	Tannerellaceae	NA	NA
110	Firmicutes	Clostridia	Lachnospirales	Lachnospiraceae	Lachnospiraceae FCS020 group	NA
111	Firmicutes	Clostridia	Clostridia UCG-014	NA	NA	NA
112	Firmicutes	Clostridia	Lachnospirales	Lachnospiraceae	Lachnospiraceae UCG-010	NA
113	Firmicutes	Clostridia	Oscillospirales	Oscillospiraceae	UCG-002	NA
114	Firmicutes	Clostridia	Lachnospirales	Lachnospiraceae	Lachnospira	pectinoschiza
115	Firmicutes	Clostridia	Peptococcales	Peptococcaceae	Peptococcus	NA
116	Bacteroidota	Bacteroidia	Bacteroidales	Bacteroidaceae	Bacteroides	uniformis
117	Firmicutes	Clostridia	Peptostreptococcales-Tissierellales	Anaerovoracaceae	Family XIII AD3011 group	NA
118	Firmicutes	Clostridia	Lachnospirales	Lachnospiraceae	Lachnospiraceae UCG-004	NA
119	Firmicutes	Clostridia	Oscillospirales	Oscillospiraceae	Intestinimonas	NA
120	Firmicutes	Clostridia	Oscillospirales	Oscillospiraceae	NK4A214 group	NA
121	Bacteroidota	Bacteroidia	Bacteroidales	Barnesiellaceae	Barnesiella	NA
122	Firmicutes	Clostridia	Lachnospirales	Lachnospiraceae	Lachnospiraceae UCG-001	NA
123	Firmicutes	Bacilli	Lactobacillales	Streptococcaceae	Streptococcus	NA
124	Firmicutes	Clostridia	Oscillospirales	Oscillospiraceae	Flavonifractor	plautii
125	Firmicutes	Clostridia	Oscillospirales	Ruminococcaceae	[Eubacterium] siraeum group	NA
126	Proteobacteria	Alphaproteobacteria	Rhodospirillales	NA	NA	NA
127	Firmicutes	Bacilli	Acholeplasmatales	Acholeplasmataceae	Anaeroplasma	NA
128	Firmicutes	Clostridia	Oscillospirales	[Eubacterium] coprostanoligenes group	NA	NA
129	Cyanobacteria	Vampirivibrionia	Gastranaerophilales	NA	NA	NA
130	Bacteroidota	Bacteroidia	Bacteroidales	Bacteroidaceae	Bacteroides	uniformis
131	Bacteroidota	Bacteroidia	Bacteroidales	Bacteroidaceae	Bacteroides	clarus
132	Firmicutes	Clostridia	Oscillospirales	Butyricicoccaceae	NA	NA
133	Bacteroidota	Bacteroidia	Bacteroidales	Prevotellaceae	Alloprevotella	NA
134	Firmicutes	Clostridia	Oscillospirales	[Eubacterium] coprostanoligenes group	NA	NA
135	Firmicutes	Clostridia	Lachnospirales	Lachnospiraceae	Tyzzerella	NA
136	Firmicutes	Clostridia	Clostridia UCG-014	NA	NA	NA
137	Actinobacteriota	Actinobacteria	Bifidobacteriales	Bifidobacteriaceae	Bifidobacterium	bifidum
138	Actinobacteriota	Actinobacteria	Bifidobacteriales	Bifidobacteriaceae	Bifidobacterium	NA
139	Euryarchaeota	Methanobacteria	Methanobacteriales	Methanobacteriaceae	Methanosphaera	NA
140	Firmicutes	Clostridia	Oscillospirales	Ruminococcaceae	Subdoligranulum	NA

ASV_nr	DESeq_baseMean	DESeq_log2FoldChange	DESeq_lfcSE	DESeq_stat	DESeq_pvalue	DESeq_padj	ANCOM_W	ANCOM_detected_0.7
105	29,18732529	1,526325634	1,302812725	1,171561809	0,241373029	0,995587377	0	FALSE
106	17,57655195	0,197429987	0,997574371	0,197910044	0,843115446	0,995587377	0	FALSE
107	65,58925749	-0,039320396	0,992634855	-0,039612146	0,968402344	0,995587377	0	FALSE
108	413,3141041	-0,10967091	0,358719569	-0,305728819	0,759811129	0,995587377	0	FALSE
109	41,84025428	1,45826674	1,051117965	1,387348317	0,165335576	0,995587377	1	FALSE
110	68,22914824	-0,489696376	0,434438669	-1,127193343	0,259660745	0,995587377	0	FALSE
111	20,53972409	1,230644611	1,158556956	1,062221934	0,288134945	0,995587377	0	FALSE
112	49,23274842	-0,036284397	0,721443536	-0,050294161	0,959887977	0,995587377	0	FALSE
113	76,03490419	-0,551570404	1,413329081	-0,390263253	0,696341893	0,995587377	0	FALSE
114	34,10394599	-0,75547478	0,666658564	-1,133225942	0,257119383	0,995587377	63	FALSE
115	22,94188086	0,05554682	1,427642145	0,038908084	0,968963671	0,995587377	0	FALSE
116	410,0822985	-0,059596176	0,511933562	-0,116413886	0,90732453	0,995587377	0	FALSE
117	13,60256485	-0,233822972	0,559297877	-0,418065188	0,675899457	0,995587377	0	FALSE
118	39,00889727	-0,325696846	0,571402306	-0,569995681	0,568680628	0,995587377	0	FALSE
119	37,18763124	0,013442814	0,395435963	0,03399492	0,972881202	0,995587377	0	FALSE
120	37,17778039	0,583963113	0,573906652	1,017522815	0,308904784	0,995587377	46	FALSE
121	53,76152698	1,359161181	1,73992293	0,781161716	0,434707387	0,995587377	0	FALSE
122	54,95023686	0,122038336	0,679659775	0,17955798	0,857499596	0,995587377	0	FALSE
123	238,5386035	-0,408399621	0,387132649	-1,054934586	0,291455243	0,995587377	0	FALSE
124	10,45997127	1,096697601	0,900832481	1,217426796	0,223441873	0,995587377	1	FALSE
125	34,49299911	0,543165869	0,866690876	0,626712342	0,530847812	0,995587377	0	FALSE
126	18,92975505	3,022696241	1,421940539	2,125754318	0,033523722	0,995587377	3	FALSE
127	24,89543894	-1,020990348	1,349221632	-0,756725451	0,449214364	0,995587377	0	FALSE
128	16,59329061	-0,395048893	1,510106151	-0,261603393	0,793627228	0,995587377	0	FALSE
129	16,5720775	1,810041502	0,911602724	1,985559559	0,047082251	0,995587377	2	FALSE
130	399,8414988	-0,166685925	0,496962042	-0,335409772	0,737315997	0,995587377	0	FALSE
131	20,64325086	0,087959725	1,435578503	0,061271275	0,951143167	0,995587377	0	FALSE
132	14,48493382	1,719884569	1,004335648	1,712459946	0,08681194	0,995587377	1	FALSE
133	70,06662897	2,446310766	1,859832205	1,315339502	0,188395828	0,995587377	0	FALSE
134	6,333431953	0,227795892	1,324580627	0,17197586	0,863456507	0,995587377	0	FALSE
135	48,10614409	-0,107889021	1,069850125	-0,100844986	0,919673515	0,995587377	0	FALSE
136	15,41770002	1,381458032	1,528429164	0,903841712	0,36607934	0,995587377	2	FALSE
137	15,39733655	-0,508372844	1,627744791	-0,312317291	0,754799401	0,995587377	0	FALSE
138	328,2141787	-0,611037086	0,479213059	-1,275084379	0,202279378	0,995587377	0	FALSE
139	4,085745334	0,0042875	1,418631419	0,003022279	0,997588574	0,997588574	0	FALSE
140	10,01473561	-0,849666338	1,365982728	-0,622018361	0,533929795	0,995587377	0	FALSE

ASV_nr	Phylum	Class	Order	Family	Genus	Species
141	Proteobacteria	Alphaproteobacteria	Rhodospirillales	NA	NA	NA
142	Bacteroidota	Bacteroidia	Bacteroidales	Prevotellaceae	Paraprevotella	NA
143	Firmicutes	Clostridia	Oscillospirales	Oscillospiraceae	NK4A214 group	NA
144	Firmicutes	Bacilli	Erysipelotrichales	Erysipelotrichaceae	Solobacterium	NA
145	Bacteroidota	Bacteroidia	Bacteroidales	Tannerellaceae	Parabacteroides	distasonis
146	Firmicutes	Clostridia	Lachnospirales	Lachnospiraceae	Lachnospira	NA
147	Actinobacteriota	Coriobacteriia	Coriobacteriales	Coriobacteriales Incertae Sedis	NA	NA
148	Firmicutes	Clostridia	Lachnospirales	Lachnospiraceae	Blautia	NA
149	Euryarchaeota	Methanobacteria	Methanobacteriales	Methanobacteriaceae	Methanobrevibacter	NA
150	Firmicutes	Clostridia	Lachnospirales	Lachnospiraceae	Lachnospiraceae AC2044 group	NA
151	Firmicutes	Clostridia	Peptostreptococcales-Tissierellales	Anaerovoracaceae	Family XIII UCG-001	NA
152	Firmicutes	Clostridia	Oscillospirales	Ruminococcaceae	Incertae Sedis	NA
153	Firmicutes	Clostridia	Oscillospirales	Oscillospiraceae	Colidextribacter	NA
154	Firmicutes	Clostridia	Oscillospirales	Ruminococcaceae	CAG-352	NA
155	Firmicutes	Clostridia	Lachnospirales	Lachnospiraceae	Lachnoclostridium	NA
156	Firmicutes	Clostridia	Oscillospirales	Ruminococcaceae	Incertae Sedis	NA
157	Firmicutes	Clostridia	Christensenellales	Christensenellaceae	Christensenellaceae R-7 group	NA
158	Firmicutes	Clostridia	Oscillospirales	Oscillospiraceae	NK4A214 group	NA
159	Firmicutes	Bacilli	Erysipelotrichales	Erysipelotrichaceae	Catenisphaera	NA
160	Firmicutes	Clostridia	Clostridiales	Clostridiaceae	Clostridium sensu stricto 1	NA
161	Bacteroidota	Bacteroidia	Bacteroidales	Rikenellaceae	Alistipes	ihumii
162	Actinobacteriota	Coriobacteriia	Coriobacteriales	Eggerthellaceae	Slackia	isoflavoniconvertens
163	Firmicutes	Clostridia	Oscillospirales	Oscillospiraceae	UCG-002	NA
164	Firmicutes	Clostridia	Lachnospirales	Lachnospiraceae	NA	NA
165	Firmicutes	Clostridia	Christensenellales	Christensenellaceae	Christensenellaceae R-7 group	NA
166	Firmicutes	Bacilli	Erysipelotrichales	Erysipelotrichaceae	NA	NA
167	Firmicutes	Bacilli	Erysipelotrichales	Erysipelatoclostridiaceae	Erysipelatoclostridium	NA
168	Firmicutes	Clostridia	Oscillospirales	[Eubacterium] coprostanoligenes group	NA	NA
169	Firmicutes	Clostridia	Peptostreptococcales-Tissierellales	Anaerovoracaceae	Mogibacterium	NA
170	Firmicutes	Clostridia	Oscillospirales	Ruminococcaceae	Subdoligranulum	NA
171	Proteobacteria	Gammaproteobacteria	Enterobacterales	Enterobacteriaceae	Escherichia-Shigella	NA
172	Firmicutes	Clostridia	Oscillospirales	Ruminococcaceae	Negativibacillus	NA
173	Proteobacteria	Gammaproteobacteria	Burkholderiales	Sutterellaceae	Sutterella	NA
174	Firmicutes	Bacilli	Lactobacillales	Streptococcaceae	Lactococcus	NA
175	Firmicutes	Clostridia	Lachnospirales	Lachnospiraceae	GCA-900066575	NA
176	Bacteroidota	Bacteroidia	Bacteroidales	Rikenellaceae	Alistipes	putredinis

ASV_nr	DESeq_baseMean	DESeq_log2FoldChange	DESeq_lfcSE	DESeq_stat	DESeq_pvalue	DESeq_padj	ANCOM_W	ANCOM_detected_0.7
141	14,24676565	1,239269167	1,372459099	0,902955263	0,366549639	0,995587377	0	FALSE
142	27,93850196	1,425485292	1,479520171	0,963478106	0,335307646	0,995587377	0	FALSE
143	21,94688004	-0,444160533	0,888830308	-0,499713533	0,617276802	0,995587377	0	FALSE
144	12,68808531	0,434913576	1,729563757	0,25145854	0,801459613	0,995587377	0	FALSE
145	34,22292334	-0,530097253	1,056164935	-0,501907643	0,615732489	0,995587377	0	FALSE
146	25,78258968	0,555771568	0,935570091	0,594045891	0,55248142	0,995587377	0	FALSE
147	3,935163854	-0,446941264	1,201811792	-0,371889564	0,709975075	0,995587377	0	FALSE
148	5,522251047	1,054339098	1,212849585	0,869307382	0,384679026	0,995587377	2	FALSE
149	106,4440215	0,396471229	0,487869309	0,812658681	0,416413778	0,995587377	0	FALSE
150	42,90995495	1,316820506	0,942449945	1,397231241	0,162344045	0,995587377	0	FALSE
151	9,085559122	-0,66098498	0,816734899	-0,809301746	0,418341602	0,995587377	0	FALSE
152	8,021608344	0,568409473	1,089261095	0,521830327	0,601788472	0,995587377	0	FALSE
153	17,68731727	-0,007003197	0,548226853	-0,012774269	0,989807886	0,997588574	0	FALSE
154	387,0932137	-0,953890755	1,127268692	-0,846196441	0,397443167	0,995587377	0	FALSE
155	34,49852299	-0,553568724	0,494092714	-1,120374189	0,262554339	0,995587377	63	FALSE
156	10,96539543	0,07669173	0,751733484	0,10201984	0,918740927	0,995587377	0	FALSE
157	8,411965432	1,19247887	0,993382223	1,200423002	0,2299751	0,995587377	6	FALSE
158	42,09229831	0,150278699	1,004803221	0,149560328	0,88111151	0,995587377	0	FALSE
159	6,719500752	0,953697683	1,515769893	0,629183682	0,52922881	0,995587377	0	FALSE
160	53,62553458	-0,286725139	0,683010388	-0,419796162	0,674634369	0,995587377	0	FALSE
161	23,15314743	0,17503107	0,70881901	0,246933375	0,804959787	0,995587377	0	FALSE
162	10,32096601	-0,601664925	0,601371919	-1,000487229	0,317074775	0,995587377	0	FALSE
163	569,0382904	-0,116738338	0,455616042	-0,256220869	0,797780295	0,995587377	0	FALSE
164	36,39353214	0,158646481	1,244883724	0,127438794	0,898593113	0,995587377	0	FALSE
165	28,77733534	-0,31553878	0,763498564	-0,413280123	0,679401392	0,995587377	0	FALSE
166	12,64937255	0,882747081	0,817147073	1,080279316	0,280017818	0,995587377	0	FALSE
167	4,4053988	0,875466784	1,013542417	0,863769261	0,387714657	0,995587377	0	FALSE
168	30,25895759	1,977703217	1,642370889	1,204175762	0,228521652	0,995587377	0	FALSE
169	18,15282111	0,315345669	1,459947432	0,215997962	0,828989336	0,995587377	0	FALSE
170	621,5564743	-0,255872289	0,283307971	-0,903159514	0,366441242	0,995587377	0	FALSE
171	651,1248701	-0,179911148	0,623115295	-0,288728505	0,772789147	0,995587377	0	FALSE
172	18,34620079	-0,263711405	0,750150632	-0,351544601	0,725179818	0,995587377	0	FALSE
173	16,69650119	1,536370655	1,24112358	1,237886928	0,21575799	0,995587377	0	FALSE
174	5,710673708	1,306386583	1,451936293	0,899754755	0,368250777	0,995587377	3	FALSE
175	17,16370681	-0,228686337	0,577368291	-0,396083991	0,692043068	0,995587377	0	FALSE
176	363,5940653	-0,153354265	0,392931065	-0,390282874	0,696327386	0,995587377	0	FALSE

ASV_nr	Phylum	Class	Order	Family	Genus	Species
177	Firmicutes	Clostridia	Lachnospirales	Lachnospiraceae	Lachnospiraceae UCG-004	NA
178	Firmicutes	Negativicutes	Veillonellales-Selenomonadales	Veillonellaceae	Veillonella	NA
179	Firmicutes	Clostridia	Lachnospirales	Lachnospiraceae	Lachnoclostridium	NA
180	Firmicutes	Clostridia	Oscillospirales	Oscillospiraceae	Colidextribacter	massiliensis
181	Firmicutes	Clostridia	Lachnospirales	Lachnospiraceae	NA	NA
182	Firmicutes	Clostridia	Oscillospirales	Oscillospiraceae	UCG-002	NA
183	Firmicutes	Clostridia	Oscillospirales	Ruminococcaceae	Subdoligranulum	NA
184	Firmicutes	Clostridia	Peptostreptococcales-Tissierellales	Anaerovoracaceae	Family XIII UCG-001	NA
185	Firmicutes	Clostridia	Clostridia vadinBB60 group	NA	NA	NA
186	Firmicutes	Clostridia	Christensenellales	Christensenellaceae	Christensenellaceae R-7 group	NA
187	Firmicutes	Clostridia	Peptostreptococcales-Tissierellales	Anaerovoracaceae	Family XIII AD3011 group	NA
188	Firmicutes	Clostridia	Peptostreptococcales-Tissierellales	Peptostreptococcaceae	Intestinibacter	bartlettii
189	Proteobacteria	Alphaproteobacteria	Rhodospirillales	NA	NA	NA
190	Proteobacteria	Alphaproteobacteria	Rhodospirillales	NA	NA	NA
191	Firmicutes	Clostridia	Peptostreptococcales-Tissierellales	Anaerovoracaceae	[Eubacterium] brachy group	NA
192	Firmicutes	Clostridia	Lachnospirales	Lachnospiraceae	NA	NA
193	Firmicutes	Clostridia	Lachnospirales	Lachnospiraceae	[Eubacterium] ventriosum group	NA
194	Firmicutes	Clostridia	Lachnospirales	Lachnospiraceae	Anaerostipes	hadrus
195	Bacteroidota	Bacteroidia	Bacteroidales	Barnesiellaceae	NA	NA
196	Firmicutes	Clostridia	Lachnospirales	Lachnospiraceae	Lachnoclostridium	NA
197	Firmicutes	Clostridia	Lachnospirales	Lachnospiraceae	Lachnotalea	NA
198	Firmicutes	Clostridia	Monoglobales	Monoglobaceae	Monoglobus	pectinilyticus
199	Firmicutes	Clostridia	Christensenellales	Christensenellaceae	Christensenellaceae R-7 group	NA
200	Firmicutes	Clostridia	Lachnospirales	Lachnospiraceae	Agathobacter	NA
201	Firmicutes	Clostridia	Lachnospirales	Lachnospiraceae	NA	NA
202	Firmicutes	Clostridia	Lachnospirales	Lachnospiraceae	Roseburia	NA
203	Firmicutes	Clostridia	Lachnospirales	Lachnospiraceae	Blautia	obeum
204	Firmicutes	Clostridia	Lachnospirales	Lachnospiraceae	[Eubacterium] xylanophilum group	NA
205	Firmicutes	Clostridia	Oscillospirales	Ruminococcaceae	Negativibacillus	NA
206	Firmicutes	Clostridia	Lachnospirales	Lachnospiraceae	Marvinbryantia	NA
207	Firmicutes	Clostridia	Lachnospirales	Lachnospiraceae	[Eubacterium] hallii group	NA
208	Firmicutes	Clostridia	Oscillospirales	Ruminococcaceae	NA	NA
209	Bacteroidota	Bacteroidia	Bacteroidales	Rikenellaceae	Alistipes	NA
210	Bacteroidota	Bacteroidia	Bacteroidales	Rikenellaceae	Alistipes	NA
211	Firmicutes	Clostridia	Oscillospirales	UCG-010	NA	NA
212	Firmicutes	Clostridia	Clostridiales	Clostridiaceae	Clostridium sensu stricto 1	NA

ASV_nr	DESeq_baseMean	DESeq_log2FoldChange	DESeq_lfcSE	DESeq_stat	DESeq_pvalue	DESeq_padj	ANCOM_W	ANCOM_detected_0.7
177	19,88185595	-0,085668921	0,782135828	-0,109532025	0,912780522	0,995587377	0	FALSE
178	24,14670033	-0,11719866	0,91516186	-0,128063313	0,898098869	0,995587377	185	FALSE
179	7,130968021	0,532095494	1,41666339	0,375597688	0,70721601	0,995587377	0	FALSE
180	36,81646243	-0,401037562	0,560647699	-0,715311171	0,474416787	0,995587377	0	FALSE
181	30,26772699	0,413130404	1,268218432	0,325756505	0,744608589	0,995587377	0	FALSE
182	669,3896048	-0,17594595	0,297561385	-0,591292955	0,554324149	0,995587377	0	FALSE
183	17,43318742	0,120485702	1,126905117	0,106917344	0,914854554	0,995587377	0	FALSE
184	16,77677315	-0,174028404	0,752615054	-0,231231628	0,81713486	0,995587377	0	FALSE
185	6,706379686	0,774910555	1,223147772	0,633537969	0,526382394	0,995587377	0	FALSE
186	22,20284028	-0,060979788	0,674715644	-0,090378501	0,927986441	0,995587377	0	FALSE
187	20,35237569	-0,059347082	0,493497918	-0,120258018	0,904278759	0,995587377	0	FALSE
188	34,01762697	0,519566118	0,692592596	0,750175675	0,453148907	0,995587377	0	FALSE
189	15,7021807	2,817699103	1,126890469	2,500419677	0,012404626	0,922904169	1	FALSE
190	9,019132461	0,287536818	0,983667103	0,292311106	0,770048768	0,995587377	0	FALSE
191	1,374418257	0,976921515	1,230248676	0,79408459	0,427146188	0,995587377	0	FALSE
192	3,878543021	1,320736415	1,284073434	1,028552091	0,303690201	0,995587377	0	FALSE
193	19,58410926	-0,032056872	1,104865544	-0,029014275	0,976853206	0,995587377	0	FALSE
194	147,4634572	-0,105163191	0,281570018	-0,373488598	0,70878483	0,995587377	0	FALSE
195	16,60318873	-0,252728069	0,87079075	-0,290228243	0,771641631	0,995587377	0	FALSE
196	29,81594723	-1,17208782	0,587500567	-1,995041173	0,046038389	0,995587377	0	FALSE
197	6,379706025	0,187150441	0,876276642	0,213574609	0,830878802	0,995587377	0	FALSE
198	7,278113555	0,281362676	1,436354486	0,195886655	0,844698883	0,995587377	0	FALSE
199	6,963252022	1,698214914	0,949537498	1,78846535	0,073700961	0,995587377	0	FALSE
200	586,4857054	-0,574753176	0,595442002	-0,965254675	0,334417268	0,995587377	0	FALSE
201	16,07428236	-1,162717614	1,165041742	-0,998005112	0,31827688	0,995587377	0	FALSE
202	30,72011155	0,418744432	0,930520237	0,450011096	0,65270244	0,995587377	0	FALSE
203	155,8576506	0,506825786	0,372240521	1,361554581	0,173338497	0,995587377	111	FALSE
204	9,193253946	-0,407785881	1,100536296	-0,370533787	0,710984807	0,995587377	0	FALSE
205	17,3986809	-1,987594819	0,882140125	-2,253150902	0,024249636	0,995587377	0	FALSE
206	26,44873928	0,050847987	0,692844025	0,073390237	0,941495587	0,995587377	0	FALSE
207	138,6430315	-0,079589955	0,281436919	-0,28279856	0,777331259	0,995587377	0	FALSE
208	18,80023712	0,044956938	0,443249475	0,101425811	0,919212448	0,995587377	0	FALSE
209	15,6044	-0,28997419	0,904177856	-0,320704813	0,748434099	0,995587377	0	FALSE
210	12,43552507	-0,830558944	0,982945734	-0,844969275	0,398127994	0,995587377	0	FALSE
211	13,47717114	-0,555191757	0,74878956	-0,741452321	0,458419231	0,995587377	0	FALSE
212	17,10468757	-0,528171136	0,958855089	-0,550835202	0,58174665	0,995587377	0	FALSE

ASV_nr	Phylum	Class	Order	Family	Genus	Species
213	Bacteroidota	Bacteroidia	Bacteroidales	Barnesiellaceae	Coprobacter	fastidiosus
214	Firmicutes	Bacilli	RF39	NA	NA	NA
215	Firmicutes	Clostridia	Christensenellales	Christensenellaceae	Christensenellaceae R-7 group	NA
216	Bacteroidota	Bacteroidia	Bacteroidales	Tannerellaceae	Parabacteroides	merdae
217	Firmicutes	Bacilli	RF39	NA	NA	NA
218	Firmicutes	Clostridia	Clostridia UCG-014	NA	NA	NA
219	Firmicutes	Clostridia	Oscillospirales	Ruminococcaceae	DTU089	NA
220	Firmicutes	Clostridia	Lachnospirales	Lachnospiraceae	NA	NA
221	Firmicutes	Clostridia	Oscillospirales	Ruminococcaceae	Ruminococcus	bromii
222	Firmicutes	Bacilli	Erysipelotrichales	Erysipelotrichaceae	Holdemanella	NA
223	Bacteroidota	Bacteroidia	Bacteroidales	Rikenellaceae	Alistipes	indistinctus
224	Firmicutes	Clostridia	Oscillospirales	Ruminococcaceae	Ruminococcus	bicirculans
225	Bacteroidota	Bacteroidia	Bacteroidales	Rikenellaceae	Rikenellaceae RC9 gut group	NA
226	Firmicutes	Clostridia	Oscillospirales	Oscillospiraceae	Colidextribacter	NA
227	Firmicutes	Clostridia	NA	NA	NA	NA
228	Firmicutes	Clostridia	Oscillospirales	Oscillospiraceae	Oscillibacter	NA
229	Firmicutes	Clostridia	Oscillospirales	Oscillospiraceae	UCG-003	NA
230	Firmicutes	Clostridia	Oscillospirales	Oscillospiraceae	Oscillospira	NA
231	Firmicutes	Clostridia	Lachnospirales	Lachnospiraceae	Blautia	faecis
232	Firmicutes	Clostridia	Oscillospirales	UCG-010	NA	NA
233	Firmicutes	Bacilli	Lactobacillales	Lactobacillaceae	Lactobacillus	NA
234	Firmicutes	Clostridia	Christensenellales	Christensenellaceae	Christensenellaceae R-7 group	NA
235	Bacteroidota	Bacteroidia	Bacteroidales	Bacteroidaceae	Bacteroides	NA
236	Firmicutes	Clostridia	Oscillospirales	Oscillospiraceae	NK4A214 group	NA
237	Bacteroidota	Bacteroidia	Bacteroidales	Marinifilaceae	Butyricimonas	virosa
238	Firmicutes	Clostridia	Lachnospirales	Lachnospiraceae	Coprococcus	comes
239	Firmicutes	Clostridia	Oscillospirales	UCG-010	NA	NA
240	Firmicutes	Clostridia	Christensenellales	Christensenellaceae	Christensenellaceae R-7 group	NA
241	Firmicutes	Clostridia	Christensenellales	Christensenellaceae	Christensenellaceae R-7 group	NA
242	Firmicutes	Clostridia	Christensenellales	Christensenellaceae	Christensenellaceae R-7 group	NA
243	Firmicutes	Clostridia	Oscillospirales	UCG-010	NA	NA
244	Firmicutes	Clostridia	Oscillospirales	[Eubacterium] coprostanoligenes group	NA	NA
245	Bacteroidota	Bacteroidia	Bacteroidales	Bacteroidaceae	Bacteroides	NA
246	Firmicutes	Negativicutes	Veillonellales-Selenomonadales	Veillonellaceae	Veillonella	NA
247	Firmicutes	Bacilli	Erysipelotrichales	Erysipelatoclostridiaceae	Coprobacillus	cateniformis
248	Firmicutes	Clostridia	Oscillospirales	Oscillospiraceae	UCG-005	NA

ASV_nr	DESeq_baseMean	DESeq_log2FoldChange	DESeq_lfcSE	DESeq_stat	DESeq_pvalue	DESeq_padj	ANCOM_W	ANCOM_detected_0.7
213	11,16238561	0,057337898	0,636590571	0,090070291	0,928231357	0,995587377	0	FALSE
214	12,93319179	-0,3062384	1,460688474	-0,209653465	0,833938148	0,995587377	0	FALSE
215	2,073926191	0,364968144	1,055745026	0,345697242	0,729570257	0,995587377	6	FALSE
216	267,8088584	0,158198695	0,396684425	0,398802386	0,690038819	0,995587377	0	FALSE
217	12,79003659	-0,971397764	1,4066744	-0,690563334	0,489839996	0,995587377	0	FALSE
218	15,45310902	0,444028615	1,632659641	0,271966431	0,785647831	0,995587377	0	FALSE
219	9,09682749	0,058976364	0,647041036	0,091147795	0,927375155	0,995587377	0	FALSE
220	28,85939818	-0,238551867	0,718828445	-0,33186203	0,739993443	0,995587377	0	FALSE
221	734,0694008	-0,223332476	0,620854926	-0,359717651	0,719058291	0,995587377	0	FALSE
222	166,4376066	0,883543874	0,898139043	0,983749544	0,325238673	0,995587377	5	FALSE
223	10,9313602	0,306694432	0,778802679	0,393802488	0,693726868	0,995587377	0	FALSE
224	158,4313979	-0,04960389	0,509691978	-0,097321309	0,922471234	0,995587377	0	FALSE
225	12,66852999	1,572885925	1,380140224	1,139656607	0,254429392	0,995587377	0	FALSE
226	10,45351636	0,244777268	0,60251482	0,406259333	0,684552063	0,995587377	0	FALSE
227	9,547282327	-0,311731192	0,436064553	-0,714873956	0,474686931	0,995587377	0	FALSE
228	11,3942313	1,036822673	1,378154729	0,752326753	0,451854583	0,995587377	0	FALSE
229	4,959202114	-0,845614412	1,028536603	-0,822152959	0,410989847	0,995587377	0	FALSE
230	8,331920095	1,338079918	0,902408232	1,4827878	0,138130798	0,995587377	0	FALSE
231	102,2274696	0,234214957	0,419347003	0,558523026	0,576487286	0,995587377	0	FALSE
232	12,18101954	-0,423266404	1,049364921	-0,403354825	0,686687215	0,995587377	0	FALSE
233	28,55942765	2,903029604	1,482852774	1,957732861	0,050261367	0,995587377	0	FALSE
234	6,346044254	1,092320328	0,923918199	1,182269523	0,237098775	0,995587377	1	FALSE
235	126,8832622	2,436249615	0,934188076	2,607879161	0,009110511	0,922904169	3	FALSE
236	15,75199479	-0,476020133	1,179599173	-0,40354397	0,686548095	0,995587377	0	FALSE
237	5,816309573	0,768990637	0,960638557	0,80049945	0,423421482	0,995587377	0	FALSE
238	132,4082777	-0,192126249	0,28364902	-0,67733796	0,498191552	0,995587377	0	FALSE
239	11,44192447	-0,136384553	0,983904966	-0,138615575	0,889753935	0,995587377	1	FALSE
240	11,08928413	0,537973924	0,838787616	0,641370848	0,521281769	0,995587377	2	FALSE
241	5,829330495	0,438617141	0,927294393	0,473007434	0,636207876	0,995587377	1	FALSE
242	4,466064881	0,120303393	0,974702897	0,123425705	0,901770003	0,995587377	0	FALSE
243	9,645364585	-0,473190053	0,807258674	-0,586169054	0,557761915	0,995587377	0	FALSE
244	11,25991734	0,199512207	0,833881141	0,239257368	0,810906021	0,995587377	0	FALSE
245	109,8841971	0,34204513	0,413788611	0,826618038	0,40845359	0,995587377	0	FALSE
246	8,771198053	-0,491655204	1,038090468	-0,473614988	0,635774486	0,995587377	0	FALSE
247	5,565852842	-0,670183158	1,398942531	-0,47906411	0,631893021	0,995587377	0	FALSE
248	19,71572758	-0,509327837	1,107065075	-0,460070368	0,645465712	0,995587377	0	FALSE

ASV_nr	Phylum	Class	Order	Family	Genus	Species
249	Actinobacteriota	Coriobacteriia	Coriobacteriales	Atopobiaceae	Olsenella	NA
250	Firmicutes	Clostridia	Clostridia UCG-014	NA	NA	NA
251	Bacteroidota	Bacteroidia	Bacteroidales	Rikenellaceae	Alistipes	NA
252	Firmicutes	Clostridia	Oscillospirales	Oscillospiraceae	UCG-005	NA
253	Verrucomicrobiota	Lentisphaeria	Victivallales	Victivallaceae	Victivallis	NA
254	Actinobacteriota	Actinobacteria	Actinomycetales	Actinomycetaceae	Actinomyces	odontolyticus
255	Firmicutes	Clostridia	Lachnospirales	Lachnospiraceae	NA	NA
256	Bacteroidota	Bacteroidia	Bacteroidales	Marinifilaceae	Butyricimonas	paravirosa
257	Bacteroidota	Bacteroidia	Bacteroidales	Bacteroidaceae	Bacteroides	caccae
258	Bacteroidota	Bacteroidia	Bacteroidales	Bacteroidaceae	Bacteroides	NA
259	Firmicutes	Clostridia	Christensenellales	Christensenellaceae	Christensenellaceae R-7 group	NA
260	Firmicutes	Clostridia	Oscillospirales	Ruminococcaceae	Ruminococcus	NA
261	Firmicutes	Clostridia	Oscillospirales	Oscillospiraceae	Intestinimonas	massiliensis
262	Firmicutes	Clostridia	Lachnospirales	Lachnospiraceae	Blautia	NA
263	Bacteroidota	Bacteroidia	Bacteroidales	Prevotellaceae	Prevotella	NA
264	Firmicutes	Clostridia	Oscillospirales	UCG-010	NA	NA
265	Firmicutes	Clostridia	Lachnospirales	Lachnospiraceae	[Eubacterium] xylanophilum group	NA
266	Firmicutes	Clostridia	Lachnospirales	Lachnospiraceae	NA	NA
267	Firmicutes	Clostridia	Oscillospirales	Oscillospiraceae	UCG-005	NA
268	Firmicutes	Clostridia	Lachnospirales	Lachnospiraceae	Howardella	ureilytica
269	Firmicutes	Clostridia	Peptostreptococcales-Tissierellales	Anaerovoracaceae	Family XIII AD3011 group	NA
270	Firmicutes	Clostridia	Lachnospirales	Lachnospiraceae	Coprococcus	eutactus
271	Firmicutes	Clostridia	Lachnospirales	Lachnospiraceae	Eisenbergiella	tayi
272	Firmicutes	Bacilli	Erysipelotrichales	Erysipelatoclostridiaceae	Erysipelatoclostridium	NA
273	Firmicutes	Clostridia	Christensenellales	Christensenellaceae	Christensenellaceae R-7 group	NA
274	Bacteroidota	Bacteroidia	Bacteroidales	Bacteroidaceae	Bacteroides	nordii
275	Firmicutes	Clostridia	Lachnospirales	Lachnospiraceae	[Ruminococcus] torques group	NA
276	Firmicutes	Clostridia	Oscillospirales	Oscillospiraceae	Colidextribacter	NA
277	Firmicutes	Clostridia	Oscillospirales	Ruminococcaceae	NA	NA
278	Firmicutes	Clostridia	Lachnospirales	Lachnospiraceae	Marvinbryantia	NA
279	Proteobacteria	Gammaproteobacteria	Burkholderiales	Oxalobacteraceae	Oxalobacter	formigenes
280	Bacteroidota	Bacteroidia	Bacteroidales	Bacteroidaceae	Bacteroides	NA
281	Firmicutes	Clostridia	Oscillospirales	UCG-010	NA	NA
282	Firmicutes	Clostridia	Lachnospirales	Lachnospiraceae	[Eubacterium] ventriosum group	NA
283	Firmicutes	Clostridia	Oscillospirales	UCG-010	NA	NA
284	Firmicutes	Clostridia	Monoglobales	Monoglobaceae	Monoglobus	NA

ASV_nr	DESeq_baseMean	DESeq_log2FoldChange	DESeq_lfcSE	DESeq_stat	DESeq_pvalue	DESeq_padj	ANCOM_W	ANCOM_detected_0.7
249	2,956479306	2,861176835	1,303412431	2,195143123	0,028153333	0,995587377	9	FALSE
250	4,508987541	-0,595525607	1,444383712	-0,412304295	0,680116401	0,995587377	0	FALSE
251	241,8563862	-0,32811619	0,629869017	-0,520927654	0,60241717	0,995587377	0	FALSE
252	19,20830524	1,320752073	1,300528874	1,01554998	0,309843735	0,995587377	0	FALSE
253	5,01491906	0,359253439	1,021969019	0,351530655	0,725190279	0,995587377	1	FALSE
254	0,910311303	0,531950774	1,322180852	0,4023283	0,68744243	0,995587377	0	FALSE
255	10,40636247	0,567732421	1,411838455	0,402122792	0,68759366	0,995587377	0	FALSE
256	4,703509114	1,882742563	1,298999405	1,44937908	0,147231748	0,995587377	6	FALSE
257	220,4089884	0,111460507	0,543936155	0,204914687	0,83763878	0,995587377	0	FALSE
258	180,4375122	0,392951634	0,796396346	0,49341215	0,621721399	0,995587377	0	FALSE
259	7,682159214	-0,927092815	0,987424322	-0,93890012	0,347782028	0,995587377	0	FALSE
260	16,0999005	1,833739949	1,703626226	1,076374571	0,28175977	0,995587377	0	FALSE
261	7,240297437	0,215261559	1,046437158	0,205709017	0,837018214	0,995587377	1	FALSE
262	434,1599779	-0,299987623	0,2025804	-1,480832415	0,138651236	0,995587377	0	FALSE
263	359,291962	-1,194322381	1,320570244	-0,904398979	0,365783879	0,995587377	0	FALSE
264	5,67876461	-1,737549424	1,469464982	-1,182436768	0,237032441	0,995587377	0	FALSE
265	8,99614807	1,411151608	1,388411181	1,016378741	0,309449065	0,995587377	0	FALSE
266	7,355994958	-0,307521388	0,886569748	-0,346866548	0,728691579	0,995587377	0	FALSE
267	258,0696551	0,221411261	0,360155463	0,614765799	0,538709399	0,995587377	169	FALSE
268	8,875186308	0,097730244	0,759719052	0,12863998	0,897642529	0,995587377	0	FALSE
269	1,913871748	0,011009629	1,320238765	0,00833912	0,993346422	0,997588574	0	FALSE
270	250,6165594	-0,023451627	0,802521813	-0,029222417	0,976687202	0,995587377	0	FALSE
271	9,143927871	2,715244275	1,497065633	1,813710912	0,069722244	0,995587377	6	FALSE
272	3,679114814	-0,021654719	1,545848019	-0,01400831	0,988823351	0,997588574	0	FALSE
273	2,973407665	0,820344603	1,079254341	0,760103131	0,447192941	0,995587377	0	FALSE
274	5,186506418	2,013133603	1,574062839	1,278941064	0,200917811	0,995587377	0	FALSE
275	283,8263342	-0,075098766	0,391144938	-0,191997285	0,847744332	0,995587377	0	FALSE
276	5,756047726	1,443180153	1,59090494	0,907144177	0,364330559	0,995587377	0	FALSE
277	2,939043546	0,125543221	1,231591275	0,101935783	0,918807647	0,995587377	0	FALSE
278	5,881380331	-0,08188161	1,574407334	-0,052007894	0,958522403	0,995587377	0	FALSE
279	10,84421713	0,481813717	0,484287058	0,994892819	0,319788396	0,995587377	84	FALSE
280	119,2980391	0,476634363	0,760450106	0,626779271	0,530803933	0,995587377	0	FALSE
281	4,361549146	-0,27987688	1,043918249	-0,268102297	0,788620578	0,995587377	0	FALSE
282	4,646443316	-0,664500806	1,470761222	-0,451807402	0,651407737	0,995587377	0	FALSE
283	6,91907006	0,288662158	0,844198724	0,341936265	0,732398858	0,995587377	0	FALSE
284	84,90238623	-0,19827166	0,340906877	-0,581600646	0,560835708	0,995587377	0	FALSE

ASV_nr	Phylum	Class	Order	Family	Genus	Species
285	Firmicutes	Clostridia	Lachnospirales	Lachnospiraceae	NA	NA
286	Firmicutes	Negativicutes	Veillonellales-Selenomonadales	Veillonellaceae	Allisonella	histaminiformans
287	Firmicutes	Bacilli	RF39	NA	NA	NA
288	Bacteroidota	Bacteroidia	Bacteroidales	Bacteroidaceae	Bacteroides	massiliensis
289	Firmicutes	Clostridia	Oscillospirales	Ruminococcaceae	Negativibacillus	massiliensis
290	Firmicutes	Clostridia	Peptostreptococcales-Tissierellales	Anaerovoracaceae	Family XIII UCG-001	NA
291	Firmicutes	Bacilli	Erysipelotrichales	Erysipelatoclostridiaceae	Asteroleplasma	NA
292	Bacteroidota	Bacteroidia	Bacteroidales	Marinifilaceae	Butyricimonas	NA
293	Bacteroidota	Bacteroidia	Bacteroidales	Marinifilaceae	Butyricimonas	NA
294	Firmicutes	Clostridia	Lachnospirales	Lachnospiraceae	Roseburia	inulinivorans
295	Firmicutes	Clostridia	Lachnospirales	Lachnospiraceae	NA	NA
296	Bacteroidota	Bacteroidia	Bacteroidales	Bacteroidaceae	Bacteroides	NA
297	Firmicutes	Clostridia	Lachnospirales	Lachnospiraceae	NA	NA
298	Firmicutes	Clostridia	Oscillospirales	[Eubacterium] coprostanoligenes group	NA	NA
299	Firmicutes	Clostridia	Oscillospirales	Ruminococcaceae	Faecalibacterium	NA
300	Firmicutes	Clostridia	Oscillospirales	Ruminococcaceae	[Eubacterium] siraeum group	NA
301	Firmicutes	Bacilli	Erysipelotrichales	Erysipelatoclostridiaceae	Erysipelatoclostridium	NA
302	Firmicutes	Bacilli	Erysipelotrichales	Erysipelatoclostridiaceae	Catenibacterium	mitsuokai
303	Bacteroidota	Bacteroidia	NA	NA	NA	NA
304	Firmicutes	Clostridia	Clostridia UCG-014	NA	NA	NA
305	Firmicutes	Clostridia	Oscillospirales	Oscillospiraceae	UCG-005	NA
306	Actinobacteriota	Coriobacteriia	Coriobacteriales	Eggerthellaceae	NA	NA
307	Firmicutes	Clostridia	Oscillospirales	UCG-010	NA	NA
308	Firmicutes	Clostridia	Lachnospirales	Lachnospiraceae	NA	NA
309	Firmicutes	Clostridia	Oscillospirales	Oscillospiraceae	UCG-002	NA
310	Firmicutes	Clostridia	Oscillospirales	Oscillospiraceae	Oscillibacter	NA
311	Firmicutes	Clostridia	Oscillospirales	Ruminococcaceae	Incertae Sedis	NA
312	Firmicutes	Clostridia	Lachnospirales	Lachnospiraceae	NA	NA
313	Firmicutes	Clostridia	Oscillospirales	Butyricicoccaceae	Butyricicoccus	NA
314	Firmicutes	Bacilli	Erysipelotrichales	Erysipelotrichaceae	Holdemanella	biformis
315	Firmicutes	Clostridia	Oscillospirales	UCG-010	NA	NA
316	Firmicutes	Clostridia	Oscillospirales	UCG-010	NA	NA
317	Firmicutes	Clostridia	Oscillospirales	NA	NA	NA
318	Firmicutes	Clostridia	Oscillospirales	Ruminococcaceae	Faecalibacterium	NA
319	Firmicutes	Clostridia	Oscillospirales	Ruminococcaceae	Angelakisella	NA
320	Firmicutes	Clostridia	Christensenellales	Christensenellaceae	NA	NA

ASV_nr	DESeq_baseMean	DESeq_log2FoldChange	DESeq_lfcSE	DESeq_stat	DESeq_pvalue	DESeq_padj	ANCOM_W	ANCOM_detected_0.7
285	6,474201915	-0,796454809	1,370981233	-0,580937791	0,561282381	0,995587377	0	FALSE
286	11,34439485	-0,652418067	1,138316239	-0,573143073	0,566547825	0,995587377	0	FALSE
287	9,803994393	0,811956177	1,619818324	0,501263731	0,616185527	0,995587377	0	FALSE
288	199,2806198	-0,589714403	0,791068511	-0,745465652	0,455990268	0,995587377	0	FALSE
289	7,22907177	1,346345812	1,336659683	1,007246518	0,313816324	0,995587377	5	FALSE
290	3,192781755	0,834408663	1,35840034	0,614258285	0,539044664	0,995587377	1	FALSE
291	6,072821769	0,841757883	1,150686779	0,73152651	0,464457618	0,995587377	0	FALSE
292	8,664115317	0,222409701	1,095289372	0,20306022	0,839087964	0,995587377	0	FALSE
293	7,470951947	-0,264429941	1,08049097	-0,244731282	0,806664505	0,995587377	0	FALSE
294	266,7571399	-0,139784787	0,408016069	-0,342596278	0,731902203	0,995587377	153	FALSE
295	9,881712389	-0,742221406	0,889493532	-0,834431481	0,404037883	0,995587377	4	FALSE
296	113,7637962	-0,48896449	0,564726708	-0,865842686	0,386576437	0,995587377	0	FALSE
297	10,60718753	0,379299028	1,409843821	0,269036203	0,787901823	0,995587377	0	FALSE
298	157,9205752	-0,800140729	0,7820701	-1,023106151	0,306257659	0,995587377	0	FALSE
299	1008,451776	-0,338701937	0,219589905	-1,542429454	0,122969269	0,995587377	144	FALSE
300	163,0258671	-0,586910079	0,583053161	-1,006615037	0,314119806	0,995587377	0	FALSE
301	2,592280315	1,029434561	1,268353045	0,811630929	0,417003438	0,995587377	0	FALSE
302	57,19302683	0,402812749	1,543977893	0,260892822	0,794175161	0,995587377	1	FALSE
303	5,670000655	-0,49000608	1,3319364	-0,367889998	0,712955258	0,995587377	0	FALSE
304	174,7170819	0,815508002	0,911793892	0,894399501	0,371108179	0,995587377	3	FALSE
305	4,471811591	-0,710946064	1,532310952	-0,463969838	0,642669356	0,995587377	0	FALSE
306	73,27392831	0,143536021	0,447388211	0,32083103	0,748338443	0,995587377	3	FALSE
307	5,202169909	-0,535657946	1,449666308	-0,369504308	0,711751863	0,995587377	0	FALSE
308	4,602419789	1,849792467	1,460668748	1,266401071	0,205369528	0,995587377	0	FALSE
309	221,972934	0,471047917	0,369614029	1,274431919	0,202510386	0,995587377	2	FALSE
310	7,748676656	0,459987773	0,899261458	0,511517278	0,608988893	0,995587377	0	FALSE
311	60,86168788	-0,390191518	0,395284501	-0,987115654	0,323585944	0,995587377	0	FALSE
312	7,498304674	-0,432685008	1,352121012	-0,320004648	0,748964807	0,995587377	0	FALSE
313	94,87113862	-0,088100519	0,367460328	-0,239755184	0,810520052	0,995587377	0	FALSE
314	26,08063339	1,215784722	1,692448007	0,718358683	0,472536155	0,995587377	0	FALSE
315	8,819422285	-0,889486021	1,443027554	-0,616402659	0,537628799	0,995587377	0	FALSE
316	3,160872788	0,781543034	1,193852545	0,654639501	0,512699885	0,995587377	0	FALSE
317	8,570385078	0,064984641	0,935712479	0,069449369	0,944631933	0,995587377	2	FALSE
318	131,1122678	-0,103470808	0,482133544	-0,214610267	0,830071189	0,995587377	0	FALSE
319	2,863681831	-1,043501291	1,294240851	-0,806265148	0,420089982	0,995587377	0	FALSE
320	66,80396032	0,675171054	0,668942311	1,009311331	0,312825348	0,995587377	210	FALSE

ASV_nr	Phylum	Class	Order	Family	Genus	Species
321	Firmicutes	Clostridia	Lachnospirales	Lachnospiraceae	Agathobacter	NA
322	Firmicutes	Clostridia	Lachnospirales	Lachnospiraceae	Roseburia	intestinalis
323	Firmicutes	Clostridia	Oscillospirales	[Eubacterium] coprostanoligenes group	NA	NA
324	Firmicutes	Clostridia	Lachnospirales	Lachnospiraceae	NA	NA
325	Proteobacteria	Gammaproteobacteria	Burkholderiales	Sutterellaceae	Parasutterella	excrementihominis
326	Firmicutes	Clostridia	Oscillospirales	Oscillospiraceae	Flavonifractor	NA
327	Firmicutes	Clostridia	Oscillospirales	Oscillospiraceae	UCG-005	NA
328	Firmicutes	Clostridia	Lachnospirales	Lachnospiraceae	NA	NA
329	Firmicutes	Bacilli	Erysipelotrichales	Erysipelotrichaceae	Holdemania	filiformis
330	Firmicutes	Clostridia	Lachnospirales	Lachnospiraceae	Coprococcus	catus
331	Firmicutes	Clostridia	Christensenellales	Christensenellaceae	Christensenellaceae R-7 group	NA
332	Firmicutes	Clostridia	Lachnospirales	Lachnospiraceae	[Ruminococcus] gauvreauii group	NA
333	Firmicutes	Clostridia	Oscillospirales	Oscillospiraceae	NA	NA
334	Firmicutes	Clostridia	Lachnospirales	Lachnospiraceae	Lachnospiraceae ND3007 group	NA
335	Firmicutes	Clostridia	Lachnospirales	Lachnospiraceae	[Ruminococcus] torques group	NA
336	Firmicutes	Clostridia	Oscillospirales	UCG-010	NA	NA
337	Firmicutes	Clostridia	Oscillospirales	Hydrogenoanaerobacterium	NA	NA
338	Firmicutes	Clostridia	Lachnospirales	Lachnospiraceae	[Eubacterium] eligens group	NA
339	Firmicutes	Clostridia	Lachnospirales	Lachnospiraceae	NA	NA
340	Firmicutes	Clostridia	Lachnospirales	Lachnospiraceae	[Eubacterium] ruminantium group	NA
341	Firmicutes	Clostridia	Oscillospirales	Butyricicoccaceae	UCG-009	NA
342	Firmicutes	Clostridia	Oscillospirales	Ruminococcaceae	Faecalibacterium	prausnitzii
343	Firmicutes	Negativicutes	Acidaminococcales	Acidaminococcaceae	Phascolarctobacterium	faecium
344	Verrucomicrobiota	Lentisphaeria	Victivallales	vadinBE97	NA	NA
345	Firmicutes	Clostridia	Lachnospirales	Lachnospiraceae	Lachnospiraceae NK4A136 group	NA
346	Firmicutes	Clostridia	Lachnospirales	Lachnospiraceae	Roseburia	NA
347	Firmicutes	Clostridia	Lachnospirales	Lachnospiraceae	Coprococcus	eutactus
348	Firmicutes	Clostridia	Lachnospirales	Lachnospiraceae	Lachnospiraceae NK4A136 group	NA
349	Bacteroidota	Bacteroidia	Bacteroidales	Bacteroidaceae	Bacteroides	NA
350	Firmicutes	Clostridia	Lachnospirales	Lachnospiraceae	Coprococcus	NA
351	Firmicutes	Clostridia	Oscillospirales	UCG-010	NA	NA
352	Firmicutes	Clostridia	Lachnospirales	Lachnospiraceae	[Eubacterium] hallii group	NA
353	Proteobacteria	Gammaproteobacteria	Burkholderiales	Sutterellaceae	Sutterella	wadsworthensis
354	Firmicutes	Clostridia	Lachnospirales	Lachnospiraceae	Dorea	formicigenerans
355	Firmicutes	Clostridia	Lachnospirales	Lachnospiraceae	Roseburia	NA
356	Firmicutes	Clostridia	Lachnospirales	Lachnospiraceae	Blautia	NA

ASV_nr	DESeq_baseMean	DESeq_log2FoldChange	DESeq_lfcSE	DESeq_stat	DESeq_pvalue	DESeq_padj	ANCOM_W	ANCOM_detected_0.7
321	2021,030813	-0,14467615	0,339691274	-0,425904818	0,670177204	0,995587377	0	FALSE
322	241,8863028	-0,377832439	0,730641924	-0,517123951	0,605069635	0,995587377	0	FALSE
323	45,90996482	-0,513020814	0,775203367	-0,661788681	0,508106661	0,995587377	0	FALSE
324	7,258924325	0,479635844	1,448940551	0,331025206	0,740625446	0,995587377	0	FALSE
325	57,99099133	-0,072208111	0,686609983	-0,105166124	0,916243991	0,995587377	0	FALSE
326	1,80845778	0,55443888	1,210595523	0,457988543	0,646960672	0,995587377	0	FALSE
327	190,315889	-0,134911824	0,52314699	-0,257885119	0,79649557	0,995587377	0	FALSE
328	12,0346784	-1,011630074	1,091350671	-0,926952354	0,353951273	0,995587377	0	FALSE
329	2,343805374	-0,360781617	0,723754131	-0,498486435	0,618141228	0,995587377	0	FALSE
330	105,3966077	-0,488017276	0,252561199	-1,932273359	0,053325775	0,995587377	0	FALSE
331	4,686277678	1,937131868	1,11966735	1,730095879	0,083613146	0,995587377	9	FALSE
332	77,6602915	-0,813923934	0,637224763	-1,277294892	0,201498156	0,995587377	161	FALSE
333	7,551672663	-0,692554055	0,995629707	-0,695594005	0,486683121	0,995587377	0	FALSE
334	56,09561636	0,198259697	0,368536869	0,537964351	0,59060166	0,995587377	0	FALSE
335	131,5150414	0,794723963	0,659076204	1,205814986	0,227888836	0,995587377	0	FALSE
336	4,123719857	-0,226899239	0,729481076	-0,311041981	0,755768707	0,995587377	0	FALSE
337	4,666892327	0,313786575	1,168862309	0,268454696	0,788349343	0,995587377	6	FALSE
338	120,8716664	-0,893741839	0,566401035	-1,577931155	0,114581427	0,995587377	0	FALSE
339	5,853763746	-0,11291514	0,757333779	-0,149095607	0,881478192	0,995587377	0	FALSE
340	230,8311531	-0,210831789	0,834349084	-0,252690143	0,800507665	0,995587377	0	FALSE
341	1,877880501	-0,885452588	1,09640181	-0,807598619	0,419321682	0,995587377	0	FALSE
342	1081,558543	0,051736206	0,285408739	0,181270574	0,856155202	0,995587377	0	FALSE
343	78,27607178	-0,691886257	1,238667009	-0,55857325	0,576453	0,995587377	0	FALSE
344	2,933213734	-1,408008194	1,390157027	-1,012841115	0,311136058	0,995587377	1	FALSE
345	166,9205722	-0,417213099	0,590207575	-0,706892144	0,479633506	0,995587377	0	FALSE
346	6,243718479	-0,24394062	1,59004976	-0,153416972	0,878069462	0,995587377	0	FALSE
347	119,7050833	-0,984902835	1,191806747	-0,826394747	0,408580202	0,995587377	0	FALSE
348	74,61267569	-0,763073867	0,65381504	-1,167109687	0,243166076	0,995587377	285	TRUE
349	55,95823586	-0,420960183	0,825457978	-0,509971669	0,61007131	0,995587377	0	FALSE
350	238,8886707	0,257764616	0,890514195	0,289455931	0,772232499	0,995587377	0	FALSE
351	4,508011422	-0,251135454	1,516602858	-0,165590783	0,868479001	0,995587377	0	FALSE
352	43,26719511	0,161153684	0,500319228	0,322101721	0,747375635	0,995587377	0	FALSE
353	25,09175593	0,131175915	0,824619045	0,159074564	0,873610128	0,995587377	0	FALSE
354	52,92407504	0,26686067	0,540824475	0,493433049	0,621706635	0,995587377	0	FALSE
355	3,977355993	0,58779203	1,58489239	0,370871886	0,710732956	0,995587377	0	FALSE
356	47,59771532	0,46285443	0,63896157	0,724385396	0,468829163	0,995587377	0	FALSE

ASV_nr	Phylum	Class	Order	Family	Genus	Species
357	Bacteroidota	Bacteroidia	Bacteroidales	Bacteroidaceae	Bacteroides	NA
358	Firmicutes	Clostridia	Lachnospirales	Lachnospiraceae	[Eubacterium] eligens group	NA
359	Firmicutes	Clostridia	Peptococcales	Peptococcaceae	Peptococcus	NA
360	Firmicutes	Clostridia	Christensenellales	Christensenellaceae	Christensenellaceae R-7 group	NA
361	Firmicutes	Bacilli	Erysipelotrichales	Erysipelotrichaceae	Turicibacter	sanguinis
362	Firmicutes	Clostridia	Lachnospirales	Lachnospiraceae	NA	NA
363	Firmicutes	Clostridia	Peptococcales	Peptococcaceae	NA	NA
364	Desulfobacterota	Desulfovibrionia	Desulfovibrionales	Desulfovibrionaceae	NA	NA
365	Firmicutes	Clostridia	Oscillospirales	Oscillospiraceae	NK4A214 group	NA
366	Bacteroidota	Bacteroidia	Bacteroidales	Rikenellaceae	Alistipes	shahii
367	Firmicutes	Clostridia	Oscillospirales	Oscillospiraceae	Colidextribacter	NA
368	Bacteroidota	Bacteroidia	Bacteroidales	Prevotellaceae	Prevotella	NA
369	Firmicutes	Clostridia	Lachnospirales	Lachnospiraceae	CAG-56	NA
370	Firmicutes	Clostridia	Lachnospirales	Lachnospiraceae	[Ruminococcus] gnavus group	NA
371	Firmicutes	Negativicutes	Acidaminococcales	Acidaminococcaceae	Phascolarctobacterium	succinatutens
372	Bacteroidota	Bacteroidia	Bacteroidales	Bacteroidaceae	Bacteroides	plebeius

ASV_nr	DESeq_baseMean	DESeq_log2FoldChange	DESeq_lfcSE	DESeq_stat	DESeq_pvalue	DESeq_padj	ANCOM_W	ANCOM_detected_0.7
357	653,890877	-0,087083343	0,596194391	-0,146065351	0,883869799	0,995587377	0	FALSE
358	112,7913315	-0,341697212	0,65268082	-0,523528809	0,600606308	0,995587377	0	FALSE
359	4,287588983	0,600205335	1,177405407	0,509769474	0,610212974	0,995587377	0	FALSE
360	1,671777459	-0,675197986	0,971098089	-0,695293291	0,486871518	0,995587377	0	FALSE
361	9,021331384	1,001540217	1,094704569	0,914895438	0,360246525	0,995587377	0	FALSE
362	4,655036569	0,682337298	1,380916013	0,494119332	0,621221906	0,995587377	0	FALSE
363	4,117548349	-0,771377081	1,179250464	-0,65412489	0,513031347	0,995587377	0	FALSE
364	1,451217894	0,57744427	1,029178072	0,561073235	0,574747613	0,995587377	1	FALSE
365	98,88149805	-0,619049506	0,53426818	-1,158686835	0,246583859	0,995587377	0	FALSE
366	98,81279479	0,296757772	0,658504963	0,450653813	0,652239076	0,995587377	0	FALSE
367	2,047129773	0,953602957	1,340522346	0,711366699	0,476857029	0,995587377	0	FALSE
368	61,54695961	-1,127595174	1,827048361	-0,617167666	0,53712414	0,995587377	0	FALSE
369	156,8983442	0,073131732	0,456187493	0,16031069	0,872636338	0,995587377	0	FALSE
370	31,28853031	-1,062116615	1,57485441	-0,674422098	0,500042998	0,995587377	0	FALSE
371	219,2496814	0,581579168	0,854020014	0,680990092	0,49587776	0,995587377	0	FALSE
372	129,2407826	-1,007244447	1,511512755	-0,666381705	0,505167153	0,995587377	0	FALSE

Supplementary table 5b: Differential abundance analysis at ASV level of FIN cohort with DESeq2 and ANCOM
All columns are spread out over two pages, after which the next two pages show the next set of rows, etc.
ASVs are numbered on both pages to easily connect the taxonomy data to the associated statistics.

ASV_nr	Phylum	Class	Order	Family	Genus	Species
1	Actinobacteriota	Actinobacteria	Bifidobacteriales	Bifidobacteriaceae	Bifidobacterium	NA
2	Verrucomicrobiota	Verrucomicrobiae	Verrucomicrobiales	Akkermansiaceae	Akkermansia	muciniphila
3	Firmicutes	Clostridia	Christensenellales	Christensenellaceae	NA	NA
4	Firmicutes	Clostridia	Lachnospirales	Lachnospiraceae	Roseburia	NA
5	Bacteroidota	Bacteroidia	Bacteroidales	Rikenellaceae	Alistipes	NA
6	Firmicutes	Incertae Sedis	DTU014	NA	NA	NA
7	Firmicutes	Clostridia	Clostridia UCG-014	NA	NA	NA
8	Firmicutes	Clostridia	Oscillospirales	Oscillospiraceae	Colidextribacter	NA
9	Firmicutes	Clostridia	Christensenellales	Christensenellaceae	Christensenellaceae R-7 group	NA
10	Firmicutes	Clostridia	Clostridia vadinBB60 group	NA	NA	NA
11	Firmicutes	Clostridia	Clostridia vadinBB60 group	NA	NA	NA
12	Bacteroidota	Bacteroidia	Bacteroidales	Bacteroidaceae	Bacteroides	NA
13	Firmicutes	Clostridia	Oscillospirales	Ruminococcaceae	Angelakisella	NA
14	Bacteroidota	Bacteroidia	Bacteroidales	Bacteroidaceae	Bacteroides	eggerthii
15	Firmicutes	Clostridia	Oscillospirales	[Eubacterium] coprostanoligenes group	NA	NA
16	Firmicutes	Clostridia	Oscillospirales	Ruminococcaceae	Subdoligranulum	NA
17	Firmicutes	Clostridia	Oscillospirales	Ruminococcaceae	Faecalibacterium	prausnitzii
18	Firmicutes	Clostridia	Lachnospirales	Lachnospiraceae	Lachnospira	pectinoschiza
19	Firmicutes	Clostridia	Oscillospirales	Oscillospiraceae	UCG-002	NA
20	Verrucomicrobiota	Verrucomicrobiae	Verrucomicrobiales	Akkermansiaceae	Akkermansia	muciniphila
21	Firmicutes	Clostridia	Oscillospirales	Oscillospiraceae	UCG-003	NA
22	Bacteroidota	Bacteroidia	Bacteroidales	Rikenellaceae	Alistipes	finegoldii
23	Firmicutes	Bacilli	Erysipelotrichales	Erysipelotrichaceae	Holdemania	NA
24	Firmicutes	Clostridia	Clostridia UCG-014	NA	NA	NA
25	Firmicutes	Clostridia	Oscillospirales	Ruminococcaceae	Subdoligranulum	NA
26	Firmicutes	Negativicutes	Veillonellales-Selenomonadales	Veillonellaceae	Dialister	invisus
27	Bacteroidota	Bacteroidia	Bacteroidales	Barnesiellaceae	Barnesiella	NA
28	Firmicutes	Clostridia	Lachnospirales	Lachnospiraceae	[Ruminococcus] torques group	NA
29	Firmicutes	Clostridia	Clostridia UCG-014	NA	NA	NA
30	Firmicutes	Clostridia	Oscillospirales	Oscillospiraceae	UCG-002	NA
31	Bacteroidota	Bacteroidia	Bacteroidales	Tannerellaceae	Parabacteroides	distasonis
32	Firmicutes	Clostridia	Lachnospirales	Lachnospiraceae	Lachnospiraceae NK4A136 group	bacterium

ASV_nr	DESeq_baseMean	DESeq_log2FoldChange	DESeq_lfcSE	DESeq_stat	DESeq_pvalue	DESeq_padj	ANCOM_W	ANCOM_detected_0.7
1	511,4776895	0,87045176	0,810561196	1,073887777	0,282872982	0,996224111	0	FALSE
2	633,6841803	0,578892553	0,745779105	0,776225224	0,437616015	0,996224111	0	FALSE
3	55,04118575	-0,242463467	0,861797803	-0,281346119	0,77844494	0,996224111	0	FALSE
4	124,3298783	-0,374195425	1,044946558	-0,35810006	0,720268434	0,996224111	0	FALSE
5	167,3259983	-0,927400367	2,156134138	-0,430121832	0,667107019	0,996224111	0	FALSE
6	2,483803603	1,988752854	0,850130669	2,339349615	0,019317347	0,562118803	107	FALSE
7	96,4204796	-0,079980446	1,898735357	-0,042123009	0,966400638	0,997979415	0	FALSE
8	2,168800241	-0,69132884	1,578633896	-0,437928542	0,661438085	0,996224111	4	FALSE
9	71,91449177	-0,62021999	0,677412629	-0,915571932	0,359891458	0,996224111	0	FALSE
10	1,306506245	-0,254691493	1,296721917	-0,196411805	0,844287857	0,996224111	22	FALSE
11	2,0537852	1,038713263	1,26746252	0,819521876	0,412488726	0,996224111	1	FALSE
12	166,5978956	-0,192089522	0,829862188	-0,231471592	0,816948453	0,996224111	0	FALSE
13	2,425236265	-0,389667263	1,418831703	-0,274639523	0,783593198	0,996224111	0	FALSE
14	203,8372926	0,426285707	1,525615157	0,279418899	0,779923369	0,996224111	0	FALSE
15	58,68377853	-0,248163826	1,040153555	-0,238583837	0,811428299	0,996224111	0	FALSE
16	737,5246076	-0,389638644	0,456184972	-0,854124244	0,393036152	0,996224111	0	FALSE
17	53,4392816	0,26526571	2,073601017	0,127925144	0,898208212	0,996224111	0	FALSE
18	175,7242827	-1,072556974	0,650248157	-1,649457923	0,099053857	0,967944373	0	FALSE
19	140,5551903	0,280496093	1,523013206	0,184171806	0,853878676	0,996224111	0	FALSE
20	68,26318689	11,12078716	1,809648727	6,145273938	7,98256E-10	2,83381E-07	141	FALSE
21	96,39915297	-0,590447681	0,637166147	-0,926677734	0,354093882	0,996224111	12	FALSE
22	111,2911343	-0,172730843	0,949088292	-0,1819966	0,855585394	0,996224111	0	FALSE
23	1,804919083	-2,199045361	1,476268513	-1,489597144	0,136330193	0,967944373	9	FALSE
24	90,24106477	1,920633217	2,217339072	0,866188325	0,386386895	0,996224111	0	FALSE
25	99,38742011	-0,583946453	0,840954399	-0,694385396	0,48744055	0,996224111	0	FALSE
26	470,1611405	0,231573379	1,014039892	0,228367129	0,819360842	0,996224111	0	FALSE
27	131,553472	1,246519786	1,500660652	0,830647345	0,40617288	0,996224111	0	FALSE
28	42,86099588	-0,332171014	1,07743403	-0,308298239	0,757855406	0,996224111	0	FALSE
29	86,7221738	1,056873843	1,460444342	0,723665951	0,469270842	0,996224111	0	FALSE
30	25,24409862	-0,618635123	1,844673549	-0,335362928	0,73735133	0,996224111	0	FALSE
31	103,0146048	-0,792957981	0,959031089	-0,826832404	0,408332061	0,996224111	0	FALSE
32	95,78871909	-0,163294251	0,884086868	-0,184703853	0,853461324	0,996224111	0	FALSE

ASV_nr	Phylum	Class	Order	Family	Genus	Species
33	Firmicutes	Clostridia	NA	NA	NA	NA
34	Firmicutes	Clostridia	Oscillospirales	Oscillospiraceae	NK4A214 group	NA
35	Firmicutes	Clostridia	Christensenellales	Christensenellaceae	Christensenellaceae R-7 group	NA
36	Firmicutes	Clostridia	Lachnospirales	Lachnospiraceae	Fusicatenibacter	saccharivorans
37	Firmicutes	Clostridia	Lachnospirales	Lachnospiraceae	NA	NA
38	Firmicutes	Clostridia	Oscillospirales	Ruminococcaceae	CAG-352	NA
39	Actinobacteriota	Coriobacteriia	Coriobacteriales	Eggerthellaceae	Senegalimassilia	anaerobia
40	Actinobacteriota	Coriobacteriia	Coriobacteriales	Eggerthellaceae	Enterorhabdus	NA
41	Bacteroidota	Bacteroidia	Bacteroidales	Rikenellaceae	Alistipes	inops
42	Firmicutes	Clostridia	Oscillospirales	Ruminococcaceae	NA	NA
43	Firmicutes	Clostridia	Clostridia vadinBB60 group	NA	NA	NA
44	Firmicutes	Clostridia	Oscillospirales	Butyricicoccaceae	Butyricicoccus	faecihominis
45	Bacteroidota	Bacteroidia	Bacteroidales	Rikenellaceae	Alistipes	obesi
46	Firmicutes	Clostridia	Christensenellales	Christensenellaceae	Christensenellaceae R-7 group	NA
47	Firmicutes	Clostridia	Lachnospirales	Lachnospiraceae	Blautia	massiliensis
48	Bacteroidota	Bacteroidia	Bacteroidales	Tannerellaceae	Parabacteroides	distasonis
49	Firmicutes	Clostridia	Christensenellales	Christensenellaceae	NA	NA
50	Firmicutes	Clostridia	Lachnospirales	Lachnospiraceae	Lachnoclostridium	NA
51	Firmicutes	Clostridia	Peptococcales	Peptococcaceae	NA	NA
52	Firmicutes	Clostridia	Oscillospirales	Ruminococcaceae	Faecalibacterium	prausnitzii
53	Desulfobacterota	Desulfovibrionia	Desulfovibrionales	Desulfovibrionaceae	Bilophila	wadsworthia
54	Firmicutes	Clostridia	Oscillospirales	Oscillospiraceae	Oscillibacter	NA
55	Firmicutes	Clostridia	Clostridia UCG-014	NA	NA	NA
56	Firmicutes	Clostridia	Oscillospirales	Oscillospiraceae	UCG-002	NA
57	Firmicutes	Clostridia	Christensenellales	Christensenellaceae	Christensenellaceae R-7 group	NA
58	Proteobacteria	Gammaproteobacteria	Burkholderiales	Sutterellaceae	Sutterella	NA
59	Firmicutes	Clostridia	Oscillospirales	Butyricicoccaceae	Butyricicoccus	NA
60	Firmicutes	Clostridia	Lachnospirales	Lachnospiraceae	Roseburia	hominis
61	Firmicutes	Bacilli	Lactobacillales	Streptococcaceae	Streptococcus	NA
62	Firmicutes	Clostridia	Clostridia UCG-014	NA	NA	NA
63	Firmicutes	Clostridia	Oscillospirales	Ruminococcaceae	Ruminococcus	NA
64	Firmicutes	Clostridia	Oscillospirales	Oscillospiraceae	UCG-005	NA
65	Firmicutes	Clostridia	Lachnospirales	Lachnospiraceae	[Eubacterium] ventriosum group	NA
66	Firmicutes	Clostridia	Peptostreptococcales-Tissierellales	Peptostreptococcaceae	Romboutsia	NA
67	Firmicutes	Clostridia	Lachnospirales	Lachnospiraceae	Roseburia	NA
68	Firmicutes	Clostridia	Oscillospirales	Ruminococcaceae	Ruminococcus	callidus

ASV_nr	DESeq_baseMean	DESeq_log2FoldChange	DESeq_lfcSE	DESeq_stat	DESeq_pvalue	DESeq_padj	ANCOM_W	ANCOM_detected_0.7
33	1,218380027	1,349622123	0,960494947	1,405131934	0,15998205	0,991381803	21	FALSE
34	99,40850097	-0,608212144	1,060366518	-0,573586712	0,566247506	0,996224111	0	FALSE
35	65,9503273	-0,153635805	1,01139655	-0,151904616	0,879262167	0,996224111	0	FALSE
36	269,6180254	-0,386552823	0,374848146	-1,031225117	0,302435265	0,996224111	0	FALSE
37	8,265744559	-0,779684793	1,500939841	-0,519464386	0,603436942	0,996224111	2	FALSE
38	75,41809841	-1,917996444	2,114484001	-0,907075411	0,36436692	0,996224111	1	FALSE
39	5,978595646	2,298809964	1,091524599	2,106054197	0,035199645	0,694215217	64	FALSE
40	2,272908693	0,427545246	1,307638459	0,326959828	0,74369827	0,996224111	9	FALSE
41	73,49593863	0,291687627	0,860000752	0,339171363	0,734480641	0,996224111	0	FALSE
42	58,3693828	0,593696889	0,873025444	0,680045344	0,49647575	0,996224111	0	FALSE
43	3,549117497	0,282640124	1,346478615	0,209910593	0,833737454	0,996224111	15	FALSE
44	42,44303883	-1,417260485	0,810670403	-1,748257342	0,080419476	0,920932712	84	FALSE
45	106,2245167	0,72010337	0,637010176	1,130442492	0,258289818	0,996224111	0	FALSE
46	71,60910791	-0,476752385	1,171202916	-0,407062157	0,683962337	0,996224111	0	FALSE
47	89,10505552	-0,428516003	0,470996288	-0,909807602	0,362923985	0,996224111	0	FALSE
48	117,3591109	0,047694364	1,054344816	0,045236021	0,963919183	0,997979415	0	FALSE
49	1,684951718	-2,198176171	1,597185532	-1,376281044	0,168734639	0,991381803	3	FALSE
50	99,74373642	-0,658494091	0,661493999	-0,995464951	0,319510182	0,996224111	216	FALSE
51	1,014407365	0,166652443	1,167923665	0,142691212	0,886534057	0,996224111	10	FALSE
52	56,5305617	0,220331543	1,646851454	0,133789567	0,893568977	0,996224111	0	FALSE
53	77,16443846	-0,288450078	0,570445019	-0,505657984	0,613096769	0,996224111	71	FALSE
54	62,14091111	-0,279453281	0,582552995	-0,479704478	0,631437543	0,996224111	0	FALSE
55	114,2610746	-0,143973827	0,953363024	-0,151016794	0,879962468	0,996224111	0	FALSE
56	82,51350304	0,315551613	1,533660312	0,205750654	0,836985689	0,996224111	0	FALSE
57	1082,928056	-0,043130733	0,826528551	-0,052182993	0,958382884	0,997979415	0	FALSE
58	66,76218188	-0,951999713	1,909210208	-0,498635357	0,618036292	0,996224111	0	FALSE
59	27,96749208	-1,989228616	0,988694075	-2,011975865	0,044222481	0,736528727	0	FALSE
60	41,49100226	-0,230136314	1,048541982	-0,219482212	0,826274435	0,996224111	0	FALSE
61	26,04363458	1,046493651	1,043441902	1,002924695	0,315897197	0,996224111	3	FALSE
62	133,1163897	-1,06439932	1,699543952	-0,626285257	0,531127855	0,996224111	0	FALSE
63	16,95695437	-1,276589734	1,79455404	-0,711368789	0,476855735	0,996224111	0	FALSE
64	72,97388549	0,330917743	0,649996626	0,509106862	0,610677322	0,996224111	0	FALSE
65	16,88975181	-0,095938585	0,876012398	-0,109517383	0,912792135	0,996224111	0	FALSE
66	17,01463137	-1,188084893	1,116692045	-1,063932441	0,287359306	0,996224111	0	FALSE
67	15,6449415	-2,917336051	1,032588701	-2,825264356	0,004724162	0,238304316	207	FALSE
68	30,24630865	0,779072143	1,649666322	0,47226044	0,636740904	0,996224111	21	FALSE

ASV_nr	Phylum	Class	Order	Family	Genus	Species
69	Firmicutes	Clostridia	Oscillospirales	Oscillospiraceae	UCG-002	NA
70	Bacteroidota	Bacteroidia	Bacteroidales	Tannerellaceae	Parabacteroides	distasonis
71	Firmicutes	Clostridia	Oscillospirales	Oscillospiraceae	Intestinimonas	NA
72	Firmicutes	Clostridia	NA	NA	NA	NA
73	Firmicutes	Clostridia	Lachnospirales	Lachnospiraceae	Lachnoclostridium	edouardi
74	Actinobacteriota	Coriobacteriia	Coriobacteriales	Coriobacteriaceae	Collinsella	aerofaciens
75	Firmicutes	Clostridia	Oscillospirales	Oscillospiraceae	NA	NA
76	Proteobacteria	Gammaproteobacteria	Pasteurellales	Pasteurellaceae	Haemophilus	NA
77	Actinobacteriota	Coriobacteriia	Coriobacteriales	Coriobacteriales Incertae Sedis	NA	NA
78	Firmicutes	Bacilli	Izemoplasmatales	NA	NA	NA
79	Bacteroidota	Bacteroidia	Bacteroidales	Prevotellaceae	Paraprevotella	NA
80	Firmicutes	Clostridia	Lachnospirales	Lachnospiraceae	Dorea	longicatena
81	Firmicutes	Clostridia	Clostridia vadinBB60 group	NA	NA	NA
82	Firmicutes	Clostridia	Oscillospirales	Ruminococcaceae	Ruminococcus	NA
83	Firmicutes	Clostridia	Oscillospirales	Ruminococcaceae	Ruminococcus	NA
84	Firmicutes	Bacilli	Erysipelotrichales	Erysipelatoclostridiaceae	Erysipelotrichaceae UCG-003	bacterium
85	Firmicutes	Clostridia	Oscillospirales	Ruminococcaceae	Ruminococcus	champanellensis
86	Firmicutes	Clostridia	Oscillospirales	Ruminococcaceae	UBA1819	NA
87	Firmicutes	Clostridia	Lachnospirales	Lachnospiraceae	Lachnospiraceae NK4A136 group	NA
88	Bacteroidota	Bacteroidia	Bacteroidales	Marinifilaceae	Odoribacter	splanchnicus
89	Actinobacteriota	Coriobacteriia	Coriobacteriales	Eggerthellaceae	Adlercreutzia	equolifaciens
90	Bacteroidota	Bacteroidia	Bacteroidales	Bacteroidaceae	Bacteroides	intestinalis
91	Firmicutes	Clostridia	Oscillospirales	Ruminococcaceae	Ruminococcus	NA
92	Bacteroidota	Bacteroidia	Bacteroidales	Bacteroidaceae	Bacteroides	eggerthii
93	Firmicutes	Clostridia	Lachnospirales	Lachnospiraceae	Lachnospiraceae UCG-010	NA
94	Bacteroidota	Bacteroidia	Bacteroidales	Bacteroidaceae	Bacteroides	vulgatus
95	Bacteroidota	Bacteroidia	Bacteroidales	Prevotellaceae	Prevotella	copri
96	Firmicutes	Clostridia	Lachnospirales	Lachnospiraceae	[Eubacterium] xylanophilum group	NA
97	Firmicutes	Clostridia	Oscillospirales	Oscillospiraceae	UCG-002	NA
98	Firmicutes	Clostridia	Christensenellales	Christensenellaceae	NA	NA
99	Firmicutes	Clostridia	Clostridia UCG-014	NA	NA	NA
100	Firmicutes	Bacilli	RF39	NA	NA	NA
101	Firmicutes	Clostridia	Oscillospirales	Oscillospiraceae	Intestinimonas	NA
102	Firmicutes	Clostridia	Oscillospirales	Ruminococcaceae	Faecalibacterium	prausnitzii
103	Bacteroidota	Bacteroidia	Bacteroidales	Tannerellaceae	NA	NA
104	Firmicutes	Clostridia	Lachnospirales	Lachnospiraceae	Lachnospiraceae FCS020 group	NA

ASV_nr	DESeq_baseMean	DESeq_log2FoldChange	DESeq_lfcSE	DESeq_stat	DESeq_pvalue	DESeq_padj	ANCOM_W	ANCOM_detected_0.7
69	82,11081055	-0,107333079	0,943274886	-0,1137877	0,909406089	0,996224111	0	FALSE
70	66,47958654	-0,204423084	1,471000557	-0,138968733	0,889474857	0,996224111	0	FALSE
71	7,136835119	-0,727890966	1,269869927	-0,5732012	0,566508472	0,996224111	0	FALSE
72	1,630956379	2,136051399	1,375409361	1,553029562	0,120416072	0,967944373	22	FALSE
73	83,0769331	-0,963494198	0,586191044	-1,643652198	0,100248065	0,967944373	252	TRUE
74	57,97797297	0,367925269	0,456415674	0,80611883	0,420174335	0,996224111	0	FALSE
75	9,342778348	0,514303483	1,046777691	0,491320638	0,623199685	0,996224111	0	FALSE
76	69,76259628	0,722471996	0,796337064	0,907243966	0,364277799	0,996224111	0	FALSE
77	6,641402801	-2,447524576	1,257889813	-1,945738451	0,051686165	0,73959756	0	FALSE
78	109,6592178	1,076787816	0,901393145	1,194581766	0,232250477	0,996224111	0	FALSE
79	58,18303657	0,688916505	1,961823249	0,351161352	0,725467303	0,996224111	0	FALSE
80	81,29982741	0,317150607	0,503940992	0,62934076	0,529125992	0,996224111	0	FALSE
81	113,8059496	1,580124046	1,525568028	1,035761118	0,300313601	0,996224111	0	FALSE
82	20,69203704	-0,549779547	2,011208485	-0,27335781	0,784578181	0,996224111	0	FALSE
83	44,1929575	-0,202839273	1,345253778	-0,150781418	0,880148145	0,996224111	0	FALSE
84	81,359185	-0,412704269	0,491303133	-0,840019614	0,400897389	0,996224111	0	FALSE
85	37,15583256	0,727459477	2,190615828	0,332079896	0,73982893	0,996224111	6	FALSE
86	36,56538947	-0,242726914	0,53786562	-0,451277987	0,651789208	0,996224111	21	FALSE
87	31,35058295	0,304668267	1,421474744	0,214332522	0,830287758	0,996224111	0	FALSE
88	56,9039602	0,529229229	0,393443247	1,345122156	0,178585789	0,99239572	0	FALSE
89	2,322523491	0,410924715	1,108660904	0,370649595	0,710898538	0,996224111	6	FALSE
90	64,4381198	-0,72573882	1,752868359	-0,414029278	0,678852665	0,996224111	0	FALSE
91	108,7248523	-0,192733221	1,864517966	-0,103368927	0,917670173	0,996224111	0	FALSE
92	34,09753725	-2,91350268	2,023744236	-1,439659532	0,149963748	0,985872787	0	FALSE
93	1,333663652	1,801528703	1,645861462	1,094581011	0,273700239	0,996224111	11	FALSE
94	1983,77782	0,573314714	0,580998521	0,986774825	0,323753039	0,996224111	0	FALSE
95	785,6434719	-1,850417057	2,003085619	-0,923783307	0,355599148	0,996224111	0	FALSE
96	36,3031655	-0,422339775	0,727672739	-0,580397963	0,561646279	0,996224111	0	FALSE
97	116,1839654	1,349016571	1,599292229	0,843508489	0,39894412	0,996224111	0	FALSE
98	0,969907139	-1,477048996	1,594835043	-0,92614531	0,354370469	0,996224111	3	FALSE
99	37,00894871	-0,483209054	1,628461109	-0,296727414	0,766674626	0,996224111	0	FALSE
100	28,7478043	0,795103897	1,921176235	0,413863071	0,67897439	0,996224111	0	FALSE
101	9,219491325	-0,69706955	0,932886349	-0,747218084	0,45493193	0,996224111	0	FALSE
102	568,0811414	0,531037216	0,454967022	1,167199358	0,24312987	0,996224111	0	FALSE
103	46,42409032	-0,661807722	1,243661448	-0,532144599	0,59462585	0,996224111	0	FALSE
104	5,45184614	-2,612196922	1,178124078	-2,217251111	0,026605937	0,657109187	69	FALSE

ASV_nr	Phylum	Class	Order	Family	Genus	Species
105	Firmicutes	Clostridia	Clostridia UCG-014	NA	NA	NA
106	Firmicutes	Clostridia	Lachnospirales	Lachnospiraceae	Lachnospiraceae UCG-010	NA
107	Firmicutes	Clostridia	Lachnospirales	Lachnospiraceae	Lachnospira	pectinoschiza
108	Bacteroidota	Bacteroidia	Bacteroidales	Bacteroidaceae	Bacteroides	uniformis
109	Firmicutes	Clostridia	Lachnospirales	Lachnospiraceae	Lachnospiraceae UCG-004	NA
110	Firmicutes	Clostridia	Oscillospirales	Oscillospiraceae	Intestinimonas	NA
111	Firmicutes	Clostridia	Oscillospirales	Oscillospiraceae	NK4A214 group	NA
112	Firmicutes	Clostridia	Lachnospirales	Lachnospiraceae	Lachnospiraceae UCG-001	NA
113	Firmicutes	Bacilli	Lactobacillales	Streptococcaceae	Streptococcus	NA
114	Firmicutes	Clostridia	Oscillospirales	Ruminococcaceae	Incertae Sedis	NA
115	Firmicutes	Clostridia	Oscillospirales	Oscillospiraceae	Flavonifractor	plautii
116	Firmicutes	Clostridia	Oscillospirales	Ruminococcaceae	[Eubacterium] siraeum group	NA
117	Proteobacteria	Alphaproteobacteria	Rhodospirillales	NA	NA	NA
118	Firmicutes	Bacilli	Acholeplasmatales	Acholeplasmataceae	Anaeroplasma	NA
119	Cyanobacteria	Vampirivibrionia	Gastranaerophilales	NA	NA	NA
120	Firmicutes	Clostridia	Oscillospirales	Oscillospiraceae	UCG-005	NA
121	Bacteroidota	Bacteroidia	Bacteroidales	Bacteroidaceae	Bacteroides	uniformis
122	Bacteroidota	Bacteroidia	Bacteroidales	Bacteroidaceae	Bacteroides	clarus
123	Firmicutes	Clostridia	Oscillospirales	[Eubacterium] coprostanoligenes group	NA	NA
124	Firmicutes	Clostridia	Lachnospirales	Lachnospiraceae	Tyzzerella	NA
125	Firmicutes	Clostridia	Clostridia UCG-014	NA	NA	NA
126	Actinobacteriota	Actinobacteria	Bifidobacteriales	Bifidobacteriaceae	Bifidobacterium	NA
127	Firmicutes	Clostridia	Oscillospirales	Ruminococcaceae	Subdoligranulum	NA
128	Firmicutes	Clostridia	Oscillospirales	Oscillospiraceae	NK4A214 group	NA
129	Bacteroidota	Bacteroidia	Bacteroidales	Tannerellaceae	Parabacteroides	distasonis
130	Firmicutes	Clostridia	Lachnospirales	Lachnospiraceae	Lachnospira	NA
131	Euryarchaeota	Methanobacteria	Methanobacteriales	Methanobacteriaceae	Methanobrevibacter	NA
132	Bacteroidota	Bacteroidia	Bacteroidales	Bacteroidaceae	Bacteroides	ovatus
133	Firmicutes	Clostridia	Lachnospirales	Lachnospiraceae	Lachnospiraceae AC2044 group	NA
134	Firmicutes	Clostridia	Peptostreptococcales-Tissierellales	Anaerovoracaceae	Family XIII UCG-001	NA
135	Firmicutes	Clostridia	Oscillospirales	Ruminococcaceae	Incertae Sedis	NA
136	Firmicutes	Clostridia	Oscillospirales	Oscillospiraceae	Colidextribacter	NA
137	Firmicutes	Clostridia	Oscillospirales	Ruminococcaceae	CAG-352	NA
138	Firmicutes	Clostridia	Lachnospirales	Lachnospiraceae	Lachnoclostridium	NA
139	Patescibacteria	Saccharimonadia	Saccharimonadales	Saccharimonadaceae	TM7x	NA
140	Firmicutes	Clostridia	Oscillospirales	Ruminococcaceae	Incertae Sedis	NA

ASV_nr	DESeq_baseMean	DESeq_log2FoldChange	DESeq_lfcSE	DESeq_stat	DESeq_pvalue	DESeq_padj	ANCOM_W	ANCOM_detected_0.7
105	64,9955861	0,508358587	1,140566388	0,445707144	0,655808803	0,996224111	0	FALSE
106	24,88491392	-1,77269968	1,177051899	-1,506050567	0,132054193	0,967944373	0	FALSE
107	54,90028964	-0,231515481	0,936311015	-0,247263439	0,804704352	0,996224111	47	FALSE
108	722,1444081	0,350446866	0,685356284	0,511335308	0,609116286	0,996224111	0	FALSE
109	65,47484656	-1,091824137	0,717352488	-1,522019029	0,128004313	0,967944373	252	TRUE
110	44,97226562	0,153116891	0,500746228	0,305777423	0,759774119	0,996224111	21	FALSE
111	37,83665474	1,29935671	0,9091585	1,429186121	0,152950746	0,987227541	0	FALSE
112	19,20439306	-0,700864922	1,025170629	-0,683656849	0,494191878	0,996224111	0	FALSE
113	66,34084773	0,473359971	0,562025933	0,842238664	0,399654376	0,996224111	0	FALSE
114	29,35177739	0,739247326	0,958046803	0,77161922	0,440339987	0,996224111	11	FALSE
115	36,26560312	-0,305172482	0,802137344	-0,380449164	0,703612026	0,996224111	0	FALSE
116	72,26775394	-0,09575622	0,958898374	-0,099860655	0,920454953	0,996224111	0	FALSE
117	53,40129994	-1,99556077	1,501310675	-1,329212403	0,183777904	0,99239572	15	FALSE
118	94,20946332	-6,942645785	2,136096926	-3,25015485	0,001153422	0,111830545	2	FALSE
119	48,92775064	-2,130759356	0,986526855	-2,159859455	0,030783551	0,683010046	1	FALSE
120	34,89129189	-0,734246152	1,066457951	-0,688490485	0,49114396	0,996224111	0	FALSE
121	622,9764759	0,262709328	0,708237823	0,370933774	0,710686859	0,996224111	0	FALSE
122	43,68670684	0,251339274	1,922442157	0,130739577	0,895981323	0,996224111	0	FALSE
123	10,50996772	-0,376256413	1,512136673	-0,248824342	0,803496661	0,996224111	0	FALSE
124	30,29361968	1,500344826	1,349720507	1,111596674	0,266311603	0,996224111	11	FALSE
125	35,61704991	0,598916411	2,030005143	0,295031968	0,767969459	0,996224111	0	FALSE
126	191,5227237	-0,193143142	0,717225339	-0,269292133	0,787704885	0,996224111	0	FALSE
127	17,49326539	1,446617899	1,607239614	0,900063616	0,368086397	0,996224111	0	FALSE
128	38,54215967	-3,791793623	1,487536276	-2,549042793	0,010801904	0,383467609	0	FALSE
129	32,05903859	1,231592344	1,535819697	0,801912064	0,422603827	0,996224111	135	FALSE
130	63,44829993	0,849289955	1,025360068	0,828284601	0,407509342	0,996224111	7	FALSE
131	95,47310806	1,549896898	0,797913991	1,942436045	0,052084335	0,73959756	0	FALSE
132	39,32949799	2,833903546	1,875392224	1,511099123	0,130763198	0,967944373	23	FALSE
133	46,24804713	-0,985996686	1,273679167	-0,774132695	0,438852325	0,996224111	0	FALSE
134	19,5018172	0,073539754	0,810432032	0,09074142	0,927698058	0,997509535	0	FALSE
135	25,28812878	-0,216863427	0,923868111	-0,234734184	0,814415054	0,996224111	0	FALSE
136	14,37477674	-0,090882481	0,835219797	-0,108812652	0,913351089	0,996224111	2	FALSE
137	320,2298575	1,578590244	1,779590615	0,887052467	0,375050654	0,996224111	0	FALSE
138	39,99823825	-1,577671904	0,742615734	-2,1244795	0,033630069	0,694215217	245	FALSE
139	0,826594384	-0,011327332	1,501710624	-0,007542953	0,993981652	0,997979415	11	FALSE
140	24,15500379	0,907275183	0,581910611	1,559131534	0,118965251	0,967944373	101	FALSE

ASV_nr	Phylum	Class	Order	Family	Genus	Species
141	Firmicutes	Clostridia	Christensenellales	Christensenellaceae	Christensenellaceae R-7 group	NA
142	Firmicutes	Clostridia	Oscillospirales	Oscillospiraceae	NK4A214 group	NA
143	Firmicutes	Clostridia	Clostridiales	Clostridiaceae	Clostridium sensu stricto 1	NA
144	Firmicutes	Clostridia	Oscillospirales	Oscillospiraceae	NK4A214 group	NA
145	Bacteroidota	Bacteroidia	Bacteroidales	Rikenellaceae	Alistipes	ihumii
146	Firmicutes	Bacilli	Erysipelotrichales	Erysipelatoclostridiaceae	Erysipelatoclostridium	ramosum
147	Actinobacteriota	Coriobacteriia	Coriobacteriales	Eggerthellaceae	Slackia	isoflavoniconvertens
148	Firmicutes	Clostridia	Oscillospirales	Oscillospiraceae	NK4A214 group	NA
149	Firmicutes	Clostridia	Oscillospirales	Oscillospiraceae	UCG-002	NA
150	Firmicutes	Clostridia	Lachnospirales	Lachnospiraceae	NA	NA
151	Firmicutes	Clostridia	Christensenellales	Christensenellaceae	Christensenellaceae R-7 group	NA
152	Firmicutes	Bacilli	Erysipelotrichales	Erysipelotrichaceae	NA	NA
153	Firmicutes	Bacilli	Erysipelotrichales	Erysipelatoclostridiaceae	Erysipelatoclostridium	NA
154	Firmicutes	Clostridia	Oscillospirales	Ruminococcaceae	Subdoligranulum	NA
155	Proteobacteria	Gammaproteobacteria	Enterobacterales	Enterobacteriaceae	Escherichia-Shigella	NA
156	Firmicutes	Clostridia	Oscillospirales	Ruminococcaceae	Negativibacillus	NA
157	Firmicutes	Bacilli	Lactobacillales	Streptococcaceae	Lactococcus	NA
158	Firmicutes	Clostridia	Lachnospirales	Lachnospiraceae	GCA-900066575	NA
159	Actinobacteriota	Coriobacteriia	Coriobacteriales	Eggerthellaceae	Eggerthella	lenta
160	Bacteroidota	Bacteroidia	Bacteroidales	Rikenellaceae	Alistipes	putredinis
161	Firmicutes	Clostridia	Lachnospirales	Lachnospiraceae	Lachnospiraceae UCG-004	NA
162	Firmicutes	Negativicutes	Veillonellales-Selenomonadales	Veillonellaceae	Veillonella	NA
163	Firmicutes	Clostridia	Lachnospirales	Lachnospiraceae	Lachnoclostridium	NA
164	Firmicutes	Clostridia	Christensenellales	Christensenellaceae	NA	NA
165	Firmicutes	Clostridia	Oscillospirales	Oscillospiraceae	Colidextribacter	massiliensis
166	Firmicutes	Clostridia	Lachnospirales	Lachnospiraceae	NA	NA
167	Firmicutes	Clostridia	Oscillospirales	Oscillospiraceae	UCG-002	NA
168	Firmicutes	Clostridia	Oscillospirales	Ruminococcaceae	Subdoligranulum	NA
169	Firmicutes	Clostridia	Peptostreptococcales-Tissierellales	Anaerovoracaceae	Family XIII UCG-001	NA
170	Firmicutes	Clostridia	Clostridia vadinBB60 group	NA	NA	NA
171	Firmicutes	Clostridia	Christensenellales	Christensenellaceae	Christensenellaceae R-7 group	NA
172	Firmicutes	Clostridia	Peptostreptococcales-Tissierellales	Anaerovoracaceae	Family XIII AD3011 group	NA
173	Firmicutes	Clostridia	Peptostreptococcales-Tissierellales	Peptostreptococcaceae	Intestinibacter	bartlettii
174	Proteobacteria	Alphaproteobacteria	Rhodospirillales	NA	NA	NA
175	Proteobacteria	Alphaproteobacteria	Rhodospirillales	NA	NA	NA
176	Firmicutes	Clostridia	Lachnospirales	Lachnospiraceae	Anaerostipes	hadrus

ASV_nr	DESeq_baseMean	DESeq_log2FoldChange	DESeq_lfcSE	DESeq_stat	DESeq_pvalue	DESeq_padj	ANCOM_W	ANCOM_detected_0.7
141	23,85221454	1,60919409	0,896032906	1,795909591	0,072508897	0,887608909	279	TRUE
142	15,08097764	-3,024928889	1,815938711	-1,66576596	0,095760039	0,967944373	6	FALSE
143	47,75144666	0,255068024	0,999846681	0,255107137	0,798640352	0,996224111	0	FALSE
144	31,35979455	0,930741026	1,197752002	0,777073237	0,437115562	0,996224111	208	FALSE
145	15,73452395	0,564728311	0,993587631	0,568372928	0,569781769	0,996224111	0	FALSE
146	4,568936661	1,349880555	1,790301067	0,753996398	0,450851385	0,996224111	13	FALSE
147	4,287745954	0,192745114	1,391667894	0,138499361	0,889845775	0,996224111	5	FALSE
148	34,51256235	0,937410184	1,347394315	0,69572075	0,486603728	0,996224111	0	FALSE
149	453,8732145	0,646119224	0,733839865	0,880463511	0,378608264	0,996224111	0	FALSE
150	25,70160899	-2,587798832	1,877472869	-1,378341533	0,168097866	0,991381803	0	FALSE
151	12,13633874	0,269269368	1,629224369	0,165274577	0,868727868	0,996224111	0	FALSE
152	12,04360918	0,04244221	1,05302802	0,04030492	0,967850032	0,997979415	0	FALSE
153	2,207303568	1,068558458	1,27638616	0,837174902	0,402494263	0,996224111	26	FALSE
154	1321,420511	-0,414556509	0,45623018	-0,90865648	0,363531483	0,996224111	12	FALSE
155	401,6256536	-0,94367389	0,773824123	-1,21949402	0,222656744	0,996224111	0	FALSE
156	20,87404095	0,842946726	0,880771272	0,9570552	0,338539394	0,996224111	127	FALSE
157	10,49356662	-0,382021323	1,07862689	-0,354173743	0,72320868	0,996224111	8	FALSE
158	14,31819658	-3,50364787	0,798706216	-4,386654068	1,15108E-05	0,002043161	274	TRUE
159	5,791558886	1,269897516	1,590444323	0,798454556	0,424606755	0,996224111	0	FALSE
160	519,463526	-0,009844784	0,477467378	-0,020618757	0,983549778	0,997979415	0	FALSE
161	36,02045171	-0,184456862	0,845708309	-0,218109318	0,827343938	0,996224111	0	FALSE
162	29,19841145	-0,404820675	1,142981745	-0,354179475	7,23E-01	0,996224111	0	FALSE
163	16,78049548	1,412225039	1,572022696	0,898349014	0,368999509	0,996224111	11	FALSE
164	37,46075277	0,257024696	1,653001643	0,15548968	0,876435289	0,996224111	0	FALSE
165	8,414380134	-1,109229666	1,051085518	-1,055318189	0,291279825	0,996224111	0	FALSE
166	18,13996477	-4,267906308	1,533043413	-2,783943542	0,005370238	0,238304316	0	FALSE
167	468,1732533	-0,706019109	0,519702094	-1,35850734	0,174302755	0,99239572	17	FALSE
168	16,58094157	1,313081936	2,029855784	0,646884348	0,517706796	0,996224111	0	FALSE
169	12,33346865	-1,743978467	1,35276652	-1,289193989	0,197330654	0,996224111	0	FALSE
170	39,14906159	0,510789946	0,95074208	0,537253959	0,591092204	0,996224111	74	FALSE
171	9,433284069	-0,551521325	1,193747967	-0,46200818	0,644075455	0,996224111	0	FALSE
172	12,44030506	-1,620979719	0,85094931	-1,904907495	0,056792097	0,770809583	73	FALSE
173	36,37880312	-1,069970676	0,808386862	-1,323587414	0,185640107	0,99239572	0	FALSE
174	35,20147084	-2,309676463	1,797337266	-1,285054568	0,198773216	0,996224111	0	FALSE
175	15,36537526	0,070819437	1,616363773	0,043814046	0,965052631	0,997979415	0	FALSE
176	24,6009874	0,079704884	0,698151634	0,114165576	0,90910654	0,996224111	0	FALSE

ASV_nr	Phylum	Class	Order	Family	Genus	Species
177	Bacteroidota	Bacteroidia	Bacteroidales	Barnesiellaceae	NA	NA
178	Firmicutes	Clostridia	Lachnospirales	Lachnospiraceae	Lachnoclostridium	NA
179	Firmicutes	Clostridia	Lachnospirales	Lachnospiraceae	Lachnotalea	NA
180	Firmicutes	Clostridia	Monoglobales	Monoglobaceae	Monoglobus	pectinilyticus
181	Firmicutes	Clostridia	Christensenellales	Christensenellaceae	Christensenellaceae R-7 group	NA
182	Firmicutes	Clostridia	Lachnospirales	Lachnospiraceae	Agathobacter	NA
183	Firmicutes	Clostridia	Lachnospirales	Lachnospiraceae	NA	NA
184	Firmicutes	Clostridia	Oscillospirales	Oscillospiraceae	UCG-005	NA
185	Firmicutes	Clostridia	Lachnospirales	Lachnospiraceae	Blautia	obeum
186	Firmicutes	Clostridia	Lachnospirales	Lachnospiraceae	Lachnospiraceae UCG-003	NA
187	Firmicutes	Clostridia	Lachnospirales	Lachnospiraceae	[Eubacterium] xylanophilum group	NA
188	Firmicutes	Clostridia	Oscillospirales	Ruminococcaceae	Negativibacillus	NA
189	Firmicutes	Clostridia	Lachnospirales	Lachnospiraceae	[Eubacterium] hallii group	NA
190	Firmicutes	Clostridia	Oscillospirales	Ruminococcaceae	NA	NA
191	Bacteroidota	Bacteroidia	Bacteroidales	Rikenellaceae	Alistipes	NA
192	Firmicutes	Clostridia	Oscillospirales	Oscillospiraceae	Colidextribacter	NA
193	Bacteroidota	Bacteroidia	Bacteroidales	Rikenellaceae	Alistipes	NA
194	Firmicutes	Clostridia	Oscillospirales	Oscillospiraceae	Colidextribacter	NA
195	Firmicutes	Clostridia	Oscillospirales	UCG-010	NA	NA
196	Firmicutes	Clostridia	Clostridiales	Clostridiaceae	Clostridium sensu stricto 1	NA
197	Bacteroidota	Bacteroidia	Bacteroidales	Barnesiellaceae	Coprobacter	fastidiosus
198	Firmicutes	Clostridia	Christensenellales	Christensenellaceae	Christensenellaceae R-7 group	NA
199	Bacteroidota	Bacteroidia	Bacteroidales	Tannerellaceae	Parabacteroides	merdae
200	Firmicutes	Bacilli	RF39	NA	NA	NA
201	Firmicutes	Clostridia	Oscillospirales	Ruminococcaceae	DTU089	NA
202	Firmicutes	Clostridia	Lachnospirales	Lachnospiraceae	NA	NA
203	Firmicutes	Clostridia	Oscillospirales	Ruminococcaceae	Ruminococcus	bromii
204	Firmicutes	Bacilli	Erysipelotrichales	Erysipelotrichaceae	Holdemanella	NA
205	Bacteroidota	Bacteroidia	Bacteroidales	Rikenellaceae	Alistipes	indistinctus
206	Firmicutes	Clostridia	Oscillospirales	Ruminococcaceae	Ruminococcus	bicirculans
207	Firmicutes	Clostridia	Clostridia UCG-014	NA	NA	NA
208	Firmicutes	Clostridia	Oscillospirales	Oscillospiraceae	Colidextribacter	NA
209	Firmicutes	Clostridia	NA	NA	NA	NA
210	Actinobacteriota	Actinobacteria	Bifidobacteriales	Bifidobacteriaceae	Bifidobacterium	NA
211	Firmicutes	Clostridia	Peptostreptococcales-Tissierellales	Anaerovoracaceae	Family XIII AD3011 group	NA
212	Firmicutes	Clostridia	Christensenellales	Christensenellaceae	NA	NA

ASV_nr	DESeq_baseMean	DESeq_log2FoldChange	DESeq_lfcSE	DESeq_stat	DESeq_pvalue	DESeq_padj	ANCOM_W	ANCOM_detected_0.7
177	30,39086102	-0,705366844	1,609628988	-0,438217036	0,66122896	0,996224111	0	FALSE
178	14,82084622	-0,256771224	0,981559059	-0,261595287	0,793633478	0,996224111	0	FALSE
179	14,61278321	-2,306709369	1,048223916	-2,20058838	0,027765177	0,657109187	241	FALSE
180	3,525322572	1,438797195	1,560967444	0,921734275	0,356667198	0,996224111	10	FALSE
181	25,05289904	1,152712592	0,921114504	1,251432462	0,21077674	0,996224111	43	FALSE
182	387,6093075	-0,17156057	0,792867382	-0,216379906	0,828691628	0,996224111	0	FALSE
183	15,01205369	-5,425699995	1,682427402	-3,224923696	0,001260062	0,111830545	37	FALSE
184	39,8568889	1,049172457	1,553879929	0,675195321	0,499551679	0,996224111	7	FALSE
185	59,15411718	-0,241648399	0,675494969	-0,357735305	0,72054141	0,996224111	0	FALSE
186	38,69891781	-2,170817552	1,851790276	-1,172280458	0,241084475	0,996224111	0	FALSE
187	20,78856673	-0,467923948	1,24988255	-0,374374335	0,708125835	0,996224111	0	FALSE
188	9,705745067	0,058021325	1,977630006	0,029338817	0,976594368	0,997979415	8	FALSE
189	5,216401976	0,694812652	1,089550334	0,637705878	0,523665159	0,996224111	0	FALSE
190	15,15524661	0,004464226	0,619201164	0,007209653	0,994247579	0,997979415	0	FALSE
191	14,44596126	-0,795255494	1,016295985	-0,782503824	0,433918539	0,996224111	0	FALSE
192	12,35414377	0,783827504	1,563804754	0,501231053	0,616208522	0,996224111	23	FALSE
193	16,34243202	1,522517457	1,239084716	1,228743634	0,219167941	0,996224111	0	FALSE
194	16,07329775	-0,058602015	1,786031098	-0,032811307	0,973825061	0,997979415	0	FALSE
195	22,25418297	-0,672340085	1,01952616	-0,659463298	0,509598307	0,996224111	0	FALSE
196	34,376241	-2,424822455	1,472654818	-1,646565391	0,099647409	0,967944373	26	FALSE
197	18,82625993	-0,52477695	1,017266652	-0,515869609	0,605945481	0,996224111	0	FALSE
198	9,144191108	-0,543347814	1,024830185	-0,530183265	0,595984873	0,996224111	0	FALSE
199	425,5829541	0,002964233	0,653076586	0,004538875	0,996378514	0,997979415	0	FALSE
200	16,97681414	-1,672833671	1,893735434	-0,883351308	0,37704649	0,996224111	0	FALSE
201	7,967212743	0,258467511	0,861615509	0,299980105	0,764192331	0,996224111	12	FALSE
202	13,4443032	-3,204144997	1,085022239	-2,953068502	0,003146321	0,223388816	91	FALSE
203	359,3723855	-0,027362781	0,682157914	-0,040112092	0,968003761	0,997979415	0	FALSE
204	61,16670071	0,520462564	1,971084365	0,264048852	0,791742276	0,996224111	0	FALSE
205	11,1745775	-0,173917011	0,918852036	-0,189276406	0,849876187	0,996224111	0	FALSE
206	368,2717285	-0,755480795	0,821628591	-0,919491852	0,357838365	0,996224111	0	FALSE
207	15,73023371	-1,101659744	1,656110263	-0,665209176	0,505916712	0,996224111	0	FALSE
208	16,59755392	-1,265603235	0,785664625	-1,610869567	0,107208154	0,967944373	248	TRUE
209	7,594492824	-0,713551027	0,664583117	-1,073682146	0,282965166	0,996224111	0	FALSE
210	59,94467078	-0,374145021	1,840053406	-0,203333784	0,838874152	0,996224111	0	FALSE
211	5,254142166	1,16713359	1,618160299	0,721271923	0,470742228	0,996224111	15	FALSE
212	15,73124011	-1,426118457	1,677785452	-0,85000049	0,395324814	0,996224111	1	FALSE

ASV_nr	Phylum	Class	Order	Family	Genus	Species
213	Firmicutes	Clostridia	Oscillospirales	Oscillospiraceae	Oscillibacter	NA
214	Firmicutes	Clostridia	Oscillospirales	Oscillospiraceae	UCG-003	NA
215	Firmicutes	Clostridia	Oscillospirales	Oscillospiraceae	Oscillospira	NA
216	Firmicutes	Clostridia	Lachnospirales	Lachnospiraceae	Blautia	faecis
217	Firmicutes	Clostridia	Oscillospirales	UCG-010	NA	NA
218	Firmicutes	Clostridia	Christensenellales	Christensenellaceae	Christensenellaceae R-7 group	NA
219	Bacteroidota	Bacteroidia	Bacteroidales	Bacteroidaceae	Bacteroides	NA
220	Firmicutes	Clostridia	Lachnospirales	Lachnospiraceae	Coprococcus	comes
221	Firmicutes	Clostridia	Christensenellales	Christensenellaceae	Christensenellaceae R-7 group	NA
222	Firmicutes	Clostridia	Christensenellales	Christensenellaceae	Christensenellaceae R-7 group	NA
223	Firmicutes	Clostridia	Christensenellales	Christensenellaceae	Christensenellaceae R-7 group	NA
224	Firmicutes	Clostridia	Oscillospirales	UCG-010	NA	NA
225	Firmicutes	Clostridia	Oscillospirales	[Eubacterium] coprostanoligenes group	NA	NA
226	Bacteroidota	Bacteroidia	Bacteroidales	Bacteroidaceae	Bacteroides	NA
227	Firmicutes	Negativicutes	Veillonellales-Selenomonadales	Veillonellaceae	Veillonella	NA
228	Firmicutes	Clostridia	Clostridia UCG-014	NA	NA	NA
229	Bacteroidota	Bacteroidia	Bacteroidales	Rikenellaceae	Alistipes	NA
230	Verrucomicrobiota	Lentisphaeria	Victivallales	Victivallaceae	Victivallis	NA
231	Bacteroidota	Bacteroidia	Bacteroidales	Marinifilaceae	Butyricimonas	paravirosa
232	Bacteroidota	Bacteroidia	Bacteroidales	Bacteroidaceae	Bacteroides	caccae
233	Bacteroidota	Bacteroidia	Bacteroidales	Bacteroidaceae	Bacteroides	NA
234	Firmicutes	Clostridia	Christensenellales	Christensenellaceae	Christensenellaceae R-7 group	NA
235	Firmicutes	Negativicutes	Veillonellales-Selenomonadales	Veillonellaceae	Veillonella	NA
236	Firmicutes	Clostridia	Oscillospirales	Oscillospiraceae	Intestinimonas	massiliensis
237	Firmicutes	Clostridia	Lachnospirales	Lachnospiraceae	Blautia	NA
238	Firmicutes	Clostridia	Oscillospirales	UCG-010	NA	NA
239	Firmicutes	Clostridia	Lachnospirales	Lachnospiraceae	Frisingicoccus	NA
240	Firmicutes	Clostridia	Oscillospirales	Oscillospiraceae	UCG-005	NA
241	Firmicutes	Clostridia	Lachnospirales	Lachnospiraceae	Howardella	ureilytica
242	Firmicutes	Clostridia	Lachnospirales	Lachnospiraceae	Coprococcus	eutactus
243	Firmicutes	Clostridia	Christensenellales	Christensenellaceae	Christensenellaceae R-7 group	NA
244	Firmicutes	Clostridia	Lachnospirales	Lachnospiraceae	[Ruminococcus] torques group	NA
245	Firmicutes	Clostridia	Clostridia vadinBB60 group	NA	NA	NA
246	Firmicutes	Clostridia	Oscillospirales	Ruminococcaceae	NA	NA
247	Proteobacteria	Gammaproteobacteria	Burkholderiales	Oxalobacteraceae	Oxalobacter	formigenes
248	Bacteroidota	Bacteroidia	Bacteroidales	Bacteroidaceae	Bacteroides	NA

ASV_nr	DESeq_baseMean	DESeq_log2FoldChange	DESeq_lfcSE	DESeq_stat	DESeq_pvalue	DESeq_padj	ANCOM_W	ANCOM_detected_0.7
213	8,103682305	-0,08073931	1,28906249	-0,062634132	0,95005785	0,997979415	0	FALSE
214	15,75309999	-0,306660618	0,879380454	-0,348723487	0,72729691	0,996224111	0	FALSE
215	15,72669519	0,380549697	0,805696253	0,472324025	0,636695525	0,996224111	0	FALSE
216	139,6097156	-0,674140165	0,491685615	-1,371079699	0,170350113	0,991381803	116	FALSE
217	17,62327869	-0,390219242	1,426268282	-0,273594559	0,784396215	0,996224111	0	FALSE
218	9,87158793	0,770558105	0,873809895	0,881837239	0,377864831	0,996224111	31	FALSE
219	257,6368766	0,751351122	0,959428021	0,783124013	0,433554291	0,996224111	0	FALSE
220	118,8665894	-0,374611761	0,437807622	-0,855653813	0,392189295	0,996224111	159	FALSE
221	10,39760337	-0,481854015	1,320193038	-0,364987544	0,715120704	0,996224111	0	FALSE
222	10,5093864	-0,022548035	1,240599889	-0,018175107	0,985499161	0,997979415	0	FALSE
223	12,5481157	0,22891631	1,130675169	0,202459837	0,839557254	0,996224111	0	FALSE
224	8,488403799	-0,653502381	1,427445254	-0,457812571	0,647087104	0,996224111	0	FALSE
225	9,081324643	0,313361934	1,08592198	0,288567632	0,772912268	0,996224111	0	FALSE
226	349,9016475	0,289348085	0,432984164	0,668264823	0,503964564	0,996224111	0	FALSE
227	18,67047417	-0,948685065	1,520902815	-0,623764422	0,532782308	0,996224111	0	FALSE
228	15,01593812	-0,612907059	1,569124995	-0,390604356	0,696089705	0,996224111	0	FALSE
229	304,0313773	0,095673919	0,708191839	0,135096048	0,892535935	0,996224111	0	FALSE
230	13,90272394	-0,637288897	0,868523772	-0,733761029	0,463094396	0,996224111	0	FALSE
231	12,62475379	0,33196946	1,383677135	0,239918295	0,810393597	0,996224111	0	FALSE
232	312,2474805	0,850148238	0,619536187	1,372233382	0,169990796	0,991381803	0	FALSE
233	301,5388324	0,630552092	1,206918033	0,522448149	0,601358339	0,996224111	0	FALSE
234	12,17208305	0,6165696	0,982337819	0,627655363	0,530229733	0,996224111	55	FALSE
235	21,43387134	-0,083629137	1,230085136	-0,067986462	0,945796411	0,997979415	6	FALSE
236	8,870084739	-0,416532114	1,349736115	-0,30860263	0,75762382	0,996224111	0	FALSE
237	136,5310259	-0,730841977	0,298411215	-2,449110289	0,014320959	0,462176404	53	FALSE
238	19,10721533	-0,520283864	2,02647156	-0,256743729	0,797376612	0,996224111	0	FALSE
239	10,19292095	-1,132942428	1,609625227	-0,703854791	0,481523208	0,996224111	0	FALSE
240	291,4195229	0,604308046	0,547068502	1,104629575	0,269320131	0,996224111	0	FALSE
241	4,798276878	0,307204418	1,19667655	0,256714664	0,797399051	0,996224111	0	FALSE
242	269,3078466	-0,519878584	1,152186929	-0,451210277	0,651838003	0,996224111	0	FALSE
243	10,9264737	0,202545876	1,396709521	0,145016464	0,884697873	0,996224111	3	FALSE
244	15,92138314	0,221523644	1,104361907	0,200589718	0,841019398	0,996224111	8	FALSE
245	10,6533639	-2,070536539	1,695605074	-1,22111957	0,222040751	0,996224111	0	FALSE
246	14,07383547	-0,007211259	0,881247523	-0,008183011	0,993470974	0,997979415	37	FALSE
247	5,298515787	0,435309884	1,023530864	0,425302157	0,670616421	0,996224111	11	FALSE
248	326,7018707	-0,017671369	0,76032856	-0,023241753	0,981457433	0,997979415	0	FALSE

ASV_nr	Phylum	Class	Order	Family	Genus	Species
249	Firmicutes	Clostridia	Christensenellales	Christensenellaceae	Christensenellaceae R-7 group	NA
250	Firmicutes	Clostridia	Oscillospirales	UCG-010	NA	NA
251	Firmicutes	Clostridia	Oscillospirales	UCG-010	NA	NA
252	Firmicutes	Clostridia	Monoglobales	Monoglobaceae	Monoglobus	NA
253	Firmicutes	Bacilli	RF39	NA	NA	NA
254	Firmicutes	Clostridia	Lachnospirales	Lachnospiraceae	NA	NA
255	Bacteroidota	Bacteroidia	Bacteroidales	Bacteroidaceae	Bacteroides	massiliensis
256	Firmicutes	Clostridia	Peptostreptococcales-Tissierellales	Anaerovoracaceae	Family XIII UCG-001	NA
257	Firmicutes	Bacilli	Erysipelotrichales	Erysipelatoclostridiaceae	Asteroleplasma	NA
258	Bacteroidota	Bacteroidia	Bacteroidales	Marinifilaceae	Butyricimonas	NA
259	Firmicutes	Clostridia	Lachnospirales	Lachnospiraceae	Roseburia	inulinivorans
260	Bacteroidota	Bacteroidia	Bacteroidales	Marinifilaceae	Butyricimonas	faecihominis
261	Firmicutes	Clostridia	Lachnospirales	Lachnospiraceae	NA	NA
262	Bacteroidota	Bacteroidia	Bacteroidales	Bacteroidaceae	Bacteroides	NA
263	Firmicutes	Clostridia	Clostridia vadinBB60 group	NA	NA	NA
264	Firmicutes	Clostridia	Oscillospirales	[Eubacterium] coprostanoligenes group	NA	NA
265	Firmicutes	Clostridia	Oscillospirales	Oscillospiraceae	UCG-005	NA
266	Firmicutes	Clostridia	Hungateiclostridiaceae	Ruminiclostridium	NA	NA
267	Firmicutes	Clostridia	Oscillospirales	Ruminococcaceae	Faecalibacterium	NA
268	Firmicutes	Clostridia	Oscillospirales	Ruminococcaceae	[Eubacterium] siraeum group	NA
269	Bacteroidota	Bacteroidia	NA	NA	NA	NA
270	Firmicutes	Clostridia	Clostridia UCG-014	NA	NA	NA
271	Firmicutes	Clostridia	Oscillospirales	Oscillospiraceae	Oscillibacter	NA
272	Firmicutes	Clostridia	Oscillospirales	Oscillospiraceae	UCG-005	NA
273	Actinobacteriota	Coriobacteriia	Coriobacteriales	Eggerthellaceae	NA	NA
274	Firmicutes	Clostridia	Oscillospirales	UCG-010	NA	NA
275	Firmicutes	Clostridia	Christensenellales	Christensenellaceae	Christensenellaceae R-7 group	NA
276	Firmicutes	Clostridia	Clostridia vadinBB60 group	NA	NA	NA
277	Firmicutes	Clostridia	Oscillospirales	Oscillospiraceae	UCG-002	NA
278	Firmicutes	Clostridia	Oscillospirales	Oscillospiraceae	Oscillibacter	NA
279	Firmicutes	Clostridia	Oscillospirales	Oscillospiraceae	UCG-003	NA
280	Firmicutes	Clostridia	Oscillospirales	Oscillospiraceae	NK4A214 group	NA
281	Firmicutes	Clostridia	Oscillospirales	Ruminococcaceae	Incertae Sedis	NA
282	Firmicutes	Clostridia	Lachnospirales	Lachnospiraceae	NA	NA
283	Firmicutes	Clostridia	Lachnospirales	Lachnospiraceae	Lachnospiraceae UCG-010	NA
284	Firmicutes	Clostridia	Oscillospirales	Butyricicoccaceae	Butyricicoccus	NA

ASV_nr	DESeq_baseMean	DESeq_log2FoldChange	DESeq_lfcSE	DESeq_stat	DESeq_pvalue	DESeq_padj	ANCOM_W	ANCOM_detected_0.7
249	7,722483542	-1,03424357	1,786673198	-0,578865553	0,562679895	0,996224111	0	FALSE
250	7,590051773	0,319594406	1,226097038	0,260659961	0,794354746	0,996224111	0	FALSE
251	9,505821676	1,496064016	0,955593693	1,565585904	0,117445592	0,967944373	225	FALSE
252	114,8357243	0,031469394	0,399276009	0,07881614	0,937178866	0,997979415	0	FALSE
253	8,813833274	0,226403018	1,612416655	0,14041223	0,888334296	0,996224111	0	FALSE
254	12,08963279	-1,311170532	1,442463339	-0,908980143	0,363360608	0,996224111	0	FALSE
255	243,9882944	-0,597395047	1,531308646	-0,390120599	0,696447371	0,996224111	0	FALSE
256	2,633399208	-0,766501346	1,670156408	-0,458939859	0,646277355	0,996224111	0	FALSE
257	16,52722983	-1,926548169	1,629804543	-1,182073137	0,237176682	0,996224111	2	FALSE
258	7,540663291	-2,838011881	1,772824071	-1,600842367	0,109411837	0,967944373	1	FALSE
259	207,6052266	-1,15737132	0,594918156	-1,945429482	0,051723309	0,73959756	258	TRUE
260	11,95623031	-2,252711028	1,287061899	-1,75027404	0,080071038	0,920932712	34	FALSE
261	7,07283801	-1,743651763	1,201051705	-1,451770773	0,146565353	0,985872787	0	FALSE
262	208,5484506	0,20039022	0,74035862	0,270666424	0,786647603	0,996224111	0	FALSE
263	13,65066228	-1,04981463	1,262917055	-0,831261741	0,405825783	0,996224111	0	FALSE
264	199,1207095	1,667851325	1,359410334	1,226893222	0,219862726	0,996224111	133	FALSE
265	1,835081409	-1,376003685	1,695848096	-0,8113956	0,417138525	0,996224111	1	FALSE
266	8,413466533	2,031473622	1,404727356	1,446169332	0,148129713	0,985872787	142	FALSE
267	814,8295991	0,072691081	0,320020084	0,227145371	0,820310705	0,996224111	0	FALSE
268	232,6915401	-0,206007655	0,617949711	-0,333372849	0,738852856	0,996224111	0	FALSE
269	10,22205018	-0,576817176	1,925452373	-0,299574886	0,764501443	0,996224111	0	FALSE
270	332,662789	1,780159136	1,35001996	1,318616901	0,18729722	0,99239572	0	FALSE
271	7,874726759	0,970341579	1,617885442	0,599759139	0,548666769	0,996224111	13	FALSE
272	7,619509547	-2,060920444	1,381015606	-1,492322343	0,135614662	0,967944373	2	FALSE
273	16,14126058	0,439707223	0,676562448	0,649913727	0,51574795	0,996224111	3	FALSE
274	14,31025291	-0,380803617	1,525051751	-0,249698816	0,802820274	0,996224111	0	FALSE
275	1,982658245	1,949806462	1,604092449	1,215520005	0,224167823	0,996224111	28	FALSE
276	7,873550461	-1,979647769	1,732911711	-1,142382359	0,253295121	0,996224111	0	FALSE
277	184,7376594	0,311932358	0,545190965	0,572152472	0,567218683	0,996224111	0	FALSE
278	2,914634678	-1,461831693	1,639535154	-0,89161351	0,372600126	0,996224111	0	FALSE
279	10,75108582	-1,800083611	1,957187796	-0,919729632	0,357714064	0,996224111	0	FALSE
280	5,232029518	0,491907661	1,777762893	0,276700376	0,782010183	0,996224111	29	FALSE
281	95,20829878	-0,374242493	0,498315524	-0,751015119	0,452643557	0,996224111	0	FALSE
282	7,187519274	0,422649572	1,676058836	0,252168697	0,800910673	0,996224111	9	FALSE
283	5,077644476	-1,156757715	1,866247818	-0,619830713	0,535369246	0,996224111	0	FALSE
284	80,51739903	-0,921538631	0,590542257	-1,560495664	0,118642794	0,967944373	22	FALSE

ASV_nr	Phylum	Class	Order	Family	Genus	Species
285	Firmicutes	Clostridia	Oscillospirales	Oscillospiraceae	Oscillibacter	NA
286	Firmicutes	Bacilli	Erysipelotrichales	Erysipelotrichaceae	Holdemanella	biformis
287	Firmicutes	Bacilli	Izemoplasmatales	NA	NA	NA
288	Firmicutes	Clostridia	Oscillospirales	UCG-010	NA	NA
289	Firmicutes	Clostridia	Oscillospirales	NA	NA	NA
290	Firmicutes	Clostridia	Oscillospirales	Ruminococcaceae	Faecalibacterium	NA
291	Firmicutes	Clostridia	Oscillospirales	Ruminococcaceae	Angelakisella	NA
292	Firmicutes	Clostridia	Christensenellales	Christensenellaceae	NA	NA
293	Firmicutes	Clostridia	Lachnospirales	Lachnospiraceae	Agathobacter	NA
294	Firmicutes	Clostridia	Lachnospirales	Lachnospiraceae	Roseburia	intestinalis
295	Firmicutes	Clostridia	Oscillospirales	Oscillospiraceae	Intestinimonas	butyriciproducens
296	Firmicutes	Clostridia	Peptostreptococcales-Tissierellales	Anaerovoracaceae	Family XIII AD3011 group	NA
297	Firmicutes	Clostridia	Oscillospirales	[Eubacterium] coprostanoligenes group	NA	NA
298	Firmicutes	Clostridia	NA	NA	NA	NA
299	Firmicutes	Clostridia	Oscillospirales	Oscillospiraceae	Intestinimonas	NA
300	Firmicutes	Clostridia	Lachnospirales	Lachnospiraceae	NA	NA
301	Proteobacteria	Gammaproteobacteria	Burkholderiales	Sutterellaceae	Parasutterella	excrementihominis
302	Firmicutes	Clostridia	Oscillospirales	Oscillospiraceae	Flavonifractor	NA
303	Firmicutes	Clostridia	Peptostreptococcales-Tissierellales	Anaerovoracaceae	Family XIII AD3011 group	NA
304	Firmicutes	Clostridia	Oscillospirales	Oscillospiraceae	UCG-005	NA
305	Firmicutes	Clostridia	Clostridia vadinBB60 group	NA	NA	NA
306	Firmicutes	Bacilli	Erysipelotrichales	Erysipelotrichaceae	Holdemania	filiformis
307	Firmicutes	Clostridia	Lachnospirales	Lachnospiraceae	Coprococcus	catus
308	Firmicutes	Clostridia	Lachnospirales	Lachnospiraceae	[Ruminococcus] gauvreauii group	NA
309	Firmicutes	Clostridia	Oscillospirales	Oscillospiraceae	NA	NA
310	Firmicutes	Clostridia	Clostridia vadinBB60 group	NA	NA	NA
311	Firmicutes	Clostridia	Lachnospirales	Lachnospiraceae	Lachnospiraceae ND3007 group	NA
312	Firmicutes	Clostridia	Lachnospirales	Lachnospiraceae	[Ruminococcus] torques group	NA
313	Firmicutes	Clostridia	Oscillospirales	UCG-010	NA	NA
314	Firmicutes	Clostridia	Oscillospirales	Hydrogenoanaerobacterium	NA	NA
315	Firmicutes	Clostridia	Lachnospirales	Lachnospiraceae	[Eubacterium] eligens group	NA
316	Firmicutes	Clostridia	Lachnospirales	Lachnospiraceae	NA	NA
317	Firmicutes	Clostridia	Peptococcales	Peptococcaceae	NA	NA
318	Firmicutes	Clostridia	Lachnospirales	Lachnospiraceae	[Eubacterium] ruminantium group	NA
319	Firmicutes	Clostridia	Oscillospirales	Butyricicoccaceae	UCG-009	NA
320	Firmicutes	Clostridia	Oscillospirales	Ruminococcaceae	Faecalibacterium	prausnitzii

ASV_nr	DESeq_baseMean	DESeq_log2FoldChange	DESeq_lfcSE	DESeq_stat	DESeq_pvalue	DESeq_padj	ANCOM_W	ANCOM_detected_0.7
285	2,174406134	1,071114295	1,42414306	0,752111446	0,451984042	0,996224111	9	FALSE
286	131,4742105	-1,137797818	2,200386045	-0,51709009	0,605093271	0,996224111	0	FALSE
287	10,19196902	1,433067411	1,839055048	0,779241172	0,435837661	0,996224111	40	FALSE
288	4,205799086	0,488414518	1,774796471	0,275194664	0,783166687	0,996224111	9	FALSE
289	4,828639615	0,84740513	1,444153266	0,586783377	0,557349202	0,996224111	1	FALSE
290	97,09417225	0,068030243	0,740340051	0,091890534	0,926785012	0,997509535	0	FALSE
291	4,932633616	0,314532537	1,271727322	0,247327026	0,804655145	0,996224111	1	FALSE
292	297,5811893	-0,007977558	0,755944751	-0,010553096	0,991580004	0,997979415	0	FALSE
293	927,1861376	-1,054732885	0,527717284	-1,998670344	0,045644034	0,736528727	0	FALSE
294	206,3879962	-0,388759292	0,857861159	-0,45317274	0,65042436	0,996224111	0	FALSE
295	5,065010797	-0,302694475	1,514093586	-0,199917943	0,841544757	0,996224111	9	FALSE
296	4,140500653	2,996858508	1,75578779	1,706845511	0,087850771	0,967944373	74	FALSE
297	245,1455962	0,46743627	1,080919587	0,432443149	0,665419363	0,996224111	0	FALSE
298	4,246154389	1,069009106	1,806250556	0,59183877	0,553958559	0,996224111	0	FALSE
299	5,163199392	-0,500721473	1,779496588	-0,281383778	0,778416059	0,996224111	15	FALSE
300	8,033378717	-1,036451667	1,400641492	-0,739983552	0,459309975	0,996224111	0	FALSE
301	201,8203316	0,058434623	0,58502183	0,099884516	0,920436009	0,996224111	0	FALSE
302	4,882285772	-1,248614068	1,374581234	-0,908359606	0,36368826	0,996224111	3	FALSE
303	4,835993763	-0,277718532	1,566306025	-0,177307964	0,859266496	0,996224111	0	FALSE
304	190,3015921	0,091730276	0,736701547	0,124514841	0,90090765	0,996224111	0	FALSE
305	9,112742826	0,316330304	1,26314128	0,250431452	0,802253709	0,996224111	0	FALSE
306	3,471526477	1,798497186	0,896465554	2,006208914	0,044833963	0,736528727	43	FALSE
307	34,08343307	-1,490715597	0,563809083	-2,644007774	0,008193077	0,323171388	63	FALSE
308	15,70700864	0,07859753	1,132396441	0,06940814	0,94466475	0,997979415	0	FALSE
309	3,461339979	-4,129474285	1,43380559	-2,880079639	0,003975747	0,235231715	44	FALSE
310	16,65320582	2,286452527	1,514245382	1,509961697	0,131053198	0,967944373	10	FALSE
311	114,7913674	-0,355376493	0,479078605	-0,741791617	0,4582136	0,996224111	0	FALSE
312	75,92753533	-0,711892377	0,803874894	-0,885576079	0,375846007	0,996224111	0	FALSE
313	6,624888871	0,202944528	0,989528013	0,205092251	0,837500051	0,996224111	5	FALSE
314	5,759212922	1,217277185	1,609378237	0,756364885	0,449430455	0,996224111	0	FALSE
315	310,9003973	-0,310346799	0,698425147	-0,444352269	0,656787917	0,996224111	0	FALSE
316	2,472722177	-2,309154959	1,222322514	-1,889153584	0,058871252	0,770809583	4	FALSE
317	7,034446543	-0,872858611	1,524387941	-0,572596114	0,566918191	0,996224111	0	FALSE
318	105,2233228	-0,221054591	1,273391988	-0,173595086	0,862183695	0,996224111	0	FALSE
319	3,293931885	1,062684979	1,29032035	0,823582282	0,410176947	0,996224111	20	FALSE
320	832,617989	0,001074909	0,42445753	0,002532431	0,997979415	0,997979415	0	FALSE

ASV_nr	Phylum	Class	Order	Family	Genus	Species
321	Firmicutes	Negativicutes	Acidaminococcales	Acidaminococcaceae	Phascolarctobacterium	faecium
322	Verrucomicrobiota	Lentisphaeria	Victivallales	vadinBE97	NA	NA
323	Firmicutes	Clostridia	Oscillospirales	Ruminococcaceae	Angelakisella	NA
324	Firmicutes	Clostridia	Lachnospirales	Lachnospiraceae	Lachnospiraceae NK4A136 group	NA
325	Firmicutes	Clostridia	Oscillospirales	Ruminococcaceae	NA	NA
326	Firmicutes	Clostridia	Lachnospirales	Lachnospiraceae	Coprococcus	eutactus
327	Firmicutes	Clostridia	Lachnospirales	Lachnospiraceae	Lachnospiraceae NK4A136 group	NA
328	Bacteroidota	Bacteroidia	Bacteroidales	Bacteroidaceae	Bacteroides	NA
329	Firmicutes	Clostridia	Oscillospirales	Butyricicoccaceae	Butyricicoccus	NA
330	Verrucomicrobiota	Verrucomicrobiae	Opitutales	Puniceicoccaceae	NA	NA
331	Firmicutes	Clostridia	Lachnospirales	Lachnospiraceae	Coprococcus	NA
332	Firmicutes	Clostridia	Oscillospirales	UCG-010	NA	NA
333	Proteobacteria	Gammaproteobacteria	Burkholderiales	Sutterellaceae	Sutterella	wadsworthensis
334	Firmicutes	Clostridia	Lachnospirales	Lachnospiraceae	Dorea	formicigenerans
335	Firmicutes	Clostridia	Lachnospirales	Lachnospiraceae	Blautia	NA
336	Firmicutes	Clostridia	Lachnospirales	Lachnospiraceae	Lachnospiraceae UCG-010	NA
337	Bacteroidota	Bacteroidia	Bacteroidales	Bacteroidaceae	Bacteroides	NA
338	Firmicutes	Clostridia	Lachnospirales	Lachnospiraceae	[Eubacterium] eligens group	NA
339	Firmicutes	Clostridia	Christensenellales	Christensenellaceae	Christensenellaceae R-7 group	NA
340	Firmicutes	Bacilli	Erysipelotrichales	Erysipelotrichaceae	Turicibacter	sanguinis
341	Firmicutes	Clostridia	Oscillospirales	Oscillospiraceae	Colidextribacter	NA
342	Firmicutes	Clostridia	Peptococcales	Peptococcaceae	NA	NA
343	Desulfobacterota	Desulfovibrionia	Desulfovibrionales	Desulfovibrionaceae	NA	NA
344	Firmicutes	Clostridia	Oscillospirales	Oscillospiraceae	NK4A214 group	NA
345	Bacteroidota	Bacteroidia	Bacteroidales	Rikenellaceae	Alistipes	shahii
346	Firmicutes	Clostridia	Oscillospirales	Oscillospiraceae	Colidextribacter	NA
347	Firmicutes	Clostridia	Lachnospirales	Lachnospiraceae	GCA-900066575	NA
348	Firmicutes	Clostridia	Christensenellales	Christensenellaceae	Christensenellaceae R-7 group	NA
349	Firmicutes	Clostridia	Lachnospirales	Lachnospiraceae	CAG-56	NA
350	Firmicutes	Clostridia	Lachnospirales	Lachnospiraceae	[Ruminococcus] gnavus group	NA
351	Verrucomicrobiota	Lentisphaeria	Victivallales	Victivallaceae	Victivallis	vadensis
352	Firmicutes	Clostridia	Oscillospirales	Oscillospiraceae	Pseudoflavonifractor	capillosus
353	Firmicutes	Negativicutes	Acidaminococcales	Acidaminococcaceae	Phascolarctobacterium	succinatutens
354	Firmicutes	Clostridia	Oscillospirales	Ruminococcaceae	NA	NA
355	Bacteroidota	Bacteroidia	Bacteroidales	Bacteroidaceae	Bacteroides	plebeius

ASV_nr	DESeq_baseMean	DESeq_log2FoldChange	DESeq_lfcSE	DESeq_stat	DESeq_pvalue	DESeq_padj	ANCOM_W	ANCOM_detected_0.7
321	203,700959	0,350311132	1,048428152	0,334129841	0,738281583	0,996224111	0	FALSE
322	9,917191298	-0,038443696	1,137688176	-0,033791066	0,97304376	0,997979415	0	FALSE
323	4,986630674	-0,246017188	1,230145781	-0,199990271	0,84148819	0,996224111	0	FALSE
324	166,416807	-1,828148722	0,902134173	-2,02647098	0,042716544	0,736528727	0	FALSE
325	4,016285451	-0,36859999	1,303376191	-0,282803992	0,777327094	0,996224111	0	FALSE
326	162,0200231	0,51925826	1,412316737	0,367664169	0,713123661	0,996224111	0	FALSE
327	102,9892585	0,068586512	0,781577852	0,08775391	0,930072271	0,997509535	0	FALSE
328	146,4374866	1,385126787	0,916702434	1,510988447	0,130791394	0,967944373	0	FALSE
329	1,84897501	2,21724764	1,673897704	1,324601638	0,185303312	0,99239572	13	FALSE
330	9,849299934	-0,418211106	1,358038793	-0,307952253	0,758118664	0,996224111	0	FALSE
331	195,2824508	0,929814075	1,424274645	0,652833411	0,513863681	0,996224111	0	FALSE
332	3,435877556	-0,714224363	1,490926778	-0,479047243	0,63190502	0,996224111	2	FALSE
333	194,1005411	-2,248603755	0,971102067	-2,315517422	0,020584632	0,562118803	217	FALSE
334	10,0869696	0,742808437	1,200016202	0,618998673	0,535917233	0,996224111	0	FALSE
335	6,959863196	-2,193031481	1,438363401	-1,524671359	0,127341089	0,967944373	15	FALSE
336	2,872232731	0,428915125	1,638168539	0,261826005	0,793455589	0,996224111	14	FALSE
337	1463,400129	-0,347175336	0,659055209	-0,526777319	0,598348235	0,996224111	217	FALSE
338	242,2905335	-0,3437502	0,717703111	-0,478958771	0,63196796	0,996224111	0	FALSE
339	1,254783965	-0,907235221	1,326303948	-0,684032662	0,493954541	0,996224111	2	FALSE
340	14,10107505	0,596750561	1,751761394	0,340657445	0,733361478	0,996224111	12	FALSE
341	2,107946003	-0,174965802	1,479244659	-0,118280503	0,905845406	0,996224111	9	FALSE
342	2,798491831	3,036913573	1,619709374	1,874974376	0,060796249	0,770809583	48	FALSE
343	3,547035701	1,605428493	1,105250697	1,452546916	0,146349594	0,985872787	9	FALSE
344	102,9006814	0,369092198	0,895936189	0,411962595	0,68036684	0,996224111	0	FALSE
345	159,1761738	0,314196926	0,75135968	0,41817113	0,675822002	0,996224111	0	FALSE
346	4,333605199	-0,047691121	1,144874258	-0,041656208	0,966772764	0,997979415	1	FALSE
347	1,719258944	0,507105667	1,670582576	0,303550195	0,761470601	0,996224111	29	FALSE
348	2,096571488	0,425212747	1,597386	0,266192859	0,790090682	0,996224111	10	FALSE
349	63,60842268	-0,620806461	0,862842432	-0,719489952	0,471839091	0,996224111	0	FALSE
350	39,02447615	1,136243579	1,328955692	0,854989813	0,392556787	0,996224111	11	FALSE
351	6,27356907	1,061325777	1,45525476	0,729305828	0,465814608	0,996224111	12	FALSE
352	2,692725866	-0,934740857	1,708791542	-0,547018659	0,584365913	0,996224111	0	FALSE
353	55,63200818	1,430852245	2,157468831	0,663208768	0,507196857	0,996224111	13	FALSE
354	1,811287724	2,018567824	1,42763109	1,413928176	0,157382992	0,991381803	23	FALSE
355	227,8945319	0,132012231	2,135477432	0,061818603	0,950707289	0,997979415	0	FALSE

Supplementary table 6a: Differential abundance analysis at genus level of NL cohort with DESeq2 and ANCOM
All columns are spread out over two pages, after which the next two pages show the next set of rows, etc.
Genera are numbered on both pages to easily connect the taxonomy data to the associated statistics.

Genus_nr	Phylum	Class	Order	Family	Genus	Species
1	Actinobacteriota	Actinobacteria	Bifidobacteriales	Bifidobacteriaceae	Bifidobacterium	NA
2	Verrucomicrobiota	Verrucomicrobiae	Verrucomicrobiales	Akkermansiaceae	Akkermansia	NA
3	Actinobacteriota	Coriobacteriia	Coriobacteriales	Atopobiaceae	Libanicoccus	NA
4	Synergistota	Synergistia	Synergistales	Synergistaceae	Cloacibacillus	NA
5	Firmicutes	Clostridia	Lachnospirales	Lachnospiraceae	Lachnospira	NA
6	Desulfobacterota	Desulfovibrionia	Desulfovibrionales	Desulfovibrionaceae	Mailhella	NA
7	Firmicutes	Clostridia	Oscillospirales	Oscillospiraceae	UCG-003	NA
8	Firmicutes	Clostridia	Oscillospirales	Butyricicoccaceae	UCG-009	NA
9	Firmicutes	Negativicutes	Veillonellales-Selenomonadales	Veillonellaceae	Dialister	NA
10	Firmicutes	Clostridia	Lachnospirales	Lachnospiraceae	Shuttleworthia	NA
11	Firmicutes	Bacilli	Erysipelotrichales	Erysipelatoclostridiaceae	UCG-004	NA
12	Firmicutes	Clostridia	Lachnospirales	Lachnospiraceae	Fusicatenibacter	NA
13	Actinobacteriota	Coriobacteriia	Coriobacteriales	Eggerthellaceae	Senegalimassilia	NA
14	Actinobacteriota	Coriobacteriia	Coriobacteriales	Eggerthellaceae	Enterorhabdus	NA
15	Firmicutes	Clostridia	Lachnospirales	Lachnospiraceae	Lachnoclostridium	NA
16	Firmicutes	Clostridia	Peptostreptococcales-Tissierellales	Peptostreptococcaceae	Terrisporobacter	NA
17	Desulfobacterota	Desulfovibrionia	Desulfovibrionales	Desulfovibrionaceae	Bilophila	NA
18	Firmicutes	Bacilli	Erysipelotrichales	Erysipelotrichaceae	Turicibacter	NA
19	Firmicutes	Clostridia	Oscillospirales	Oscillospiraceae	Oscillibacter	NA
20	Firmicutes	Clostridia	Christensenellales	Christensenellaceae	Christensenellaceae R-7 group	NA
21	Proteobacteria	Gammaproteobacteria	Burkholderiales	Sutterellaceae	Sutterella	NA
22	Firmicutes	Clostridia	Lachnospirales	Lachnospiraceae	[Eubacterium] ventriosum group	NA
23	Firmicutes	Clostridia	Peptostreptococcales-Tissierellales	Peptostreptococcaceae	Romboutsia	NA
24	Desulfobacterota	Desulfovibrionia	Desulfovibrionales	Desulfovibrionaceae	Desulfovibrio	NA
25	Actinobacteriota	Coriobacteriia	Coriobacteriales	Coriobacteriaceae	Collinsella	NA
26	Bacteroidota	Bacteroidia	Bacteroidales	Barnesiellaceae	Barnesiella	NA
27	Proteobacteria	Gammaproteobacteria	Pasteurellales	Pasteurellaceae	Haemophilus	NA
28	Bacteroidota	Bacteroidia	Bacteroidales	Prevotellaceae	Paraprevotella	NA
29	Firmicutes	Clostridia	Lachnospirales	Lachnospiraceae	Dorea	NA
30	Firmicutes	Clostridia	Oscillospirales	Ruminococcaceae	Candidatus Soleaferrea	NA
31	Firmicutes	Bacilli	Erysipelotrichales	Erysipelatoclostridiaceae	Erysipelotrichaceae UCG-003	NA
32	Firmicutes	Clostridia	Oscillospirales	Ruminococcaceae	UBA1819	NA

Genus_nr	DESeq_baseMean	DESeq_log2FoldChange	DESeq_lfcSE	DESeq_stat	DESeq_pvalue	DESeq_padj	ANCOM_W	ANCOM_detected_0.7
1	787,5552612	-0,504009137	0,279282132	-1,804659445	0,07112799	0,80827261	3	FALSE
2	938,9544094	0,02656419	0,414547307	0,064079998	0,948906529	0,998724382	0	FALSE
3	2,790269298	0,16003065	1,458442011	0,109727126	0,912625787	0,998724382	0	FALSE
4	7,050227468	0,791843921	1,449335313	0,546349705	0,584825575	0,93728538	0	FALSE
5	144,3353381	-0,34665133	0,310674909	-1,115800858	0,264507386	0,921961878	48	FALSE
6	5,917839987	-1,286972665	1,347007353	-0,955431061	0,339359752	0,921961878	0	FALSE
7	141,0222826	0,308169373	0,349490068	0,881768614	0,377901947	0,921961878	0	FALSE
8	7,180016445	0,359363495	0,716023978	0,501887515	0,615746648	0,93728538	0	FALSE
9	710,8030274	-0,602108993	0,759300466	-0,792978565	0,427790312	0,921961878	0	FALSE
10	7,33839919	-0,475693753	0,931319581	-0,510773921	0,609509373	0,93728538	0	FALSE
11	4,48506281	1,430211278	1,154951853	1,238329783	0,215593805	0,921961878	0	FALSE
12	945,1505594	-0,635614766	0,240862278	-2,638913704	0,008317215	0,387284257	41	FALSE
13	11,10627197	0,376693874	0,576457094	0,653463854	0,513457285	0,93728538	40	FALSE
14	6,682729397	0,886704144	0,604077392	1,467865137	0,14214086	0,921961878	0	FALSE
15	273,5963996	-0,612697215	0,245005478	-2,500749046	0,012393096	0,387284257	74	FALSE
16	5,775397511	1,261948743	1,503562666	0,839305718	0,401297775	0,921961878	2	FALSE
17	55,77006489	0,512592472	0,381344033	1,344173311	0,17889235	0,921961878	0	FALSE
18	19,8641007	0,620800991	0,769008607	0,807274437	0,419508388	0,921961878	0	FALSE
19	79,16425223	0,122872864	0,284055372	0,432566591	0,665329665	0,93728538	1	FALSE
20	1042,818811	-0,360317217	0,261909466	-1,375731936	0,168904641	0,921961878	0	FALSE
21	166,0098111	-0,014148884	0,356670011	-0,039669396	0,968356701	0,998724382	0	FALSE
22	151,6197809	-0,235919097	0,353010121	-0,668306893	0,503937715	0,93728538	0	FALSE
23	47,62452854	0,569186643	0,522287179	1,089796316	0,275802877	0,921961878	0	FALSE
24	192,981777	0,444792368	0,537474419	0,827560069	0,40791969	0,921961878	0	FALSE
25	175,6915922	-0,061223094	0,247601909	-0,247264225	0,804703743	0,998724382	0	FALSE
26	342,1157616	-0,150073213	0,343875433	-0,436417373	0,662533937	0,93728538	0	FALSE
27	16,51625845	-0,444863607	0,665350499	-0,668615426	0,50374083	0,93728538	4	FALSE
28	129,2953813	0,198437571	0,629004222	0,315478917	0,752398056	0,959691398	0	FALSE
29	833,1119969	-0,108324282	0,197765557	-0,547740887	0,583869833	0,93728538	4	FALSE
30	2,718751682	2,850962407	1,352017831	2,108672195	0,034972886	0,546451352	5	FALSE
31	161,9355255	0,182689477	0,382270963	0,477905714	0,632717315	0,93728538	0	FALSE
32	29,02087296	1,847033184	0,72650209	2,542364585	0,011010526	0,387284257	67	FALSE

Genus_nr	Phylum	Class	Order	Family	Genus	Species
33	Bacteroidota	Bacteroidia	Bacteroidales	Marinifilaceae	Odoribacter	NA
34	Actinobacteriota	Coriobacteriia	Coriobacteriales	Eggerthellaceae	Adlercreutzia	NA
35	Bacteroidota	Bacteroidia	Bacteroidales	Prevotellaceae	Prevotellaceae NK3B31 group	NA
36	Bacteroidota	Bacteroidia	Bacteroidales	Bacteroidaceae	Bacteroides	NA
37	Bacteroidota	Bacteroidia	Bacteroidales	Prevotellaceae	Prevotella	NA
38	Firmicutes	Clostridia	Lachnospirales	Lachnospiraceae	[Eubacterium] xylanophilum group	NA
39	Bacteroidota	Bacteroidia	Bacteroidales	Marinifilaceae	Sanguibacteroides	NA
40	Firmicutes	Clostridia	Lachnospirales	Lachnospiraceae	Lachnospiraceae FCS020 group	NA
41	Firmicutes	Clostridia	Lachnospirales	Lachnospiraceae	Lachnospiraceae UCG-010	NA
42	Firmicutes	Clostridia	Peptococcales	Peptococcaceae	Peptococcus	NA
43	Firmicutes	Clostridia	Lachnospirales	Lachnospiraceae	Lachnospiraceae UCG-004	NA
44	Firmicutes	Clostridia	Oscillospirales	Oscillospiraceae	Intestinimonas	NA
45	Firmicutes	Clostridia	Lachnospirales	Lachnospiraceae	Lachnospiraceae UCG-001	NA
46	Firmicutes	Bacilli	Lactobacillales	Streptococcaceae	Streptococcus	NA
47	Firmicutes	Clostridia	Oscillospirales	Oscillospiraceae	Flavonifractor	NA
48	Firmicutes	Bacilli	Acholeplasmatales	Acholeplasmataceae	Anaeroplasma	NA
49	Bacteroidota	Bacteroidia	Bacteroidales	Prevotellaceae	Alloprevotella	NA
50	Firmicutes	Clostridia	Lachnospirales	Lachnospiraceae	Tyzzerella	NA
51	Euryarchaeota	Methanobacteria	Methanobacteriales	Methanobacteriaceae	Methanosphaera	NA
52	Firmicutes	Bacilli	Erysipelotrichales	Erysipelotrichaceae	Solobacterium	NA
53	Euryarchaeota	Methanobacteria	Methanobacteriales	Methanobacteriaceae	Methanobrevibacter	NA
54	Firmicutes	Clostridia	Lachnospirales	Lachnospiraceae	Lachnospiraceae AC2044 group	NA
55	Firmicutes	Clostridia	Oscillospirales	Ruminococcaceae	CAG-352	NA
56	Firmicutes	Clostridia	Oscillospirales	Ruminococcaceae	Paludicola	NA
57	Firmicutes	Clostridia	Clostridiales	Clostridiaceae	Clostridium sensu stricto 1	NA
58	Firmicutes	Bacilli	Erysipelotrichales	Erysipelatoclostridiaceae	Erysipelatoclostridium	NA
59	Bacteroidota	Bacteroidia	Bacteroidales	Rikenellaceae	Rikenellaceae RC9 gut group	NA
60	Actinobacteriota	Coriobacteriia	Coriobacteriales	Eggerthellaceae	Slackia	NA
61	Firmicutes	Bacilli	Erysipelotrichales	Erysipelotrichaceae	Catenisphaera	NA
62	Firmicutes	Clostridia	Peptostreptococcales-Tissierellales	Anaerovoracaceae	Mogibacterium	NA
63	Firmicutes	Clostridia	Oscillospirales	Ruminococcaceae	Subdoligranulum	NA
64	Proteobacteria	Gammaproteobacteria	Enterobacterales	Enterobacteriaceae	Escherichia-Shigella	NA
65	Firmicutes	Clostridia	Oscillospirales	Ruminococcaceae	Negativibacillus	NA
66	Firmicutes	Bacilli	Lactobacillales	Streptococcaceae	Lactococcus	NA
67	Firmicutes	Clostridia	Lachnospirales	Lachnospiraceae	GCA-900066575	NA
68	Bacteroidota	Bacteroidia	Bacteroidales	Rikenellaceae	Alistipes	NA

Genus_nr	DESeq_baseMean	DESeq_log2FoldChange	DESeq_lfcSE	DESeq_stat	DESeq_pvalue	DESeq_padj	ANCOM_W	ANCOM_detected_0.7
33	36,92306656	-0,043690106	0,250652075	-0,174305781	0,861625156	0,998724382	0	FALSE
34	10,78988812	-0,854777686	0,684353996	-1,24902856	0,211654628	0,921961878	0	FALSE
35	40,69506426	2,328610312	1,875805548	1,241392166	0,214460912	0,921961878	5	FALSE
36	4973,201333	0,111285557	0,154716704	0,719285985	0,47196473	0,93728538	0	FALSE
37	1242,415231	0,09507852	0,700557148	0,135718435	0,892043874	0,998724382	0	FALSE
38	63,11978266	-0,037472695	0,39757669	-0,094252747	0,924908385	0,998724382	48	FALSE
39	1,766825591	0,51204276	1,335094567	0,383525461	0,701330195	0,949155048	3	FALSE
40	68,29439319	-0,544493784	0,383103716	-1,421269909	0,155238308	0,921961878	3	FALSE
41	70,98356239	-0,095364531	0,471641676	-0,202196998	0,83976272	0,998724382	0	FALSE
42	41,49514272	-0,028140662	0,587494676	-0,047899433	0,961796391	0,998724382	0	FALSE
43	62,48573769	-0,204197529	0,455416761	-0,448375086	0,653882519	0,93728538	0	FALSE
44	105,6107238	0,427359412	0,228018989	1,87422729	0,060899104	0,761238803	5	FALSE
45	75,06824636	-0,248460527	0,519890254	-0,477909569	0,63271457	0,93728538	44	FALSE
46	297,7428676	-0,437455675	0,367402048	-1,190672938	0,233782006	0,921961878	0	FALSE
47	17,89575696	0,482164496	0,684959035	0,703931872	0,481475202	0,93728538	1	FALSE
48	27,76841217	-0,861239674	1,290114188	-0,667568563	0,504409031	0,93728538	0	FALSE
49	96,87725678	1,671358608	1,288421083	1,297214576	0,194557367	0,921961878	0	FALSE
50	47,29805697	-0,055039912	0,998116048	-0,055143801	0,956023901	0,998724382	0	FALSE
51	4,105280962	-0,096925801	1,401415012	-0,069162811	0,944860025	0,998724382	3	FALSE
52	11,98506367	-0,263785297	1,466506754	-0,179873223	0,857252097	0,998724382	0	FALSE
53	109,8245709	0,387639092	0,481816047	0,80453753	0,421086604	0,921961878	10	FALSE
54	48,32614331	0,725555175	0,908096077	0,798985034	0,42429909	0,921961878	0	FALSE
55	416,3831935	-0,811980869	0,924862599	-0,877947567	0,379972174	0,921961878	0	FALSE
56	4,624830107	2,728651348	1,20584544	2,262853312	0,023644733	0,428886148	20	FALSE
57	105,1803156	-0,398228711	0,499124279	-0,797854819	0,424954745	0,921961878	0	FALSE
58	20,55194144	0,849003776	0,643980994	1,318367752	0,187380571	0,921961878	0	FALSE
59	157,6692463	2,407030706	1,001820232	2,402657314	0,016276432	0,406910803	89	TRUE
60	10,14499136	-0,362841136	0,580724847	-0,624807321	0,532097525	0,93728538	0	FALSE
61	28,61694042	1,746562077	1,290894194	1,352986236	0,176060028	0,921961878	0	FALSE
62	17,5929751	0,019948155	1,321208475	0,015098416	0,987953665	0,998724382	0	FALSE
63	1153,941853	-0,281478191	0,171776387	-1,638631455	0,101290037	0,921961878	0	FALSE
64	619,6209959	-0,280603779	0,618917497	-0,453378326	0,650276341	0,93728538	0	FALSE
65	48,02344319	-0,574101865	0,372101736	-1,542862637	0,122864108	0,921961878	1	FALSE
66	6,529281582	1,322018368	1,472226626	0,897972055	0,369200448	0,921961878	4	FALSE
67	21,21528441	-0,018156759	0,46564122	-0,038993023	0,968895952	0,998724382	2	FALSE
68	1278,678738	-0,169267992	0,205024441	-0,825599091	0,409031551	0,921961878	0	FALSE

Genus_nr	Phylum	Class	Order	Family	Genus	Species
69	Firmicutes	Negativicutes	Veillonellales-Selenomonadales	Veillonellaceae	Veillonella	NA
70	Firmicutes	Clostridia	Oscillospirales	Oscillospiraceae	Colidextribacter	NA
71	Firmicutes	Clostridia	Oscillospirales	Oscillospiraceae	UCG-002	NA
72	Firmicutes	Clostridia	Peptostreptococcales-Tissierellales	Anaerovoracaceae	Family XIII UCG-001	NA
73	Firmicutes	Clostridia	Peptostreptococcales-Tissierellales	Anaerovoracaceae	Family XIII AD3011 group	NA
74	Firmicutes	Clostridia	Peptostreptococcales-Tissierellales	Peptostreptococcaceae	Intestinibacter	NA
75	Firmicutes	Clostridia	Peptostreptococcales-Tissierellales	Anaerovoracaceae	[Eubacterium] brachy group	NA
76	Firmicutes	Clostridia	Lachnospirales	Lachnospiraceae	Anaerostipes	NA
77	Firmicutes	Clostridia	Lachnospirales	Lachnospiraceae	Lachnotalea	NA
78	Firmicutes	Clostridia	Lachnospirales	Lachnospiraceae	Lachnospiraceae UCG-003	NA
79	Firmicutes	Clostridia	Lachnospirales	Lachnospiraceae	Marvinbryantia	NA
80	Firmicutes	Clostridia	Lachnospirales	Lachnospiraceae	[Eubacterium] hallii group	NA
81	Bacteroidota	Bacteroidia	Bacteroidales	Barnesiellaceae	Coprobacter	NA
82	Bacteroidota	Bacteroidia	Bacteroidales	Tannerellaceae	Parabacteroides	NA
83	Firmicutes	Clostridia	Oscillospirales	Ruminococcaceae	DTU089	NA
84	Firmicutes	Clostridia	Oscillospirales	Ruminococcaceae	Ruminococcus	NA
85	Firmicutes	Bacilli	Erysipelotrichales	Erysipelotrichaceae	Holdemanella	NA
86	Firmicutes	Clostridia	Oscillospirales	Oscillospiraceae	Oscillospira	NA
87	Firmicutes	Bacilli	Lactobacillales	Lactobacillaceae	Lactobacillus	NA
88	Firmicutes	Bacilli	Erysipelotrichales	Erysipelatoclostridiaceae	Coprobacillus	NA
89	Actinobacteriota	Coriobacteriia	Coriobacteriales	Atopobiaceae	Olsenella	NA
90	Verrucomicrobiota	Lentisphaeria	Victivallales	Victivallaceae	Victivallis	NA
91	Actinobacteriota	Actinobacteria	Actinomycetales	Actinomycetaceae	Actinomyces	NA
92	Firmicutes	Clostridia	Lachnospirales	Lachnospiraceae	Blautia	NA
93	Firmicutes	Clostridia	Oscillospirales	Oscillospiraceae	UCG-005	NA
94	Firmicutes	Clostridia	Lachnospirales	Lachnospiraceae	Howardella	NA
95	Firmicutes	Clostridia	Lachnospirales	Lachnospiraceae	Eisenbergiella	NA
96	Firmicutes	Clostridia	Lachnospirales	Lachnospiraceae	[Ruminococcus] torques group	NA
97	Proteobacteria	Gammaproteobacteria	Burkholderiales	Oxalobacteraceae	Oxalobacter	NA
98	Firmicutes	Clostridia	Monoglobales	Monoglobaceae	Monoglobus	NA
99	Firmicutes	Negativicutes	Veillonellales-Selenomonadales	Veillonellaceae	Allisonella	NA
100	Firmicutes	Bacilli	Erysipelotrichales	Erysipelatoclostridiaceae	Asteroleplasma	NA
101	Bacteroidota	Bacteroidia	Bacteroidales	Marinifilaceae	Butyricimonas	NA
102	Firmicutes	Clostridia	Lachnospirales	Lachnospiraceae	Roseburia	NA
103	Firmicutes	Negativicutes	Acidaminococcales	Acidaminococcaceae	Acidaminococcus	NA
104	Firmicutes	Bacilli	Erysipelotrichales	Erysipelotrichaceae	Merdibacter	NA

Genus_nr	DESeq_baseMean	DESeq_log2FoldChange	DESeq_lfcSE	DESeq_stat	DESeq_pvalue	DESeq_padj	ANCOM_W	ANCOM_detected_0.7
69	33,21073277	-0,556086329	0,681201266	-0,816331907	0,41431033	0,921961878	60	FALSE
70	95,62793386	0,027619168	0,20425417	0,135219603	0,892438248	0,998724382	2	FALSE
71	1900,689219	-0,035638101	0,192765955	-0,184877568	0,853325066	0,998724382	0	FALSE
72	29,75171423	-0,39447939	0,335074863	-1,177287329	0,239080843	0,921961878	0	FALSE
73	61,73380419	0,03302554	0,257813495	0,12809857	0,898070968	0,998724382	0	FALSE
74	34,05938274	0,6775447	0,689323732	0,982912192	0,325650657	0,921961878	0	FALSE
75	1,895133085	1,88417833	1,184358162	1,590885587	0,111635327	0,921961878	1	FALSE
76	170,1486797	-0,016073111	0,271994184	-0,059093582	0,95287757	0,998724382	0	FALSE
77	6,099233461	0,198300381	0,87575583	0,226433412	0,820864344	0,998724382	0	FALSE
78	10,31188165	4,265271911	1,63184464	2,613773277	0,008954844	0,387284257	6	FALSE
79	45,21048324	0,316036117	0,52729285	0,599355968	0,548935533	0,93728538	0	FALSE
80	197,9264895	-0,085018139	0,197812439	-0,429791673	0,66734719	0,93728538	0	FALSE
81	16,84236381	0,115321886	0,529047643	0,217980153	0,827444575	0,998724382	0	FALSE
82	579,4437653	0,442263477	0,195965184	2,256847199	0,024017624	0,428886148	28	FALSE
83	11,63682114	0,117652642	0,586060503	0,200751699	0,840892733	0,998724382	0	FALSE
84	1446,773467	-0,135837169	0,230277256	-0,589885302	0,555267549	0,93728538	0	FALSE
85	213,2490598	0,480783925	0,660334595	0,728091378	0,466557652	0,93728538	0	FALSE
86	20,74463267	1,421379977	0,693674857	2,049057946	0,040456449	0,561895126	0	FALSE
87	115,5111088	-0,304812768	0,808515441	-0,377003026	0,706171356	0,949155048	0	FALSE
88	6,108803747	-1,005911706	1,41997606	-0,708400469	0,478696601	0,93728538	2	FALSE
89	4,620308429	1,54687758	0,987669744	1,566189093	0,117304355	0,921961878	13	FALSE
90	6,790501764	-0,001346275	0,842078976	-0,001598751	0,998724382	0,998724382	0	FALSE
91	1,337681554	0,567423659	1,277317392	0,444230747	0,656875765	0,93728538	0	FALSE
92	1293,319652	-0,079984683	0,143679145	-0,556689583	0,577739535	0,93728538	0	FALSE
93	651,2670336	0,009025617	0,246129904	0,036670136	0,97074802	0,998724382	18	FALSE
94	9,020497354	0,243308127	0,730142981	0,333233536	0,738958005	0,959691398	0	FALSE
95	14,73235237	1,39169402	1,162597829	1,197055409	0,231284959	0,921961878	11	FALSE
96	614,1446083	0,069653798	0,247789346	0,28110086	0,778633042	0,983122528	0	FALSE
97	11,41139592	0,430475005	0,450985719	0,954520258	0,33982036	0,921961878	48	FALSE
98	123,7042608	0,304683122	0,267362729	1,139587118	0,254458355	0,921961878	0	FALSE
99	11,76170698	-0,441788277	1,100374034	-0,401489187	0,688059997	0,949155048	0	FALSE
100	9,015050718	0,642483719	1,085458465	0,591900787	0,553917027	0,93728538	0	FALSE
101	36,75284707	0,258348548	0,318662775	0,8107271	0,417522407	0,921961878	0	FALSE
102	896,8603581	-0,14095468	0,262223066	-0,537537305	0,590896524	0,93728538	73	FALSE
103	40,5458594	-0,557502231	1,747570979	-0,319015501	0,749714759	0,959691398	1	FALSE
104	3,177706112	-1,711707942	1,289149435	-1,327780858	0,184250512	0,921961878	4	FALSE

Genus_nr	Phylum	Class	Order	Family	Genus	Species
105	Firmicutes	Clostridia	Oscillospirales	Ruminococcaceae	[Eubacterium] siraeum group	NA
106	Firmicutes	Bacilli	Erysipelotrichales	Erysipelatoclostridiaceae	Catenibacterium	NA
107	Firmicutes	Clostridia	Oscillospirales	Ruminococcaceae	Incertae Sedis	NA
108	Firmicutes	Clostridia	Oscillospirales	Butyricicoccaceae	Butyricicoccus	NA
109	Firmicutes	Clostridia	Oscillospirales	Ruminococcaceae	Angelakisella	NA
110	Firmicutes	Clostridia	Lachnospirales	Lachnospiraceae	Agathobacter	NA
111	Proteobacteria	Gammaproteobacteria	Burkholderiales	Sutterellaceae	Parasutterella	NA
112	Firmicutes	Bacilli	Erysipelotrichales	Erysipelotrichaceae	Holdemania	NA
113	Firmicutes	Clostridia	Lachnospirales	Lachnospiraceae	[Ruminococcus] gauvreauii group	NA
114	Firmicutes	Clostridia	Lachnospirales	Lachnospiraceae	Lachnospiraceae ND3007 group	NA
115	Firmicutes	Clostridia	Lachnospirales	Lachnospiraceae	[Eubacterium] eligens group	NA
116	Firmicutes	Clostridia	Lachnospirales	Lachnospiraceae	[Eubacterium] ruminantium group	NA
117	Firmicutes	Clostridia	Oscillospirales	Ruminococcaceae	Faecalibacterium	NA
118	Firmicutes	Clostridia	Lachnospirales	Lachnospiraceae	Lachnospiraceae NK4A136 group	NA
119	Firmicutes	Clostridia	Lachnospirales	Lachnospiraceae	Coprococcus	NA
120	Firmicutes	Clostridia	Lachnospirales	Defluviitaleaceae	Defluviitaleaceae UCG-011	NA
121	Firmicutes	Clostridia	Oscillospirales	Ruminococcaceae	Fournierella	NA
122	Firmicutes	Clostridia	Oscillospirales	Oscillospiraceae	NK4A214 group	NA
123	Firmicutes	Clostridia	Lachnospirales	Lachnospiraceae	CAG-56	NA
124	Firmicutes	Clostridia	Lachnospirales	Lachnospiraceae	[Ruminococcus] gnavus group	NA
125	Firmicutes	Negativicutes	Acidaminococcales	Acidaminococcaceae	Phascolarctobacterium	NA

Genus_nr	DESeq_baseMean	DESeq_log2FoldChange	DESeq_lfcSE	DESeq_stat	DESeq_pvalue	DESeq_padj	ANCOM_W	ANCOM_detected_0.7
105	226,7335705	-0,412365691	0,428427996	-0,962508739	0,335794116	0,921961878	0	FALSE
106	62,1699047	-0,0039917	1,351774028	-0,002952934	0,997643903	0,998724382	6	FALSE
107	97,27127289	0,006880694	0,23885853	0,028806567	0,977018864	0,998724382	0	FALSE
108	187,6701238	-0,213726749	0,219771967	-0,972493223	0,330805235	0,921961878	66	FALSE
109	5,834726144	-0,400719704	0,744407708	-0,538306763	0,590365283	0,93728538	0	FALSE
110	2638,991627	-0,25543167	0,259242029	-0,985301925	0,324475787	0,921961878	5	FALSE
111	67,04614986	0,081862713	0,628403447	0,130270948	0,896352064	0,998724382	13	FALSE
112	3,042323865	-0,189205143	0,557049636	-0,339655806	0,734115747	0,959691398	0	FALSE
113	84,82470081	-0,659233272	0,541044782	-1,21844493	0,22305494	0,921961878	66	FALSE
114	55,43497377	0,171584909	0,361406376	0,474770011	0,634950911	0,93728538	0	FALSE
115	236,4222898	-0,554448118	0,353751416	-1,567338229	0,117035654	0,921961878	13	FALSE
116	231,7958093	-0,381688241	0,824609199	-0,462871674	0,643456352	0,93728538	0	FALSE
117	2761,210438	-0,153656571	0,167791147	-0,915760898	0,359792316	0,921961878	6	FALSE
118	367,1059827	-0,426759667	0,333087664	-1,281223276	0,200115253	0,921961878	88	TRUE
119	852,3860397	-0,188192165	0,218632209	-0,860770541	0,389364433	0,921961878	4	FALSE
120	1,993675396	1,167477218	1,251455501	0,93289551	0,350873921	0,921961878	4	FALSE
121	10,14285887	-0,416369382	1,098630245	-0,37898955	0,704695624	0,949155048	0	FALSE
122	322,1012395	-0,173975838	0,260948127	-0,666706599	0,504959563	0,93728538	30	FALSE
123	157,9673326	0,152397127	0,427038808	0,356869502	0,721189502	0,959028593	0	FALSE
124	33,36559137	-0,965982131	1,544252383	-0,625533845	0,531620742	0,93728538	0	FALSE
125	373,3522964	0,464318641	0,466979054	0,994302928	0,320075411	0,921961878	0	FALSE

Supplementary table 6b: Differential abundance analysis at genus level of FIN cohort with DESeq2 and ANCOM
All columns are spread out over two pages, after which the next two pages show the next set of rows, etc.
Genera are numbered on both pages to easily connect the taxonomy data to the associated statistics.

Genus_nr	Phylum	Class	Order	Family	Genus	Species
1	Actinobacteriota	Actinobacteria	Bifidobacteriales	Bifidobacteriaceae	Bifidobacterium	NA
2	Verrucomicrobiota	Verrucomicrobiae	Verrucomicrobiales	Akkermansiaceae	Akkermansia	NA
3	Firmicutes	Clostridia	Oscillospirales	Ruminococcaceae	Paludicola	NA
4	Synergistota	Synergistia	Synergistales	Synergistaceae	Cloacibacillus	NA
5	Firmicutes	Clostridia	Lachnospirales	Lachnospiraceae	Lachnospira	NA
6	Firmicutes	Clostridia	Oscillospirales	Oscillospiraceae	UCG-003	NA
7	Firmicutes	Negativicutes	Veillonellales-Selenomonadales	Veillonellaceae	Dialister	NA
8	Bacteroidota	Bacteroidia	Bacteroidales	Barnesiellaceae	Barnesiella	NA
9	Firmicutes	Clostridia	Lachnospirales	Lachnospiraceae	Fusicatenibacter	NA
10	Actinobacteriota	Coriobacteriia	Coriobacteriales	Eggerthellaceae	Senegalimassilia	NA
11	Actinobacteriota	Coriobacteriia	Coriobacteriales	Eggerthellaceae	Enterorhabdus	NA
12	Firmicutes	Clostridia	Lachnospirales	Lachnospiraceae	Lachnoclostridium	NA
13	Firmicutes	Bacilli	Erysipelotrichales	Erysipelatoclostridiaceae	UCG-004	NA
14	Desulfobacterota	Desulfovibrionia	Desulfovibrionales	Desulfovibrionaceae	Bilophila	NA
15	Firmicutes	Clostridia	Oscillospirales	Oscillospiraceae	Oscillibacter	NA
16	Firmicutes	Clostridia	Christensenellales	Christensenellaceae	Christensenellaceae R-7 group	NA
17	Firmicutes	Clostridia	Lachnospirales	Lachnospiraceae	[Eubacterium] ventriosum group	NA
18	Firmicutes	Clostridia	Peptostreptococcales-Tissierellales	Peptostreptococcaceae	Romboutsia	NA
19	Desulfobacterota	Desulfovibrionia	Desulfovibrionales	Desulfovibrionaceae	Desulfovibrio	NA
20	Actinobacteriota	Coriobacteriia	Coriobacteriales	Coriobacteriaceae	Collinsella	NA
21	Proteobacteria	Gammaproteobacteria	Pasteurellales	Pasteurellaceae	Haemophilus	NA
22	Bacteroidota	Bacteroidia	Bacteroidales	Prevotellaceae	Paraprevotella	NA
23	Firmicutes	Clostridia	Oscillospirales	Ruminococcaceae	Candidatus Soleaferrea	NA
24	Firmicutes	Clostridia	Lachnospirales	Lachnospiraceae	Dorea	NA
25	Firmicutes	Bacilli	Erysipelotrichales	Erysipelatoclostridiaceae	Asteroleplasma	NA
26	Firmicutes	Bacilli	Erysipelotrichales	Erysipelatoclostridiaceae	Erysipelotrichaceae UCG-003	NA
27	Firmicutes	Clostridia	Oscillospirales	Ruminococcaceae	UBA1819	NA
28	Bacteroidota	Bacteroidia	Bacteroidales	Marinifilaceae	Odoribacter	NA
29	Actinobacteriota	Coriobacteriia	Coriobacteriales	Eggerthellaceae	Adlercreutzia	NA
30	Bacteroidota	Bacteroidia	Bacteroidales	Bacteroidaceae	Bacteroides	NA
31	Bacteroidota	Bacteroidia	Bacteroidales	Prevotellaceae	Prevotella	NA
32	Firmicutes	Clostridia	Lachnospirales	Lachnospiraceae	[Eubacterium] xylanophilum group	NA

Genus_nr	DESeq_baseMean	DESeq_log2FoldChange	DESeq_lfcSE	DESeq_stat	DESeq_pvalue	DESeq_padj	ANCOM_W	ANCOM_detected_0.7
1	775,5820718	0,602164753	0,407865368	1,47638118	0,139841592	0,884937089	0	FALSE
2	804,2323755	0,640903482	0,521830412	1,228183462	0,219378105	0,884937089	15	FALSE
3	10,17494755	-0,85074008	0,94595369	-0,899346436	0,368468161	0,95754171	0	FALSE
4	3,816103183	1,592623542	1,694189552	0,940050386	0,347191716	0,95754171	9	FALSE
5	275,4525062	-0,272631419	0,436579078	-0,62447202	0,53231764	0,984760571	0	FALSE
6	119,5658276	-0,384500321	0,468133779	-0,821347098	0,411448588	0,984760571	1	FALSE
7	599,4864219	-0,33171634	0,721473455	-0,459776223	0,645676851	0,984760571	0	FALSE
8	564,6487909	0,598574203	0,581389143	1,029558619	0,303217249	0,929393191	0	FALSE
9	262,8175553	-0,29044081	0,367380153	-0,790572948	0,429193238	0,984760571	0	FALSE
10	6,299103573	2,558516206	1,055959484	2,422930278	0,015395881	0,292521743	36	FALSE
11	2,652740593	0,655015247	1,217580714	0,537964539	0,590601531	0,984760571	6	FALSE
12	304,0905361	-0,497284808	0,375009351	-1,326059757	0,184819905	0,884937089	0	FALSE
13	7,107510876	0,429884622	1,487805525	0,288938719	0,772628274	0,984760571	0	FALSE
14	98,36745439	0,074308053	0,449676309	0,165247871	0,868748887	0,984760571	0	FALSE
15	95,76257914	-0,001491509	0,354965976	-0,004201837	0,996647429	0,996647429	0	FALSE
16	1459,930191	-0,0176949	0,390312246	-0,045335242	0,963840097	0,989163896	0	FALSE
17	34,86169399	1,052346437	0,759689471	1,385232358	0,165981429	0,884937089	0	FALSE
18	19,15380607	-0,603343609	1,017285312	-0,593091832	0,553119696	0,984760571	0	FALSE
19	83,63412293	-0,010933237	1,158705008	-0,009435738	0,992471482	0,996647429	0	FALSE
20	62,50387182	0,174690105	0,440612993	0,396470617	0,69175788	0,984760571	0	FALSE
21	89,59610647	1,344045805	0,788662396	1,704209319	0,088341989	0,884937089	0	FALSE
22	137,5263711	-0,549385348	1,243027421	-0,441973635	0,658508284	0,984760571	0	FALSE
23	1,355431216	-0,518417049	1,478609499	-0,350611199	0,725880054	0,984760571	4	FALSE
24	94,4269867	0,395066049	0,410291841	0,962890335	0,33560256	0,95754171	0	FALSE
25	178,3116858	-0,228457268	1,503459944	-0,151954343	0,879222946	0,984760571	0	FALSE
26	78,8550164	-0,122103393	0,483420133	-0,25258235	0,800590971	0,984760571	0	FALSE
27	41,82856338	-0,361964399	0,518754706	-0,697756367	0,485329571	0,984760571	7	FALSE
28	147,3542091	0,661855938	0,400026918	1,654528503	0,098020177	0,884937089	0	FALSE
29	2,356183708	0,513993923	1,120756738	0,45861328	0,646511899	0,984760571	4	FALSE
30	9037,295113	0,369993894	0,16372914	2,259792571	0,023834128	0,388155794	5	FALSE
31	1758,38562	-0,683657853	1,148839032	-0,595085851	0,551786086	0,984760571	0	FALSE
32	60,85391201	-0,308735497	0,531761592	-0,580590065	0,56151677	0,984760571	17	FALSE

Genus_nr	Phylum	Class	Order	Family	Genus	Species
33	Bacteroidota	Bacteroidia	Bacteroidales	Porphyromonadaceae	Porphyromonas	NA
34	Bacteroidota	Bacteroidia	Bacteroidales	Rikenellaceae	Rikenellaceae RC9 gut group	NA
35	Firmicutes	Clostridia	Lachnospirales	Lachnospiraceae	Lachnospiraceae FCS020 group	NA
36	Firmicutes	Clostridia	Lachnospirales	Lachnospiraceae	Lachnospiraceae UCG-010	NA
37	Firmicutes	Clostridia	Lachnospirales	Lachnospiraceae	Lachnospiraceae UCG-004	NA
38	Firmicutes	Clostridia	Oscillospirales	Oscillospiraceae	Intestinimonas	NA
39	Firmicutes	Clostridia	Lachnospirales	Lachnospiraceae	Lachnospiraceae UCG-001	NA
40	Firmicutes	Bacilli	Lactobacillales	Streptococcaceae	Streptococcus	NA
41	Firmicutes	Clostridia	Oscillospirales	Oscillospiraceae	Flavonifractor	NA
42	Firmicutes	Bacilli	Acholeplasmatales	Acholeplasmataceae	Anaeroplasma	NA
43	Firmicutes	Clostridia	Lachnospirales	Lachnospiraceae	Tyzzerella	NA
44	Euryarchaeota	Methanobacteria	Methanobacteriales	Methanobacteriaceae	Methanobrevibacter	NA
45	Firmicutes	Clostridia	Lachnospirales	Lachnospiraceae	Lachnospiraceae AC2044 group	NA
46	Firmicutes	Clostridia	Peptostreptococcales-Tissierellales	Anaerovoracaceae	Family XIII UCG-001	NA
47	Firmicutes	Clostridia	Oscillospirales	Ruminococcaceae	CAG-352	NA
48	Patescibacteria	Saccharimonadia	Saccharimonadales	Saccharimonadaceae	TM7x	NA
49	Firmicutes	Clostridia	Clostridiales	Clostridiaceae	Clostridium sensu stricto 1	NA
50	Firmicutes	Bacilli	Erysipelotrichales	Erysipelatoclostridiaceae	Erysipelatoclostridium	NA
51	Actinobacteriota	Coriobacteriia	Coriobacteriales	Eggerthellaceae	Slackia	NA
52	Firmicutes	Clostridia	Oscillospirales	Ruminococcaceae	Subdoligranulum	NA
53	Proteobacteria	Gammaproteobacteria	Enterobacterales	Enterobacteriaceae	Escherichia-Shigella	NA
54	Firmicutes	Clostridia	Oscillospirales	Ruminococcaceae	Negativibacillus	NA
55	Firmicutes	Bacilli	Lactobacillales	Streptococcaceae	Lactococcus	NA
56	Firmicutes	Clostridia	Lachnospirales	Lachnospiraceae	GCA-900066575	NA
57	Actinobacteriota	Coriobacteriia	Coriobacteriales	Eggerthellaceae	Eggerthella	NA
58	Bacteroidota	Bacteroidia	Bacteroidales	Rikenellaceae	Alistipes	NA
59	Firmicutes	Bacilli	Lactobacillales	Lactobacillaceae	Lactobacillus	NA
60	Firmicutes	Clostridia	Oscillospirales	Oscillospiraceae	UCG-002	NA
61	Firmicutes	Clostridia	Peptostreptococcales-Tissierellales	Anaerovoracaceae	Family XIII AD3011 group	NA
62	Firmicutes	Clostridia	Peptostreptococcales-Tissierellales	Peptostreptococcaceae	Intestinibacter	NA
63	Firmicutes	Clostridia	Lachnospirales	Lachnospiraceae	Anaerostipes	NA
64	Firmicutes	Clostridia	Lachnospirales	Lachnospiraceae	Lachnotalea	NA
65	Firmicutes	Clostridia	Lachnospirales	Lachnospiraceae	Lachnospiraceae UCG-003	NA
66	Firmicutes	Clostridia	Lachnospirales	Lachnospiraceae	[Eubacterium] hallii group	NA
67	Firmicutes	Clostridia	Oscillospirales	Oscillospiraceae	Colidextribacter	NA
68	Bacteroidota	Bacteroidia	Bacteroidales	Barnesiellaceae	Coprobacter	NA

Genus_nr	DESeq_baseMean	DESeq_log2FoldChange	DESeq_lfcSE	DESeq_stat	DESeq_pvalue	DESeq_padj	ANCOM_W	ANCOM_detected_0.7
33	1,314579778	-1,489438421	1,707555137	-0,872263735	0,383064517	0,970430109	3	FALSE
34	136,9392278	0,793723888	2,124258865	0,373647441	0,708666634	0,984760571	0	FALSE
35	6,017547394	-1,806508123	0,978923288	-1,845403154	0,064978915	0,740759627	3	FALSE
36	41,12128815	-0,693869099	0,572009717	-1,21303726	0,225115575	0,884937089	0	FALSE
37	101,8859789	-0,605070735	0,482567831	-1,25385634	0,209894227	0,884937089	48	FALSE
38	92,80810667	0,086248769	0,294395984	0,292968563	0,769546181	0,984760571	1	FALSE
39	29,34021106	-0,558737514	0,835085368	-0,669078319	0,50344552	0,984760571	0	FALSE
40	108,5226514	0,098517848	0,474770349	0,207506321	0,835614457	0,984760571	0	FALSE
41	52,2587205	-0,199871341	0,640520043	-0,312045413	0,755006011	0,984760571	0	FALSE
42	77,86746416	-5,182124336	1,831952823	-2,828743334	0,004673116	0,133183807	0	FALSE
43	37,87387385	1,73487241	1,146680078	1,51295243	0,130291743	0,884937089	0	FALSE
44	113,3823166	1,486791525	0,787086745	1,888980515	0,05889444	0,740759627	0	FALSE
45	40,09212544	-0,753306071	1,216475169	-0,619253142	0,535749608	0,984760571	0	FALSE
46	35,31034152	-0,207224374	0,379523728	-0,546011641	0,585057935	0,984760571	0	FALSE
47	371,850877	0,572047283	1,264110821	0,452529377	0,650887663	0,984760571	0	FALSE
48	0,817865401	0,276548667	1,555155718	0,177826994	0,858858847	0,984760571	6	FALSE
49	112,4439981	0,194006628	0,751883241	0,2580276	0,796385606	0,984760571	0	FALSE
50	12,48720858	1,02544232	0,81666342	1,255648649	0,209243385	0,884937089	33	FALSE
51	3,867813976	0,048933126	1,384713025	0,035338099	0,971810144	0,989163896	3	FALSE
52	2266,213968	-0,330353646	0,311171135	-1,061646176	0,288396342	0,929393191	0	FALSE
53	392,6928449	-0,693852108	0,759779789	-0,913227908	0,361122689	0,95754171	0	FALSE
54	38,90681401	0,825078489	0,631117881	1,307328652	0,191101117	0,884937089	45	FALSE
55	10,58131867	-0,186108281	1,030051487	-0,18067862	0,856619842	0,984760571	5	FALSE
56	14,2901	-1,94564223	0,613709354	-3,170299128	0,001522821	0,086800787	46	FALSE
57	8,254600273	2,075916726	1,605536209	1,292974095	0,196020019	0,884937089	1	FALSE
58	1775,597039	0,017254546	0,267534183	0,064494736	0,948576299	0,984760571	0	FALSE
59	23,25246006	0,4233932	1,374607054	0,30801035	0,758074457	0,984760571	0	FALSE
60	1646,024615	0,314251931	0,308852975	1,017480665	0,308924826	0,929393191	0	FALSE
61	31,17933203	-0,22441333	0,486553534	-0,4612305	0,644633242	0,984760571	0	FALSE
62	34,21764089	-1,018706734	0,794162248	-1,282743842	0,199581834	0,884937089	0	FALSE
63	30,53219782	0,107210396	0,64806099	0,165432571	0,868603519	0,984760571	0	FALSE
64	12,86976158	-2,499989185	1,013926761	-2,465650657	0,013676465	0,292521743	67	FALSE
65	38,51355377	-1,529170746	1,701302715	-0,898823433	0,368746717	0,95754171	0	FALSE
66	6,351742356	0,45540757	0,958766278	0,474993312	0,63479174	0,984760571	0	FALSE
67	85,05748329	-0,285733856	0,252386018	-1,132130293	0,257579661	0,917627542	0	FALSE
68	28,44212762	0,176353486	0,826624124	0,213341809	0,831060365	0,984760571	0	FALSE

Genus_nr	Phylum	Class	Order	Family	Genus	Species
69	Bacteroidota	Bacteroidia	Bacteroidales	Tannerellaceae	Parabacteroides	NA
70	Firmicutes	Clostridia	Oscillospirales	Ruminococcaceae	DTU089	NA
71	Firmicutes	Clostridia	Oscillospirales	Ruminococcaceae	Ruminococcus	NA
72	Firmicutes	Clostridia	Oscillospirales	Oscillospiraceae	Oscillospira	NA
73	Firmicutes	Clostridia	Lachnospirales	Lachnospiraceae	Blautia	NA
74	Firmicutes	Negativicutes	Veillonellales-Selenomonadales	Veillonellaceae	Veillonella	NA
75	Verrucomicrobiota	Lentisphaeria	Victivallales	Victivallaceae	Victivallis	NA
76	Bacteroidota	Bacteroidia	Bacteroidales	Marinifilaceae	Butyricimonas	NA
77	Firmicutes	Clostridia	Lachnospirales	Lachnospiraceae	Frisingicoccus	NA
78	Firmicutes	Clostridia	Oscillospirales	Oscillospiraceae	UCG-005	NA
79	Firmicutes	Clostridia	Lachnospirales	Lachnospiraceae	Howardella	NA
80	Firmicutes	Clostridia	Lachnospirales	Lachnospiraceae	Coprococcus	NA
81	Proteobacteria	Gammaproteobacteria	Burkholderiales	Oxalobacteraceae	Oxalobacter	NA
82	Firmicutes	Clostridia	Monoglobales	Monoglobaceae	Monoglobus	NA
83	Firmicutes	Negativicutes	Veillonellales-Selenomonadales	Veillonellaceae	Allisonella	NA
84	Firmicutes	Clostridia	Lachnospirales	Lachnospiraceae	Roseburia	NA
85	Firmicutes	Bacilli	Erysipelotrichales	Erysipelotrichaceae	Merdibacter	NA
86	Firmicutes	Clostridia	Oscillospirales	Ruminococcaceae	Faecalibacterium	NA
87	Firmicutes	Clostridia	Oscillospirales	Ruminococcaceae	[Eubacterium] siraeum group	NA
88	Firmicutes	Bacilli	Erysipelotrichales	Erysipelatoclostridiaceae	Catenibacterium	NA
89	Firmicutes	Clostridia	Oscillospirales	Ruminococcaceae	Incertae Sedis	NA
90	Firmicutes	Clostridia	Oscillospirales	Butyricicoccaceae	Butyricicoccus	NA
91	Firmicutes	Bacilli	Erysipelotrichales	Erysipelotrichaceae	Holdemanella	NA
92	Firmicutes	Clostridia	Oscillospirales	Ruminococcaceae	Angelakisella	NA
93	Firmicutes	Clostridia	Lachnospirales	Lachnospiraceae	Agathobacter	NA
94	Proteobacteria	Gammaproteobacteria	Burkholderiales	Sutterellaceae	Parasutterella	NA
95	Firmicutes	Bacilli	Erysipelotrichales	Erysipelotrichaceae	Holdemania	NA
96	Firmicutes	Clostridia	Lachnospirales	Lachnospiraceae	[Ruminococcus] gauvreauii group	NA
97	Firmicutes	Clostridia	Lachnospirales	Lachnospiraceae	Lachnospiraceae ND3007 group	NA
98	Firmicutes	Clostridia	Oscillospirales	Ruminococcaceae	Fournierella	NA
99	Firmicutes	Clostridia	Lachnospirales	Lachnospiraceae	[Ruminococcus] torques group	NA
100	Firmicutes	Clostridia	Lachnospirales	Lachnospiraceae	[Eubacterium] eligens group	NA
101	Firmicutes	Clostridia	Lachnospirales	Lachnospiraceae	[Eubacterium] ruminantium group	NA
102	Firmicutes	Clostridia	Oscillospirales	Butyricicoccaceae	UCG-009	NA
103	Firmicutes	Negativicutes	Acidaminococcales	Acidaminococcaceae	Phascolarctobacterium	NA
104	Firmicutes	Clostridia	Lachnospirales	Lachnospiraceae	Lachnospiraceae NK4A136 group	NA

Genus_nr	DESeq_baseMean	DESeq_log2FoldChange	DESeq_lfcSE	DESeq_stat	DESeq_pvalue	DESeq_padj	ANCOM_W	ANCOM_detected_0.7
69	901,9390753	0,131123935	0,326970738	0,401026513	0,688400604	0,984760571	0	FALSE
70	9,893652029	0,098335872	0,80252124	0,122533669	0,902476383	0,984760571	6	FALSE
71	1076,851417	0,037280476	0,357340126	0,104327708	0,91690929	0,984760571	0	FALSE
72	27,04166394	-0,622085274	0,693312723	-0,897265049	0,369577502	0,95754171	0	FALSE
73	448,5552221	-0,508420015	0,175027319	-2,904803765	0,003674834	0,133183807	3	FALSE
74	69,90522029	-0,533509782	0,781174579	-0,682958453	0,494633096	0,984760571	0	FALSE
75	20,33105584	-0,1876924	0,722795847	-0,259675537	0,795114064	0,984760571	0	FALSE
76	81,31027981	-0,283084525	0,524400019	-0,539825543	0,58931735	0,984760571	0	FALSE
77	13,46508959	-0,235525184	1,387023722	-0,169806168	0,865162576	0,984760571	1	FALSE
78	690,1291152	0,239797739	0,338641462	0,70811689	0,478872672	0,984760571	0	FALSE
79	5,043657078	0,251530756	1,14361951	0,219942694	0,825915785	0,984760571	0	FALSE
80	746,530487	0,023389876	0,374560921	0,062446119	0,950207569	0,984760571	21	FALSE
81	6,559860021	0,922971672	0,867239477	1,064263905	0,287209164	0,929393191	4	FALSE
82	128,2138726	0,340863952	0,335612771	1,015646548	0,30979773	0,929393191	4	FALSE
83	5,596718442	-0,136141982	1,830840745	-0,074360363	0,940723647	0,984760571	1	FALSE
84	588,9744955	-0,500981409	0,364328377	-1,375082043	0,169106011	0,884937089	82	TRUE
85	2,12156651	-1,060492945	1,518086438	-0,698572175	0,484819438	0,984760571	4	FALSE
86	2413,668911	0,309431992	0,207418658	1,491823321	0,135745468	0,884937089	12	FALSE
87	323,4656358	-0,189678637	0,444827555	-0,42640937	0,669809575	0,984760571	0	FALSE
88	18,35025176	1,411501628	1,947937964	0,724613234	0,468689338	0,984760571	7	FALSE
89	184,7176507	0,112181615	0,215035276	0,521689357	0,601886636	0,984760571	4	FALSE
90	147,3860336	-0,94596326	0,263534443	-3,589524196	0,000331282	0,037766157	49	FALSE
91	179,2881431	-0,420881236	1,323951384	-0,317897803	0,750562459	0,984760571	0	FALSE
92	12,74711901	-0,114223942	0,574594555	-0,198790506	0,842426623	0,984760571	0	FALSE
93	1266,427516	-0,584317764	0,399936559	-1,461026134	0,14400827	0,884937089	2	FALSE
94	243,2840938	0,037795374	0,544847037	0,069368779	0,94469608	0,984760571	0	FALSE
95	6,007070035	0,932444681	0,653558998	1,426718451	0,153661065	0,884937089	6	FALSE
96	19,63288332	-0,0940597	0,963881405	-0,097584308	0,922262385	0,984760571	0	FALSE
97	102,1603544	-0,173137548	0,44616689	-0,388055572	0,697974909	0,984760571	0	FALSE
98	8,66394602	-1,509871053	1,188754179	-1,270128912	0,204038714	0,884937089	0	FALSE
99	149,1295328	-0,597167076	0,435209907	-1,372135758	0,17002118	0,884937089	0	FALSE
100	536,1081195	-0,228456327	0,442988025	-0,515716711	0,606052282	0,984760571	0	FALSE
101	104,568101	-0,122246554	1,252169977	-0,097627763	0,922227878	0,984760571	0	FALSE
102	5,709587377	0,115855603	0,876624912	0,132160975	0,894856967	0,984760571	0	FALSE
103	329,0892046	0,39215712	0,730254245	0,537014502	0,591257597	0,984760571	0	FALSE
104	407,6261091	-0,327740911	0,399911765	-0,819533057	0,41248235	0,984760571	0	FALSE

Genus_nr	Phylum	Class	Order	Family	Genus	Species
105	Firmicutes	Clostridia	Oscillospirales	Ruminococcaceae	Anaerotruncus	NA
106	Firmicutes	Clostridia	Lachnospirales	Defluviitaleaceae	Defluviitaleaceae UCG-011	NA
107	Proteobacteria	Gammaproteobacteria	Burkholderiales	Sutterellaceae	Sutterella	NA
108	Firmicutes	Clostridia	Peptococcales	Peptococcaceae	Peptococcus	NA
109	Firmicutes	Bacilli	Erysipelotrichales	Erysipelotrichaceae	Turicibacter	NA
110	Firmicutes	Clostridia	Oscillospirales	Oscillospiraceae	NK4A214 group	NA
111	Firmicutes	Clostridia	Lachnospirales	Lachnospiraceae	Eisenbergiella	NA
112	Firmicutes	Clostridia	Lachnospirales	Lachnospiraceae	CAG-56	NA
113	Firmicutes	Clostridia	Lachnospirales	Lachnospiraceae	[Ruminococcus] gnavus group	NA
114	Firmicutes	Clostridia	Oscillospirales	Oscillospiraceae	Pseudoflavonifractor	NA

Genus_nr	DESeq_baseMean	DESeq_log2FoldChange	DESeq_lfcSE	DESeq_stat	DESeq_pvalue	DESeq_padj	ANCOM_W	ANCOM_detected_0.7
105	4,717744857	-1,088626836	1,653250421	-0,658476672	0,510231882	0,984760571	4	FALSE
106	13,13078688	0,7558132	0,642764228	1,175879378	0,239643081	0,910643706	4	FALSE
107	417,5121978	-1,088247707	0,534219643	-2,03707917	0,041642109	0,593400052	46	FALSE
108	7,924334045	-0,327591731	0,933621775	-0,350882702	0,725676349	0,984760571	0	FALSE
109	20,62996963	0,658429053	1,33801844	0,49209266	0,622653841	0,984760571	0	FALSE
110	494,9680119	0,380758327	0,34946114	1,089558417	0,275907709	0,929393191	0	FALSE
111	5,467651163	0,190192308	1,532192233	0,124130839	0,901211681	0,984760571	6	FALSE
112	60,02149746	-0,483212015	0,847725856	-0,570009764	0,568671076	0,984760571	0	FALSE
113	45,31518149	1,496864221	1,317789826	1,135889951	0,256002624	0,917627542	6	FALSE
114	2,59670799	-0,989471848	1,725561105	-0,573420348	0,566360117	0,984760571	0	FALSE

Supplementary table 7a: Differential abundance at family level in NL cohort with DESeq2 and ANCOM

All columns are spread out over two pages, after which the next two pages show the next set of rows, etc.

Families are numbered on both pages to easily connect the taxonomy data to the associated statistics.

Family_nr	Kingdom	Phylum	Class	Order	Family	Genus	Species
1	Bacteria	Actinobacteriota	Actinobacteria	Bifidobacteriales	Bifidobacteriaceae	NA	NA
2	Bacteria	Verrucomicrobiota	Verrucomicrobiae	Verrucomicrobiales	Akkermansiaceae	NA	NA
3	Bacteria	Synergistota	Synergistia	Synergistales	Synergistaceae	NA	NA
4	Bacteria	Firmicutes	Negativicutes	Veillonellales-Selenomonadales	Veillonellaceae	NA	NA
5	Bacteria	Bacteroidota	Bacteroidia	Bacteroidales	Muribaculaceae	NA	NA
6	Bacteria	Firmicutes	Clostridia	Christensenellales	Christensenellaceae	NA	NA
7	Bacteria	Proteobacteria	Gammaproteobacteria	Burkholderiales	Sutterellaceae	NA	NA
8	Bacteria	Firmicutes	Clostridia	Peptostreptococcales-Tissierellales	Peptostreptococcaceae	NA	NA
9	Bacteria	Desulfobacterota	Desulfovibrionia	Desulfovibrionales	Desulfovibrionaceae	NA	NA
10	Bacteria	Actinobacteriota	Coriobacteriia	Coriobacteriales	Coriobacteriaceae	NA	NA
11	Bacteria	Bacteroidota	Bacteroidia	Bacteroidales	Barnesiellaceae	NA	NA
12	Bacteria	Proteobacteria	Gammaproteobacteria	Pasteurellales	Pasteurellaceae	NA	NA
13	Bacteria	Actinobacteriota	Coriobacteriia	Coriobacteriales	Coriobacteriales Incertae Sedis	NA	NA
14	Bacteria	Firmicutes	Bacilli	Erysipelotrichales	Erysipelatoclostridiaceae	NA	NA
15	Bacteria	Bacteroidota	Bacteroidia	Bacteroidales	Marinifilaceae	NA	NA
16	Bacteria	Bacteroidota	Bacteroidia	Bacteroidales	Bacteroidaceae	NA	NA
17	Bacteria	Bacteroidota	Bacteroidia	Bacteroidales	Prevotellaceae	NA	NA
18	Bacteria	Firmicutes	Clostridia	Peptococcales	Peptococcaceae	NA	NA
19	Bacteria	Firmicutes	Negativicutes	Veillonellales-Selenomonadales	Selenomonadaceae	NA	NA
20	Bacteria	Firmicutes	Bacilli	Lactobacillales	Streptococcaceae	NA	NA
21	Bacteria	Firmicutes	Bacilli	Acholeplasmatales	Acholeplasmataceae	NA	NA
22	Archaea	Euryarchaeota	Methanobacteria	Methanobacteriales	Methanobacteriaceae	NA	NA
23	Bacteria	Firmicutes	Clostridia	Clostridiales	Clostridiaceae	NA	NA
24	Bacteria	Proteobacteria	Gammaproteobacteria	Enterobacterales	Enterobacteriaceae	NA	NA
25	Bacteria	Bacteroidota	Bacteroidia	Bacteroidales	Rikenellaceae	NA	NA
26	Bacteria	Firmicutes	Clostridia	Oscillospirales	Oscillospiraceae	NA	NA
27	Bacteria	Firmicutes	Clostridia	Peptostreptococcales-Tissierellales	Anaerovoracaceae	NA	NA
28	Bacteria	Firmicutes	Clostridia	Oscillospirales	UCG-010	NA	NA
29	Bacteria	Bacteroidota	Bacteroidia	Bacteroidales	Tannerellaceae	NA	NA
30	Bacteria	Firmicutes	Bacilli	Erysipelotrichales	Erysipelotrichaceae	NA	NA
31	Bacteria	Firmicutes	Bacilli	Lactobacillales	Lactobacillaceae	NA	NA
32	Bacteria	Actinobacteriota	Coriobacteriia	Coriobacteriales	Atopobiaceae	NA	NA

Family_nr	DESeq_baseMean	DESeq_log2FoldChange	DESeq_lfcSE	DESEq_stat	DESeq_pvalue	DESeq_padj	ANCOM_W	ANCOM_detected_0.7
1	791,3154088	-0,509159743	0,276630657	-1,840575979	0,065683721	0,507473708	1	FALSE
2	832,8822926	-0,019220728	0,407500358	-0,04716739	0,962379818	0,983744133	0	FALSE
3	5,459128106	0,231899149	1,298165093	0,178636099	0,858223447	0,962884843	0	FALSE
4	842,2984738	-0,546948696	0,484300163	-1,129358892	0,258746467	0,962884843	42	TRUE
5	476,9646176	-0,013909709	0,682681148	-0,020375118	0,983744133	0,983744133	1	FALSE
6	1291,335607	-0,231807214	0,240910505	-0,962212977	0,335942632	0,962884843	1	FALSE
7	230,6651383	-0,013632257	0,244098777	-0,055847299	0,955463455	0,983744133	0	FALSE
8	90,71412267	0,593313132	0,412273964	1,439123456	0,150115547	0,863164398	0	FALSE
9	245,421865	0,432078307	0,261117675	1,654726384	0,097980013	0,643868654	3	FALSE
10	176,9492198	-0,208438897	0,248911569	-0,837401403	0,402366977	0,962884843	0	FALSE
11	381,286812	-0,082212095	0,289535948	-0,283944345	0,776453032	0,962884843	0	FALSE
12	18,78428731	-0,278551037	0,665096195	-0,418813157	0,675352689	0,962884843	9	FALSE
13	28,39039757	0,289064128	0,69565929	0,415525434	0,6777573	0,962884843	0	FALSE
14	269,6315649	0,130980868	0,220298765	0,594560155	0,552137521	0,962884843	0	FALSE
15	71,60480907	0,138450394	0,194667453	0,711214902	0,476951075	0,962884843	0	FALSE
16	5106,339409	0,047392264	0,157510466	0,30088327	0,763503508	0,962884843	0	FALSE
17	1751,055617	0,155382276	0,487636255	0,318643814	0,749996625	0,962884843	0	FALSE
18	54,93708176	-0,338185673	0,351836377	-0,961201556	0,336450834	0,962884843	0	FALSE
19	100,0182935	-2,878839165	1,567039494	-1,837119725	0,066192223	0,507473708	0	FALSE
20	335,2061189	-0,378085252	0,363610569	-1,0398082	0,298429019	0,962884843	0	FALSE
21	23,3580212	-1,180168879	1,289911564	-0,914922319	0,360232412	0,962884843	0	FALSE
22	100,3574694	0,246953873	0,472770801	0,522354325	0,601423652	0,962884843	1	FALSE
23	115,3422544	-0,348833879	0,504989339	-0,690774738	0,489707113	0,962884843	0	FALSE
24	699,9963765	-0,137474408	0,587843042	-0,23386244	0,815091773	0,962884843	0	FALSE
25	1378,097359	-0,038731735	0,177462791	-0,218252707	0,82723222	0,962884843	0	FALSE
26	3231,690156	-0,023175064	0,115705555	-0,20029344	0,841251093	0,962884843	2	FALSE
27	112,7416783	-0,172786169	0,189400976	-0,912277077	0,361622878	0,962884843	0	FALSE
28	127,2044514	-0,214556517	0,333905209	-0,642567146	0,520505007	0,962884843	1	FALSE
29	616,8415503	0,418787815	0,18575417	2,254527118	0,024163027	0,279542428	8	FALSE
30	324,4754581	0,067305792	0,321631727	0,209263534	0,834242517	0,962884843	1	FALSE
31	107,9915249	-0,390411245	0,791956813	-0,492970373	0,622033522	0,962884843	0	FALSE
32	9,220105014	1,807892833	0,736067216	2,456151822	0,014043381	0,279542428	10	FALSE

Family_nr	Kingdom	Phylum	Class	Order	Family	Genus	Species
33	Bacteria	Verrucomicrobiota	Lentisphaeria	Victivallales	Victivallaceae	NA	NA
34	Bacteria	Actinobacteriota	Actinobacteria	Actinomycetales	Actinomycetaceae	NA	NA
35	Bacteria	Proteobacteria	Gammaproteobacteria	Burkholderiales	Oxalobacteraceae	NA	NA
36	Bacteria	Firmicutes	Clostridia	Monoglobales	Monoglobaceae	NA	NA
37	Bacteria	Firmicutes	Clostridia	Oscillospirales	[Eubacterium] coprostanoligenes group	NA	NA
38	Bacteria	Actinobacteriota	Coriobacteriia	Coriobacteriales	Eggerthellaceae	NA	NA
39	Bacteria	Firmicutes	Clostridia	Oscillospirales	Butyricicoccaceae	NA	NA
40	Bacteria	Firmicutes	Clostridia	Lachnospirales	Lachnospiraceae	NA	NA
41	Bacteria	Firmicutes	Clostridia	Oscillospirales	Hydrogenoanaerobacterium	NA	NA
42	Bacteria	Firmicutes	Clostridia	Oscillospirales	Ruminococcaceae	NA	NA
43	Bacteria	Verrucomicrobiota	Lentisphaeria	Victivallales	vadinBE97	NA	NA
44	Bacteria	Verrucomicrobiota	Verrucomicrobiae	Opitutales	Puniceicoccaceae	NA	NA
45	Bacteria	Firmicutes	Clostridia	Lachnospirales	Defluviitaleaceae	NA	NA
46	Bacteria	Firmicutes	Negativicutes	Acidaminococcales	Acidaminococcaceae	NA	NA

Family_nr	DESeq_baseMean	DESeq_log2FoldChange	DESeq_lfcSE	DESEq_stat	DESeq_pvalue	DESeq_padj	ANCOM_W	ANCOM_detected_0.7
33	8,478346981	0,786711997	0,709676236	1,108550572	0,267624111	0,962884843	3	FALSE
34	1,336305279	0,452532131	1,115156519	0,405801449	0,684888494	0,962884843	0	FALSE
35	10,67273893	0,283904462	0,421031695	0,674306626	0,500116392	0,962884843	6	FALSE
36	129,3327755	0,222430275	0,27346787	0,813368954	0,416006554	0,962884843	0	FALSE
37	390,5126163	-0,21120745	0,176009684	-1,199976304	0,230148543	0,962884843	0	FALSE
38	120,8610455	-0,058790077	0,200983692	-0,292511681	0,76989543	0,962884843	0	FALSE
39	221,5668429	-0,259748942	0,213837844	-1,214700531	0,22448033	0,962884843	18	FALSE
40	12433,68906	-0,298905561	0,124043613	-2,409681182	0,015966466	0,279542428	17	FALSE
41	4,261401008	0,335872095	1,16428453	0,288479394	0,772979802	0,962884843	3	FALSE
42	6597,2832	-0,235949748	0,104762941	-2,252225312	0,024308037	0,279542428	3	FALSE
43	8,1656645	-0,06502265	1,106753246	-0,05875081	0,953150589	0,983744133	1	FALSE
44	4,029293454	0,083806882	1,030224082	0,081348207	0,935165037	0,983744133	1	FALSE
45	1,617649477	0,816639308	1,224099228	0,667134893	0,504685974	0,962884843	2	FALSE
46	447,6357993	0,268417623	0,42415461	0,632829672	0,526844878	0,962884843	0	FALSE

Supplementary table 7b: Differential abundance at family level in FIN cohort with DESeq2 and ANCOM

All columns are spread out over two pages, after which the next two pages show the next set of rows, etc.

Families are numbered on both pages to easily connect the taxonomy data to the associated statistics.

Family_nr	Kingdom	Phylum	Class	Order	Family	Genus	Species
1	Bacteria	Actinobacteriota	Actinobacteria	Bifidobacteriales	Bifidobacteriaceae	NA	NA
2	Bacteria	Verrucomicrobiota	Verrucomicrobiae	Verrucomicrobiales	Akkermansiaceae	NA	NA
3	Bacteria	Synergistota	Synergistia	Synergistales	Synergistaceae	NA	NA
4	Bacteria	Firmicutes	Negativicutes	Veillonellales-Selenomonadales	Veillonellaceae	NA	NA
5	Bacteria	Bacteroidota	Bacteroidia	Bacteroidales	Barnesiellaceae	NA	NA
6	Bacteria	Bacteroidota	Bacteroidia	Bacteroidales	Muribaculaceae	NA	NA
7	Bacteria	Desulfobacterota	Desulfovibrionia	Desulfovibrionales	Desulfovibrionaceae	NA	NA
8	Bacteria	Firmicutes	Clostridia	Christensenellales	Christensenellaceae	NA	NA
9	Bacteria	Actinobacteriota	Coriobacteriia	Coriobacteriales	Coriobacteriaceae	NA	NA
10	Bacteria	Proteobacteria	Gammaproteobacteria	Pasteurellales	Pasteurellaceae	NA	NA
11	Bacteria	Actinobacteriota	Coriobacteriia	Coriobacteriales	Coriobacteriales Incertae Sedis	NA	NA
12	Bacteria	Firmicutes	Bacilli	Erysipelotrichales	Erysipelatoclostridiaceae	NA	NA
13	Bacteria	Bacteroidota	Bacteroidia	Bacteroidales	Marinifilaceae	NA	NA
14	Bacteria	Bacteroidota	Bacteroidia	Bacteroidales	Bacteroidaceae	NA	NA
15	Bacteria	Bacteroidota	Bacteroidia	Bacteroidales	Prevotellaceae	NA	NA
16	Bacteria	Bacteroidota	Bacteroidia	Bacteroidales	Porphyromonadaceae	NA	NA
17	Bacteria	Firmicutes	Bacilli	Lactobacillales	Streptococcaceae	NA	NA
18	Bacteria	Firmicutes	Bacilli	Acholeplasmatales	Acholeplasmataceae	NA	NA
19	Archaea	Euryarchaeota	Methanobacteria	Methanobacteriales	Methanobacteriaceae	NA	NA
20	Bacteria	Firmicutes	Clostridia	Peptostreptococcales-Tissierellales	Anaerovoracaceae	NA	NA
21	Bacteria	Patescibacteria	Saccharimonadia	Saccharimonadales	Saccharimonadaceae	NA	NA
22	Bacteria	Firmicutes	Clostridia	Clostridiales	Clostridiaceae	NA	NA
23	Bacteria	Firmicutes	Clostridia	Oscillospirales	Ruminococcaceae	NA	NA
24	Bacteria	Proteobacteria	Gammaproteobacteria	Enterobacterales	Enterobacteriaceae	NA	NA
25	Bacteria	Bacteroidota	Bacteroidia	Bacteroidales	Rikenellaceae	NA	NA
26	Bacteria	Firmicutes	Bacilli	Lactobacillales	Lactobacillaceae	NA	NA
27	Bacteria	Firmicutes	Clostridia	Oscillospirales	Oscillospiraceae	NA	NA
28	Bacteria	Firmicutes	Clostridia	Peptostreptococcales-Tissierellales	Peptostreptococcaceae	NA	NA
29	Bacteria	Firmicutes	Clostridia	Oscillospirales	UCG-010	NA	NA
30	Bacteria	Bacteroidota	Bacteroidia	Bacteroidales	Tannerellaceae	NA	NA
31	Bacteria	Actinobacteriota	Coriobacteriia	Coriobacteriales	Atopobiaceae	NA	NA
32	Bacteria	Verrucomicrobiota	Lentisphaeria	Victivallales	Victivallaceae	NA	NA

Family_nr	DESeq_baseMean	DESeq_log2FoldChange	DESeq_lfcSE	DESEq_stat	DESeq_pvalue	DESeq_padj	ANCOM_W	ANCOM_detected_0.7
1	746,033086	0,512476455	0,402337563	1,273747474	0,202752926	0,567512029	0	FALSE
2	716,3578344	0,560586461	0,514161503	1,09029256	0,275584292	0,645442025	4	FALSE
3	3,260601615	1,540142477	1,615310429	0,953465321	0,340354359	0,645442025	3	FALSE
4	715,0513866	-0,294573142	0,502450031	-0,586273506	0,557691732	0,748900326	0	FALSE
5	569,0479169	0,42538842	0,476852511	0,892075452	0,372352492	0,645442025	0	FALSE
6	637,0540221	-0,060316325	1,201645822	-0,050194761	0,959967187	0,980836038	0	FALSE
7	190,7061027	0,188072883	0,369069929	0,509586039	0,610341507	0,785498686	0	FALSE
8	1789,851873	-0,098280983	0,370226847	-0,265461525	0,790653945	0,906359401	0	FALSE
9	55,59045912	0,297657836	0,416877257	0,714017929	0,475216093	0,697973637	0	FALSE
10	127,8476547	1,457204194	0,805958745	1,808038193	0,070600556	0,366187817	0	FALSE
11	12,06260587	-2,10536625	1,004447721	-2,096043634	0,03607832	0,28261351	0	FALSE
12	284,7121071	-0,266233628	0,326783268	-0,814710097	0,415238272	0,645442025	1	FALSE
13	202,9494748	0,395439856	0,325718133	1,214055393	0,224726572	0,584915735	0	FALSE
14	9956,572761	0,469335867	0,192692278	2,435675535	0,014864011	0,174652126	3	FALSE
15	2043,198872	-0,8495145	0,837731805	-1,014064996	0,310551739	0,645442025	0	FALSE
16	1,145111591	-1,570804865	1,661301975	-0,945526394	0,344390203	0,645442025	1	FALSE
17	122,2171794	-0,144758904	0,433842821	-0,333666705	0,738631077	0,867891515	0	FALSE
18	81,04069127	-5,692532804	1,852642409	-3,072655994	0,002121629	0,033238856	0	FALSE
19	90,31080823	1,345037063	0,767585967	1,752295015	0,079723093	0,366187817	0	FALSE
20	70,25625087	-0,238926028	0,297190904	-0,803947982	0,421427017	0,645442025	0	FALSE
21	0,658794391	-0,011834909	1,473416241	-0,008032292	0,993591227	0,993591227	3	FALSE
22	114,5033915	0,106141572	0,735579495	0,144296534	0,885266315	0,924611484	0	FALSE
23	7133,90848	-0,034451126	0,147781964	-0,233121317	0,815667202	0,912770441	2	FALSE
24	643,68558	-1,196839714	0,712051919	-1,680832088	0,092795534	0,366187817	0	FALSE
25	1923,394831	0,123898307	0,249488991	0,496608314	0,619465289	0,785498686	0	FALSE
26	23,55258573	0,254604782	1,360292706	0,187169115	0,851528039	0,924611484	0	FALSE
27	3245,987101	0,132116896	0,152535693	0,866137579	0,38641472	0,645442025	1	FALSE
28	56,22550352	-0,829962713	0,655227654	-1,266678395	0,205270308	0,567512029	0	FALSE
29	192,0768532	-0,555860089	0,469518733	-1,183893314	0,236455297	0,584915735	0	FALSE
30	950,7984127	0,225282807	0,282826028	0,796541989	0,42571708	0,645442025	0	FALSE
31	1,641035727	-2,065660295	1,457260661	-1,417495408	0,156338136	0,524849457	0	FALSE
32	29,92932593	-0,673903672	0,696895306	-0,967008482	0,333539793	0,645442025	0	FALSE

Family_nr	Kingdom	Phylum	Class	Order	Family	Genus	Species
33	Bacteria	Proteobacteria	Gammaproteobacteria	Burkholderiales	Oxalobacteraceae	NA	NA
34	Bacteria	Firmicutes	Clostridia	Monoglobales	Monoglobaceae	NA	NA
35	Bacteria	Firmicutes	Clostridia	Hungateiclostridiaceae	Ruminiclostridium	NA	NA
36	Bacteria	Actinobacteriota	Coriobacteriia	Coriobacteriales	Eggerthellaceae	NA	NA
37	Bacteria	Firmicutes	Clostridia	Oscillospirales	Butyricicoccaceae	NA	NA
38	Bacteria	Firmicutes	Bacilli	Erysipelotrichales	Erysipelotrichaceae	NA	NA
39	Bacteria	Firmicutes	Clostridia	Lachnospirales	Lachnospiraceae	NA	NA
40	Bacteria	Firmicutes	Clostridia	Oscillospirales	[Eubacterium] coprostanoligenes group	NA	NA
41	Bacteria	Proteobacteria	Gammaproteobacteria	Burkholderiales	Sutterellaceae	NA	NA
42	Bacteria	Firmicutes	Clostridia	Oscillospirales	Hydrogenoanaerobacterium	NA	NA
43	Bacteria	Firmicutes	Clostridia	Peptococcales	Peptococcaceae	NA	NA
44	Bacteria	Firmicutes	Negativicutes	Acidaminococcales	Acidaminococcaceae	NA	NA
45	Bacteria	Verrucomicrobiota	Lentisphaeria	Victivallales	vadinBE97	NA	NA
46	Bacteria	Verrucomicrobiota	Verrucomicrobiae	Opitutales	Puniceicoccaceae	NA	NA
47	Bacteria	Firmicutes	Clostridia	Lachnospirales	Defluviitaleaceae	NA	NA

Family_nr	DESeq_baseMean	DESeq_log2FoldChange	DESeq_lfcSE	DESEq_stat	DESeq_pvalue	DESeq_padj	ANCOM_W	ANCOM_detected_0.7
33	6,038488066	0,652661722	0,791911049	0,824160393	0,409848428	0,645442025	1	FALSE
34	135,0670696	0,581513565	0,339917696	1,710748136	0,087127617	0,366187817	1	FALSE
35	11,3580818	0,772600002	1,217156316	0,634758241	0,525586102	0,740944072	3	FALSE
36	38,91509177	1,076384463	0,324863874	3,313339983	0,000921888	0,021664378	9	FALSE
37	164,4762331	-0,978595584	0,243342106	-4,0214807	5,78334E-05	0,002718172	22	FALSE
38	215,3175462	-0,558194166	0,414045151	-1,348148056	0,177610768	0,556513739	0	FALSE
39	6758,722155	-0,361086372	0,156163333	-2,3122353	0,02076472	0,195188368	11	FALSE
40	578,2715749	0,411757251	0,251278206	1,638650868	0,101285992	0,366187817	1	FALSE
41	711,6880728	-0,606330534	0,300822022	-2,015578945	0,043844023	0,294381299	19	FALSE
42	4,723868665	1,14795148	1,410228241	0,814018218	0,415634514	0,645442025	0	FALSE
43	21,21536744	-0,195978358	0,510888569	-0,383602943	0,701272759	0,845123581	0	FALSE
44	411,5632828	0,115520221	0,719982118	0,160448736	0,872527601	0,924611484	0	FALSE
45	15,27516372	-0,649542845	1,049562899	-0,618869861	0,536002095	0,740944072	0	FALSE
46	12,94985188	-0,493435146	1,039723044	-0,474583254	0,635084044	0,785498686	0	FALSE
47	14,28304543	1,075834886	0,652037263	1,649959206	0,09895128	0,366187817	0	FALSE

Supplementary table 8A: PERMANOVA (univariable) of DNA extraction method in samples processed in parallel using both the NL (Qiagen Allprep) and FIN (PSP Spin Stool Kit) method

variable	Df	SumOfSqs	R2	F	Pr(>F)
DNA-extraction-method	1	5886,687375	0,019783064	1,856774555	1,00E-04
Residual	92	291675,2802	0,980216936	NA	NA
Total	93	297561,9676	1	NA	NA

Supplementary table 8B: Taxa identified by ANCOM as statistically significantly differentially abundant between DNA extraction methods in samples processed in parallel using both the NL (Qiagen Allprep) and FIN (PSP Spin Stool Kit) method, at ASV, genus and family level.

taxLevel	Phylum	Family	Genus	Species
ASV	Firmicutes	Lachnospiraceae	Blautia	NA
ASV	Firmicutes	Lachnospiraceae	Fusicatenibacter	saccharivorans
ASV	Firmicutes	Lachnospiraceae	Blautia	massiliensis
ASV	Firmicutes	Peptostrepto-coccaceae	Romboutsia	NA
ASV	Firmicutes	Lachnospiraceae	Dorea	longicatena
ASV	Firmicutes	Lachnospiraceae	Anaerostipes	hadrus
ASV	Firmicutes	Lachnospiraceae	Blautia	obeum
ASV	Firmicutes	Lachnospiraceae	[Eubacterium] hallii group	NA
ASV	Bacteroidota	Bacteroidaceae	Bacteroides	NA
ASV	Firmicutes	Lachnospiraceae	[Ruminococcus] torques group	NA
ASV	Actino-bacteriota	Eggerthellaceae	NA	NA
ASV	Firmicutes	Lachnospiraceae	Coprococcus	catus
ASV	Firmicutes	Lachnospiraceae	[Ruminococcus] gauvreauii group	NA
ASV	Firmicutes	Lachnospiraceae	[Eubacterium] hallii group	NA
ASV	Firmicutes	Lachnospiraceae	Dorea	formicigenerans
ASV	Firmicutes	Lachnospiraceae	Blautia	NA
ASV	Firmicutes	Lachnospiraceae	NA	NA

ASV	Firmicutes	Lachnospiraceae	[Eubacterium] ventriosum group	NA
ASV	Firmicutes	Lachnospiraceae	Roseburia	NA
ASV	Firmicutes	Lachnospiraceae	Lachnospiraceae FCS020 group	NA
ASV	Firmicutes	Anaerovoracaceae	Family XIII AD3011 group	NA
ASV	Firmicutes	Lachnospiraceae	Marvinbryantia	NA
Genus	Firmicutes	Lachnospiraceae	Blautia	NA
Genus	Firmicutes	Lachnospiraceae	Fusicatenibacter	NA
Genus	Firmicutes	Peptostrepto-coccaceae	Romboutsia	NA
Genus	Firmicutes	Lachnospiraceae	Dorea	NA
Genus	Firmicutes	Lachnospiraceae	Anaerostipes	NA
Genus	Firmicutes	Lachnospiraceae	[Eubacterium] hallii group	NA
Genus	Firmicutes	Lachnospiraceae	[Ruminococcus] torques group	NA
Genus	Proteobacteria	Sutterellaceae	Sutterella	NA
Genus	Firmicutes	Lachnospiraceae	[Eubacterium] ventriosum group	NA
Genus	Firmicutes	Lachnospiraceae	Lachnospiraceae FCS020 group	NA
Genus	Firmicutes	Lachnospiraceae	Marvinbryantia	NA
Genus	Verruco-microbiota	Victivallaceae	Victivallis	NA
Family	Firmicutes	Peptostrepto-coccaceae	NA	NA
Family	Actino-bacteriota	Eggerthellaceae	NA	NA
Family	Proteobacteria	Sutterellaceae	NA	NA
Family	Verruco-microbiota	Victivallaceae	NA	NA

Supplementary table 8C: Taxa identified by DESeq as statistically significantly differentially abundant between DNA extraction methods in samples processed in parallel using both the NL (Qiagen Allprep) and FIN (PSP Spin Stool Kit) method, at ASV, genus and family level.

taxLevel	baseMean	log2FoldChange	lfcSE	stat	pvalue	padj	Phylum	Family	Genus	Species
ASV	428,872091	2,967983592	0,3551975	8,355868474	6,50E-17	1,54E-14	Firmicutes	Lachnospiraceae	Dorea	longicatena
ASV	61,20187544	4,149655759	0,533740905	7,774663174	7,56E-15	8,96E-13	Firmicutes	Lachnospiraceae	[Eubacterium] hallii group	NA
ASV	535,4229464	2,411428946	0,369951274	6,518233923	7,11E-11	5,62E-09	Firmicutes	Lachnospiraceae	Fusicatenibacter	saccharivorans
ASV	20,06095287	6,274266308	1,011957612	6,20012759	5,64E-10	3,34E-08	Firmicutes	Lachnospiraceae	[Eubacterium] hallii group	NA
ASV	61,21591777	2,76841206	0,452780968	6,114241224	9,70E-10	4,60E-08	Firmicutes	Lachnospiraceae	Coprococcus	catus
ASV	18,86533323	6,611018111	1,160436061	5,697011952	1,22E-08	4,82E-07	Firmicutes	Lachnospiraceae	Marvinbryantia	NA
ASV	252,5401103	1,777418487	0,318044405	5,588585939	2,29E-08	7,75E-07	Firmicutes	Lachnospiraceae	Blautia	NA
ASV	245,7453024	2,05083448	0,389204493	5,269298056	1,37E-07	3,79E-06	Firmicutes	Lachnospiraceae	Blautia	massiliensis
ASV	39,37562833	4,674431822	0,888621442	5,260318512	1,44E-07	3,79E-06	Firmicutes	Lachnospiraceae	Lachnospiraceae FCS020 group	NA
ASV	117,2258425	3,427170952	0,737729704	4,645564541	3,39E-06	8,04E-05	Firmicutes	Lachnospiraceae	[Ruminococcus] torques group	NA
ASV	131,8538661	3,161753396	0,741573017	4,263576644	2,01E-05	0,000433452	Firmicutes	Lachnospiraceae	NA	NA
ASV	48,04581306	2,171456325	0,530764894	4,091183028	4,29E-05	0,000847627	Actino-bacteriota	Eggerthellaceae	NA	NA
ASV	76,41865568	2,164052526	0,586395993	3,690428573	0,000223877	0,003959623	Firmicutes	Lachnospiraceae	Anaerostipes	hadrus
ASV	45,15433239	3,187682109	0,866389643	3,679270793	0,000233902	0,003959623	Firmicutes	Lachnospiraceae	Blautia	NA
ASV	192,3223115	2,314540231	0,711304077	3,253939214	0,001138166	0,017983028	Firmicutes	Christensenellaceae	NA	NA
ASV	46,98472758	3,302243398	1,054375891	3,131941298	0,001736546	0,025722587	Firmicutes	Lachnospiraceae	Roseburia	NA
ASV	27,96653123	3,297194844	1,071315607	3,077706348	0,002086004	0,029081344	Firmicutes	Lachnospiraceae	Dorea	formicigenerans
ASV	1086,052624	1,683940731	0,554872012	3,034827301	0,002406735	0,031688672	Firmicutes	Lachnospiraceae	Agathobacter	NA
ASV	37,34393818	2,215248419	0,754707402	2,935241411	0,003332883	0,041573327	Firmicutes	Lachnospiraceae	[Eubacterium] ventriosum group	NA
ASV	75,56284907	1,462138269	0,503040766	2,90659996	0,003653801	0,043297546	Firmicutes	Erysipelato-clostridiaceae	Erysipelotricha-ceae UCG-003	bacterium
ASV	31,58174162	2,375640396	0,832004145	2,855322788	0,004299311	0,048520794	Firmicutes	Peptostrepto-coccaceae	Romboutsia	NA
Genus	84,73861152	4,372933779	0,437282986	10,00023765	1,52E-23	1,84E-21	Firmicutes	Lachnospiraceae	[Eubacterium] hallii group	NA
Genus	440,0183409	2,750130363	0,316752258	8,682275489	3,88E-18	2,35E-16	Firmicutes	Lachnospiraceae	Dorea	NA
Genus	810,5423302	1,464377525	0,208613456	7,019573671	2,23E-12	8,98E-11	Firmicutes	Lachnospiraceae	Blautia	NA
Genus	503,6388874	2,238221013	0,357062317	6,26843244	3,65E-10	1,10E-08	Firmicutes	Lachnospiraceae	Fusicatenibacter	NA
Genus	38,97238176	4,571924894	0,745723709	6,130856293	8,74E-10	2,12E-08	Firmicutes	Lachnospiraceae	Lachnospiraceae FCS020 group	NA

Genus	5898,2941	-0,998179549	0,176758669	-5,647132069	1,63E-08	3,29E-07	Bacte-roidota	Bacteroidaceae	Bacteroides	NA
Genus	359,5193788	-2,112238218	0,447509866	-4,719981347	2,36E-06	4,08E-05	Proteo-bacteria	Sutterellaceae	Sutterella	NA
Genus	1449,174246	-1,000744595	0,22694519	-4,409631213	1,04E-05	0,000156615	Firmicutes	Ruminococcaceae	Subdoligranulum	NA
Genus	76,99239517	2,65884708	0,619866648	4,289385612	1,79E-05	0,000240882	Firmicutes	Lachnospiraceae	[Eubacterium] ventriosum group	NA
Genus	737,2833665	-0,971174966	0,238470263	-4,07252022	4,65E-05	0,000562737	Bacte-roidota	Tannerellaceae	Parabacteroides	NA
Genus	26,49018211	-2,784659969	0,704725085	-3,951413149	7,77E-05	0,000854602	Verruco-microbiota	Victivallaceae	Victivallis	NA
Genus	656,7768375	-1,086719797	0,298844873	-3,636401007	0,000276474	0,002787778	Firmicutes	Oscillospiraceae	NK4A214 group	NA
Genus	1366,466541	1,240344492	0,366140276	3,387621013	0,000705016	0,006562073	Firmicutes	Lachnospiraceae	Agathobacter	NA
Genus	51,98516263	-1,056822311	0,323494665	-3,266892554	0,001087349	0,009397806	Bacte-roidota	Marinifilaceae	Odoribacter	NA
Genus	3,048307049	4,55982469	1,475979425	3,089355186	0,002005915	0,016181045	Firmicutes	Lachnospiraceae	Shuttleworthia	NA
Genus	326,0052196	1,408565105	0,462991573	3,04231262	0,002347679	0,017572421	Firmicutes	Lachnospiraceae	[Ruminococcus] torques group	NA
Genus	83,99141032	-1,373584697	0,45375762	-3,02713307	0,002468852	0,017572421	Bacte-roidota	Marinifilaceae	Butyricimonas	NA
Genus	32,99667404	2,165698781	0,74840403	2,893756172	0,003806636	0,025589055	Firmicutes	Peptostrepto-coccaceae	Romboutsia	NA
Genus	23,6091585	-3,591977961	1,256265931	-2,85924968	0,004246444	0,027043144	Firmicutes	Erysipelato-clostridiaceae	UCG-004	NA
Genus	16,8348102	-1,669440185	0,588346835	-2,837510267	0,004546688	0,027507465	Bacte-roidota	Barnesiellaceae	Coprobacter	NA
Genus	103,4916098	1,466753222	0,539725727	2,717589969	0,006575927	0,037889868	Firmicutes	Lachnospiraceae	Anaerostipes	NA
Genus	61,86396734	-1,229034256	0,463990209	-2,648836621	0,008076937	0,042844684	Firmicutes	Lachnospiraceae	Lachnospiraceae ND3007 group	NA
Genus	79,97513934	-1,927559831	0,728469789	-2,646039494	0,008144031	0,042844684	Proteo-bacteria	Sutterellaceae	Parasutterella	NA
Genus	70,90755424	1,273570955	0,489028614	2,60428719	0,009206558	0,046416394	Firmicutes	Erysipelato-clostridiaceae	Erysipelotricha-ceae UCG-003	NA
Family	75,77841059	1,960221806	0,297280085	6,593855115	4,29E-11	1,93E-09	Actino-bacteriota	Eggerthellaceae	NA	NA
Family	8935,623831	1,262154847	0,202166004	6,24316068	4,29E-10	9,65E-09	Firmicutes	Lachnospiraceae	NA	NA
Family	421,7757539	-1,715046594	0,318755702	-5,380442081	7,43E-08	1,11E-06	Proteo-bacteria	Sutterellaceae	NA	NA

Family	67,32924584	2,64389124	0,604801141	4,371505044	1,23E-05	0,000138817	Firmicutes	Peptostrepto-coccaceae	NA	NA
Family	30,54433436	-2,677264504	0,649021543	-4,125078022	3,71E-05	0,000333548	Verruco-microbiota	Victivallaceae	NA	NA
Family	5823,217819	-0,709893083	0,182870361	-3,881947177	0,000103623	0,000777175	Bacte-roidota	Bacteroidaceae	NA	NA
Family	106,4727207	1,033201119	0,268873094	3,842709221	0,000121684	0,000782252	Firmicutes	Anaerovoracaceae	NA	NA
Family	129,9282005	-1,020693841	0,306014291	-3,335445014	0,00085163	0,004790418	Bacte-roidota	Marinifilaceae	NA	NA
Family	659,3745528	-0,832828677	0,261823486	-3,18087842	0,001468292	0,007341462	Firmicutes	[Eubacterium] coprostanoligenes group	NA	NA
Family	67,34563749	1,358887553	0,468072418	2,903156653	0,003694218	0,015334294	Firmicutes	Peptococcaceae	NA	NA
Family	6,288003893	-3,02249417	1,042744428	-2,898595368	0,003748383	0,015334294	Verruco-microbiota	vadinBE97	NA	NA
Family	756,3137611	-0,63542341	0,223586553	-2,84195718	0,004483751	0,016275328	Bacte-roidota	Tannerellaceae	NA	NA
Family	235,0875626	-0,992013468	0,350933241	-2,826786839	0,004701761	0,016275328	Firmicutes	UCG-010	NA	NA
Family	6176,524879	0,353763814	0,144061096	2,455651273	0,014062955	0,045202356	Firmicutes	Ruminococcaceae	NA	NA

Supplementary table 9A: PERMANOVA (univariable) results of samehousehold stratification

Samehousehold cases and controls (45 cases and 45 controls of which each case-control pair is from the same household)

	Df	SumOfSqs	R2	F	Pr(>F)
group	1	3412,647255	0,012017711	1,070422554	0,221477852
Residual	88	280555,5221	0,987982289	NA	NA
Total	89	283968,1694	1	NA	NA

Different household: the 45 same household controls from above PERMANOVA were matched according to age and sex to 45 PD subjects who did not have someone from their household participate as a control subject in the study

	Df	SumOfSqs	R2	F	Pr(>F)
group	1	3377,819272	0,012339635	1,099454737	0,135086491
Residual	88	270359,5572	0,987660365	NA	NA
Total	89	273737,3765	1	NA	NA

Supplementary table 9B: Taxa identified by ANCOM and DESEq as statistically significantly differentially abundant at ASV, genus and family level when stratifying samehousehold controls

Samehousehold cases and controls (45 cases and 45 controls of which each case-control pair is from the same household)

ANCOM

taxLevel	Phylum	Order	Family	Genus	Species
ASV	Firmicutes	Lachnospirales	Lachnospiraceae	Lachnoclostridium	NA

DESeq

taxLevel	baseMean	log2FoldChange	lfcSE	stat	pvalue	padj	Phylum	Order	Family	Genus	Species
ASV	15,16852695	24,35085407	1,89201414	12,87033408	6,61E-38	2,39E-35	Firmicutes	Clostridia UCG-014	NA	NA	NA
ASV	19,66922115	6,882288219	1,805060736	3,812773766	0,000137416	0,024872275	Firmicutes	Clostridia UCG-014	NA	NA	NA
Genus	23,86956334	-25,00895145	2,127513273	-11,75501547	6,65E-32	7,99E-30	Firmicutes	Acidaminococcales	Acidaminococcaceae	Acidamino-coccus	NA

Different household: the 45 same household controls from above differential abundance analysis were matched according to age and sex to 45 PD subjects who did not have someone from their household participate as a control subject in the study

ANCOM

taxLevel	Kingdom	Phylum	Order	Family	Genus	Species	ASV
Family	Bacteria	Firmicutes	Veillonellales-Selenomonadales	Veillonellaceae	NA	NA	ASV12

DESeq

taxLevel	baseMean	log2FoldChange	lfcSE	stat	pvalue	padj	Phylum	Order	Family	Genus	Species
Species	42,40068099	25,88693171	1,968648131	13,1495981	1,71E-39	1,27E-37	Bacteroidota	Bacteroidales	Bacteroidaceae	Bacteroides	coprocola
Family	743,9686291	-1,37457947	0,369892918	-3,716155146	0,000202277	0,009304761	Actino-bacteriota	Bifidobacteriales	Bifidobacteriaceae	NA	NA

Supplementary table 10: overview of lost reads for all four sequencing runs. Columns represent the number of reads in the raw data, the output after using cutadapt, the input used for DADA2, after quality trimming and filtering, after denoising the forward reads, after denoising the reverse reads, after merging and in the eventual sequence table, respectively. Last column represents the percentage of lost reads at the end of the pipeline.

Sequencing run 1

sampleID	type	rawData	output-Cutadapt	input-DADA2	filtered	denoisedF	denoisedR	merged	seqtab	percLost
blank_control_1	PCR_control	10	10	10	7	2	1	0	0	100
blank_control_2	PCR_control	14	14	14	10	1	1	0	0	100
blank_control_3	PCR_control	11	11	11	9	1	1	0	0	100
blank_control_4	PCR_control	12	12	12	11	1	1	0	0	100
blank_control_5	PCR_control	13	13	13	9	1	1	0	0	100
blank_control_6	PCR_control	8	8	8	5	2	1	0	0	100
blank_control_7	PCR_control	11	11	11	9	1	1	0	0	100
blank_control_8	PCR_control	17	17	17	10	1	1	0	0	100
blank_control_9	PCR_control	11	11	11	9	3	1	0	0	100
blank_control_10	PCR_control	5	5	5	4	1	1	0	0	100
blank_control_11	PCR_control	14	14	14	6	1	1	0	0	100
blank_control_12	PCR_control	6	6	6	5	1	1	0	0	100
blank_control_13	PCR_control	4	4	4	4	1	1	0	0	100
blank_control_14	PCR_control	6	6	6	5	1	1	0	0	100
blank_control_15	PCR_control	13	13	13	10	2	1	0	0	100
blank_control_18	DNA_extraction_control	95	95	95	77	1	1	0	0	100
blank_control_19	DNA_extraction_control	87	87	87	74	14	1	0	0	100
blank_control_20	DNA_extraction_control	76	76	76	69	24	1	0	0	100
blank_control_21	DNA_extraction_control	16	16	16	13	1	1	0	0	100
blank_control_22	DNA_extraction_control	19	19	19	17	3	1	0	0	100
blank_control_23	DNA_extraction_control	61	61	61	52	5	1	0	0	100
blank_control_24	DNA_extraction_control	65	65	65	57	5	1	0	0	100
blank_control_25	DNA_extraction_control	85	85	85	73	6	1	0	0	100
blank_control_26	DNA_extraction_control	69	69	69	59	11	10	0	0	100
blank_control_27	DNA_extraction_control	73	73	73	62	19	1	0	0	100
blank_control_28	DNA_extraction_control	68	68	68	54	8	1	0	0	100
blank_control_29	DNA_extraction_control	92	92	92	78	18	1	0	0	100
blank_control_30	DNA_extraction_control	60	60	60	54	2	1	0	0	100
blank_control_31	DNA_extraction_control	66	66	66	57	9	1	0	0	100
blank_control_32	DNA_extraction_control	72	72	72	63	14	1	0	0	100
blank_control_33	DNA_extraction_control	72	72	72	64	8	1	0	0	100
blank_control_34	DNA_extraction_control	91	91	91	81	13	1	0	0	100
blank_control_35	DNA_extraction_control	49	49	49	42	1	1	0	0	100
blank_control_36	DNA_extraction_control	80	80	80	71	15	1	0	0	100
blank_control_37	DNA_extraction_control	99	99	99	87	16	1	0	0	100
blank_control_38	DNA_extraction_control	46	46	46	41	1	1	0	0	100
blank_control_39	DNA_extraction_control	20	20	20	16	2	1	0	0	100
blank_control_40	DNA_extraction_control	12	12	12	11	2	1	0	0	100
blank_control_41	DNA_extraction_control	110	110	110	97	40	51	23	23	79,091
blank_control_42	DNA_extraction_control	130	130	130	121	73	45	18	18	86,154
blank_control_43	DNA_extraction_control	42	42	42	38	3	1	0	0	100
blank_control_44	DNA_extraction_control	52	52	52	45	3	1	0	0	100
blank_control_45	DNA_extraction_control	67	67	67	60	1	1	0	0	100
blank_control_46	DNA_extraction_control	58	58	58	50	14	1	0	0	100
blank_control_47	DNA_extraction_control	33	33	33	31	6	10	4	4	87,879
blank_control_48	DNA_extraction_control	121	121	121	109	9	21	2	2	98,347
blank_control_49	DNA_extraction_control	41	41	41	35	1	1	0	0	100
blank_control_50	DNA_extraction_control	63	63	63	56	10	1	0	0	100
sample_1	sample	13624	13624	13624	12719	12305	11998	10855	10855	20,324
sample_2	sample	15089	15089	15089	14036	13578	13088	11593	11593	23,169
sample_3	sample	12095	12095	12095	11265	10822	10373	8626	8626	28,681
sample_4	sample	12440	12440	12440	11571	11179	10783	9361	9361	24,751
sample_5	sample	12972	12972	12972	12068	11520	11235	9544	9544	26,426
sample_6	sample	17365	17365	17365	16163	15591	15089	12936	12936	25,505
sample_7	sample	17283	17283	17283	16124	15598	15052	13078	13078	24,33
sample_8	sample	10159	10159	10159	9515	9125	8846	7637	7637	24,825
sample_9	sample	14287	14287	14287	13283	12821	12461	10949	10949	23,364
sample_10	sample	20058	20058	20058	18644	18033	17432	15451	15451	22,968

sample_11	sample	15357	15357	15357	14372	13874	13408	11725	11725	23,65
sample_12	sample	11306	11306	11306	10545	10188	9858	8602	8602	23,917
sample_13	sample	13212	13212	13212	12200	11708	11313	9649	9649	26,968
sample_14	sample	11339	11339	11339	10561	10253	9938	8948	8948	21,087
sample_15	sample	19110	19110	19110	17594	16878	16361	14093	14093	26,253
sample_16	sample	15118	15118	15118	14124	13568	13184	11239	11239	25,658
sample_17	sample	7800	7800	7800	7252	7011	6791	6158	6158	21,051
sample_18	sample	15681	15681	15681	14641	14089	13651	11677	11677	25,534
sample_19	sample	10026	10026	10026	9345	8959	8572	7288	7288	27,309
sample_20	sample	13387	13387	13387	12463	11988	11646	9733	9733	27,295
sample_21	sample	13034	13034	13034	12059	11690	11214	10164	10164	22,019
sample_22	sample	19957	19957	19957	18602	18043	17409	14303	14303	28,331
sample_23	sample	16570	16570	16570	15500	15088	14390	12754	12754	23,03
sample_24	sample	11143	11143	11143	10316	9969	9638	8402	8402	24,598
sample_25	sample	15625	15625	15625	14547	14036	13562	11759	11759	24,742
sample_26	sample	15693	15693	15693	14604	14104	13623	11578	11578	26,222
sample_27	sample	13305	13305	13305	12377	11942	11523	9905	9905	25,554
sample_28	sample	16289	16289	16289	15195	14622	14265	12471	12471	23,439
sample_29	sample	10377	10377	10377	9656	9333	9013	8021	8021	22,704
sample_30	sample	17933	17933	17933	16772	16376	15832	14067	14067	21,558
sample_31	sample	10870	10870	10870	10145	9818	9506	8504	8504	21,766
sample_32	sample	14654	14654	14654	13623	13130	12768	10922	10922	25,467
sample_33	sample	16997	16997	16997	15847	15221	14847	12752	12752	24,975
sample_34	sample	13376	13376	13376	12456	11958	11555	9763	9763	27,011
sample_35	sample	10850	10850	10850	10088	9798	9431	8251	8251	23,954
sample_36	sample	16982	16982	16982	15755	15270	14726	12646	12646	25,533
sample_37	sample	15845	15845	15845	14776	14259	13828	12114	12114	23,547
sample_38	sample	11581	11581	11581	10808	10373	10040	8821	8821	23,832
sample_39	sample	12285	12285	12285	11451	11123	10869	9762	9762	20,537
sample_40	sample	16487	16487	16487	15361	14796	14298	12133	12133	26,409
sample_41	sample	13351	13351	13351	12412	11997	11479	9998	9998	25,114
sample_42	sample	17429	17429	17429	16239	15772	15163	13413	13413	23,042
sample_43	sample	14181	14181	14181	13220	12686	12275	10469	10469	26,176
sample_44	sample	10720	10720	10720	10030	9605	9261	7803	7803	27,211
sample_45	sample	15332	15332	15332	14235	13726	13262	11442	11442	25,372
sample_46	sample	15053	15053	15053	13990	13594	13155	11369	11369	24,474
sample_47	sample	15608	15608	15608	14522	14008	13543	11905	11905	23,725
sample_48	sample	14007	14007	14007	12944	12491	12116	10538	10538	24,766
sample_49	sample	15132	15132	15132	14039	13585	12999	11043	11043	27,022
sample_50	sample	12945	12945	12945	11902	11586	11141	10029	10029	22,526
sample_51	sample	11006	11006	11006	10235	9932	9577	8474	8474	23,006
sample_52	sample	11649	11649	11649	10850	10491	10228	9065	9065	22,182
sample_53	sample	16744	16744	16744	15607	14965	14442	12064	12064	27,95
sample_54	sample	10843	10843	10843	10010	9657	9328	8246	8246	23,951
sample_55	sample	13312	13312	13312	12358	11943	11438	9863	9863	25,909
sample_56	sample	12722	12722	12722	11818	11374	11093	9394	9394	26,159
sample_57	sample	14466	14466	14466	13464	12993	12535	10814	10814	25,245
sample_58	sample	15326	15326	15326	14268	13739	13256	11320	11320	26,139
sample_59	sample	10888	10888	10888	10153	9739	9315	7922	7922	27,241
sample_60	sample	13572	13572	13572	12626	12098	11729	10037	10037	26,046
sample_61	sample	13780	13780	13780	11883	11387	10856	9174	9174	33,425
sample_62	sample	9867	9867	9867	9191	8770	8468	7082	7082	28,225
sample_63	sample	14369	14369	14369	13425	12996	12605	10752	10752	25,172
sample_64	sample	12989	12989	12989	12055	11664	11256	9887	9887	23,882
sample_65	sample	14013	14013	14013	12993	12525	12031	10490	10490	25,141
sample_66	sample	10616	10616	10616	9825	9437	9132	7855	7855	26,008
sample_67	sample	17676	17676	17676	16502	16032	15301	13518	13518	23,523
sample_68	sample	14494	14494	14494	13499	13072	12529	11062	11062	23,679
sample_69	sample	15264	15264	15264	14248	13711	13124	11039	11039	27,68
sample_70	sample	11983	11983	11983	11143	10685	10348	8778	8778	26,746
sample_71	sample	24602	24602	24602	22928	22287	21627	19277	19277	21,645
sample_72	sample	13851	13851	13851	12927	12616	12143	10947	10947	20,966
sample_73	sample	16900	16900	16900	15738	15116	14669	12501	12501	26,03
sample_74	sample	13625	13625	13625	12713	12345	11914	10454	10454	23,273
sample_75	sample	13641	13641	13641	12722	12300	11935	10534	10534	22,777
sample_76	sample	18927	18927	18927	17643	17068	16361	14182	14182	25,07

sample_77	sample	12938	12938	12938	12085	11717	11337	9508	9508	26,511
sample_78	sample	14878	14878	14878	13833	13307	12970	10902	10902	26,724
sample_79	sample	15716	15716	15716	14661	14161	13755	11571	11571	26,374
sample_80	sample	16140	16140	16140	15041	14576	13922	12163	12163	24,641
sample_81	sample	24018	24018	24018	22361	21543	20810	17063	17063	28,957
sample_82	sample	18778	18778	18778	17470	16899	16342	14399	14399	23,32
sample_83	sample	14443	14443	14443	13420	12864	12415	10156	10156	29,682
sample_84	sample	19070	19070	19070	17819	17227	16584	14332	14332	24,845
sample_85	sample	7869	7869	7869	7370	7059	6924	6301	6301	19,926
sample_86	sample	12986	12986	12986	12083	11688	11294	9679	9679	25,466
sample_87	sample	21351	21351	21351	19806	19141	18368	16113	16113	24,533
sample_88	sample	13606	13606	13606	12629	12263	11827	10615	10615	21,983
sample_89	sample	11679	11679	11679	10843	10415	10061	8737	8737	25,191
sample_90	sample	8977	8977	8977	8350	8067	7816	7135	7135	20,519
sample_91	sample	13002	13002	13002	12124	11691	11420	9793	9793	24,681
sample_92	sample	25290	25290	25290	23560	22959	22259	20016	20016	20,854
sample_93	sample	15640	15640	15640	14560	13973	13467	11621	11621	25,697
sample_94	sample	11098	11098	11098	10327	9930	9621	8403	8403	24,284
sample_95	sample	17614	17614	17614	16408	15963	15466	13664	13664	22,425
sample_96	sample	12183	12183	12183	11322	11040	10594	9269	9269	23,919
sample_97	sample	6351	6351	6351	5882	5607	5356	4297	4297	32,341
sample_98	sample	13079	13079	13079	12168	11725	11241	9983	9983	23,672
sample_99	sample	19818	19818	19818	18468	17895	17228	14883	14883	24,902
sample_100	sample	18614	18614	18614	17344	16854	16347	14196	14196	23,735
sample_101	sample	12402	12402	12402	11538	11132	10762	9284	9284	25,141
sample_102	sample	17389	17389	17389	16227	15609	15235	12866	12866	26,011
sample_103	sample	4419	4419	4419	4123	3939	3833	3383	3383	23,444
sample_104	sample	21736	21736	21736	20247	19683	18903	16527	16527	23,965
sample_105	sample	17106	17106	17106	15961	15406	14907	12685	12685	25,845
sample_106	sample	14591	14591	14591	13671	13132	12644	10887	10887	25,386
sample_107	sample	15159	15159	15159	14112	13510	13059	10613	10613	29,989
sample_108	sample	14585	14585	14585	13603	12957	12580	10421	10421	28,55
sample_109	sample	13603	13603	13603	12666	12222	11863	10443	10443	23,23
sample_110	sample	10388	10388	10388	9552	9226	8926	7796	7796	24,952
sample_111	sample	10826	10826	10826	10097	9747	9473	8517	8517	21,328
sample_112	sample	11207	11207	11207	10410	10016	9686	8381	8381	25,216
sample_113	sample	13491	13491	13491	12565	12032	11668	9708	9708	28,041
sample_114	sample	18545	18545	18545	17272	16615	16179	13366	13366	27,927
sample_115	sample	16766	16766	16766	15556	14945	14531	12209	12209	27,18
sample_116	sample	10661	10661	10661	9909	9507	9225	8048	8048	24,51
sample_117	sample	13172	13172	13172	12266	11773	11415	9786	9786	25,706
sample_118	sample	17010	17010	17010	15857	15415	14923	13331	13331	21,628
sample_119	sample	19457	19457	19457	18120	17449	16742	14065	14065	27,712
sample_120	sample	17370	17370	17370	16196	15635	15023	13001	13001	25,153
sample_121	sample	22503	22503	22503	21039	20576	19983	18004	18004	19,993
sample_122	sample	10249	10249	10249	9503	9116	8794	7033	7033	31,379
sample_123	sample	13987	13987	13987	13050	12546	12222	10194	10194	27,118
sample_124	sample	8950	8950	8950	8346	8110	7838	7221	7221	19,318
sample_125	sample	21117	21117	21117	19697	19012	18311	15616	15616	26,05
sample_126	sample	14426	14426	14426	13469	13009	12678	11395	11395	21,011
sample_127	sample	16748	16748	16748	15597	15065	14501	12642	12642	24,516
sample_128	sample	12846	12846	12846	12006	11437	11097	9031	9031	29,698
sample_129	sample	22167	22167	22167	20622	19948	19270	16442	16442	25,827
sample_130	sample	17154	17154	17154	15951	15378	14862	12932	12932	24,612
sample_131	sample	5257	5257	5257	4926	4734	4606	4249	4249	19,174
sample_132	sample	13204	13204	13204	12271	11808	11383	9351	9351	29,181
sample_133	sample	4433	4433	4433	4142	3926	3788	3439	3439	22,423
sample_134	sample	13387	13387	13387	12499	11983	11670	10001	10001	25,293
sample_135	sample	20479	20479	20479	19110	18403	17800	14455	14455	29,415
sample_136	sample	14470	14470	14470	13530	13080	12615	11013	11013	23,891
sample_137	sample	18568	18568	18568	17309	16752	16117	13720	13720	26,109
sample_138	sample	11005	11005	11005	10236	9869	9550	8279	8279	24,771
sample_139	sample	17145	17145	17145	16008	15389	15023	12697	12697	25,943
sample_140	sample	8961	8961	8961	8386	8101	7765	6882	6882	23,201
sample_141	sample	9024	9024	9024	8392	8121	7679	6708	6708	25,665
sample_142	sample	12267	12267	12267	11380	10958	10528	9209	9209	24,929

sample_143	sample	13627	13627	13627	12691	12211	11718	9839	9839	27,798
sample_144	sample	1556	1556	1556	1455	1357	1306	1051	1051	32,455
sample_145	sample	13909	13909	13909	12959	12436	12019	10283	10283	26,069
sample_146	sample	11159	11159	11159	10438	10134	9699	8375	8375	24,948
sample_147	sample	16215	16215	16215	15108	14455	14022	11628	11628	28,289
sample_148	sample	15353	15353	15353	14339	13837	13478	11343	11343	26,119
sample_149	sample	10093	10093	10093	9407	9124	8760	7952	7952	21,213
sample_150	sample	10147	10147	10147	9443	9172	8835	7676	7676	24,352
sample_151	sample	16412	16412	16412	15334	14844	14448	12863	12863	21,624
sample_152	sample	15049	15049	15049	14061	13664	13103	11844	11844	21,297
sample_153	sample	12505	12505	12505	11666	11251	10908	9356	9356	25,182
sample_154	sample	8019	8019	8019	7451	7189	6895	6019	6019	24,941
sample_155	sample	16879	16879	16879	15713	15139	14586	12528	12528	25,778
sample_156	sample	16329	16329	16329	15168	14718	14179	12512	12512	23,376
sample_157	sample	17108	17108	17108	15997	15342	14980	13222	13222	22,715
sample_158	sample	12858	12858	12858	11905	11497	11054	9804	9804	23,752
sample_159	sample	13593	13593	13593	12691	12418	11975	10906	10906	19,768
sample_160	sample	13247	13247	13247	12361	11940	11578	10201	10201	22,994
sample_161	sample	12648	12648	12648	11756	11304	11014	9437	9437	25,387
sample_162	sample	18842	18842	18842	17573	16882	16421	13389	13389	28,941
sample_163	sample	10577	10577	10577	9843	9574	9259	8304	8304	21,49
sample_164	sample	12465	12465	12465	11660	11215	10908	9303	9303	25,367
sample_165	sample	14122	14122	14122	13146	12609	12243	10168	10168	27,999
sample_166	sample	5006	5006	5006	4648	4488	4335	4006	4006	19,976
sample_167	sample	11253	11253	11253	10505	10082	9753	8118	8118	27,859
sample_168	sample	13789	13789	13789	12042	11720	11291	10415	10415	24,469
sample_169	sample	9188	9188	9188	8583	8284	7962	7382	7382	19,656
sample_170	sample	10172	10172	10172	9450	9076	8854	7402	7402	27,232
sample_171	sample	9826	9826	9826	9147	8762	8387	7262	7262	26,094
sample_172	sample	16343	16343	16343	15121	14558	13966	11926	11926	27,027
sample_173	sample	13383	13383	13383	12523	12136	11574	10428	10428	22,08
sample_174	sample	16605	16605	16605	15471	14891	14462	12030	12030	27,552
sample_175	sample	15365	15365	15365	14305	13938	13327	11635	11635	24,276
sample_176	sample	20690	20690	20690	19269	18705	18014	15542	15542	24,882
sample_177	sample	17354	17354	17354	16199	15627	15187	12864	12864	25,873
sample_178	sample	15172	15172	15172	14091	13501	13037	10597	10597	30,154
sample_179	sample	13369	13369	13369	12458	12065	11577	9653	9653	27,796
sample_180	sample	7067	7067	7067	6593	6269	6047	5088	5088	28,003
sample_181	sample	13976	13976	13976	12924	12451	11969	10055	10055	28,055
sample_182	sample	21354	21354	21354	19865	19342	18522	16374	16374	23,321
sample_183	sample	13235	13235	13235	12341	11949	11434	9788	9788	26,045
sample_184	sample	13916	13916	13916	12964	12607	12100	10496	10496	24,576
sample_185	sample	22080	22080	22080	20561	19840	19135	16049	16049	27,314
sample_186	sample	17384	17384	17384	16143	15519	15171	12826	12826	26,22
sample_187	sample	17054	17054	17054	15921	15375	14929	12718	12718	25,425
sample_188	sample	15105	15105	15105	14133	13754	13316	11817	11817	21,768
sample_189	sample	10943	10943	10943	9648	9315	9005	8191	8191	25,148
sample_190	sample	10446	10446	10446	9695	9358	9140	8227	8227	21,243
sample_191	sample	18184	18184	18184	16939	16443	15825	13917	13917	23,466
sample_192	sample	12467	12467	12467	11549	11167	10879	9810	9810	21,312
sample_193	sample	15124	15124	15124	14111	13583	12991	10766	10766	28,815
sample_194	sample	12142	12142	12142	11258	10833	10523	9171	9171	24,469
sample_195	sample	20896	20896	20896	19420	18754	18107	15135	15135	27,57
sample_196	sample	11469	11469	11469	10616	10300	9928	9185	9185	19,915
sample_197	sample	12154	12154	12154	11288	10870	10491	9202	9202	24,288
sample_198	sample	11830	11830	11830	11001	10670	10298	9554	9554	19,239
sample_199	sample	14127	14127	14127	13078	12676	12270	10752	10752	23,89
sample_200	sample	12810	12810	12810	11980	11532	11104	9149	9149	28,579
sample_201	sample	10618	10618	10618	9852	9441	9326	8128	8128	23,451
sample_202	sample	16181	16181	16181	15139	14657	14147	12212	12212	24,529
sample_203	sample	14992	14992	14992	14006	13610	13148	11612	11612	22,545
sample_204	sample	4536	4536	4536	4228	4069	3912	3575	3575	21,186
sample_205	sample	163	163	163	150	73	81	48	48	70,552
sample_206	sample	15068	15068	15068	14017	13556	13158	11393	11393	24,389
sample_207	sample	9261	9261	9261	8625	8272	8065	6866	6866	25,861
sample_208	sample	17512	17512	17512	16341	15777	15173	12988	12988	25,834

sample_209	sample	10762	10762	10762	9947	9513	9180	7758	7758	27,913
sample_210	sample	10978	10978	10978	10188	9855	9541	8491	8491	22,654
sample_211	sample	19567	19567	19567	18232	17671	17028	14835	14835	24,184
sample_212	sample	17289	17289	17289	16151	15722	15202	13514	13514	21,835
sample_213	sample	14687	14687	14687	13633	13128	12649	10992	10992	25,158
sample_214	sample	18087	18087	18087	16834	16246	15388	13410	13410	25,858
sample_215	sample	12363	12363	12363	11517	11137	10785	9525	9525	22,956
sample_216	sample	12843	12843	12843	11980	11474	11109	9168	9168	28,615
sample_217	sample	19474	19474	19474	18150	17754	16877	15135	15135	22,281
sample_218	sample	12887	12887	12887	11974	11457	11153	9322	9322	27,664
sample_219	sample	10958	10958	10958	10167	9938	9607	9043	9043	17,476
sample_220	sample	9334	9334	9334	8633	8365	8065	7296	7296	21,834
sample_221	sample	14934	14934	14934	13934	13595	13122	11746	11746	21,347
sample_222	sample	14020	14020	14020	13055	12571	12099	10165	10165	27,496
sample_223	sample	12838	12838	12838	11938	11631	11309	9954	9954	22,465
sample_224	sample	20114	20114	20114	18760	17930	17262	13514	13514	32,813
sample_225	sample	17597	17597	17597	16385	15781	15268	12815	12815	27,175
sample_226	sample	13547	13547	13547	12670	12250	11955	10715	10715	20,905
sample_227	sample	13551	13551	13551	12610	12116	11736	9775	9775	27,865
sample_228	sample	13119	13119	13119	12214	11759	11315	9512	9512	27,494
sample_229	sample	13421	13421	13421	12463	11960	11530	9465	9465	29,476
sample_230	sample	17159	17159	17159	15945	15509	14861	13286	13286	22,571
sample_231	sample	16547	16547	16547	15409	14877	14348	12403	12403	25,044
sample_232	sample	19953	19953	19953	18569	17979	17302	14571	14571	26,973
sample_233	sample	7738	7738	7738	7148	6828	6628	5947	5947	23,146
sample_234	sample	11570	11570	11570	10739	10489	10136	9206	9206	20,432
sample_235	sample	17383	17383	17383	16144	15557	14993	12964	12964	25,421
sample_236	sample	18989	18989	18989	17712	17041	16377	13535	13535	28,722
sample_237	sample	13696	13696	13696	12777	12341	11922	10158	10158	25,832
sample_238	sample	12363	12363	12363	11507	11083	10738	9234	9234	25,309
sample_239	sample	94	94	94	82	29	24	13	13	86,17
sample_240	sample	6662	6662	6662	6190	5942	5725	5272	5272	20,865
sample_241	sample	15947	15947	15947	14823	14246	13633	11610	11610	27,196
sample_242	sample	15651	15651	15651	14577	13989	13540	11450	11450	26,842
sample_243	sample	14217	14217	14217	13258	12775	12427	10492	10492	26,201
sample_244	sample	18323	18323	18323	17055	16410	16022	13396	13396	26,89
sample_245	sample	12628	12628	12628	11686	11193	10862	9056	9056	28,286
sample_246	sample	13896	13896	13896	12920	12405	11993	10160	10160	26,885
sample_247	sample	15305	15305	15305	14269	13725	13300	11241	11241	26,553
sample_248	sample	14754	14754	14754	13748	13227	12790	11192	11192	24,143
sample_249	sample	16037	16037	16037	14960	14414	13793	11633	11633	27,461
sample_250	sample	19975	19975	19975	18657	17883	17186	13776	13776	31,034
sample_251	sample	14461	14461	14461	13452	13055	12531	10842	10842	25,026
sample_252	sample	21224	21224	21224	19693	19196	18530	16569	16569	21,933
sample_253	sample	17062	17062	17062	15829	15281	14698	12784	12784	25,073
sample_254	sample	226	226	226	204	133	110	72	72	68,142
sample_255	sample	17540	17540	17540	16390	15902	15329	13769	13769	21,499
sample_256	sample	12958	12958	12958	12134	11703	11313	9724	9724	24,958
sample_257	sample	12747	12747	12747	11896	11462	11052	9432	9432	26,006
sample_258	sample	17514	17514	17514	16306	15746	15227	12936	12936	26,139
sample_259	sample	8650	8650	8650	8072	7711	7502	6397	6397	26,046
sample_260	sample	16205	16205	16205	15224	14694	14193	12311	12311	24,03
sample_261	sample	11784	11784	11784	10941	10629	10266	9279	9279	21,258
sample_262	sample	14696	14696	14696	13655	13163	12806	10596	10596	27,899
sample_263	sample	17723	17723	17723	16528	15958	15443	13653	13653	22,965
sample_264	sample	7663	7663	7663	7158	6886	6624	5700	5700	25,617
sample_265	sample	9812	9812	9812	9163	8861	8642	7816	7816	20,342
sample_266	sample	15389	15389	15389	14369	13823	13470	11772	11772	23,504
sample_267	sample	15739	15739	15739	14679	14298	13805	12296	12296	21,876
sample_268	sample	15993	15993	15993	14878	14366	13943	12189	12189	23,785
sample_269	sample	10717	10717	10717	10003	9635	9237	8060	8060	24,792
sample_270	sample	17010	17010	17010	15782	15262	14790	11954	11954	29,724
sample_271	sample	20085	20085	20085	18743	18271	17541	15374	15374	23,455
sample_272	sample	16331	16331	16331	15217	14868	14315	13037	13037	20,17
sample_273	sample	13313	13313	13313	12420	11950	11584	9819	9819	26,245
sample_274	sample	16630	16630	16630	15473	14845	14393	12117	12117	27,138

sample_275	sample	8746	8746	8746	8192	7821	7554	6403	6403	26,789
sample_276	sample	14406	14406	14406	13425	12929	12584	11147	11147	22,623
sample_277	sample	18811	18811	18811	17524	16789	16190	13820	13820	26,532
sample_278	sample	9745	9745	9745	9066	8676	8433	7276	7276	25,336
sample_279	sample	14783	14783	14783	13798	13315	12921	11151	11151	24,569
sample_280	sample	12602	12602	12602	11737	11264	10860	9229	9229	26,766
sample_281	sample	18571	18571	18571	17253	16652	16050	13870	13870	25,314
sample_282	sample	14970	14970	14970	13931	13462	12906	11371	11371	24,041
sample_283	sample	13298	13298	13298	12330	11865	11476	10235	10235	23,034
sample_284	sample	13029	13029	13029	12083	11702	11271	10034	10034	22,987
sample_285	sample	31979	31979	31979	29858	29152	28232	25170	25170	21,292
sample_286	sample	11203	11203	11203	10432	10114	9827	8905	8905	20,512
sample_287	sample	15273	15273	15273	14218	13567	13203	11258	11258	26,288
sample_288	sample	12628	12628	12628	11781	11293	10829	8842	8842	29,981
sample_289	sample	19659	19659	19659	18304	17641	17102	14951	14951	23,948
sample_290	sample	15543	15543	15543	14434	13959	13473	11456	11456	26,295
sample_291	sample	15181	15181	15181	14103	13614	13090	11052	11052	27,198
sample_292	sample	13529	13529	13529	12624	12105	11822	10229	10229	24,392
sample_293	sample	13607	13607	13607	12729	12290	11874	10625	10625	21,915
sample_294	sample	11758	11758	11758	10935	10469	10061	8496	8496	27,743
sample_295	sample	13194	13194	13194	12257	11773	11446	9672	9672	26,694
sample_296	sample	12568	12568	12568	11765	11502	11115	10216	10216	18,714
sample_297	sample	14450	14450	14450	13536	13039	12583	10503	10503	27,315
sample_298	sample	14666	14666	14666	13672	13126	12698	10954	10954	25,31
sample_299	sample	14893	14893	14893	13887	13363	12956	10885	10885	26,912
sample_300	sample	16625	16625	16625	15507	14967	14395	12481	12481	24,926
sample_301	sample	18526	18526	18526	17047	16459	15746	13739	13739	25,839
sample_302	sample	5778	5778	5778	5382	5219	5094	4735	4735	18,051
sample_303	sample	11034	11034	11034	10266	9879	9609	8011	8011	27,397
sample_304	sample	16678	16678	16678	15555	15174	14733	13098	13098	21,465
sample_305	sample	15999	15999	15999	14904	14394	13964	11947	11947	25,327
sample_306	sample	14214	14214	14214	13211	12866	12447	11042	11042	22,316
sample_307	sample	15280	15280	15280	14234	13840	13322	11513	11513	24,653
sample_308	sample	16698	16698	16698	15572	15192	14501	13087	13087	21,625
sample_309	sample	140	140	140	132	73	71	41	41	70,714
sample_310	sample	13666	13666	13666	12729	12305	11877	10621	10621	22,282
sample_311	sample	19286	19286	19286	18021	17471	16722	14546	14546	24,577
sample_312	sample	91	91	91	81	22	7	2	2	97,802
sample_313	sample	12913	12913	12913	12002	11633	11281	9748	9748	24,51
sample_314	sample	11759	11759	11759	10953	10411	10036	8155	8155	30,649
sample_315	sample	17529	17529	17529	16278	15754	15220	13250	13250	24,411
sample_316	sample	15088	15088	15088	14084	13589	13041	11342	11342	24,828
sample_317	sample	16671	16671	16671	15518	14983	14470	12498	12498	25,031
sample_318	sample	15368	15368	15368	14312	13925	13516	11861	11861	22,82
sample_319	sample	9571	9571	9571	8873	8515	8074	7135	7135	25,452
sample_320	sample	12095	12095	12095	11282	10868	10505	9157	9157	24,291
sample_321	sample	11535	11535	11535	10728	10244	9965	8620	8620	25,271
sample_322	sample	11016	11016	11016	10224	9881	9590	8500	8500	22,84
sample_323	sample	17355	17355	17355	16132	15522	14931	12434	12434	28,355
sample_324	sample	557	557	557	516	457	433	316	316	43,268
sample_325	sample	17579	17579	17579	16416	15854	15329	13167	13167	25,098
sample_326	sample	10030	10030	10030	9361	9034	8681	7446	7446	25,763
sample_327	sample	11549	11549	11549	10783	10436	10115	8692	8692	24,738
sample_328	sample	13048	13048	13048	12117	11659	11328	9529	9529	26,97
sample_329	sample	15287	15287	15287	14199	13682	13227	11218	11218	26,617
sample_330	sample	12541	12541	12541	11680	11285	10934	9208	9208	26,577
sample_331	sample	20906	20906	20906	19527	18995	18408	15574	15574	25,505
sample_332	sample	12758	12758	12758	11672	11201	10811	9438	9438	26,023
sample_333	sample	15281	15281	15281	14219	13804	13413	11515	11515	24,645
sample_334	sample	9440	9440	9440	8737	8434	8181	7254	7254	23,157
sample_335	sample	10932	10932	10932	10195	9795	9439	7911	7911	27,634
sample_336	sample	13516	13516	13516	12615	12136	11707	10021	10021	25,858
sample_337	sample	16450	16450	16450	15397	14809	14281	11937	11937	27,435
sample_338	sample	17403	17403	17403	16243	15721	15134	13131	13131	24,547
sample_339	sample	9108	9108	9108	8503	8224	8007	7037	7037	22,738
sample_340	sample	11131	11131	11131	10379	10061	9619	8236	8236	26,008

sample_341	sample	11655	11655	11655	10876	10460	10113	8336	8336	28,477
sample_342	sample	15481	15481	15481	14445	13936	13476	11621	11621	24,934
sample_343	sample	13102	13102	13102	12226	11763	11429	9536	9536	27,217
sample_344	sample	15380	15380	15380	14286	13758	13307	11382	11382	25,995
sample_345	sample	10971	10971	10971	10224	9741	9440	7880	7880	28,174
sample_346	sample	18748	18748	18748	17489	16939	16208	14134	14134	24,611
sample_347	sample	10705	10705	10705	9955	9590	9255	8019	8019	25,091
sample_348	sample	16381	16381	16381	15291	14847	14328	12857	12857	21,513
sample_349	sample	13688	13688	13688	12800	12310	11912	10393	10393	24,072
sample_350	sample	13225	13225	13225	12352	11892	11470	9726	9726	26,457
sample_351	sample	18621	18621	18621	17353	16752	16213	14000	14000	24,816
sample_352	sample	11267	11267	11267	10485	10065	9788	8104	8104	28,073
sample_353	sample	16579	16579	16579	15525	15008	14532	12632	12632	23,807
sample_354	sample	13005	13005	13005	12092	11533	11212	9468	9468	27,197
sample_355	sample	12574	12574	12574	11717	11128	10825	8419	8419	33,044
sample_356	sample	18767	18767	18767	17442	16824	16243	13532	13532	27,895
sample_357	sample	9072	9072	9072	8431	8151	7840	6758	6758	25,507
sample_358	sample	9149	9149	9149	8538	8261	7969	6975	6975	23,762
sample_359	sample	11170	11170	11170	10431	10049	9747	8355	8355	25,201
sample_360	sample	17669	17669	17669	16436	15882	15302	13230	13230	25,123
sample_361	sample	11696	11696	11696	10928	10499	10052	8490	8490	27,411
sample_362	sample	15970	15970	15970	14814	14339	13734	11975	11975	25,016
sample_363	sample	20295	20295	20295	18638	18104	17453	15336	15336	24,435
sample_364	sample	8719	8719	8719	8152	7879	7623	7080	7080	18,798

Sequencing run 2

sampleID	type	rawData	output-Cutadapt	input-DADA2	filtered	denoisedF	denoisedR	merged	seqtab	percLost
blank_control_1	PCR_control	12	12	12	8	1	1	0	0	100
blank_control_2	PCR_control	6	6	6	6	1	1	0	0	100
blank_control_3	PCR_control	13	13	13	7	1	1	0	0	100
blank_control_4	PCR_control	19	19	19	17	2	1	0	0	100
blank_control_5	PCR_control	16	16	16	16	2	1	0	0	100
blank_control_6	PCR_control	7	7	7	7	2	1	0	0	100
blank_control_7	PCR_control	1	1	1	1	1	1	0	0	100
blank_control_8	PCR_control	1	1	1	1	1	1	0	0	100
blank_control_9	PCR_control	9	9	9	7	1	1	0	0	100
blank_control_10	PCR_control	24	24	24	13	3	1	0	0	100
blank_control_11	PCR_control	10	10	10	9	1	1	0	0	100
blank_control_12	PCR_control	2	2	2	2	1	1	0	0	100
blank_control_13	PCR_control	7	7	7	6	1	1	0	0	100
blank_control_14	PCR_control	5	5	5	4	1	1	0	0	100
blank_control_15	PCR_control	6	6	6	5	1	1	0	0	100
blank_control_16	PCR_control	7	7	7	5	1	1	0	0	100
blank_control_17	PCR_control	10	10	10	9	1	1	0	0	100
blank_control_18	DNA_extraction_control	87	87	87	84	17	1	0	0	100
blank_control_19	DNA_extraction_control	82	82	82	74	21	1	0	0	100
blank_control_20	DNA_extraction_control	75	75	75	72	2	1	0	0	100
blank_control_21	DNA_extraction_control	17	17	17	15	1	1	0	0	100
blank_control_22	DNA_extraction_control	21	21	21	18	1	1	0	0	100
blank_control_23	DNA_extraction_control	56	56	56	53	12	1	0	0	100
blank_control_24	DNA_extraction_control	61	61	61	56	2	1	0	0	100
blank_control_25	DNA_extraction_control	81	81	81	77	10	1	0	0	100
blank_control_26	DNA_extraction_control	67	67	67	60	16	1	0	0	100
blank_control_27	DNA_extraction_control	67	67	67	63	1	1	0	0	100
blank_control_28	DNA_extraction_control	79	79	79	69	16	1	0	0	100
blank_control_29	DNA_extraction_control	129	129	129	125	60	13	0	0	100
blank_control_30	DNA_extraction_control	71	71	71	68	12	1	0	0	100
blank_control_31	DNA_extraction_control	49	49	49	47	1	1	0	0	100
blank_control_32	DNA_extraction_control	62	62	62	60	2	1	0	0	100
blank_control_33	DNA_extraction_control	66	66	66	61	2	1	0	0	100
blank_control_34	DNA_extraction_control	75	75	75	71	15	1	0	0	100
blank_control_35	DNA_extraction_control	91	91	91	88	3	1	0	0	100
blank_control_36	DNA_extraction_control	102	102	102	98	35	1	0	0	100
blank_control_37	DNA_extraction_control	102	102	102	100	25	1	0	0	100

blank_control_38	DNA_extraction_control	58	58	58	56	16	1	0	0	100
blank_control_39	DNA_extraction_control	17	17	17	17	2	1	0	0	100
blank_control_40	DNA_extraction_control	13	13	13	12	1	1	0	0	100
blank_control_41	DNA_extraction_control	99	99	99	96	47	39	17	17	82,828
blank_control_42	DNA_extraction_control	128	128	128	125	50	52	24	24	81,25
blank_control_43	DNA_extraction_control	58	58	58	53	5	1	0	0	100
blank_control_44	DNA_extraction_control	51	51	51	46	16	1	0	0	100
blank_control_45	DNA_extraction_control	90	90	90	82	31	16	7	7	92,222
blank_control_46	DNA_extraction_control	62	62	62	55	4	1	0	0	100
blank_control_47	DNA_extraction_control	35	35	35	33	2	1	0	0	100
blank_control_48	DNA_extraction_control	135	135	135	126	32	44	5	5	96,296
blank_control_49	DNA_extraction_control	38	38	38	37	6	1	0	0	100
blank_control_50	DNA_extraction_control	64	64	64	64	19	1	0	0	100
sample_1	sample	14033	14033	14033	13952	13479	13164	11807	11807	15,863
sample_2	sample	15681	15681	15681	15599	15143	14635	13034	13034	16,88
sample_3	sample	12835	12835	12835	12775	12247	11789	9905	9905	22,828
sample_4	sample	13160	13160	13160	13090	12640	12203	10605	10605	19,415
sample_5	sample	13758	13758	13758	13694	13217	12876	11017	11017	19,923
sample_6	sample	18324	18324	18324	18212	17763	16969	14705	14705	19,75
sample_7	sample	17391	17391	17391	17295	16859	16197	14134	14134	18,728
sample_8	sample	10583	10583	10583	10541	10204	9742	8229	8229	22,243
sample_9	sample	15013	15013	15013	14924	14535	13944	12418	12418	17,285
sample_10	sample	20925	20925	20925	20839	20218	19481	17257	17257	17,529
sample_11	sample	15845	15845	15845	15736	15321	14789	13188	13188	16,769
sample_12	sample	11688	11688	11688	11625	11256	10896	9511	9511	18,626
sample_13	sample	14105	14105	14105	14038	13550	13042	11165	11165	20,844
sample_14	sample	11858	11858	11858	11783	11501	11214	10258	10258	13,493
sample_15	sample	19780	19780	19780	19478	18818	18174	15771	15771	20,268
sample_16	sample	15441	15441	15441	15385	14860	14389	12407	12407	19,649
sample_17	sample	8245	8245	8245	8209	7981	7700	6956	6956	15,634
sample_18	sample	15928	15928	15928	15836	15315	14755	12785	12785	19,733
sample_19	sample	10283	10283	10283	10220	9802	9399	7938	7938	22,805
sample_20	sample	13934	13934	13934	13853	13415	12940	10789	10789	22,571
sample_21	sample	13566	13566	13566	13497	13102	12566	11313	11313	16,608
sample_22	sample	20700	20700	20700	20571	19876	19255	15966	15966	22,87
sample_23	sample	17259	17259	17259	17182	16664	16099	14100	14100	18,303
sample_24	sample	11614	11614	11614	11492	11126	10726	9532	9532	17,927
sample_25	sample	16399	16399	16399	16311	15826	15185	13165	13165	19,721
sample_26	sample	16485	16485	16485	16403	15854	15176	12714	12714	22,875
sample_27	sample	13526	13526	13526	13452	12979	12567	10761	10761	20,442
sample_28	sample	16946	16946	16946	16870	16412	15726	13880	13880	18,093
sample_29	sample	10528	10528	10528	10431	10101	9709	8650	8650	17,838
sample_30	sample	18154	18154	18154	18070	17664	16917	15136	15136	16,624
sample_31	sample	11417	11417	11417	11293	10943	10603	9518	9518	16,633
sample_32	sample	15152	15152	15152	15065	14467	14076	11943	11943	21,179
sample_33	sample	17886	17886	17886	17813	17169	16694	14288	14288	20,116
sample_34	sample	13816	13816	13816	13725	13334	12779	10762	10762	22,105
sample_35	sample	11096	11096	11096	11019	10722	10353	9161	9161	17,439
sample_36	sample	17775	17775	17775	17695	17164	16506	14155	14155	20,366
sample_37	sample	16172	16172	16172	16098	15558	15100	13141	13141	18,742
sample_38	sample	12092	12092	12092	12016	11597	11110	9781	9781	19,112
sample_39	sample	12600	12600	12600	12549	12301	11917	10807	10807	14,23
sample_40	sample	17196	17196	17196	17124	16524	15952	13498	13498	21,505
sample_41	sample	13610	13610	13610	13521	13055	12544	11034	11034	18,927
sample_42	sample	18060	18060	18060	17984	17537	16722	14937	14937	17,292
sample_43	sample	14552	14552	14552	14456	13983	13546	11544	11544	20,671
sample_44	sample	11321	11321	11321	11258	10858	10312	8643	8643	23,655
sample_45	sample	15851	15851	15851	15777	15227	14575	12522	12522	21,002
sample_46	sample	15542	15542	15542	15470	15039	14500	12468	12468	19,779
sample_47	sample	16189	16189	16189	16102	15668	15068	13319	13319	17,728
sample_48	sample	14282	14282	14282	14197	13741	13235	11512	11512	19,395
sample_49	sample	15871	15871	15871	15709	15229	14652	12614	12614	20,522
sample_50	sample	13157	13157	13157	12890	12623	12112	11109	11109	15,566
sample_51	sample	11414	11414	11414	11351	11000	10544	9459	9459	17,128
sample_52	sample	12070	12070	12070	11975	11656	11281	10062	10062	16,636
sample_53	sample	17289	17289	17289	17209	16598	15958	13424	13424	22,355

sample_54	sample	11244	11244	11244	11062	10698	10327	9268	9268	17,574
sample_55	sample	13875	13875	13875	13819	13396	12891	11195	11195	19,315
sample_56	sample	13138	13138	13138	13052	12688	12170	10488	10488	20,17
sample_57	sample	14837	14837	14837	14758	14238	13761	11949	11949	19,465
sample_58	sample	15791	15791	15791	15716	15173	14671	12580	12580	20,334
sample_59	sample	11474	11474	11474	11409	10979	10414	8861	8861	22,773
sample_60	sample	14238	14238	14238	14164	13588	13119	11325	11325	20,459
sample_61	sample	14655	14655	14655	13399	12971	12276	10549	10549	28,018
sample_62	sample	10273	10273	10273	10224	9755	9432	7957	7957	22,545
sample_63	sample	14841	14841	14841	14770	14307	13841	11743	11743	20,875
sample_64	sample	13200	13200	13200	13131	12657	12212	10712	10712	18,848
sample_65	sample	14503	14503	14503	14408	13975	13447	11753	11753	18,962
sample_66	sample	10741	10741	10741	10674	10337	9955	8680	8680	19,188
sample_67	sample	18612	18612	18612	18510	17959	17206	15088	15088	18,934
sample_68	sample	14739	14739	14739	14658	14248	13687	12147	12147	17,586
sample_69	sample	16285	16285	16285	16191	15556	14949	12645	12645	22,352
sample_70	sample	12475	12475	12475	12407	11922	11377	9761	9761	21,756
sample_71	sample	25585	25585	25585	25475	24858	24158	21465	21465	16,103
sample_72	sample	14239	14239	14239	14173	13812	13363	12100	12100	15,022
sample_73	sample	17334	17334	17334	17237	16645	16080	13818	13818	20,284
sample_74	sample	14176	14176	14176	14115	13727	13271	11800	11800	16,761
sample_75	sample	14521	14521	14521	14426	13986	13478	11931	11931	17,836
sample_76	sample	19914	19914	19914	19835	19252	18333	16188	16188	18,71
sample_77	sample	13284	13284	13284	13215	12854	12391	10553	10553	20,559
sample_78	sample	15318	15318	15318	15242	14703	14286	12046	12046	21,36
sample_79	sample	15758	15758	15758	15683	15131	14675	12523	12523	20,529
sample_80	sample	16870	16870	16870	16788	16341	15599	13865	13865	17,813
sample_81	sample	24514	24514	24514	24385	23657	22760	18702	18702	23,709
sample_82	sample	19092	19092	19092	18989	18428	17820	15778	15778	17,358
sample_83	sample	14909	14909	14909	14822	14229	13769	11230	11230	24,676
sample_84	sample	19532	19532	19532	19419	18808	18098	15785	15785	19,184
sample_85	sample	8220	8220	8220	8191	7950	7640	7123	7123	13,345
sample_86	sample	13419	13419	13419	13341	12975	12414	10568	10568	21,246
sample_87	sample	21721	21721	21721	21491	20797	20005	17388	17388	19,948
sample_88	sample	14002	14002	14002	13902	13650	13015	11847	11847	15,391
sample_89	sample	11772	11772	11772	11701	11313	10832	9456	9456	19,674
sample_90	sample	9150	9150	9150	9110	8807	8505	7686	7686	16
sample_91	sample	13215	13215	13215	13158	12869	12335	10830	10830	18,048
sample_92	sample	25996	25996	25996	25870	25271	24467	21980	21980	15,449
sample_93	sample	16634	16634	16634	16553	16006	15369	13244	13244	20,38
sample_94	sample	11208	11208	11208	11153	10750	10409	9158	9158	18,291
sample_95	sample	18206	18206	18206	18104	17676	17018	15148	15148	16,797
sample_96	sample	12619	12619	12619	12548	12262	11722	10214	10214	19,059
sample_97	sample	6737	6737	6737	6698	6370	6162	4906	4906	27,178
sample_98	sample	13721	13721	13721	13659	13267	12715	11413	11413	16,821
sample_99	sample	20407	20407	20407	20306	19808	18961	16349	16349	19,885
sample_100	sample	19258	19258	19258	19144	18543	17986	15617	15617	18,906
sample_101	sample	12862	12862	12862	12792	12381	11990	10309	10309	19,849
sample_102	sample	18081	18081	18081	18001	17313	16958	14300	14300	20,911
sample_103	sample	32493	32493	32493	32354	31742	30738	28816	28816	11,316
sample_104	sample	22244	22244	22244	22134	21514	20618	18163	18163	18,347
sample_105	sample	17788	17788	17788	17688	17091	16398	14082	14082	20,834
sample_106	sample	15396	15396	15396	15320	14804	14212	12264	12264	20,343
sample_107	sample	16068	16068	16068	15972	15238	14792	11928	11928	25,765
sample_108	sample	14768	14768	14768	14699	14090	13614	11308	11308	23,429
sample_109	sample	14032	14032	14032	13944	13579	13077	11487	11487	18,137
sample_110	sample	11000	11000	11000	10841	10480	10169	8843	8843	19,609
sample_111	sample	11184	11184	11184	11131	10808	10419	9436	9436	15,629
sample_112	sample	11755	11755	11755	11650	11244	10904	9444	9444	19,66
sample_113	sample	13782	13782	13782	13691	13131	12652	10645	10645	22,762
sample_114	sample	18839	18839	18839	18734	18083	17516	14760	14760	21,652
sample_115	sample	17461	17461	17461	17375	16834	16312	13813	13813	20,892
sample_116	sample	11184	11184	11184	11110	10721	10345	9008	9008	19,456
sample_117	sample	13754	13754	13754	13671	13183	12791	11189	11189	18,649
sample_118	sample	17672	17672	17672	17563	17051	16507	14721	14721	16,699
sample_119	sample	20197	20197	20197	20094	19355	18567	15524	15524	23,137

sample_120	sample	17930	17930	17930	17836	17358	16624	14488	14488	19,197
sample_121	sample	22882	22882	22882	22758	22305	21658	19528	19528	14,658
sample_122	sample	10731	10731	10731	10618	10246	9762	8051	8051	24,974
sample_123	sample	14851	14851	14851	14781	14331	13888	11722	11722	21,069
sample_124	sample	9221	9221	9221	9172	8925	8589	7845	7845	14,922
sample_125	sample	21547	21547	21547	21441	20762	20045	17022	17022	21,001
sample_126	sample	15035	15035	15035	14971	14505	14100	12681	12681	15,657
sample_127	sample	17433	17433	17433	17359	16833	16084	14160	14160	18,775
sample_128	sample	13202	13202	13202	13141	12616	12178	9988	9988	24,345
sample_129	sample	23449	23449	23449	23328	22703	21852	18916	18916	19,331
sample_130	sample	17531	17531	17531	17443	16886	16403	14305	14305	18,402
sample_131	sample	28210	28210	28210	28064	27668	26577	25668	25668	9,011
sample_132	sample	13639	13639	13639	13562	13004	12515	10409	10409	23,682
sample_133	sample	31801	31801	31801	31651	31047	29734	28588	28588	10,103
sample_134	sample	13749	13749	13749	13672	13193	12762	11170	11170	18,758
sample_135	sample	21351	21351	21351	21247	20533	19772	16439	16439	23,006
sample_136	sample	14780	14780	14780	14679	14287	13638	11835	11835	19,926
sample_137	sample	19095	19095	19095	19013	18511	17782	15066	15066	21,1
sample_138	sample	11422	11422	11422	11335	10973	10506	9081	9081	20,496
sample_139	sample	17757	17757	17757	17639	17069	16487	14103	14103	20,578
sample_140	sample	9303	9303	9303	9253	8976	8628	7751	7751	16,683
sample_141	sample	9504	9504	9504	9449	9157	8737	7555	7555	20,507
sample_142	sample	12805	12805	12805	12727	12360	11795	10354	10354	19,141
sample_143	sample	14346	14346	14346	14265	13770	13157	10990	10990	23,393
sample_144	sample	15245	15245	15245	15181	14860	14465	13672	13672	10,318
sample_145	sample	14378	14378	14378	14291	13742	13201	11247	11247	21,776
sample_146	sample	11402	11402	11402	11356	10969	10600	9112	9112	20,084
sample_147	sample	16683	16683	16683	16596	15967	15366	12812	12812	23,203
sample_148	sample	16106	16106	16106	16028	15448	15048	12603	12603	21,75
sample_149	sample	10494	10494	10494	10430	10097	9751	8761	8761	16,514
sample_150	sample	10480	10480	10480	10403	10091	9784	8523	8523	18,674
sample_151	sample	16935	16935	16935	16867	16406	15855	14147	14147	16,463
sample_152	sample	15201	15201	15201	15123	14731	14214	12887	12887	15,223
sample_153	sample	12954	12954	12954	12889	12390	12069	10280	10280	20,642
sample_154	sample	8579	8579	8579	8532	8245	7922	6896	6896	19,618
sample_155	sample	17451	17451	17451	17371	16764	16121	13771	13771	21,088
sample_156	sample	17231	17231	17231	17121	16652	15973	14130	14130	17,997
sample_157	sample	17645	17645	17645	17556	16967	16456	14631	14631	17,081
sample_158	sample	13423	13423	13423	13255	12772	12302	10774	10774	19,735
sample_159	sample	13983	13983	13983	13894	13589	13057	11899	11899	14,904
sample_160	sample	13938	13938	13938	13863	13525	13005	11504	11504	17,463
sample_161	sample	13225	13225	13225	13151	12661	12339	10721	10721	18,934
sample_162	sample	19638	19638	19638	19532	18862	18266	15132	15132	22,945
sample_163	sample	10875	10875	10875	10822	10577	10162	9155	9155	15,816
sample_164	sample	12897	12897	12897	12827	12377	11940	10325	10325	19,943
sample_165	sample	14579	14579	14579	14523	14004	13596	11277	11277	22,649
sample_166	sample	21568	21568	21568	21474	21075	20409	19617	19617	9,0458
sample_167	sample	11711	11711	11711	11655	11247	10916	9035	9035	22,85
sample_168	sample	14296	14296	14296	13275	12989	12404	11525	11525	19,383
sample_169	sample	9551	9551	9551	9505	9219	8894	8334	8334	12,742
sample_170	sample	10481	10481	10481	10424	10025	9728	8190	8190	21,859
sample_171	sample	9923	9923	9923	9851	9466	9139	7856	7856	20,83
sample_172	sample	16902	16902	16902	16814	16180	15485	13301	13301	21,305
sample_173	sample	13607	13607	13607	13527	13114	12560	11262	11262	17,234
sample_174	sample	16978	16978	16978	16906	16269	15781	13216	13216	22,158
sample_175	sample	15910	15910	15910	15769	15393	14630	12960	12960	18,542
sample_176	sample	21610	21610	21610	21487	20916	20044	17510	17510	18,973
sample_177	sample	17853	17853	17853	17746	17196	16683	14141	14141	20,792
sample_178	sample	15635	15635	15635	15561	14903	14455	11761	11761	24,778
sample_179	sample	14152	14152	14152	14063	13498	13036	10976	10976	22,442
sample_180	sample	7578	7578	7578	7521	7265	6914	5873	5873	22,499
sample_181	sample	14552	14552	14552	14472	13930	13487	11394	11394	21,701
sample_182	sample	21966	21966	21966	21849	21296	20439	17954	17954	18,265
sample_183	sample	13800	13800	13800	13726	13324	12824	11030	11030	20,072
sample_184	sample	14541	14541	14541	14470	14030	13649	11738	11738	19,277
sample_185	sample	22934	22934	22934	22819	22092	21305	17840	17840	22,212

sample_186	sample	17941	17941	17941	17846	17298	16802	14102	14102	21,398
sample_187	sample	17353	17353	17353	17263	16726	16164	13824	13824	20,337
sample_188	sample	15499	15499	15499	15419	14993	14561	12994	12994	16,162
sample_189	sample	11122	11122	11122	10413	10119	9710	8936	8936	19,655
sample_190	sample	10934	10934	10934	10838	10511	10191	9180	9180	16,042
sample_191	sample	18928	18928	18928	18823	18310	17533	15526	15526	17,973
sample_192	sample	13009	13009	13009	12941	12599	12196	10941	10941	15,897
sample_193	sample	15613	15613	15613	15533	14866	14290	11692	11692	25,114
sample_194	sample	12592	12592	12592	12531	12090	11674	10223	10223	18,814
sample_195	sample	21613	21613	21613	21454	20614	20064	16860	16860	21,991
sample_196	sample	11729	11729	11729	11592	11308	10829	10077	10077	14,085
sample_197	sample	12629	12629	12629	12566	12176	11646	10267	10267	18,703
sample_198	sample	12033	12033	12033	11973	11639	11229	10459	10459	13,081
sample_199	sample	14589	14589	14589	14469	14010	13446	11590	11590	20,557
sample_200	sample	12979	12979	12979	12912	12542	12011	9996	9996	22,983
sample_201	sample	11226	11226	11226	11140	10775	10453	9181	9181	18,217
sample_202	sample	16702	16702	16702	16613	16071	15505	13408	13408	19,722
sample_203	sample	15336	15336	15336	15236	14836	14327	12570	12570	18,036
sample_204	sample	24768	24768	24768	24660	24138	23448	22350	22350	9,7626
sample_205	sample	4794	4794	4794	4766	4605	4399	3988	3988	16,813
sample_206	sample	15415	15415	15415	15282	14793	14310	12410	12410	19,494
sample_207	sample	9616	9616	9616	9577	9169	8847	7503	7503	21,974
sample_208	sample	17950	17950	17950	17851	17239	16643	14241	14241	20,663
sample_209	sample	11119	11119	11119	10948	10458	10142	8558	8558	23,033
sample_210	sample	11368	11368	11368	11307	10936	10634	9517	9517	16,283
sample_211	sample	19952	19952	19952	19863	19235	18515	16360	16360	18,003
sample_212	sample	18066	18066	18066	17981	17494	16929	15096	15096	16,44
sample_213	sample	15196	15196	15196	15106	14645	13962	12217	12217	19,604
sample_214	sample	18718	18718	18718	18613	18075	17136	15063	15063	19,527
sample_215	sample	12916	12916	12916	12863	12472	12080	10751	10751	16,762
sample_216	sample	13396	13396	13396	13326	12732	12319	10253	10253	23,462
sample_217	sample	19579	19579	19579	19459	18996	18189	16196	16196	17,279
sample_218	sample	13491	13491	13491	13412	12945	12446	10708	10708	20,629
sample_219	sample	11265	11265	11265	11222	11033	10570	10008	10008	11,158
sample_220	sample	9817	9817	9817	9663	9392	9010	8282	8282	15,636
sample_221	sample	15525	15525	15525	15451	15086	14454	13047	13047	15,961
sample_222	sample	14505	14505	14505	14403	13867	13466	11452	11452	21,048
sample_223	sample	13449	13449	13449	13362	13024	12614	11155	11155	17,057
sample_224	sample	20272	20272	20272	20117	19228	18431	14711	14711	27,432
sample_225	sample	18263	18263	18263	18155	17472	16905	14081	14081	22,899
sample_226	sample	14020	14020	14020	13959	13605	13146	11969	11969	14,629
sample_227	sample	13710	13710	13710	13628	13170	12625	10690	10690	22,028
sample_228	sample	13417	13417	13417	13327	12869	12379	10317	10317	23,105
sample_229	sample	13853	13853	13853	13780	13225	12806	10577	10577	23,648
sample_230	sample	17176	17176	17176	17039	16645	15851	14242	14242	17,082
sample_231	sample	17280	17280	17280	17210	16644	15993	13849	13849	19,855
sample_232	sample	20906	20906	20906	20784	20218	19482	16498	16498	21,085
sample_233	sample	8098	8098	8098	8051	7772	7471	6788	6788	16,177
sample_234	sample	11813	11813	11813	11732	11410	11047	9963	9963	15,661
sample_235	sample	17933	17933	17933	17841	17284	16526	14475	14475	19,283
sample_236	sample	19237	19237	19237	19142	18476	17705	14803	14803	23,049
sample_237	sample	14454	14454	14454	14340	13921	13346	11459	11459	20,721
sample_238	sample	12841	12841	12841	12774	12312	12005	10251	10251	20,17
sample_239	sample	3483	3483	3483	3465	3365	3237	2991	2991	14,126
sample_240	sample	6805	6805	6805	6769	6536	6294	5767	5767	15,253
sample_241	sample	16020	16020	16020	15908	15338	14688	12508	12508	21,923
sample_242	sample	16611	16611	16611	16513	15944	15411	13093	13093	21,179
sample_243	sample	14534	14534	14534	14460	13983	13583	11529	11529	20,676
sample_244	sample	18788	18788	18788	18712	18168	17600	14927	14927	20,55
sample_245	sample	12758	12758	12758	12675	12162	11723	9910	9910	22,323
sample_246	sample	14567	14567	14567	14481	13905	13392	11287	11287	22,517
sample_247	sample	15797	15797	15797	15711	15158	14651	12500	12500	20,871
sample_248	sample	15078	15078	15078	15010	14514	14014	12177	12177	19,24
sample_249	sample	16890	16890	16890	16796	16337	15491	13354	13354	20,935
sample_250	sample	20173	20173	20173	20087	19322	18578	14899	14899	26,144
sample_251	sample	15008	15008	15008	14934	14476	13926	12096	12096	19,403

sample_252	sample	22044	22044	22044	21880	21401	20507	18465	18465	16,236
sample_253	sample	17473	17473	17473	17377	16768	16237	14001	14001	19,871
sample_254	sample	4574	4574	4574	4548	4386	4202	3780	3780	17,359
sample_255	sample	17698	17698	17698	17601	17107	16472	14827	14827	16,222
sample_256	sample	13296	13296	13296	13234	12809	12375	10644	10644	19,946
sample_257	sample	13130	13130	13130	13060	12590	12145	10485	10485	20,145
sample_258	sample	18222	18222	18222	18120	17546	16916	14523	14523	20,3
sample_259	sample	8913	8913	8913	8876	8541	8144	6925	6925	22,304
sample_260	sample	16765	16765	16765	16695	16265	15612	13813	13813	17,608
sample_261	sample	12246	12246	12246	12174	11888	11398	10333	10333	15,621
sample_262	sample	14870	14870	14870	14803	14410	13930	11843	11843	20,356
sample_263	sample	18406	18406	18406	18301	17774	17184	15220	15220	17,31
sample_264	sample	8046	8046	8046	8008	7755	7381	6474	6474	19,538
sample_265	sample	10045	10045	10045	9999	9738	9392	8519	8519	15,192
sample_266	sample	15868	15868	15868	15768	15307	14687	12836	12836	19,108
sample_267	sample	16330	16330	16330	16225	15794	15199	13497	13497	17,348
sample_268	sample	16256	16256	16256	16180	15691	15166	13314	13314	18,098
sample_269	sample	11327	11327	11327	11285	10920	10435	9163	9163	19,105
sample_270	sample	17312	17312	17312	17242	16631	16030	13273	13273	23,331
sample_271	sample	20590	20590	20590	20499	19878	19174	16927	16927	17,79
sample_272	sample	16790	16790	16790	16716	16407	15741	14341	14341	14,586
sample_273	sample	13920	13920	13920	13851	13383	12995	10965	10965	21,228
sample_274	sample	17152	17152	17152	17054	16415	15980	13327	13327	22,301
sample_275	sample	9072	9072	9072	9022	8708	8296	7165	7165	21,021
sample_276	sample	14634	14634	14634	14563	14125	13569	12011	12011	17,924
sample_277	sample	19783	19783	19783	19694	18953	18290	15550	15550	21,397
sample_278	sample	10258	10258	10258	10211	9839	9478	8138	8138	20,667
sample_279	sample	15355	15355	15355	15298	14721	14337	12268	12268	20,104
sample_280	sample	13356	13356	13356	13269	12850	12295	10567	10567	20,882
sample_281	sample	19251	19251	19251	19077	18534	17718	15381	15381	20,103
sample_282	sample	15409	15409	15409	15306	14868	14273	12568	12568	18,437
sample_283	sample	13414	13414	13414	13351	12894	12395	11015	11015	17,884
sample_284	sample	13565	13565	13565	13452	13104	12553	11141	11141	17,87
sample_285	sample	33099	33099	33099	32967	32239	31123	27698	27698	16,318
sample_286	sample	11652	11652	11652	11566	11223	10914	9939	9939	14,701
sample_287	sample	15876	15876	15876	15774	15183	14712	12737	12737	19,772
sample_288	sample	13341	13341	13341	13252	12708	12337	10124	10124	24,114
sample_289	sample	20319	20319	20319	20219	19642	18849	16519	16519	18,702
sample_290	sample	15929	15929	15929	15846	15381	14817	12719	12719	20,152
sample_291	sample	15442	15442	15442	15368	14884	14343	12122	12122	21,5
sample_292	sample	13872	13872	13872	13813	13416	12936	11356	11356	18,137
sample_293	sample	14052	14052	14052	13962	13559	13080	11815	11815	15,919
sample_294	sample	11827	11827	11827	11765	11377	10864	9307	9307	21,307
sample_295	sample	13638	13638	13638	13536	13007	12699	10781	10781	20,949
sample_296	sample	12922	12922	12922	12855	12585	12148	11224	11224	13,14
sample_297	sample	14837	14837	14837	14753	14265	13707	11406	11406	23,125
sample_298	sample	15329	15329	15329	15264	14811	14226	12315	12315	19,662
sample_299	sample	15179	15179	15179	15102	14612	14068	11764	11764	22,498
sample_300	sample	17482	17482	17482	17404	16853	16175	14170	14170	18,945
sample_301	sample	19440	19440	19440	19171	18506	17597	15348	15348	21,049
sample_302	sample	29398	29398	29398	29278	28835	27842	26642	26642	9,3748
sample_303	sample	11170	11170	11170	11115	10729	10461	8859	8859	20,689
sample_304	sample	16800	16800	16800	16659	16257	15731	14015	14015	16,577
sample_305	sample	16634	16634	16634	16530	15960	15488	13231	13231	20,458
sample_306	sample	14684	14684	14684	14627	14261	13766	12154	12154	17,23
sample_307	sample	15930	15930	15930	15836	15377	14805	12802	12802	19,636
sample_308	sample	17390	17390	17390	17273	16780	16156	14443	14443	16,947
sample_309	sample	4587	4587	4587	4561	4443	4254	3925	3925	14,432
sample_310	sample	13927	13927	13927	13863	13462	12989	11692	11692	16,048
sample_311	sample	20140	20140	20140	20033	19496	18636	16297	16297	19,081
sample_312	sample	2992	2992	2992	2969	2780	2626	2220	2220	25,802
sample_313	sample	13309	13309	13309	13241	12869	12325	10667	10667	19,851
sample_314	sample	12297	12297	12297	12221	11637	11222	9196	9196	25,218
sample_315	sample	17883	17883	17883	17779	17272	16557	14400	14400	19,477
sample_316	sample	15538	15538	15538	15431	14946	14262	12408	12408	20,144
sample_317	sample	17134	17134	17134	17024	16399	15918	13634	13634	20,427

sample_318	sample	15897	15897	15897	15812	15397	14883	13135	13135	17,374
sample_319	sample	9867	9867	9867	9809	9468	9042	7986	7986	19,064
sample_320	sample	12677	12677	12677	12619	12185	11792	10217	10217	19,405
sample_321	sample	11795	11795	11795	11714	11191	10990	9625	9625	18,398
sample_322	sample	11506	11506	11506	11427	11101	10726	9559	9559	16,922
sample_323	sample	17732	17732	17732	17652	16980	16403	13550	13550	23,584
sample_324	sample	651	651	651	646	580	538	429	429	34,101
sample_325	sample	18311	18311	18311	18226	17633	16940	14763	14763	19,376
sample_326	sample	10181	10181	10181	10126	9801	9369	8064	8064	20,794
sample_327	sample	11658	11658	11658	11602	11173	10830	9267	9267	20,51
sample_328	sample	13362	13362	13362	13303	12868	12418	10586	10586	20,775
sample_329	sample	16206	16206	16206	16111	15642	15051	12832	12832	20,819
sample_330	sample	12921	12921	12921	12832	12464	11951	10298	10298	20,3
sample_331	sample	21431	21431	21431	21337	20731	20153	17082	17082	20,293
sample_332	sample	13522	13522	13522	13075	12659	12066	10648	10648	21,254
sample_333	sample	15606	15606	15606	15533	15131	14581	12618	12618	19,146
sample_334	sample	10007	10007	10007	9868	9567	9277	8306	8306	16,998
sample_335	sample	10993	10993	10993	10937	10548	10119	8461	8461	23,033
sample_336	sample	13750	13750	13750	13671	13202	12687	10969	10969	20,225
sample_337	sample	17065	17065	17065	16981	16383	15742	13129	13129	23,065
sample_338	sample	18068	18068	18068	17967	17486	16746	14528	14528	19,593
sample_339	sample	9550	9550	9550	9489	9171	8923	7698	7698	19,393
sample_340	sample	11280	11280	11280	11217	10876	10323	8804	8804	21,95
sample_341	sample	11889	11889	11889	11814	11331	10970	8974	8974	24,518
sample_342	sample	16316	16316	16316	16248	15689	15194	12991	12991	20,379
sample_343	sample	13426	13426	13426	13344	12913	12542	10602	10602	21,034
sample_344	sample	16018	16018	16018	15883	15392	14719	12582	12582	21,451
sample_345	sample	11309	11309	11309	11246	10795	10430	8777	8777	22,389
sample_346	sample	19373	19373	19373	19281	18632	18072	15840	15840	18,237
sample_347	sample	10984	10984	10984	10930	10535	10192	8862	8862	19,319
sample_348	sample	16630	16630	16630	16573	16120	15470	13881	13881	16,53
sample_349	sample	13982	13982	13982	13914	13475	12993	11462	11462	18,023
sample_350	sample	13890	13890	13890	13822	13314	12783	10889	10889	21,605
sample_351	sample	19341	19341	19341	19226	18601	18031	15673	15673	18,965
sample_352	sample	11721	11721	11721	11663	11206	10791	9041	9041	22,865
sample_353	sample	17133	17133	17133	17052	16615	15968	14055	14055	17,965
sample_354	sample	13406	13406	13406	13325	12825	12242	10348	10348	22,811
sample_355	sample	13035	13035	13035	12968	12361	11902	9271	9271	28,876
sample_356	sample	19205	19205	19205	19103	18604	17692	14971	14971	22,046
sample_357	sample	9220	9220	9220	9145	8832	8533	7408	7408	19,653
sample_358	sample	9572	9572	9572	9508	9261	8915	7922	7922	17,238
sample_359	sample	11561	11561	11561	11488	11106	10758	9214	9214	20,301
sample_360	sample	18080	18080	18080	17993	17441	16662	14439	14439	20,138
sample_361	sample	12215	12215	12215	12140	11775	11352	9432	9432	22,783
sample_362	sample	16654	16654	16654	16548	16053	15189	13197	13197	20,758
sample_363	sample	20849	20849	20849	20422	19855	18945	16775	16775	19,541
sample_364	sample	9085	9085	9085	9033	8803	8443	7846	7846	13,638

Sequencing run 3

sampleID	type	rawData	output-Cutadapt	input-DADA2	filtered	denoisedF	denoisedR	merged	seqtab	percLost
blank_control_1	PCR_control	5	5	5	4	3	1	0	0	100
blank_control_2	PCR_control	10	10	10	7	1	1	0	0	100
blank_control_3	PCR_control	6	6	6	4	1	1	0	0	100
blank_control_4	PCR_control	11	11	11	9	1	1	0	0	100
blank_control_5	PCR_control	23	23	23	16	1	1	0	0	100
blank_control_6	PCR_control	7	7	7	5	1	1	0	0	100
blank_control_7	PCR_control	5	5	5	5	1	1	0	0	100
blank_control_8	PCR_control	1	1	1	1	1	1	0	0	100
blank_control_9	PCR_control	10	10	10	8	1	1	0	0	100
blank_control_10	PCR_control	30	30	30	20	1	1	0	0	100
blank_control_11	PCR_control	5	5	5	4	1	1	0	0	100
blank_control_12	PCR_control	18	18	18	11	1	1	0	0	100
blank_control_13	PCR_control	7	7	7	4	1	1	0	0	100
blank_control_14	PCR_control	7	7	7	6	2	2	1	1	85,714

blank_control_15	PCR_control	5	5	5	2	1	1	1	1	80
blank_control_16	PCR_control	5	5	5	3	1	1	0	0	100
blank_control_17	PCR_control	9	9	9	8	1	1	0	0	100
blank_control_18	DNA_extraction_control	81	81	81	75	10	1	0	0	100
blank_control_19	DNA_extraction_control	52	52	52	50	18	1	0	0	100
blank_control_20	DNA_extraction_control	62	62	62	60	12	1	0	0	100
blank_control_21	DNA_extraction_control	9	9	9	8	1	1	0	0	100
blank_control_22	DNA_extraction_control	16	16	16	15	2	1	0	0	100
blank_control_23	DNA_extraction_control	51	51	51	48	17	1	0	0	100
blank_control_24	DNA_extraction_control	75	75	75	65	17	1	0	0	100
blank_control_25	DNA_extraction_control	94	94	94	90	26	1	0	0	100
blank_control_26	DNA_extraction_control	49	49	49	46	2	1	0	0	100
blank_control_27	DNA_extraction_control	84	84	84	78	20	1	0	0	100
blank_control_28	DNA_extraction_control	80	80	80	70	3	1	0	0	100
blank_control_29	DNA_extraction_control	115	115	115	108	32	1	0	0	100
blank_control_30	DNA_extraction_control	48	48	48	46	4	1	0	0	100
blank_control_31	DNA_extraction_control	61	61	61	59	12	1	0	0	100
blank_control_32	DNA_extraction_control	81	81	81	76	8	1	0	0	100
blank_control_33	DNA_extraction_control	86	86	86	80	25	1	0	0	100
blank_control_34	DNA_extraction_control	92	92	92	89	25	14	6	6	93,478
blank_control_35	DNA_extraction_control	59	59	59	55	5	9	0	0	100
blank_control_36	DNA_extraction_control	89	89	89	84	20	1	0	0	100
blank_control_37	DNA_extraction_control	96	96	96	89	25	1	0	0	100
blank_control_38	DNA_extraction_control	51	51	51	48	15	1	0	0	100
blank_control_39	DNA_extraction_control	20	20	20	18	1	1	0	0	100
blank_control_40	DNA_extraction_control	23	23	23	22	5	1	0	0	100
blank_control_41	DNA_extraction_control	104	104	104	100	35	43	15	15	85,577
blank_control_42	DNA_extraction_control	123	123	123	121	60	32	6	6	95,122
blank_control_43	DNA_extraction_control	76	76	76	69	1	1	0	0	100
blank_control_44	DNA_extraction_control	55	55	55	52	7	1	0	0	100
blank_control_45	DNA_extraction_control	66	66	66	59	15	1	0	0	100
blank_control_46	DNA_extraction_control	62	62	62	58	2	3	0	0	100
blank_control_47	DNA_extraction_control	41	41	41	39	2	1	0	0	100
blank_control_48	DNA_extraction_control	96	96	96	88	30	1	0	0	100
blank_control_49	DNA_extraction_control	44	44	44	40	5	1	0	0	100
blank_control_50	DNA_extraction_control	58	58	58	58	4	1	0	0	100
sample_1	sample	14427	14427	14427	14113	13683	13338	12030	12030	16,615
sample_2	sample	15932	15932	15932	15549	15048	14592	12967	12967	18,61
sample_3	sample	12866	12866	12866	12575	12091	11689	9733	9733	24,351
sample_4	sample	13179	13179	13179	12875	12433	12032	10483	10483	20,457
sample_5	sample	14044	14044	14044	13734	13240	12868	11035	11035	21,426
sample_6	sample	18189	18189	18189	17742	17196	16660	14444	14444	20,589
sample_7	sample	17898	17898	17898	17471	16876	16393	14266	14266	20,293
sample_8	sample	10674	10674	10674	10417	10005	9657	8336	8336	21,904
sample_9	sample	15084	15084	15084	14704	14196	13822	12229	12229	18,927
sample_10	sample	20994	20994	20994	20528	19892	19285	17068	17068	18,701
sample_11	sample	16163	16163	16163	15744	15275	14833	13138	13138	18,716
sample_12	sample	11997	11997	11997	11709	11346	10954	9669	9669	19,405
sample_13	sample	13983	13983	13983	13684	13160	12869	10937	10937	21,784
sample_14	sample	11932	11932	11932	11661	11389	11057	10074	10074	15,572
sample_15	sample	20237	20237	20237	19540	18878	18187	15819	15819	21,831
sample_16	sample	15653	15653	15653	15301	14764	14334	12354	12354	21,076
sample_17	sample	8400	8400	8400	8206	7989	7717	7080	7080	15,714
sample_18	sample	16419	16419	16419	16056	15517	14924	13041	13041	20,574
sample_19	sample	10261	10261	10261	9995	9604	9288	7964	7964	22,386
sample_20	sample	14061	14061	14061	13707	13221	12809	10774	10774	23,377
sample_21	sample	13904	13904	13904	13548	13126	12662	11404	11404	17,98
sample_22	sample	21182	21182	21182	20729	20019	19562	16006	16006	24,436
sample_23	sample	17217	17217	17217	16815	16216	15742	13842	13842	19,603
sample_24	sample	11817	11817	11817	11502	11131	10781	9495	9495	19,65
sample_25	sample	16665	16665	16665	16279	15810	15225	13239	13239	20,558
sample_26	sample	16814	16814	16814	16412	15812	15299	12833	12833	23,677
sample_27	sample	13900	13900	13900	13553	13123	12734	10908	10908	21,525
sample_28	sample	17123	17123	17123	16784	16316	15728	14030	14030	18,063
sample_29	sample	10893	10893	10893	10588	10231	9895	8719	8719	19,958
sample_30	sample	18589	18589	18589	18189	17673	17204	15286	15286	17,769

sample_31	sample	11569	11569	11569	11259	10873	10547	9469	9469	18,152
sample_32	sample	15458	15458	15458	15081	14565	14171	12054	12054	22,021
sample_33	sample	17919	17919	17919	17488	16853	16376	14108	14108	21,268
sample_34	sample	13916	13916	13916	13565	13090	12643	10675	10675	23,29
sample_35	sample	11362	11362	11362	11081	10731	10370	9236	9236	18,711
sample_36	sample	17972	17972	17972	17491	16923	16295	13954	13954	22,357
sample_37	sample	16676	16676	16676	16307	15794	15334	13281	13281	20,359
sample_38	sample	12442	12442	12442	12157	11745	11338	9945	9945	20,069
sample_39	sample	12970	12970	12970	12672	12378	12097	10939	10939	15,659
sample_40	sample	17450	17450	17450	17076	16437	15904	13530	13530	22,464
sample_41	sample	14022	14022	14022	13719	13243	12846	11293	11293	19,462
sample_42	sample	18239	18239	18239	17829	17282	16729	14931	14931	18,137
sample_43	sample	14706	14706	14706	14348	13823	13443	11627	11627	20,937
sample_44	sample	11471	11471	11471	11198	10751	10362	8681	8681	24,322
sample_45	sample	16109	16109	16109	15715	15085	14670	12569	12569	21,975
sample_46	sample	15752	15752	15752	15417	14825	14521	12419	12419	21,159
sample_47	sample	16321	16321	16321	15948	15461	14991	13205	13205	19,092
sample_48	sample	14544	14544	14544	14194	13755	13246	11624	11624	20,077
sample_49	sample	15974	15974	15974	15533	14888	14516	12433	12433	22,167
sample_50	sample	13187	13187	13187	12696	12276	11896	10703	10703	18,837
sample_51	sample	11458	11458	11458	11198	10939	10523	9465	9465	17,394
sample_52	sample	12333	12333	12333	12037	11582	11367	9872	9872	19,955
sample_53	sample	17623	17623	17623	17250	16592	16010	13306	13306	24,496
sample_54	sample	11674	11674	11674	11280	10920	10602	9471	9471	18,871
sample_55	sample	14269	14269	14269	13948	13384	13093	11309	11309	20,744
sample_56	sample	13175	13175	13175	12829	12425	12051	10295	10295	21,86
sample_57	sample	15304	15304	15304	14977	14369	13981	12085	12085	21,034
sample_58	sample	15964	15964	15964	15574	15041	14590	12466	12466	21,912
sample_59	sample	11435	11435	11435	11172	10720	10307	8807	8807	22,982
sample_60	sample	14437	14437	14437	14115	13583	13146	11322	11322	21,577
sample_61	sample	14691	14691	14691	13215	12709	12183	10507	10507	28,48
sample_62	sample	10294	10294	10294	10074	9634	9259	7911	7911	23,149
sample_63	sample	14738	14738	14738	14422	13955	13562	11567	11567	21,516
sample_64	sample	13579	13579	13579	13253	12818	12401	10925	10925	19,545
sample_65	sample	14643	14643	14643	14297	13811	13359	11668	11668	20,317
sample_66	sample	11094	11094	11094	10813	10426	10159	8879	8879	19,966
sample_67	sample	18715	18715	18715	18314	17768	17137	15073	15073	19,46
sample_68	sample	14981	14981	14981	14634	14133	13731	12157	12157	18,851
sample_69	sample	16354	16354	16354	15967	15260	14766	12285	12285	24,881
sample_70	sample	12467	12467	12467	12168	11708	11294	9688	9688	22,291
sample_71	sample	25725	25725	25725	25173	24559	23804	21477	21477	16,513
sample_72	sample	14479	14479	14479	14156	13805	13354	12084	12084	16,541
sample_73	sample	17457	17457	17457	17039	16460	15825	13374	13374	23,389
sample_74	sample	14113	14113	14113	13770	13409	12922	11532	11532	18,288
sample_75	sample	14619	14619	14619	14258	13762	13369	11828	11828	19,092
sample_76	sample	19936	19936	19936	19478	18915	18230	15868	15868	20,405
sample_77	sample	13416	13416	13416	13120	12661	12388	10494	10494	21,78
sample_78	sample	15635	15635	15635	15286	14758	14397	12106	12106	22,571
sample_79	sample	16102	16102	16102	15724	15110	14773	12550	12550	22,059
sample_80	sample	17194	17194	17194	16816	16198	15758	13869	13869	19,338
sample_81	sample	25054	25054	25054	24513	23568	22875	18767	18767	25,094
sample_82	sample	19252	19252	19252	18813	18257	17595	15641	15641	18,756
sample_83	sample	15340	15340	15340	14967	14415	13966	11432	11432	25,476
sample_84	sample	19944	19944	19944	19463	18754	18107	15685	15685	21,355
sample_85	sample	8406	8406	8406	8214	7954	7684	7144	7144	15,013
sample_86	sample	13514	13514	13514	13188	12759	12407	10503	10503	22,281
sample_87	sample	21941	21941	21941	21323	20616	19891	17416	17416	20,623
sample_88	sample	14164	14164	14164	13823	13447	12976	11638	11638	17,834
sample_89	sample	11909	11909	11909	11611	11257	10805	9519	9519	20,069
sample_90	sample	9440	9440	9440	9229	8930	8639	7873	7873	16,6
sample_91	sample	13743	13743	13743	13442	13078	12617	10950	10950	20,323
sample_92	sample	26294	26294	26294	25698	25048	24312	21957	21957	16,494
sample_93	sample	16533	16533	16533	16173	15547	15094	13003	13003	21,351
sample_94	sample	11466	11466	11466	11201	10784	10509	9221	9221	19,58
sample_95	sample	18734	18734	18734	18277	17726	17308	15348	15348	18,074
sample_96	sample	12829	12829	12829	12551	12218	11828	10385	10385	19,051

sample_97	sample	6819	6819	6819	6645	6245	6121	4892	4892	28,259
sample_98	sample	13681	13681	13681	13367	12923	12470	11126	11126	18,676
sample_99	sample	20627	20627	20627	20128	19526	18763	16102	16102	21,937
sample_100	sample	19521	19521	19521	19064	18492	18046	15741	15741	19,364
sample_101	sample	13037	13037	13037	12742	12380	11902	10322	10322	20,825
sample_102	sample	17964	17964	17964	17561	16903	16512	13912	13912	22,556
sample_103	sample	22476	22476	22476	21977	21448	20889	19526	19526	13,125
sample_104	sample	22517	22517	22517	22007	21311	20603	18015	18015	19,994
sample_105	sample	18021	18021	18021	17619	17021	16525	14051	14051	22,03
sample_106	sample	15822	15822	15822	15486	14929	14522	12454	12454	21,287
sample_107	sample	16121	16121	16121	15770	15139	14684	11902	11902	26,171
sample_108	sample	15270	15270	15270	14909	14289	13869	11684	11684	23,484
sample_109	sample	14251	14251	14251	13914	13537	13173	11696	11696	17,929
sample_110	sample	11117	11117	11117	10790	10423	10115	8772	8772	21,094
sample_111	sample	11309	11309	11309	11071	10638	10376	9280	9280	17,941
sample_112	sample	11884	11884	11884	11557	11186	10824	9589	9589	19,312
sample_113	sample	14109	14109	14109	13781	13280	12828	10808	10808	23,396
sample_114	sample	19147	19147	19147	18740	18071	17481	14815	14815	22,625
sample_115	sample	17458	17458	17458	17031	16361	15950	13522	13522	22,546
sample_116	sample	11032	11032	11032	10781	10349	10105	8768	8768	20,522
sample_117	sample	14041	14041	14041	13727	13237	12897	11261	11261	19,799
sample_118	sample	18029	18029	18029	17601	17112	16577	14872	14872	17,511
sample_119	sample	20472	20472	20472	20035	19248	18644	15507	15507	24,253
sample_120	sample	18097	18097	18097	17687	17077	16460	14288	14288	21,048
sample_121	sample	23448	23448	23448	22906	22465	21885	19842	19842	15,379
sample_122	sample	10766	10766	10766	10494	10024	9627	7893	7893	26,686
sample_123	sample	14968	14968	14968	14592	14027	13708	11460	11460	23,437
sample_124	sample	9341	9341	9341	9150	8889	8593	7830	7830	16,176
sample_125	sample	22046	22046	22046	21576	20859	20129	17075	17075	22,548
sample_126	sample	15435	15435	15435	15110	14649	14249	12848	12848	16,761
sample_127	sample	17275	17275	17275	16897	16424	15875	13861	13861	19,763
sample_128	sample	13408	13408	13408	13138	12551	12121	9965	9965	25,679
sample_129	sample	23282	23282	23282	22776	22104	21422	18524	18524	20,436
sample_130	sample	17363	17363	17363	16929	16349	15937	13920	13920	19,83
sample_131	sample	20046	20046	20046	19617	19172	18533	17760	17760	11,404
sample_132	sample	14095	14095	14095	13763	13169	12791	10588	10588	24,881
sample_133	sample	21983	21983	21983	21465	20916	20304	19361	19361	11,927
sample_134	sample	13915	13915	13915	13572	13098	12738	11068	11068	20,46
sample_135	sample	21748	21748	21748	21243	20467	19727	16387	16387	24,651
sample_136	sample	15009	15009	15009	14649	14149	13744	11860	11860	20,981
sample_137	sample	19383	19383	19383	18932	18357	17811	15096	15096	22,117
sample_138	sample	11477	11477	11477	11171	10756	10485	9049	9049	21,155
sample_139	sample	18071	18071	18071	17619	16946	16577	14043	14043	22,29
sample_140	sample	9350	9350	9350	9104	8758	8530	7475	7475	20,053
sample_141	sample	9573	9573	9573	9370	9000	8659	7619	7619	20,412
sample_142	sample	12982	12982	12982	12692	12179	11761	10227	10227	21,222
sample_143	sample	14505	14505	14505	14168	13606	13220	11177	11177	22,944
sample_144	sample	10104	10104	10104	9882	9583	9364	8793	8793	12,975
sample_145	sample	14520	14520	14520	14147	13624	13162	11207	11207	22,817
sample_146	sample	11659	11659	11659	11415	11049	10700	9316	9316	20,096
sample_147	sample	17014	17014	17014	16650	16006	15477	12944	12944	23,921
sample_148	sample	16106	16106	16106	15762	15183	14797	12346	12346	23,345
sample_149	sample	10672	10672	10672	10412	10044	9751	8797	8797	17,569
sample_150	sample	10477	10477	10477	10230	9949	9668	8449	8449	19,357
sample_151	sample	17168	17168	17168	16828	16286	15833	14148	14148	17,591
sample_152	sample	15507	15507	15507	15153	14638	14162	12639	12639	18,495
sample_153	sample	13243	13243	13243	12950	12399	12172	10484	10484	20,834
sample_154	sample	8582	8582	8582	8379	8080	7849	6880	6880	19,832
sample_155	sample	17605	17605	17605	17180	16535	16139	13877	13877	21,176
sample_156	sample	17122	17122	17122	16724	16154	15633	13743	13743	19,735
sample_157	sample	17953	17953	17953	17504	16849	16457	14413	14413	19,718
sample_158	sample	13327	13327	13327	12917	12494	12002	10625	10625	20,275
sample_159	sample	14371	14371	14371	14010	13637	13118	12025	12025	16,325
sample_160	sample	14094	14094	14094	13754	13405	12929	11558	11558	17,993
sample_161	sample	13327	13327	13327	12992	12514	12221	10532	10532	20,972
sample_162	sample	19267	19267	19267	18796	18115	17628	14431	14431	25,1

sample_163	sample	11143	11143	11143	10895	10589	10213	9263	9263	16,872
sample_164	sample	13053	13053	13053	12779	12309	11893	10195	10195	21,895
sample_165	sample	14876	14876	14876	14551	13886	13652	11267	11267	24,261
sample_166	sample	15406	15406	15406	15027	14748	14351	13746	13746	10,775
sample_167	sample	11785	11785	11785	11535	11069	10827	9051	9051	23,199
sample_168	sample	14120	14120	14120	12827	12504	12069	11132	11132	21,161
sample_169	sample	9744	9744	9744	9507	9252	8865	8302	8302	14,799
sample_170	sample	10350	10350	10350	10082	9657	9440	8014	8014	22,57
sample_171	sample	10446	10446	10446	10182	9738	9503	8272	8272	20,812
sample_172	sample	17228	17228	17228	16827	16216	15755	13522	13522	21,511
sample_173	sample	13862	13862	13862	13483	13047	12575	11219	11219	19,067
sample_174	sample	17199	17199	17199	16824	16151	15743	13216	13216	23,158
sample_175	sample	16235	16235	16235	15820	15423	14770	13057	13057	19,575
sample_176	sample	22276	22276	22276	21752	21064	20341	17830	17830	19,959
sample_177	sample	18245	18245	18245	17846	17316	16754	14145	14145	22,472
sample_178	sample	16117	16117	16117	15750	14943	14650	11764	11764	27,009
sample_179	sample	14241	14241	14241	13870	13360	12922	10805	10805	24,128
sample_180	sample	7562	7562	7562	7395	7133	6895	5963	5963	21,145
sample_181	sample	14477	14477	14477	14168	13644	13248	11108	11108	23,271
sample_182	sample	22464	22464	22464	21935	21338	20652	18100	18100	19,427
sample_183	sample	13766	13766	13766	13450	12971	12545	10759	10759	21,844
sample_184	sample	14798	14798	14798	14469	13926	13699	11793	11793	20,307
sample_185	sample	23032	23032	23032	22537	21721	21036	17587	17587	23,641
sample_186	sample	18255	18255	18255	17840	17160	16769	14145	14145	22,514
sample_187	sample	17965	17965	17965	17567	17028	16519	14246	14246	20,701
sample_188	sample	15701	15701	15701	15344	14915	14428	12792	12792	18,527
sample_189	sample	11559	11559	11559	10700	10333	9996	9110	9110	21,187
sample_190	sample	11114	11114	11114	10805	10496	10146	9154	9154	17,635
sample_191	sample	19222	19222	19222	18748	18101	17567	15433	15433	19,712
sample_192	sample	13123	13123	13123	12836	12436	12049	10848	10848	17,336
sample_193	sample	16041	16041	16041	15711	15106	14495	12089	12089	24,637
sample_194	sample	12779	12779	12779	12490	12022	11687	10282	10282	19,54
sample_195	sample	21662	21662	21662	21096	20282	19779	16673	16673	23,031
sample_196	sample	11931	11931	11931	11588	11194	10866	10091	10091	15,422
sample_197	sample	12839	12839	12839	12539	12043	11693	10141	10141	21,014
sample_198	sample	12284	12284	12284	11988	11559	11345	10477	10477	14,71
sample_199	sample	15128	15128	15128	14761	14229	13774	11848	11848	21,682
sample_200	sample	13333	13333	13333	13033	12569	12237	10252	10252	23,108
sample_201	sample	11183	11183	11183	10878	10548	10351	9070	9070	18,895
sample_202	sample	16863	16863	16863	16516	15981	15318	13211	13211	21,657
sample_203	sample	15424	15424	15424	15068	14545	14254	12478	12478	19,1
sample_204	sample	17347	17347	17347	16956	16510	16026	15274	15274	11,95
sample_205	sample	3090	3090	3090	3024	2849	2765	2477	2477	19,838
sample_206	sample	15864	15864	15864	15479	14917	14430	12480	12480	21,331
sample_207	sample	9774	9774	9774	9559	9142	8913	7609	7609	22,151
sample_208	sample	18059	18059	18059	17658	16989	16544	14163	14163	21,574
sample_209	sample	11353	11353	11353	10981	10535	10154	8572	8572	24,496
sample_210	sample	11455	11455	11455	11194	10820	10542	9364	9364	18,254
sample_211	sample	20479	20479	20479	20002	19300	18598	16459	16459	19,63
sample_212	sample	18198	18198	18198	17761	17265	16754	14910	14910	18,068
sample_213	sample	15558	15558	15558	15182	14599	14125	12270	12270	21,134
sample_214	sample	19000	19000	19000	18562	17907	17187	15060	15060	20,737
sample_215	sample	12976	12976	12976	12666	12255	11859	10594	10594	18,357
sample_216	sample	13517	13517	13517	13225	12679	12320	10274	10274	23,992
sample_217	sample	20065	20065	20065	19599	19056	18367	16314	16314	18,694
sample_218	sample	13394	13394	13394	13083	12641	12249	10540	10540	21,308
sample_219	sample	11414	11414	11414	11156	10903	10517	9858	9858	13,632
sample_220	sample	9926	9926	9926	9621	9342	9007	8299	8299	16,391
sample_221	sample	15861	15861	15861	15523	15039	14607	13161	13161	17,023
sample_222	sample	14874	14874	14874	14498	14019	13564	11468	11468	22,899
sample_223	sample	13626	13626	13626	13310	12908	12552	11036	11036	19,008
sample_224	sample	21045	21045	21045	20484	19596	18904	14880	14880	29,294
sample_225	sample	18104	18104	18104	17694	17096	16600	13953	13953	22,929
sample_226	sample	14150	14150	14150	13807	13389	13066	11811	11811	16,53
sample_227	sample	13966	13966	13966	13661	13141	12642	10727	10727	23,192
sample_228	sample	13577	13577	13577	13237	12705	12364	10302	10302	24,122

sample_229	sample	13831	13831	13831	13536	13079	12595	10422	10422	24,648
sample_230	sample	17515	17515	17515	17063	16542	15969	14207	14207	18,887
sample_231	sample	17450	17450	17450	17044	16457	15961	13928	13928	20,183
sample_232	sample	21430	21430	21430	20954	20265	19708	16758	16758	21,801
sample_233	sample	8182	8182	8182	7983	7677	7389	6673	6673	18,443
sample_234	sample	12011	12011	12011	11724	11417	11087	10079	10079	16,085
sample_235	sample	18212	18212	18212	17762	17119	16612	14454	14454	20,635
sample_236	sample	19951	19951	19951	19521	18728	18172	15172	15172	23,954
sample_237	sample	14515	14515	14515	14156	13650	13301	11502	11502	20,758
sample_238	sample	13076	13076	13076	12774	12323	11933	10304	10304	21,199
sample_239	sample	2173	2173	2173	2110	2019	1932	1763	1763	18,868
sample_240	sample	6939	6939	6939	6774	6477	6212	5736	5736	17,337
sample_241	sample	16235	16235	16235	15861	15232	14690	12479	12479	23,135
sample_242	sample	16682	16682	16682	16284	15687	15145	12921	12921	22,545
sample_243	sample	14678	14678	14678	14340	13924	13477	11460	11460	21,924
sample_244	sample	18837	18837	18837	18425	17781	17338	14538	14538	22,822
sample_245	sample	13088	13088	13088	12718	12189	11865	9962	9962	23,884
sample_246	sample	14776	14776	14776	14445	13919	13515	11564	11564	21,738
sample_247	sample	16181	16181	16181	15795	15246	14759	12795	12795	20,926
sample_248	sample	15458	15458	15458	15113	14579	14087	12301	12301	20,423
sample_249	sample	16203	16203	16203	15797	15266	14730	12563	12563	22,465
sample_250	sample	20779	20779	20779	20320	19451	18863	15131	15131	27,181
sample_251	sample	14989	14989	14989	14640	14182	13665	11966	11966	20,168
sample_252	sample	22622	22622	22622	22093	21705	20851	18975	18975	16,121
sample_253	sample	18000	18000	18000	17560	16922	16368	14241	14241	20,883
sample_254	sample	3014	3014	3014	2930	2779	2680	2433	2433	19,277
sample_255	sample	18276	18276	18276	17875	17368	16796	15111	15111	17,318
sample_256	sample	13474	13474	13474	13153	12669	12279	10615	10615	21,219
sample_257	sample	13685	13685	13685	13358	12889	12488	10684	10684	21,929
sample_258	sample	18436	18436	18436	18027	17352	16960	14422	14422	21,773
sample_259	sample	9368	9368	9368	9142	8749	8500	7210	7210	23,036
sample_260	sample	16765	16765	16765	16383	15878	15387	13459	13459	19,72
sample_261	sample	12190	12190	12190	11894	11521	11175	10034	10034	17,687
sample_262	sample	15385	15385	15385	15012	14439	14128	11829	11829	23,113
sample_263	sample	18567	18567	18567	18118	17524	16993	15079	15079	18,786
sample_264	sample	8238	8238	8238	8054	7762	7490	6536	6536	20,66
sample_265	sample	10428	10428	10428	10202	9913	9616	8730	8730	16,283
sample_266	sample	15927	15927	15927	15542	15025	14583	12744	12744	19,985
sample_267	sample	16682	16682	16682	16326	15829	15389	13535	13535	18,865
sample_268	sample	16815	16815	16815	16450	15923	15362	13464	13464	19,929
sample_269	sample	11583	11583	11583	11300	10875	10503	9212	9212	20,47
sample_270	sample	17426	17426	17426	17018	16340	15897	13051	13051	25,106
sample_271	sample	20839	20839	20839	20373	19780	19222	16889	16889	18,955
sample_272	sample	17228	17228	17228	16865	16421	15850	14426	14426	16,264
sample_273	sample	13787	13787	13787	13458	12981	12629	10576	10576	23,29
sample_274	sample	18072	18072	18072	17716	17066	16643	14094	14094	22,012
sample_275	sample	9167	9167	9167	8954	8623	8256	7106	7106	22,483
sample_276	sample	14984	14984	14984	14657	14196	13771	12280	12280	18,046
sample_277	sample	19627	19627	19627	19163	18373	17862	15156	15156	22,78
sample_278	sample	10369	10369	10369	10140	9774	9441	8181	8181	21,101
sample_279	sample	15409	15409	15409	15041	14525	14047	12210	12210	20,761
sample_280	sample	13391	13391	13391	13086	12604	12119	10311	10311	23,001
sample_281	sample	19384	19384	19384	18867	18287	17578	15476	15476	20,161
sample_282	sample	15343	15343	15343	14971	14437	13992	12245	12245	20,192
sample_283	sample	13853	13853	13853	13555	13130	12709	11460	11460	17,274
sample_284	sample	13813	13813	13813	13470	13074	12642	11307	11307	18,142
sample_285	sample	33736	33736	33736	32930	32110	31044	27872	27872	17,382
sample_286	sample	11775	11775	11775	11454	11149	10745	9831	9831	16,51
sample_287	sample	16121	16121	16121	15747	15218	14732	12801	12801	20,594
sample_288	sample	13386	13386	13386	13054	12484	12157	9998	9998	25,31
sample_289	sample	20639	20639	20639	20163	19532	18861	16603	16603	19,555
sample_290	sample	16348	16348	16348	15923	15446	14903	12796	12796	21,727
sample_291	sample	15886	15886	15886	15505	14945	14448	12188	12188	23,278
sample_292	sample	14001	14001	14001	13684	13166	12903	11272	11272	19,491
sample_293	sample	13914	13914	13914	13585	13115	12737	11395	11395	18,104
sample_294	sample	12320	12320	12320	12047	11568	11151	9629	9629	21,843

sample_295	sample	14026	14026	14026	13694	13143	12802	10823	10823	22,836
sample_296	sample	13290	13290	13290	12974	12649	12296	11367	11367	14,47
sample_297	sample	15143	15143	15143	14765	14245	13712	11394	11394	24,757
sample_298	sample	15426	15426	15426	15060	14474	13953	12082	12082	21,678
sample_299	sample	15586	15586	15586	15234	14617	14282	11887	11887	23,733
sample_300	sample	17638	17638	17638	17204	16602	16057	13884	13884	21,284
sample_301	sample	19516	19516	19516	18878	18281	17533	15286	15286	21,675
sample_302	sample	20634	20634	20634	20167	19701	19215	18194	18194	11,825
sample_303	sample	11668	11668	11668	11416	10967	10758	8959	8959	23,217
sample_304	sample	17229	17229	17229	16787	16389	15849	14189	14189	17,645
sample_305	sample	16848	16848	16848	16452	15955	15428	13113	13113	22,169
sample_306	sample	14719	14719	14719	14369	13967	13515	12011	12011	18,398
sample_307	sample	16001	16001	16001	15593	15062	14620	12586	12586	21,342
sample_308	sample	17552	17552	17552	17102	16585	15983	14233	14233	18,91
sample_309	sample	2943	2943	2943	2879	2753	2692	2454	2454	16,616
sample_310	sample	14434	14434	14434	14091	13698	13243	11905	11905	17,521
sample_311	sample	20552	20552	20552	20053	19436	18779	16250	16250	20,932
sample_312	sample	1893	1893	1893	1837	1705	1592	1328	1328	29,847
sample_313	sample	13540	13540	13540	13228	12770	12407	10750	10750	20,606
sample_314	sample	12582	12582	12582	12287	11731	11349	9410	9410	25,211
sample_315	sample	18005	18005	18005	17568	17027	16524	14482	14482	19,567
sample_316	sample	15817	15817	15817	15437	14890	14294	12383	12383	21,711
sample_317	sample	17407	17407	17407	17018	16432	15936	13762	13762	20,94
sample_318	sample	16316	16316	16316	15955	15450	15084	13165	13165	19,312
sample_319	sample	10127	10127	10127	9893	9530	9191	8146	8146	19,562
sample_320	sample	12844	12844	12844	12548	12056	11762	10106	10106	21,317
sample_321	sample	12401	12401	12401	12126	11591	11425	9978	9978	19,539
sample_322	sample	11415	11415	11415	11138	10795	10517	9347	9347	18,117
sample_323	sample	17781	17781	17781	17380	16702	16183	13388	13388	24,706
sample_324	sample	635	635	635	617	542	519	425	425	33,071
sample_325	sample	18617	18617	18617	18203	17602	17054	14811	14811	20,444
sample_326	sample	10490	10490	10490	10229	9868	9568	8338	8338	20,515
sample_327	sample	12018	12018	12018	11754	11343	10997	9411	9411	21,692
sample_328	sample	13534	13534	13534	13198	12753	12340	10502	10502	22,403
sample_329	sample	16323	16323	16323	15971	15466	14933	12891	12891	21,026
sample_330	sample	12927	12927	12927	12642	12172	11831	10046	10046	22,287
sample_331	sample	21384	21384	21384	20834	20247	19636	16656	16656	22,11
sample_332	sample	13555	13555	13555	12993	12591	12108	10691	10691	21,129
sample_333	sample	16225	16225	16225	15862	15419	14916	12889	12889	20,561
sample_334	sample	9967	9967	9967	9687	9377	9099	8106	8106	18,672
sample_335	sample	11372	11372	11372	11102	10663	10343	8718	8718	23,338
sample_336	sample	13951	13951	13951	13617	13077	12617	10738	10738	23,031
sample_337	sample	16928	16928	16928	16537	15847	15408	12686	12686	25,059
sample_338	sample	18120	18120	18120	17711	17127	16676	14342	14342	20,85
sample_339	sample	9556	9556	9556	9350	9050	8811	7709	7709	19,328
sample_340	sample	11385	11385	11385	11120	10721	10364	8848	8848	22,284
sample_341	sample	12210	12210	12210	11907	11361	11059	9181	9181	24,808
sample_342	sample	16180	16180	16180	15797	15217	14717	12607	12607	22,083
sample_343	sample	13614	13614	13614	13296	12843	12495	10503	10503	22,851
sample_344	sample	16234	16234	16234	15847	15280	14763	12701	12701	21,763
sample_345	sample	11596	11596	11596	11327	10807	10550	8897	8897	23,275
sample_346	sample	19687	19687	19687	19259	18662	17987	15740	15740	20,049
sample_347	sample	11252	11252	11252	10959	10521	10234	8906	8906	20,85
sample_348	sample	17150	17150	17150	16789	16318	15709	14113	14113	17,708
sample_349	sample	14106	14106	14106	13778	13300	12850	11393	11393	19,233
sample_350	sample	14041	14041	14041	13719	13156	12786	10874	10874	22,555
sample_351	sample	19233	19233	19233	18803	18119	17628	15435	15435	19,747
sample_352	sample	11921	11921	11921	11663	11117	10803	9024	9024	24,302
sample_353	sample	17522	17522	17522	17091	16590	16100	14208	14208	18,913
sample_354	sample	13756	13756	13756	13450	12950	12511	10645	10645	22,616
sample_355	sample	13193	13193	13193	12896	12305	11849	9168	9168	30,509
sample_356	sample	19525	19525	19525	19087	18467	17931	15052	15052	22,909
sample_357	sample	9498	9498	9498	9243	8923	8658	7464	7464	21,415
sample_358	sample	9757	9757	9757	9539	9265	8946	7941	7941	18,612
sample_359	sample	11790	11790	11790	11521	11082	10812	9286	9286	21,238
sample_360	sample	18583	18583	18583	18163	17555	16931	14770	14770	20,519

sample_361	sample	12283	12283	12283	11990	11527	11205	9406	9406	23,423
sample_362	sample	16924	16924	16924	16516	15938	15383	13251	13251	21,703
sample_363	sample	21672	21672	21672	20834	20293	19463	17298	17298	20,183
sample_364	sample	9086	9086	9086	8857	8596	8402	7765	7765	14,539

Sequencing run 4

sampleID	type	rawData	output-Cutadapt	input-DADA2	filtered	denoisedF	denoisedR	merged	seqtab	percLost
blank_control_1	PCR_control	9	9	9	8	1	1	0	0	100
blank_control_2	PCR_control	11	11	11	9	3	1	0	0	100
blank_control_3	PCR_control	10	10	10	5	1	1	0	0	100
blank_control_4	PCR_control	6	6	6	6	1	1	0	0	100
blank_control_5	PCR_control	11	11	11	7	1	1	0	0	100
blank_control_6	PCR_control	6	6	6	4	1	1	0	0	100
blank_control_7	PCR_control	2	2	2	2	1	1	0	0	100
blank_control_8	PCR_control	1	1	1	1	1	1	0	0	100
blank_control_9	PCR_control	9	9	9	6	1	1	0	0	100
blank_control_10	PCR_control	26	26	26	18	3	1	0	0	100
blank_control_11	PCR_control	5	5	5	4	1	1	0	0	100
blank_control_12	PCR_control	5	5	5	4	1	1	0	0	100
blank_control_13	PCR_control	5	5	5	2	1	1	0	0	100
blank_control_14	PCR_control	4	4	4	2	1	1	0	0	100
blank_control_15	PCR_control	4	4	4	2	1	1	0	0	100
blank_control_16	PCR_control	9	9	9	6	1	1	0	0	100
blank_control_17	PCR_control	10	10	10	7	1	1	0	0	100
blank_control_18	DNA_extraction_control	76	76	76	70	33	1	0	0	100
blank_control_19	DNA_extraction_control	89	89	89	82	27	1	0	0	100
blank_control_20	DNA_extraction_control	57	57	57	51	2	1	0	0	100
blank_control_21	DNA_extraction_control	15	15	15	12	1	1	0	0	100
blank_control_22	DNA_extraction_control	25	25	25	20	1	1	0	0	100
blank_control_23	DNA_extraction_control	40	40	40	38	1	1	0	0	100
blank_control_24	DNA_extraction_control	51	51	51	43	14	1	0	0	100
blank_control_25	DNA_extraction_control	65	65	65	58	3	3	0	0	100
blank_control_26	DNA_extraction_control	62	62	62	54	13	1	0	0	100
blank_control_27	DNA_extraction_control	69	69	69	60	16	1	0	0	100
blank_control_28	DNA_extraction_control	58	58	58	52	15	1	0	0	100
blank_control_29	DNA_extraction_control	95	95	95	84	25	10	6	6	93,684
blank_control_30	DNA_extraction_control	53	53	53	44	8	1	0	0	100
blank_control_31	DNA_extraction_control	37	37	37	30	1	1	0	0	100
blank_control_32	DNA_extraction_control	54	54	54	51	17	1	0	0	100
blank_control_33	DNA_extraction_control	44	44	44	42	17	1	0	0	100
blank_control_34	DNA_extraction_control	95	95	95	88	20	1	0	0	100
blank_control_35	DNA_extraction_control	53	53	53	48	11	1	0	0	100
blank_control_36	DNA_extraction_control	57	57	57	53	1	1	0	0	100
blank_control_37	DNA_extraction_control	85	85	85	75	16	1	0	0	100
blank_control_38	DNA_extraction_control	53	53	53	50	13	1	0	0	100
blank_control_39	DNA_extraction_control	12	12	12	11	3	1	0	0	100
blank_control_40	DNA_extraction_control	12	12	12	10	1	1	0	0	100
blank_control_41	DNA_extraction_control	97	97	97	94	39	40	27	27	72,165
blank_control_42	DNA_extraction_control	137	137	137	129	66	32	16	16	88,321
blank_control_43	DNA_extraction_control	70	70	70	63	16	1	0	0	100
blank_control_44	DNA_extraction_control	60	60	60	53	22	1	0	0	100
blank_control_45	DNA_extraction_control	92	92	92	80	32	1	0	0	100
blank_control_46	DNA_extraction_control	35	35	35	29	1	1	0	0	100
blank_control_47	DNA_extraction_control	31	31	31	26	11	1	0	0	100
blank_control_48	DNA_extraction_control	116	116	116	108	46	43	20	20	82,759
blank_control_49	DNA_extraction_control	35	35	35	31	6	1	0	0	100
blank_control_50	DNA_extraction_control	50	50	50	46	13	1	0	0	100
sample_1	sample	14013	14013	14013	13041	12598	12264	11092	11092	20,845
sample_2	sample	15380	15380	15380	14292	13933	13352	11897	11897	22,646
sample_3	sample	12284	12284	12284	11451	10987	10604	8810	8810	28,281
sample_4	sample	12528	12528	12528	11650	11331	10911	9661	9661	22,885
sample_5	sample	13455	13455	13455	12518	12017	11762	10153	10153	24,541
sample_6	sample	17896	17896	17896	16589	16128	15518	13326	13326	25,536
sample_7	sample	17214	17214	17214	15984	15481	14949	13062	13062	24,12

sample_8	sample	10359	10359	10359	9669	9328	8953	7675	7675	25,91
sample_9	sample	14917	14917	14917	13848	13459	13032	11603	11603	22,216
sample_10	sample	20323	20323	20323	18860	18323	17639	15664	15664	22,925
sample_11	sample	15491	15491	15491	14342	13936	13434	11910	11910	23,117
sample_12	sample	11380	11380	11380	10607	10296	9972	8705	8705	23,506
sample_13	sample	13556	13556	13556	12670	12201	11777	9985	9985	26,343
sample_14	sample	11755	11755	11755	10964	10709	10398	9421	9421	19,855
sample_15	sample	19300	19300	19300	17762	17212	16599	14456	14456	25,098
sample_16	sample	15450	15450	15450	14318	13838	13449	11610	11610	24,854
sample_17	sample	8060	8060	8060	7505	7311	7009	6381	6381	20,831
sample_18	sample	15881	15881	15881	14761	14282	13855	12121	12121	23,676
sample_19	sample	10206	10206	10206	9489	9088	8760	7334	7334	28,14
sample_20	sample	13682	13682	13682	12730	12246	11929	10016	10016	26,794
sample_21	sample	13382	13382	13382	12498	12165	11604	10593	10593	20,841
sample_22	sample	20450	20450	20450	19017	18352	17756	14559	14559	28,807
sample_23	sample	16999	16999	16999	15874	15395	14931	13111	13111	22,872
sample_24	sample	11576	11576	11576	10752	10414	10150	9013	9013	22,141
sample_25	sample	15916	15916	15916	14818	14356	13845	12011	12011	24,535
sample_26	sample	15997	15997	15997	14933	14478	13883	11651	11651	27,168
sample_27	sample	13227	13227	13227	12326	11928	11551	9973	9973	24,601
sample_28	sample	16949	16949	16949	15805	15323	14900	13123	13123	22,574
sample_29	sample	10522	10522	10522	9715	9458	9131	8113	8113	22,895
sample_30	sample	18237	18237	18237	16979	16574	16080	14343	14343	21,352
sample_31	sample	11321	11321	11321	10566	10248	9896	8966	8966	20,802
sample_32	sample	15062	15062	15062	13966	13466	13093	11272	11272	25,163
sample_33	sample	17544	17544	17544	16349	15749	15365	13167	13167	24,949
sample_34	sample	13515	13515	13515	12535	12169	11724	10031	10031	25,779
sample_35	sample	11078	11078	11078	10325	10074	9707	8633	8633	22,071
sample_36	sample	17464	17464	17464	16310	15835	15210	13231	13231	24,238
sample_37	sample	16281	16281	16281	15170	14767	14199	12269	12269	24,642
sample_38	sample	11836	11836	11836	11022	10708	10204	8962	8962	24,282
sample_39	sample	12345	12345	12345	11515	11252	10966	9903	9903	19,781
sample_40	sample	16595	16595	16595	15504	15072	14513	12546	12546	24,399
sample_41	sample	13322	13322	13322	12408	11955	11580	10115	10115	24,073
sample_42	sample	18113	18113	18113	16862	16335	15728	13850	13850	23,536
sample_43	sample	14457	14457	14457	13448	13024	12574	10853	10853	24,929
sample_44	sample	11003	11003	11003	10244	9899	9407	7899	7899	28,21
sample_45	sample	15672	15672	15672	14533	14116	13566	11730	11730	25,153
sample_46	sample	15226	15226	15226	14170	13767	13380	11560	11560	24,077
sample_47	sample	15843	15843	15843	14673	14171	13719	12073	12073	23,796
sample_48	sample	14102	14102	14102	13102	12714	12232	10796	10796	23,443
sample_49	sample	15678	15678	15678	14486	13968	13506	11400	11400	27,287
sample_50	sample	13188	13188	13188	12112	11844	11336	10295	10295	21,937
sample_51	sample	10867	10867	10867	10084	9811	9447	8418	8418	22,536
sample_52	sample	11903	11903	11903	11121	10812	10523	9364	9364	21,331
sample_53	sample	16761	16761	16761	15579	14953	14548	12281	12281	26,729
sample_54	sample	11192	11192	11192	10317	10044	9630	8667	8667	22,561
sample_55	sample	13702	13702	13702	12768	12370	11947	10379	10379	24,252
sample_56	sample	12666	12666	12666	11777	11474	11031	9505	9505	24,957
sample_57	sample	14922	14922	14922	13853	13349	12919	11175	11175	25,111
sample_58	sample	15689	15689	15689	14640	14193	13658	11698	11698	25,438
sample_59	sample	11237	11237	11237	10425	10057	9619	8211	8211	26,929
sample_60	sample	13804	13804	13804	12878	12369	11915	10184	10184	26,224
sample_61	sample	14166	14166	14166	12212	11777	11291	9707	9707	31,477
sample_62	sample	9882	9882	9882	9220	8833	8490	7244	7244	26,695
sample_63	sample	14468	14468	14468	13449	13023	12566	10647	10647	26,41
sample_64	sample	13034	13034	13034	12147	11758	11446	10123	10123	22,334
sample_65	sample	14345	14345	14345	13322	12944	12472	10972	10972	23,513
sample_66	sample	11019	11019	11019	10217	9900	9555	8326	8326	24,44
sample_67	sample	18078	18078	18078	16778	16227	15706	13815	13815	23,581
sample_68	sample	14672	14672	14672	13623	13239	12724	11295	11295	23,017
sample_69	sample	15618	15618	15618	14540	13936	13497	11367	11367	27,219
sample_70	sample	12166	12166	12166	11344	10929	10549	9016	9016	25,892
sample_71	sample	25117	25117	25117	23337	22828	22139	19718	19718	21,495
sample_72	sample	14219	14219	14219	13245	12974	12551	11337	11337	20,269
sample_73	sample	17273	17273	17273	15995	15419	14910	12644	12644	26,799

sample_74	sample	13653	13653	13653	12760	12431	12046	10727	10727	21,431
sample_75	sample	14080	14080	14080	13064	12665	12229	10904	10904	22,557
sample_76	sample	19453	19453	19453	18192	17649	16987	14754	14754	24,156
sample_77	sample	12929	12929	12929	12037	11726	11357	9616	9616	25,625
sample_78	sample	15064	15064	15064	14014	13559	13159	11270	11270	25,186
sample_79	sample	15777	15777	15777	14665	14237	13823	11844	11844	24,929
sample_80	sample	16627	16627	16627	15464	14992	14516	12784	12784	23,113
sample_81	sample	23958	23958	23958	22397	21725	21007	17338	17338	27,632
sample_82	sample	18652	18652	18652	17362	16807	16272	14386	14386	22,872
sample_83	sample	14827	14827	14827	13758	13299	12804	10426	10426	29,682
sample_84	sample	19267	19267	19267	17981	17435	16843	14659	14659	23,917
sample_85	sample	8072	8072	8072	7505	7300	7108	6641	6641	17,728
sample_86	sample	13420	13420	13420	12483	12125	11746	10078	10078	24,903
sample_87	sample	21365	21365	21365	19823	19227	18417	16109	16109	24,601
sample_88	sample	13931	13931	13931	12955	12691	12113	11090	11090	20,393
sample_89	sample	11525	11525	11525	10755	10421	9956	8619	8619	25,215
sample_90	sample	8999	8999	8999	8407	8155	7901	7213	7213	19,847
sample_91	sample	13028	13028	13028	12147	11872	11479	10102	10102	22,459
sample_92	sample	25664	25664	25664	23920	23321	22663	20291	20291	20,936
sample_93	sample	16227	16227	16227	15163	14684	14152	12198	12198	24,829
sample_94	sample	10826	10826	10826	10029	9606	9324	8076	8076	25,402
sample_95	sample	18110	18110	18110	16825	16385	15865	14169	14169	21,761
sample_96	sample	12124	12124	12124	11306	10978	10634	9310	9310	23,21
sample_97	sample	6505	6505	6505	6097	5775	5646	4631	4631	28,809
sample_98	sample	13541	13541	13541	12652	12333	11730	10478	10478	22,62
sample_99	sample	20030	20030	20030	18661	18228	17490	15199	15199	24,119
sample_100	sample	18796	18796	18796	17465	16994	16597	14590	14590	22,377
sample_101	sample	12525	12525	12525	11660	11273	10871	9255	9255	26,108
sample_102	sample	17590	17590	17590	16386	15872	15295	12967	12967	26,282
sample_103	sample	4637	4637	4637	4293	4116	3998	3523	3523	24,024
sample_104	sample	21940	21940	21940	20399	19868	19144	16896	16896	22,99
sample_105	sample	17142	17142	17142	15936	15444	14867	12589	12589	26,56
sample_106	sample	15106	15106	15106	14024	13519	13084	11223	11223	25,705
sample_107	sample	15691	15691	15691	14592	14000	13528	11020	11020	29,769
sample_108	sample	14657	14657	14657	13651	13122	12690	10683	10683	27,113
sample_109	sample	13638	13638	13638	12707	12410	11979	10617	10617	22,151
sample_110	sample	10736	10736	10736	9926	9629	9278	8181	8181	23,798
sample_111	sample	11015	11015	11015	10312	9977	9708	8818	8818	19,946
sample_112	sample	11488	11488	11488	10628	10252	9984	8667	8667	24,556
sample_113	sample	13313	13313	13313	12398	11934	11525	9794	9794	26,433
sample_114	sample	18776	18776	18776	17438	16832	16223	13668	13668	27,205
sample_115	sample	17038	17038	17038	15867	15262	14861	12527	12527	26,476
sample_116	sample	10991	10991	10991	10209	9886	9504	8332	8332	24,193
sample_117	sample	13625	13625	13625	12624	12224	11824	10348	10348	24,051
sample_118	sample	17333	17333	17333	16129	15643	15171	13614	13614	21,456
sample_119	sample	20038	20038	20038	18678	18018	17277	14390	14390	28,186
sample_120	sample	17540	17540	17540	16341	15791	15263	13154	13154	25,006
sample_121	sample	22703	22703	22703	21101	20685	20157	18153	18153	20,041
sample_122	sample	10554	10554	10554	9792	9426	9075	7342	7342	30,434
sample_123	sample	14363	14363	14363	13392	12920	12602	10524	10524	26,728
sample_124	sample	9009	9009	9009	8414	8194	7911	7242	7242	19,614
sample_125	sample	21447	21447	21447	19866	19234	18593	15857	15857	26,064
sample_126	sample	14802	14802	14802	13804	13400	12981	11851	11851	19,936
sample_127	sample	17202	17202	17202	16017	15560	14996	13186	13186	23,346
sample_128	sample	13072	13072	13072	12151	11631	11286	9232	9232	29,376
sample_129	sample	22803	22803	22803	21277	20727	20038	17243	17243	24,383
sample_130	sample	17093	17093	17093	15892	15398	14888	13001	13001	23,94
sample_131	sample	5392	5392	5392	5028	4869	4736	4393	4393	18,527
sample_132	sample	13385	13385	13385	12453	11918	11616	9624	9624	28,099
sample_133	sample	4526	4526	4526	4204	4001	3845	3493	3493	22,824
sample_134	sample	13487	13487	13487	12622	12229	11869	10272	10272	23,838
sample_135	sample	20907	20907	20907	19508	18825	18219	14881	14881	28,823
sample_136	sample	14736	14736	14736	13737	13287	12834	11099	11099	24,681
sample_137	sample	18959	18959	18959	17693	17212	16625	14148	14148	25,376
sample_138	sample	11093	11093	11093	10299	9993	9646	8394	8394	24,331
sample_139	sample	17561	17561	17561	16348	15864	15320	13011	13011	25,91

sample_140	sample	9058	9058	9058	8434	8154	7814	6926	6926	23,537
sample_141	sample	9357	9357	9357	8729	8448	8108	7103	7103	24,089
sample_142	sample	12748	12748	12748	11896	11450	11005	9574	9574	24,898
sample_143	sample	13955	13955	13955	13043	12587	12109	10064	10064	27,882
sample_144	sample	23738	23738	23738	22106	21710	21214	20261	20261	14,647
sample_145	sample	14136	14136	14136	13096	12677	12180	10443	10443	26,125
sample_146	sample	11238	11238	11238	10411	10118	9631	8375	8375	25,476
sample_147	sample	16267	16267	16267	15140	14626	13971	11794	11794	27,497
sample_148	sample	15532	15532	15532	14474	14059	13617	11516	11516	25,856
sample_149	sample	10407	10407	10407	9666	9356	9081	8239	8239	20,832
sample_150	sample	10432	10432	10432	9731	9472	9163	8011	8011	23,207
sample_151	sample	16731	16731	16731	15610	15163	14739	13162	13162	21,332
sample_152	sample	15224	15224	15224	14161	13849	13245	12091	12091	20,579
sample_153	sample	13017	13017	13017	12097	11698	11345	9704	9704	25,451
sample_154	sample	8518	8518	8518	7900	7664	7350	6482	6482	23,902
sample_155	sample	17212	17212	17212	16076	15587	15047	13007	13007	24,431
sample_156	sample	16834	16834	16834	15631	15176	14659	12903	12903	23,352
sample_157	sample	17317	17317	17317	16129	15645	15138	13460	13460	22,273
sample_158	sample	13062	13062	13062	12021	11676	11269	9967	9967	23,695
sample_159	sample	13737	13737	13737	12873	12596	12134	11210	11210	18,396
sample_160	sample	13604	13604	13604	12675	12364	11927	10544	10544	22,493
sample_161	sample	12547	12547	12547	11667	11268	10949	9443	9443	24,739
sample_162	sample	19031	19031	19031	17685	17088	16555	13540	13540	28,853
sample_163	sample	10857	10857	10857	10081	9842	9436	8568	8568	21,083
sample_164	sample	12590	12590	12590	11734	11279	10952	9321	9321	25,965
sample_165	sample	14305	14305	14305	13359	12855	12522	10407	10407	27,249
sample_166	sample	5209	5209	5209	4835	4678	4597	4288	4288	17,681
sample_167	sample	11737	11737	11737	10952	10556	10315	8661	8661	26,208
sample_168	sample	13983	13983	13983	12125	11900	11371	10560	10560	24,48
sample_169	sample	9213	9213	9213	8544	8328	8011	7373	7373	19,972
sample_170	sample	10123	10123	10123	9393	9031	8779	7329	7329	27,601
sample_171	sample	10008	10008	10008	9305	8980	8703	7531	7531	24,75
sample_172	sample	16745	16745	16745	15636	15160	14553	12520	12520	25,231
sample_173	sample	13331	13331	13331	12392	12062	11559	10367	10367	22,234
sample_174	sample	16758	16758	16758	15616	15063	14589	12101	12101	27,79
sample_175	sample	15475	15475	15475	14361	13966	13479	11919	11919	22,979
sample_176	sample	21168	21168	21168	19692	19123	18435	15976	15976	24,528
sample_177	sample	17519	17519	17519	16339	15920	15375	13167	13167	24,842
sample_178	sample	15287	15287	15287	14282	13755	13317	10898	10898	28,711
sample_179	sample	13918	13918	13918	12970	12540	12032	10299	10299	26,002
sample_180	sample	7502	7502	7502	6997	6720	6557	5570	5570	25,753
sample_181	sample	14321	14321	14321	13352	12892	12476	10606	10606	25,941
sample_182	sample	21457	21457	21457	19935	19447	18722	16414	16414	23,503
sample_183	sample	13805	13805	13805	12855	12389	12022	10245	10245	25,788
sample_184	sample	14299	14299	14299	13354	12934	12609	10887	10887	23,862
sample_185	sample	22361	22361	22361	20770	20176	19414	16241	16241	27,369
sample_186	sample	17751	17751	17751	16523	16125	15594	13298	13298	25,086
sample_187	sample	17158	17158	17158	15999	15540	15023	13023	13023	24,1
sample_188	sample	15419	15419	15419	14286	13905	13502	11975	11975	22,336
sample_189	sample	11139	11139	11139	9853	9566	9234	8452	8452	24,122
sample_190	sample	10610	10610	10610	9875	9589	9391	8399	8399	20,839
sample_191	sample	18626	18626	18626	17359	16867	16317	14487	14487	22,222
sample_192	sample	12733	12733	12733	11840	11499	11191	10116	10116	20,553
sample_193	sample	15933	15933	15933	14884	14291	13636	11114	11114	30,245
sample_194	sample	12276	12276	12276	11454	11088	10682	9419	9419	23,273
sample_195	sample	20959	20959	20959	19424	18638	18163	15135	15135	27,788
sample_196	sample	11365	11365	11365	10521	10253	9872	9214	9214	18,927
sample_197	sample	12418	12418	12418	11579	11248	10744	9484	9484	23,627
sample_198	sample	11905	11905	11905	11090	10841	10439	9840	9840	17,346
sample_199	sample	14704	14704	14704	13641	13148	12846	11113	11113	24,422
sample_200	sample	13066	13066	13066	12155	11819	11336	9548	9548	26,925
sample_201	sample	10965	10965	10965	10191	9920	9623	8594	8594	21,623
sample_202	sample	16443	16443	16443	15304	14893	14321	12511	12511	23,913
sample_203	sample	15190	15190	15190	14092	13701	13315	11843	11843	22,034
sample_204	sample	4751	4751	4751	4397	4245	4101	3774	3774	20,564
sample_205	sample	2167	2167	2167	1999	1872	1801	1583	1583	26,95

sample_206	sample	15079	15079	15079	14042	13582	13187	11377	11377	24,551
sample_207	sample	9430	9430	9430	8733	8462	8192	7077	7077	24,952
sample_208	sample	17305	17305	17305	16118	15614	15055	12873	12873	25,611
sample_209	sample	10606	10606	10606	9719	9309	8994	7617	7617	28,182
sample_210	sample	11225	11225	11225	10466	10143	9772	8781	8781	21,773
sample_211	sample	19988	19988	19988	18622	18065	17458	15244	15244	23,734
sample_212	sample	17981	17981	17981	16778	16392	15788	14212	14212	20,961
sample_213	sample	15014	15014	15014	13965	13523	12957	11323	11323	24,584
sample_214	sample	18562	18562	18562	17315	16775	16025	14173	14173	23,645
sample_215	sample	12485	12485	12485	11607	11296	10847	9671	9671	22,539
sample_216	sample	13065	13065	13065	12225	11732	11378	9492	9492	27,348
sample_217	sample	19426	19426	19426	18020	17645	16889	15095	15095	22,295
sample_218	sample	13141	13141	13141	12272	11899	11474	9810	9810	25,348
sample_219	sample	11183	11183	11183	10371	10216	9786	9257	9257	17,223
sample_220	sample	9620	9620	9620	8897	8675	8308	7672	7672	20,249
sample_221	sample	15333	15333	15333	14262	13894	13316	12012	12012	21,659
sample_222	sample	14309	14309	14309	13348	12902	12485	10504	10504	26,592
sample_223	sample	13182	13182	13182	12229	11886	11511	10016	10016	24,018
sample_224	sample	20024	20024	20024	18657	17923	17222	13723	13723	31,467
sample_225	sample	17696	17696	17696	16460	15915	15273	12967	12967	26,724
sample_226	sample	13853	13853	13853	12857	12491	12114	11059	11059	20,169
sample_227	sample	13413	13413	13413	12504	12065	11581	9759	9759	27,242
sample_228	sample	13296	13296	13296	12334	11893	11515	9773	9773	26,497
sample_229	sample	13698	13698	13698	12848	12299	11942	9769	9769	28,683
sample_230	sample	17126	17126	17126	15911	15548	14920	13279	13279	22,463
sample_231	sample	16813	16813	16813	15582	15163	14566	12620	12620	24,939
sample_232	sample	20535	20535	20535	19101	18604	17778	15194	15194	26,009
sample_233	sample	8093	8093	8093	7567	7335	7041	6391	6391	21,031
sample_234	sample	11351	11351	11351	10532	10281	9928	9038	9038	20,377
sample_235	sample	17681	17681	17681	16422	15786	15242	13169	13169	25,519
sample_236	sample	19436	19436	19436	18083	17378	16817	14022	14022	27,856
sample_237	sample	14258	14258	14258	13281	12792	12475	10667	10667	25,186
sample_238	sample	12753	12753	12753	11850	11489	11132	9415	9415	26,174
sample_239	sample	1526	1526	1526	1429	1358	1294	1154	1154	24,377
sample_240	sample	6728	6728	6728	6273	6027	5818	5353	5353	20,437
sample_241	sample	15763	15763	15763	14651	14113	13623	11473	11473	27,216
sample_242	sample	16529	16529	16529	15389	14872	14316	12193	12193	26,233
sample_243	sample	14238	14238	14238	13290	12910	12493	10541	10541	25,966
sample_244	sample	18323	18323	18323	17127	16622	16147	13681	13681	25,334
sample_245	sample	12751	12751	12751	11800	11327	11003	9177	9177	28,029
sample_246	sample	14074	14074	14074	13061	12588	12167	10343	10343	26,51
sample_247	sample	15740	15740	15740	14627	14138	13643	11824	11824	24,879
sample_248	sample	14924	14924	14924	13854	13427	12960	11222	11222	24,806
sample_249	sample	16302	16302	16302	15232	14778	14157	12221	12221	25,034
sample_250	sample	20230	20230	20230	18912	18232	17617	14318	14318	29,224
sample_251	sample	14765	14765	14765	13755	13376	12864	11131	11131	24,612
sample_252	sample	21524	21524	21524	20006	19647	18815	17023	17023	20,912
sample_253	sample	17572	17572	17572	16327	15854	15248	13262	13262	24,528
sample_254	sample	2359	2359	2359	2182	2095	2009	1786	1786	24,29
sample_255	sample	17798	17798	17798	16628	16241	15623	14200	14200	20,216
sample_256	sample	13093	13093	13093	12218	11818	11390	9736	9736	25,64
sample_257	sample	12984	12984	12984	12051	11569	11191	9561	9561	26,363
sample_258	sample	18144	18144	18144	16939	16383	15828	13494	13494	25,628
sample_259	sample	9114	9114	9114	8455	8092	7895	6772	6772	25,697
sample_260	sample	16113	16113	16113	14989	14578	14052	12356	12356	23,317
sample_261	sample	11983	11983	11983	11117	10834	10437	9477	9477	20,913
sample_262	sample	15072	15072	15072	14020	13603	13228	11119	11119	26,227
sample_263	sample	18078	18078	18078	16793	16339	15690	14018	14018	22,458
sample_264	sample	7939	7939	7939	7412	7135	6906	6055	6055	23,731
sample_265	sample	9890	9890	9890	9226	8979	8628	7976	7976	19,353
sample_266	sample	15693	15693	15693	14612	14196	13756	12025	12025	23,373
sample_267	sample	16277	16277	16277	15132	14769	14259	12601	12601	22,584
sample_268	sample	16200	16200	16200	15089	14608	14151	12394	12394	23,494
sample_269	sample	11162	11162	11162	10389	10040	9557	8407	8407	24,682
sample_270	sample	17086	17086	17086	15837	15330	14783	12030	12030	29,591
sample_271	sample	20572	20572	20572	19218	18704	17998	15669	15669	23,833

sample_272	sample	16518	16518	16518	15369	15042	14500	13311	13311	19,415
sample_273	sample	13483	13483	13483	12622	12231	11888	9911	9911	26,493
sample_274	sample	16895	16895	16895	15693	15105	14775	12562	12562	25,647
sample_275	sample	9036	9036	9036	8420	8090	7809	6627	6627	26,66
sample_276	sample	14594	14594	14594	13581	13143	12675	11118	11118	23,818
sample_277	sample	19185	19185	19185	17934	17236	16611	14063	14063	26,698
sample_278	sample	10059	10059	10059	9378	9149	8734	7533	7533	25,112
sample_279	sample	14899	14899	14899	13956	13455	13066	11206	11206	24,787
sample_280	sample	13109	13109	13109	12162	11760	11340	9685	9685	26,119
sample_281	sample	18822	18822	18822	17435	16933	16261	14267	14267	24,2
sample_282	sample	15206	15206	15206	14108	13636	13141	11551	11551	24,037
sample_283	sample	13187	13187	13187	12308	11928	11430	10239	10239	22,355
sample_284	sample	13558	13558	13558	12573	12246	11787	10633	10633	21,574
sample_285	sample	32661	32661	32661	30388	29653	28701	25680	25680	21,374
sample_286	sample	11330	11330	11330	10593	10288	10050	9115	9115	19,55
sample_287	sample	15506	15506	15506	14424	13928	13494	11654	11654	24,842
sample_288	sample	12840	12840	12840	11944	11470	11111	9116	9116	29,003
sample_289	sample	20171	20171	20171	18804	18329	17566	15549	15549	22,914
sample_290	sample	15732	15732	15732	14664	14205	13700	11704	11704	25,604
sample_291	sample	15398	15398	15398	14380	13928	13395	11425	11425	25,802
sample_292	sample	13667	13667	13667	12757	12359	11954	10412	10412	23,816
sample_293	sample	13830	13830	13830	12874	12463	12040	10868	10868	21,417
sample_294	sample	12039	12039	12039	11244	10854	10413	8889	8889	26,165
sample_295	sample	13779	13779	13779	12860	12429	12046	10259	10259	25,546
sample_296	sample	12881	12881	12881	12028	11774	11437	10581	10581	17,856
sample_297	sample	14675	14675	14675	13639	13174	12712	10674	10674	27,264
sample_298	sample	15054	15054	15054	14024	13505	13075	11304	11304	24,91
sample_299	sample	14963	14963	14963	13883	13394	13064	10867	10867	27,374
sample_300	sample	17112	17112	17112	15974	15470	14835	13009	13009	23,977
sample_301	sample	19148	19148	19148	17723	17215	16433	14360	14360	25,005
sample_302	sample	5732	5732	5732	5360	5212	5074	4746	4746	17,202
sample_303	sample	11093	11093	11093	10296	9880	9623	7961	7961	28,234
sample_304	sample	16905	16905	16905	15779	15500	14961	13261	13261	21,556
sample_305	sample	16130	16130	16130	15028	14530	14133	12118	12118	24,873
sample_306	sample	14289	14289	14289	13323	13011	12533	11128	11128	22,122
sample_307	sample	15516	15516	15516	14412	14036	13515	11751	11751	24,265
sample_308	sample	17020	17020	17020	15800	15406	14783	13226	13226	22,291
sample_309	sample	3671	3671	3671	3414	3289	3208	2992	2992	18,496
sample_310	sample	13867	13867	13867	12885	12541	12122	10960	10960	20,963
sample_311	sample	19876	19876	19876	18508	17951	17270	15090	15090	24,079
sample_312	sample	2094	2094	2094	1953	1828	1761	1496	1496	28,558
sample_313	sample	12894	12894	12894	12003	11624	11331	9809	9809	23,926
sample_314	sample	12047	12047	12047	11282	10782	10412	8618	8618	28,464
sample_315	sample	17636	17636	17636	16391	15943	15334	13447	13447	23,753
sample_316	sample	15492	15492	15492	14429	13871	13435	11615	11615	25,026
sample_317	sample	16606	16606	16606	15479	14975	14543	12582	12582	24,232
sample_318	sample	15859	15859	15859	14755	14350	13900	12173	12173	23,242
sample_319	sample	9775	9775	9775	9083	8773	8390	7495	7495	23,325
sample_320	sample	12393	12393	12393	11500	11087	10753	9344	9344	24,603
sample_321	sample	11883	11883	11883	11002	10617	10376	9065	9065	23,715
sample_322	sample	11284	11284	11284	10520	10215	9905	8855	8855	21,526
sample_323	sample	17274	17274	17274	16063	15550	14958	12536	12536	27,429
sample_324	sample	672	672	672	629	557	506	440	440	34,524
sample_325	sample	17881	17881	17881	16686	16135	15651	13622	13622	23,819
sample_326	sample	10151	10151	10151	9416	9113	8846	7635	7635	24,786
sample_327	sample	11549	11549	11549	10782	10426	10100	8573	8573	25,768
sample_328	sample	13041	13041	13041	12143	11716	11428	9858	9858	24,408
sample_329	sample	15477	15477	15477	14473	14070	13515	11585	11585	25,147
sample_330	sample	12699	12699	12699	11824	11438	11077	9423	9423	25,797
sample_331	sample	21228	21228	21228	19762	19287	18727	16046	16046	24,411
sample_332	sample	13174	13174	13174	11979	11670	11137	9870	9870	25,08
sample_333	sample	15739	15739	15739	14658	14305	13847	11964	11964	23,985
sample_334	sample	9703	9703	9703	8971	8691	8382	7549	7549	22,199
sample_335	sample	10695	10695	10695	9952	9606	9269	7735	7735	27,676
sample_336	sample	13230	13230	13230	12316	11830	11509	9894	9894	25,215
sample_337	sample	16580	16580	16580	15467	14950	14353	11947	11947	27,943

sample_338	sample	17786	17786	17786	16596	16086	15536	13514	13514	24,019
sample_339	sample	9206	9206	9206	8578	8342	8092	7203	7203	21,758
sample_340	sample	11230	11230	11230	10529	10144	9805	8263	8263	26,42
sample_341	sample	11897	11897	11897	11115	10760	10407	8524	8524	28,352
sample_342	sample	15664	15664	15664	14616	14171	13696	11758	11758	24,936
sample_343	sample	12995	12995	12995	12076	11661	11363	9708	9708	25,294
sample_344	sample	15677	15677	15677	14614	14125	13568	11550	11550	26,325
sample_345	sample	11128	11128	11128	10328	9940	9572	8149	8149	26,77
sample_346	sample	19173	19173	19173	17858	17355	16771	14851	14851	22,542
sample_347	sample	11059	11059	11059	10336	9969	9699	8514	8514	23,013
sample_348	sample	16623	16623	16623	15455	15036	14547	13135	13135	20,983
sample_349	sample	13879	13879	13879	12918	12450	12035	10615	10615	23,518
sample_350	sample	13383	13383	13383	12468	11936	11645	9929	9929	25,809
sample_351	sample	19124	19124	19124	17706	17201	16668	14682	14682	23,227
sample_352	sample	11670	11670	11670	10818	10416	10023	8467	8467	27,446
sample_353	sample	16822	16822	16822	15676	15289	14837	12914	12914	23,231
sample_354	sample	13350	13350	13350	12475	11948	11615	9815	9815	26,479
sample_355	sample	12661	12661	12661	11787	11201	10888	8491	8491	32,936
sample_356	sample	19017	19017	19017	17745	17265	16582	14110	14110	25,803
sample_357	sample	9310	9310	9310	8652	8311	8033	6940	6940	25,456
sample_358	sample	9368	9368	9368	8721	8511	8215	7324	7324	21,819
sample_359	sample	11343	11343	11343	10557	10219	9909	8602	8602	24,165
sample_360	sample	17940	17940	17940	16766	16200	15707	13540	13540	24,526
sample_361	sample	12138	12138	12138	11249	10861	10442	8740	8740	27,995
sample_362	sample	15984	15984	15984	14781	14366	13832	12092	12092	24,349
sample_363	sample	20773	20773	20773	19012	18587	17860	15776	15776	24,055
sample_364	sample	8785	8785	8785	8223	8029	7780	7283	7283	17,097
